# Genome-Wide Association and Trans-ethnic Meta-Analysis for Advanced Diabetic Kidney Disease: Family Investigation of Nephropathy and Diabetes (FIND)

**DOI:** 10.1371/journal.pgen.1005352

**Published:** 2015-08-25

**Authors:** Sudha K. Iyengar, John R. Sedor, Barry I. Freedman, W. H. Linda Kao, Matthias Kretzler, Benjamin J. Keller, Hanna E. Abboud, Sharon G. Adler, Lyle G. Best, Donald W. Bowden, Allison Burlock, Yii-Der Ida Chen, Shelley A. Cole, Mary E. Comeau, Jeffrey M. Curtis, Jasmin Divers, Christiane Drechsler, Ravi Duggirala, Robert C. Elston, Xiuqing Guo, Huateng Huang, Michael Marcus Hoffmann, Barbara V. Howard, Eli Ipp, Paul L. Kimmel, Michael J. Klag, William C. Knowler, Orly F. Kohn, Tennille S. Leak, David J. Leehey, Man Li, Alka Malhotra, Winfried März, Viji Nair, Robert G. Nelson, Susanne B. Nicholas, Stephen J. O’Brien, Madeleine V. Pahl, Rulan S. Parekh, Marcus G. Pezzolesi, Rebekah S. Rasooly, Charles N. Rotimi, Jerome I. Rotter, Jeffrey R. Schelling, Michael F. Seldin, Vallabh O. Shah, Adam M. Smiles, Michael W. Smith, Kent D. Taylor, Farook Thameem, Denyse P. Thornley-Brown, Barbara J. Truitt, Christoph Wanner, E. Jennifer Weil, Cheryl A. Winkler, Philip G. Zager, Robert P. Igo, Robert L. Hanson, Carl D. Langefeld, Family Investigation of Nephropathy and Diabetes (FIND)

**Affiliations:** 1 Department of Epidemiology & Biostatistics, Case Western Reserve University, Cleveland, Ohio, United States of America; 2 Departments of Medicine, Case Western Reserve University, Cleveland, Ohio, United States of America; 3 Departments of Physiology and Biophysics, Case Western Reserve University, Cleveland, Ohio, United States of America; 4 Department of Internal Medicine, Section on Nephrology, Wake Forest School of Medicine, Winston-Salem, North Carolina, United States of America; 5 Department of Epidemiology and Medicine, John Hopkins University, Baltimore, Maryland, United States of America; 6 Department of Internal Medicine/Nephrology, University of Michigan, Ann Arbor, Michigan, United States of America; 7 Department of Medicine/Nephrology, The University of Texas Health Science Center, San Antonio, Texas, United States of America; 8 Department of Medicine, Division of Nephrology and Hypertension, Harbor-UCLA Medical Center, Torrance, California, United States of America; 9 Missouri Breaks Industries Research, Timber Lake, South Dakota, United States of America; 10 Department of Biochemistry, Center for Human Genomics, Wake Forest School of Medicine, Winston-Salem, North Carolina, United States of America; 11 The Institute for Translational Genomics and Population Sciences, Harbor-UCLA Medical Center, Torrance, California, United States of America; 12 Department of Genetics, Texas Biomedical Research Institute, San Antonio, Texas, United States of America; 13 Center for Public Health Genomics and Department of Biostatistical Sciences, Division of Public Health Sciences, Wake Forest School of Medicine, Winston-Salem, United States of America; 14 National Institutes of Diabetes and Digestive and Kidney Diseases, National Institutes of Health, Phoenix, Arizona, United States of America; 15 University Hospital Würzburg, Renal Division and Comprehensive Heart Failure Center, Würzburg, Germany; 16 Department of Ecology and Evolutionary Biology, University of Michigan, Ann Arbor, Michigan, United States of America; 17 Department of Clinical Chemistry, University Medical Center, Freiburg, Germany; 18 MedStar Health Research Institute, Hyattsville, Maryland, United States of America; 19 Department of Medicine, Section of Diabetes and Metabolism, Harbor-UCLA Medical Center, Torrance, California, United States of America; 20 Division of Kidney, Urologic, and Hematologic Diseases, National Institute of Diabetes and Digestive and Kidney Diseases, Bethesda, Maryland, United States of America; 21 Johns Hopkins Bloomberg School of Public Health, Johns Hopkins School of Medicine, Baltimore, Maryland, United States of America; 22 Department of Medicine, University of Chicago Medicine, Chicago, Illinois, United States of America; 23 Department of Medicine, Loyola School of Medicine, Maywood, Illinois, United States of America; 24 Department of Epidemiology, Johns Hopkins Bloomberg School of Public Health, Baltimore, Maryland, United States of America; 25 Heidelberg University and Synlab Academy, University of Graz, Graz, Austria; 26 Department of Medicine, University of California, Los Angeles, Los Angeles, California, United States of America; 27 Theodosius Dobzhansky Center for Genome Bioinformatics, St. Petersburg, Russia, and Oceanographic Center, Nova Southeastern University, Ft. Lauderdale, Florida, United States of America; 28 Department of Medicine, University of California, Irvine, Irvine, California, United States of America; 29 Departments of Paediatrics and Medicine, Hospital for Sick Children, University Health Network and the University of Toronto, Toronto, Ontario, Canada; 30 Department of Medicine, Joslin Diabetes Center, Harvard Medical School, Boston, Massachusetts, United States of America; 31 National Institute of Diabetes and Digestive Disease, National Institutes of Health, Bethesda, Maryland, United States of America; 32 Center for Research on Genomics and Global Health, Bethesda, Maryland, United States of America; 33 Department of Biochemistry and Molecular Medicine, UC Davis School of Medicine, Davis, California, United States of America; 34 Department of Biochemistry & Molecular Biology, University of New Mexico, Albuquerque, New Mexico, United States of America; 35 Joslin Diabetes Center, Section on Genetics and Epidemiology, Boston, Massachusetts, United States of America; 36 National Human Genome Research Institute, Rockville, Maryland, United States of America; 37 Department of Medicine, The University of Texas Health Science Center, San Antonio, Texas, United States of America; 38 Nephrology, University of Alabama Birmingham, Birmingham, Alabama, United States of America; 39 Department of Medicine, Division of Nephrology, University Hospital Würzburg, Würzburg, Germany; 40 Center for Cancer Research, National Cancer Institute, Frederick, Maryland, United States of America; 41 Department of Internal Medicine, University of New Mexico, Albuquerque, New Mexico, United States of America; Dartmouth College, UNITED STATES

## Abstract

Diabetic kidney disease (DKD) is the most common etiology of chronic kidney disease (CKD) in the industrialized world and accounts for much of the excess mortality in patients with diabetes mellitus. Approximately 45% of U.S. patients with incident end-stage kidney disease (ESKD) have DKD. Independent of glycemic control, DKD aggregates in families and has higher incidence rates in African, Mexican, and American Indian ancestral groups relative to European populations. The Family Investigation of Nephropathy and Diabetes (FIND) performed a genome-wide association study (GWAS) contrasting 6,197 unrelated individuals with advanced DKD with healthy and diabetic individuals lacking nephropathy of European American, African American, Mexican American, or American Indian ancestry. A large-scale replication and trans-ethnic meta-analysis included 7,539 additional European American, African American and American Indian DKD cases and non-nephropathy controls. Within ethnic group meta-analysis of discovery GWAS and replication set results identified genome-wide significant evidence for association between DKD and rs12523822 on chromosome 6q25.2 in American Indians (P = 5.74x10^-9^). The strongest signal of association in the trans-ethnic meta-analysis was with a SNP in strong linkage disequilibrium with rs12523822 (rs955333; P = 1.31x10^-8^), with directionally consistent results across ethnic groups. These 6q25.2 SNPs are located between the *SCAF8* and *CNKSR3* genes, a region with DKD relevant changes in gene expression and an eQTL with *IPCEF1*, a gene co-translated with *CNKSR3*. Several other SNPs demonstrated suggestive evidence of association with DKD, within and across populations. These data identify a novel DKD susceptibility locus with consistent directions of effect across diverse ancestral groups and provide insight into the genetic architecture of DKD.

## Author Summary

Type 2 diabetes is the most common cause of severe kidney disease worldwide and diabetic kidney disease (DKD) associates with premature death. Individuals of non-European ancestry have the highest burden of type 2 DKD; hence understanding the causes of DKD remains critical to reducing health disparities. Family studies demonstrate that genes regulate the onset and progression of DKD; however, identifying these genes has proven to be challenging. The Family Investigation of Diabetes and Nephropathy consortium (FIND) recruited a large multi-ethnic collection of individuals with type 2 diabetes with and without kidney disease in order to detect genes associated with DKD. FIND discovered and replicated a DKD-associated genetic locus on human chromosome 6q25.2 (rs955333) between the *SCAF8* and *CNKSR* genes. Findings were supported by significantly different expression of genes in this region from kidney tissue of subjects with, versus without DKD. The present findings identify a novel kidney disease susceptibility locus in individuals with type 2 diabetes which is consistent across subjects of differing ancestries. In addition, FIND results provide a rich catalogue of genetic variation in DKD patients for future research on the genetic architecture regulating this common and devastating disease.

## Introduction

Diabetic kidney disease (DKD) is a devastating complication in patients with diabetes mellitus (DM) and is associated with high risk for cardiovascular disease and death.[1,2] DKD is the leading cause of end-stage kidney disease (ESKD) requiring renal replacement therapy in developed nations; these procedures incur high healthcare costs with great personal, family and societal burden.[3] The prevalence of DKD continues to rise in the United States in proportion to the growing prevalence of DM. Unfortunately, intensification of glycemic, lipid and blood pressure control have not dramatically impacted the prevalence of DKD.[3,4] Hyperglycemia alone is insufficient to cause DKD. Genetic factors appear critical in its pathogenesis based upon variable incidence rates of DKD between population groups, aggregation of DKD-associated ESKD in families, and the highly heritable nature of diabetic renal histologic changes, estimated glomerular filtration rate (eGFR) and proteinuria.[5]

Genome-wide association studies (GWAS) have identified multiple loci for kidney function and chronic kidney disease (CKD) in population- and community-based cohorts, primarily of European ancestry.[6–10] However, CKD phenotypes in many studies included minimally to moderately reduced eGFR, not fully reflective of the progressive forms of CKD seen in kidney disease clinics. In early reports, published GWAS signals for DKD were equivocal, confounded by small sample sizes and failure to consistently replicate. Recently, the GEnetics of Nephropathy: an International Effort (GENIE) consortium identified genome-wide significant, replicated signals in a meta-analysis of over 12,000 type 1 (T1) DM patients with DKD of European ancestry.[9] Type 2 (T2) DM is far more prevalent than T1DM, accounting for 90% of cases worldwide and for the majority of prevalent cases of DKD. Relative to European Americans (EAs) with T2DM, African American (AA), American Indian (AI), and Mexican American (MA) patients with T2DM are disproportionately affected by severe DKD,[3] yet under-represented in genetic analyses. Defining the underlying genetic architecture responsible for advanced T2DM-associated kidney disease in multiple populations could provide critical insights into pathogenesis and identify new molecular targets for therapy. We report the results of a GWAS in AA, EA, MA, and AI patients with DKD enrolled in the National Institute of Diabetes and Digestive and Kidney Diseases (NIDDK)-sponsored “Family Investigation of Nephropathy and Diabetes” (FIND) [11] and the corresponding large replication study and trans-ethnic meta-analysis.

## Results and Discussion

Demographic characteristics of the Discovery and Find Large Replication study (FILR) samples that met FIND phenotype qualifications and genotype quality control (QC) are summarized in **Table 1** and **S1 Table**. The proportion of females, age at ESKD or enrollment, hemoglobin A1c and proportion with diabetic retinopathy (DR) varied by ancestry but was generally comparable between the Discovery and FILR samples within a specific ethnic population. Phenotypic differences among these populations and their genetic and DKD prevalence differences motivated the meta-analysis approach.

**Table 1:** Demographic characteristics of study populations.

**African American** ^**†**^							
	**FIND Samples**			**FILR Samples**			**Out of Study**
	**DKD Case**	**Diabetic Control**		**DKD Case**	**Diabetic Control**	**Non-Diabetic Control**	**Non-Diabetic Control**
**N**	633	277	**N**	950	50	1	1886
**Female**	347 (54.8%)	213 (76.9%)	**Female**	520 (54.7%)	31 (62.0%)	1 (100%)	938 (49.7%)
**Age, years**	56.8 ± 11.9	59.5 ± 11.0	**Age, years**	59.9 ± 11.0	64.3 ± 12.9		45.4 ± 13.2
**ESRD**	581 (91.8%)	0 (0%)	**ESRD**	930 (97.9%)	0 (0%)		0 (0%)
**HbA1c, %**	7.02 ± 1.79	7.83 ± 1.92	**HbA1c, %**	7.27 ± 1.90	8.41 ± 2.15	5.80	not available
**DM duration, y**	20.8 ± 10.8	17.8 ± 7.56	**DM duration, y**	19.4 ± 10.5	21.8 ± 8.39	-	-
**Retinopathy, %**	69.6%	14.2%	**Retinopathy, %**	75.4%	20.8%		not available
**American Indian**							
	**FIND Samples**			**FILR Samples**			
	**DKD Case**	**Diabetic Control**		**DKD Case**	**Diabetic Control**	**Non-Diabetic Control**	
**N**	538	319	**N**	471	340	486	
**Female**	323 (60.0%)	238 (74.6%)	**Female**	268 (56.9%)	248 (72.9%)	246 (50.6%)	
**Age, years**	55.9 ± 12.8	53.4 ± 12.4	**Age, years**	52.1 ± 12.6	54.7 ± 12.2	50.5 ± 11.1	
**ESRD**	369 (68.6%)	0 (0%)	**ESRD**	58 (12.3%)	0(0%)	0(0%)	
**HbA1c, %**	7.39 ± 2.14	7.72 ± 1.61	**HbA1c, %**	8.58 ± 2.46	8.18 ± 1.91		
**DM duration, y**	21.8 ± 10.2	17.9 ± 7.30	**DM duration, y**	15.3 ± 10.5	17.9 ± 6.9		
**Retinopathy, %**	72.3%	8.0%	**Retinopathy, %**	48.7%	10.4%	-	
**European Ancestry**							
	**FIND Samples**			**FILR Samples**			**Out of Study**
	**DKD Case**	**Diabetic Control**		**DKD Case**	**Diabetic Control**	**Non-Diabetic Control**	**Non-Diabetic Control**
**N**	342	404	**N**	582	205	23	2545
**Female**	165 (48.3%)	236 (58.4%)	**Female**	265 (45.5%)	94 (45.9%)	6 (26.1%)	1094 (43.0%)
**Age, years**	63.9 ± 11.3	61.7 ± 9.30	**Age, years**	66.1 ± 11.0	65.2 ± 9.57		51.1 ± 17.0
**ESRD**	284 (83.0%)	0 (0%)	**ESRD**	421 (72.3%)	0 (0%)	0 (0%)	0 (0%)
**HbA1c, %**	7.07 ± 1.75	7.53 ± 1.58	**HbA1c, %**	7.05 ± 1.50	6.94 ± 1.76	7.17 ± 1.00	not available
**DM duration, y**	21.8 ± 8.88	18.8 ± 8.94	**DM duration, y**	19.55 ± 10.6	17.0 ± 8.05	-	not available
**Retinopathy, %**	70.2%	9.4%	**Retinopathy, %**	64.5%	10.2%	-	not available
**Mexican Ancestry**							
	**FIND Samples**						
	**DKD Case**	**Diabetic Control**					
**N**	779	594	**N**				
**Female**	413 (53.0%)	414 (69.7%)	**Female**				
**Age, years**	59.2 ± 10.7	57.7 ± 9.83	**Age, years**				
**ESRD**	552 (70.9%)	0 (0%)	**ESRD**				
**HbA1c, %**	7.25 ± 1.75	8.28 ± 1.89	**HbA1c, %**				
**DM duration, y**	19.8 ± 8.50	15.8 ± 6.07	**DM duration, y**				
**Retinopathy, %**	81.9%	17.0%	**Retinopathy, %**				

**† In the African American sample, we obtained out-of-study samples from Wake Forest University and Howard University (see** Methods **). Their demographic characteristics are as follows: 931 DKD cases are 60.3% female, Age in years 61.6 ± 10.5, DM duration in years 19.7 ± 10.7; 92 Diabetic controls are all female, Age in years 55.1 ± 11.6, No Diabetes duration available; 1288 Non-Diabetic controls are 35.5% female, Age in years 48.0 ± 12.4. FILR–FIND Large Replication study.**

The principal component (PC) analysis identified PCs that genetically partitioned the Discovery sample into ancestry groups consistent with self-report. **S1 Fig** displays the two-dimensional partitioning via PC analysis with the boundaries for inclusion into the GWAS analysis. The logistic regression model that included the PCs as covariates reduced the inflation factor to nominal levels and combined with the P-P plot show no evidence of a systematic inflation (**S2 Fig**). In the replication, the inflation factor was λ = 1.05 using 278 AIMs. If we scale to 1,000 cases and 1,000 controls this would be λ_1000_ = 1.017, an appropriate inflation factor for the replication study. **S3 Fig** provides a summary of the statistical power analyses for the race-specific discovery, replication analysis, and meta-analyses. These calculations show that for risk-predisposing variants shared across ancestries the study has power >0.50 and 0.80 to detect odds ratios (OR) on the order of 1.06 to 1.15 for a minor allele frequency (MAF) = 0.45 and 1.30 to 1.36 for a MAF = 0.05, respectively. Further, leveraging the differences in linkage disequilibrium (LD) among the four ancestries, the study is powered to potentially reduce the size of the associated region via trans-ethnic mapping.

### Trans-ethnic Meta-Analysis Associations

**Fig 1:**
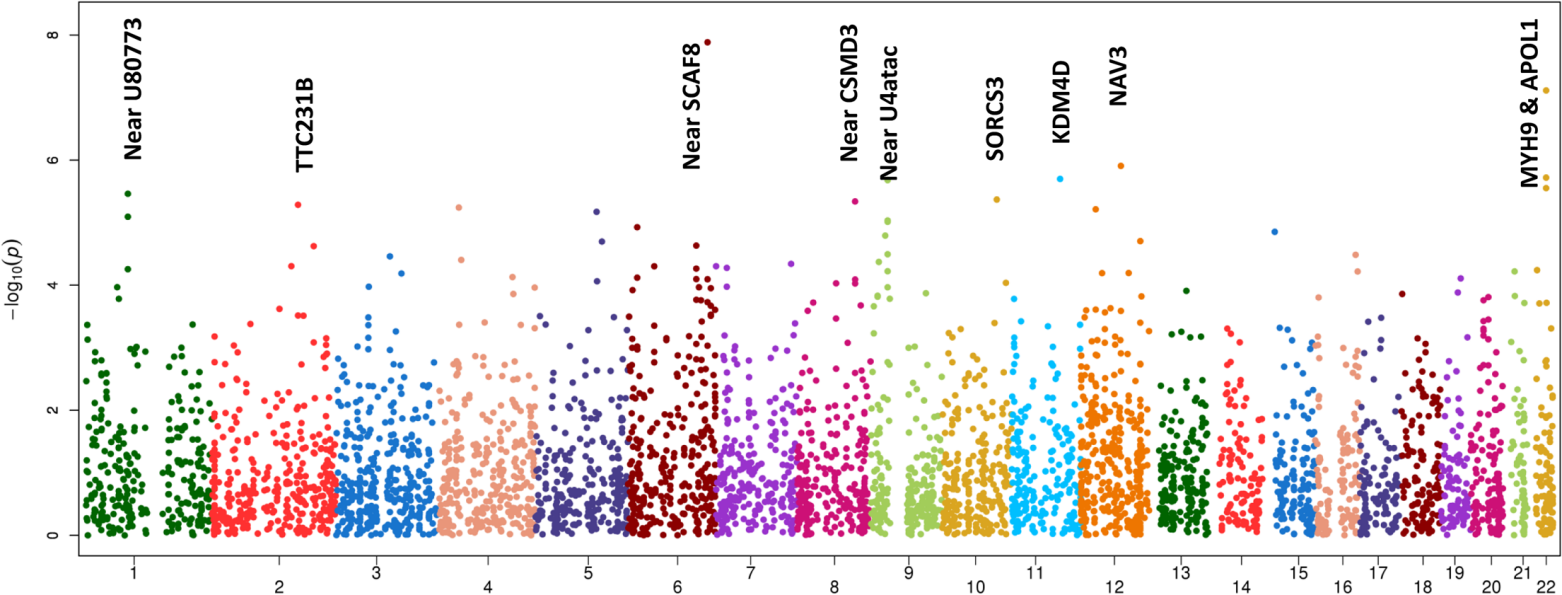
Manhattan plot of FIND GWAS meta-analysis associations across ancestries included in Discovery and Replication samples.

**Fig 2:**
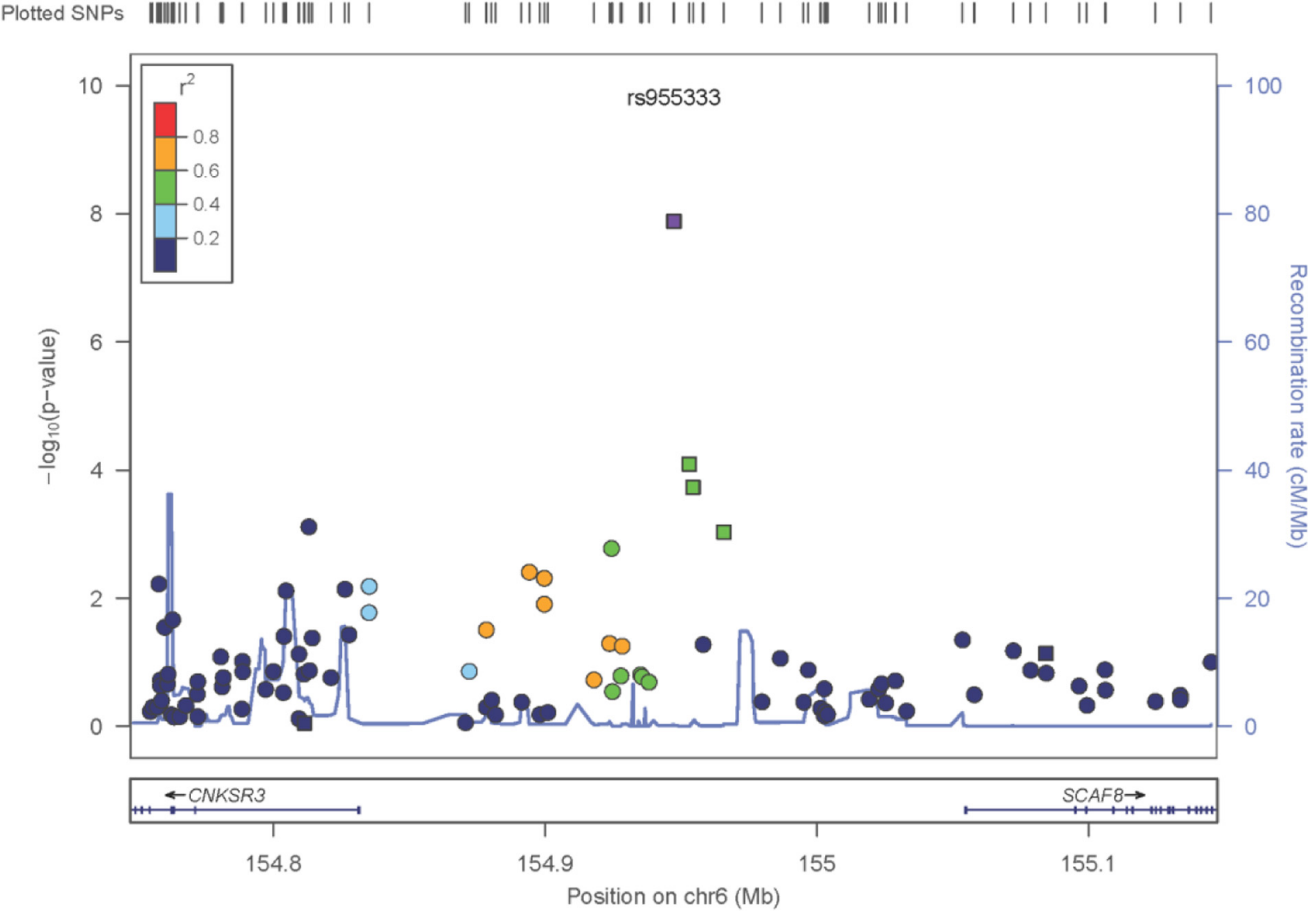
Zoom plot of the *SCAF8* gene region (trans-ancestry meta-analysis across Discovery and Replication samples). Squares denote SNPs in the Replication Study, Circles are SNPs that are only present in the GWAS, so the P-values shown reflect the GWAS Trans-Ancestry Meta-Analysis. The Ad-Mixed American population in 1000 Genomes was used for LD information.

The only locus that reached genome wide significance for DKD in the trans-ethnic meta-analysis encompassing all FIND Discovery and Replication samples was rs955333 on chromosome 6 (minimum p-value 1.31x10^-8^ [additive]; minimum p-value 9.02x10^-11^ [dominant]) (**Table 2**). **Fig 1** contains the Manhattan plot for the meta-analysis across all ancestries included in the Discovery and Replication samples. Consistent directions of association were present in three ethnic groups (only AA samples did not pass QC) and several supporting single nucleotide polymorphisms (SNPs) were detected in the region (regional plot in **Fig 2**). This SNP lies between the SR-like carboxyl-terminal domain associated factor 8 gene (*SCAF8*) and the connector enhancer of KSR family of scaffold proteins gene (*CNKSR3*), suggesting a possible role in transcription regulation. *CNKSR3* is a direct mineralocorticoid receptor target gene involved in regulation of the epithelial sodium channel (ENaC) on the apical membrane of cells in the distal nephron.[12] *CNKSR3* is highly expressed in the renal cortical collecting duct and upregulated in response to physiologic aldosterone concentrations. ENaC precisely regulates renal sodium absorption and plays important roles in maintenance of plasma volume and blood pressure. Ziera *et al*. [12] suggested that CNKSR3, a PSD-95/DLG-1/ZO-1 (PDZ) domain containing protein, inhibits the RAS/ERK signaling pathway, stimulating ENaC activity with enhanced renal sodium absorption. More recently, CNKSR3 was shown to function as an aldosterone-induced scaffolding platform that orchestrated assembly of ENaC and its regulators Nedd4-2, Raf-1 and SGK-1 and was essential for stimulation of ENaC function by aldosterone.[13] Clinically, renin-angiotensin-aldosterone system (RAAS) blockade serves as a mainstay of therapy for patients with DKD and other proteinuric kidney diseases.[14,15] Inhibition of aldosterone may further limit renal fibrosis, independent of natriuretic effects.[16,17] Hence, significant association between DKD and markers near CNKSR3 is consistent with clinical trial data demonstrating that blockade of the renin angiotensin system or the aldosterone receptor slows DKD progression. However, further experiments are needed to demonstrate that the associated SNP regulates the pathogenesis of progressive DKD. Further studies will be necessary to assess if the *CNKSR3* regulates DKD pathogenesis indirectly by its effects on ENaC activity or directly by promoting aldosterone-dependent fibrosis.

**Table 2:** Trans-ethnic meta-analysis GWAS results, across Discovery and Replication samples.

					Discovery				Replication				Meta-Analysis			
SNP	Cytoband	Position	Gene	RA	OR	95% CI	P-value	Direction	OR	95% CI	P-value	Direction	OR	95% CI	P-value	*
rs955333	6q25.2	154947408		G	0.75	0.64–0.87	3.30E-04	?—-	0.72	0.63–0.83	1.04E-05	?—	0.73	0.66–0.81	1.31E-08	a
rs5750250	22q12.3	36708483	MYH9	G	1.39	1.23–1.57	2.56E-07	+???	1.15	1.03–1.29	1.76E-02	+??	1.27	1.16–1.38	7.70E-08	a
rs11107616	12q21.2	78364780	NAV3	C	1.42	1.20–1.69	2.55E-05	?+++	1.31	1.05–1.63	1.53E-02	?+?	1.38	1.21–1.57	1.24E-06	a
rs136161	22q12.3	36657432	APOL1	G	1.52	1.26–1.85	7.30E-07	+?++	1.09	0.88–1.34	3.97E-02	+-+	1.27	1.10–1.46	1.91E-06	r
rs12285658	11q21	94723623	KDM4D	G	0.55	0.33–0.93	1.75E-04	-??-	0.73	0.59–0.90	3.52E-03	-??	0.62	0.46–0.85	2.00E-06	d
rs4879670	9p13.3	33205408		G	1.20	1.10–1.31	2.37E-05	++-+	1.11	1.03–1.20	1.01E-02	+++	1.15	1.09–1.22	2.09E-06	a
rs11582609	1p31.1	83220656		A	0.76	0.66–0.87	4.55E-05	-+—	0.85	0.75–0.97	1.02E-02	—-	0.81	0.73–0.89	3.46E-06	d
rs1997066	10q25.1	106763563	SORCS3	C	0.59	0.46–0.76	2.30E-05	-?—	0.77	0.62–0.96	1.89E-02	-?-	0.68	0.57–0.80	4.28E-06	d
rs13259109	8q23.3	113115897		G	1.15	1.03–1.28	7.37E-03	++++	1.22	1.11–1.34	1.72E-04	+++	1.19	1.11–1.27	4.58E-06	a
rs6432852	2q24.3	166754553	TTC21B	G	0.84	0.77–0.92	3.63E-04	——	0.87	0.80–0.94	3.51E-03	—-	0.85	0.80–0.91	5.18E-06	a

Direction: RA is risk allele. The odds ratio (OR) is presented for the risk allele, compared with the non-risk allele, for a given model. The FIND ancestry groups are presented in the following order: AA-AI-EA-MA. A “+” or “-”indicates the direction of the effect in individuals of a specific ancestry. A “?” denotes that the indicated SNP did not pass QC in that ancestry and the results were not included in the meta-analysis. *a, additive; r, recessive; d, dominant

Less is known about the function of *SCAF8*, also known as *RBM16*. *SCAF8* is a RNA maturation factor recruited to the carboxy-terminal domain of RNA polymerase II in a phosphorylation-dependent manner.[18] It also is a target for ataxia telangiectasia mutated (ATM) kinase, a crucial component of the DNA damage response required for DNA repair and cell cycle control.[19] ATM kinase is associated with responsiveness of patients with DM to the insulin sensitizer metformin in some but not all studies.[20,21] Thus, genes in the region of rs955333 are suggestive of DKD-related pathogenesis.

GWAS loci identify elements that may regulate gene expression, and recent data indicate GWAS associations are located in regions bounded by recombination hot spots near non-coding causal variants, which regulate transcription.[22,23] We next contrasted transcript abundance of the genes within the megabase region centered on rs955333, *TIAM2*, *SCAF8*, *CNKSR3*, *IPCEF1* and *OPRM1*, in DKD and living donor kidney biopsies. DKD biopsies were obtained from European and AI cohorts and were analyzed separately. All five genes show statistically significant differential expression in at least one kidney tissue compartment of one population. *SCAF8* steady state mRNA levels show increased expression in DKD compared to living donor biopsies in glomerular and tubulo-interstitial compartments of both populations (**S2B Table**); *TIAM2* and *OPRM1* show glomerular-specific differential expression; *IPCEF1* is repressed in both tissue compartments of AI subjects; and *CNKSR3* is increased in the tubulo-interstitial compartment of AIs (**S2B Table**). Normalized tubulo-interstitial expression of *CNKSR3* correlated with urine albumin (r = 0.78, q = 0.0056) and urine albumin:creatinine ratio (UACR) (r = 0.74, q = 0.0107). In addition, *IPCEF1*, located downstream of *CNKSR3*, has been reported to be translated with *CNKSR3* as one protein,[24] and has a tubulo-interstitial expression quantitative trait locus (eQTL) (NM_001130699, rs249964, P = 2.34E-04) (**S2A Table**). LD between this SNP and the sentinel variants in the region significantly associated with DKD in the trans-ethnic (rs955333) and AI association analysis (rs12523822; see below) is negligible (D’ = 0.43, r^2^ = 0.01 in AI). However, tubulo-interstitial expression of *IPCEF1* in kidney tissue from AIs was significantly correlated with the DKD phenotype UACR (r = -0.54, q = 0.031). These studies were limited by the small number of available biopsies the narrow criteria used to define the region of interest (see Methods). As proxies, disease-dependent differential gene expression and the rs249964 eQTL demonstrate DKD regulatory activity in the locus. Significant results of eQTL and differential gene expression analyses for other loci in **Table 2** are also sown in **S2A Table** and **S2B Table**, respectively.

### African American Associations

No SNP reached genome-wide significance (P<5x10^-8^) in the AA GWAS; however, a number provided suggestive evidence for association with DKD (**Table 3**; **S5A Table** and **S6A Table** summarize the top 200 SNP associations in the discovery GWAS and replication study, respectively). The strongest associations were found within the apolipoprotein L1 (*APOL1*) and non-muscle heavy chain 9 gene (*MYH9*) region on 22q (**Table 2**, Discovery + FILR meta-analysis: rs5750250, P = 7.7x10^-8^; rs136161, P = 5.23x10^-7^). Since G1 and G2 variants of *APOL1* are strongly associated with non-diabetic nephropathy in AA patients,[25–27] the G1/G2 compound risk was modeled under a recessive genetic model and these variants accounted for the associations on 22q in **Table 2** (rs5750250 P = 7.70x10^-8^, OR = 1.27; rs136161 P = 5.23x10^-7^, OR = 1.36). Association with G1/G2 within *APOL1* likely exists due to inclusion of non-FIND AA cases with coincident DM and unrecognized non-diabetic kidney disease.[28] *APOL1* was not associated with T2D-ESKD in a logistic regression analysis adjusting for age, gender and global ancestry restricted to FIND MALD and CHOICE (Choices for Healthy Outcomes In Caring for End-stage renal disease) study cases meeting the original FIND DKD case definition (rs73885319 P = 0.1098; rs71785313 P = 0.1182).[29]

**Table 3:** Top GWAS associations, by ethnicity.

Ethnicity					Discovery					Replication					Trans-Sample Meta-Analysis			Trans-ethnic Meta-Analysis		
**African American**																				
**SNP**	**Cytoband**	**Position**	**Gene**	**RA**	**Case RAF**	**Control RAF**	**OR**	**95% CI**	**P-value**	**Case RAF**	**Control RAF**	**OR**	**95% CI**	**P-value**	**OR**	**95% CI**	**P-value** ^**1**^	**OR**	**95% CI**	**P-value** ^**1**^
rs5750250	22q12.3	36708483	MYH9	G	0.58	0.51	1.39	1.23–1.57	2.56E-07	0.58	0.52	1.15	1.02–1.29	1.76E-02	1.27	1.16–1.38	7.70E-08	1.27	1.16–1.38	7.70E-08
rs136161	22q12.3	36657432	APOL1	G	0.78	0.75	1.45	1.21–1.73	4.17E-05	0.78	0.74	1.28	1.09–1.51	2.98E-03	1.36	1.21–1.54	5.23E-07^r^	1.27	1.10–1.46	1.91E-06^r^
rs1298908	10q23.1	82013134		G	0.22	0.21	1.41	1.18–1.69	2.13E-04	0.23	0.22	1.31	1.11–1.54	1.20E-03	1.36	1.21–1.54	8.83E-07^d^	1.07	1.01–1.14	3.98E-03
rs304029	3p26.1	4545824	ITPR1	G	0.31	0.27	1.30	1.14–1.49	1.47E-04	0.31	0.28	1.21	1.07–1.37	2.08E-03	1.26	1.15–1.38	1.10E-06	1.08	1.02–1.15	1.50E-03
rs590884	6q26	161378192		T	0.30	0.33	0.80	0.70–0.91	6.12E-04	0.28	0.34	0.81	0.71–0.91	7.31E-04	0.80	0.73–0.88	1.51E-06	0.91	0.85–0.97	2.13E-04
rs8067287	17p11.2	16816656		T	0.21	0.23	0.45	0.30–0.68	1.40E-04	0.21	0.22	0.52	0.33–0.81	4.41E-03	0.48	0.36–0.65	2.33E-06^r^	0.71	0.57–0.88	3.84E-04^r^
rs6943931	7p15.3	22414646		C	0.24	0.28	0.67	0.56–0.80	6.84E-06	0.25	0.28	0.84	0.72–0.99	3.56E-02	0.75	0.67–0.84	2.46E-06^d^	0.86	0.79–0.94	5.29E-05^d^
rs9510795	13q12.12	24216012	TNFRSF19	A	0.66	0.61	1.33	1.17–1.51	1.10E-05	0.65	0.61	1.13	1.01–1.28	3.59E-02	1.23	1.13–1.34	3.58E-06	1.02	0.96–1.09	8.09E-02
rs12285658	11q21	94723623	KDM4D	G	0.09	0.11	0.67	0.54–0.83	3.28E-04	0.09	0.10	0.73	0.59–0.90	3.52E-03	0.70	0.60–0.81	3.93E-06^d^	0.62	0.46–0.85	2.00E-06^d^
rs10766496	11p15.1	18738718	IGSF22	T	0.20	0.22	0.70	0.59–0.84	9.52E-05	0.18	0.21	0.81	0.68–0.96	1.27E-02	0.75	0.66–0.85	5.44E-06^d^	0.88	0.81–0.95	3.78E-04^d^
**American Indian**																				
**SNP**	**Cytoband**	**Position**	**Gene**	**RA**	**Case RAF**	**Control RAF**	**OR**	**95% CI**	**P-value**	**Case RAF**	**Control RAF**	**OR**	**95% CI**	**P-value**	**OR**	**95% CI**	**P-value** ^**1**^	**OR**	**95% CI**	**P-value** ^**1**^
rs12523822	6q25.2	154954420		T	0.20	0.25	0.58	0.43–0.80	7.97E-04	0.18	0.26	0.56	0.44–0.71	1.76E-06	0.57	0.47–0.69	5.74E-09^d^	0.83	0.76–0.90	1.87E-04^d^
rs13254600	8q24.13	124089526	WDR67	T	0.20	0.27	0.55	0.40–0.75	1.74E-04	0.16	0.23	0.61	0.48–0.78	7.81E-05	0.58	0.48–0.71	5.54E-08^d^	0.90	0.85–0.96	3.08E-02
rs10952362	7q36.1	152262798		C	0.59	0.51	1.47	1.18–1.84	5.30E-04	0.58	0.50	1.38	1.17–1.63	1.20E-04	1.42	1.24–1.63	2.34E-07	1.21	1.10–1.32	6.32E-04^d^
rs10019835	4q32.1	156633186	GUCY1A3	T	0.33	0.40	0.70	0.56–0.88	2.51E-03	0.29	0.37	0.70	0.59–0.83	6.38E-05	0.70	0.61–0.80	5.47E-07	0.89	0.84–0.95	3.16E-03
rs4667466	2q24.2	163689147	KCNH7	T	0.42	0.34	1.42	1.14–1.77	2.05E-03	0.48	0.39	1.36	1.16–1.60	1.87E-04	1.39	1.21–1.58	1.28E-06	1.05	0.99–1.11	3.86E-01
rs4453858	3p14.1	67457498	SUCLG2	T	0.62	0.69	0.67	0.53–0.85	8.51E-04	0.51	0.59	0.74	0.63–0.88	5.61E-04	0.71	0.62–0.82	1.74E-06	0.78	0.63–0.97	9.01E-03^r^
rs10778560	12q23.3	107801401	BTBD11	C	0.07	0.04	2.95	1.69–5.14	1.37E-04	0.11	0.07	1.61	1.18–2.20	2.87E-03	2.11	1.56–2.86	2.38E-06^d^	1.18	1.03–1.34	9.60E-02^d^
rs13421350	2q31.1	173318571	ITGA6	A	0.15	0.18	0.69	0.52–0.93	1.45E-02	0.10	0.15	0.59	0.45–0.76	4.90E-05	0.63	0.52–0.77	2.71E-06	0.85	0.79–0.93	1.85E-03
rs12497655	3q13.31	116764010		G	0.26	0.33	0.66	0.52–0.84	5.89E-04	0.21	0.27	0.73	0.61–0.89	1.31E-03	0.70	0.60–0.81	3.14E-06	0.87	0.80–0.95	5.48E-04
rs2077212	8p23.2	5982272		G	0.48	0.40	1.41	1.13–1.76	2.33E-03	0.56	0.48	1.33	1.13–1.55	4.77E-04	1.36	1.20–1.55	3.63E-06	1.08	1.02–1.14	5.16E-02
**European Ancestry**																				
**SNP**	**Cytoband**	**Position**	**Gene**	**RA**	**Case RAF**	**Control RAF**	**OR**	**95% CI**	**P-value**	**Case RAF**	**Control RAF**	**OR**	**95% CI**	**P-value**	**OR**	**95% CI**	**P-value** ^**1**^	**OR**	**95% CI**	**P-value** ^**1**^
rs7636648	3q21.1	123624772		A	0.57	0.64	0.71	0.57–0.89	3.17E-03	0.58	0.62	0.81	0.72–0.92	1.61E-03	0.78	0.70–0.87	3.94E-05	1.01	0.94–1.08	8.79E-01
rs9294977	6q27	169624172	THBS2	G	0.29	0.37	0.65	0.48–0.89	7.86E-03	0.30	0.33	0.75	0.63–0.90	2.29E-03	0.72	0.62–0.84	9.98E-05^d^	0.85	0.78–0.92	2.48E-04^d^
rs12469173	2p16.1	59802586		G	0.25	0.33	0.64	0.47–0.87	4.86E-03	0.26	0.29	0.77	0.64–0.92	4.52E-03	0.72	0.62–0.85	1.64E-04^d^	0.99	0.93–1.05	7.68E-01
rs10129960	14q21.2	43929782		G	0.52	0.45	1.25	1.01–1.55	4.40E-02	0.52	0.46	1.23	1.08–1.40	1.34E-03	1.24	1.11–1.38	1.71E-04	1.10	1.03–1.17	1.69E-03
rs9766355	6q27	169584640		G	0.50	0.59	0.52	0.36–0.75	5.93E-04	0.54	0.56	0.76	0.61–0.94	1.27E-02	0.67	0.56–0.81	1.99E-04^d^	0.93	0.86–1.00	7.46E-02
rs10109898	8q24.23	136819328		T	0.16	0.24	0.59	0.45–0.79	3.27E-04	0.18	0.21	0.79	0.60–1.04	8.88E-02	0.68	0.56–0.84	2.04E-04	0.94	0.81–1.08	2.11E-01^d^
rs215700	7p14.3	32399166		A	0.22	0.29	0.59	0.43–0.80	8.65E-04	0.26	0.28	0.79	0.66–0.95	1.15E-02	0.72	0.61–0.84	2.10E-04^d^	0.91	0.82–1.01	4.81E-02^d^
rs1198061	10q21.1	60773378		A	0.57	0.53	1.24	0.89–1.73	2.11E-01	0.61	0.56	1.39	1.16–1.68	5.33E-04	1.34	1.14–1.58	2.46E-04^r^	1.09	1.02–1.15	1.35E-03
rs11198399	10q26.11	120177638		C	0.21	0.20	1.09	0.80–1.51	5.79E-01	0.25	0.21	1.42	1.18–1.70	1.70E-04	1.30	1.11–1.53	2.76E-04^d^	1.14	1.03–1.25	2.48E-03^d^
rs1563916	8p21.3	21085331		C	0.39	0.35	1.18	0.94–1.49	1.56E-01	0.42	0.38	1.25	1.09–1.42	1.03E-03	1.22	1.09–1.38	3.50E-04	1.15	1.06–1.24	2.57E-04
**Mexican Ancestry**																				
**SNP**	**Cytoband**	**Position**	**Gene**	**RA**	**Case RAF**	**Control RAF**	**OR**	**95% CI**	**P-value**	**Case RAF**	**Control RAF**	**OR**	**95% CI**	**P-value**	**OR**	**95% CI**	**P-value** ^**1**^	**OR**	**95% CI**	**P-value** ^**1**^
rs7975752	12q24.21	116154654		G	0.31	0.25	1.76	1.39–2.21	1.67E-06	.	.	.	.	.	1.76	1.39–2.21	1.67E-06^d^	1.06	0.99–1.14	9.50E-02
rs731565	7q35	147406262	CNTNAP2	T	0.28	0.20	1.58	1.30–1.92	4.06E-06	.	.	.	.	.	1.58	1.30–1.92	4.06E-06	1.22	1.00–1.49	5.82E-02^r^
rs728571	16q12.2	55214093		A	0.32	0.39	0.67	0.56–0.80	5.70E-06	.	.	.	.	.	0.67	0.56–0.80	5.70E-06	0.87	0.80–0.96	1.01E-03
rs4849965	2p25.2	4608927		C	0.36	0.28	1.50	1.26–1.79	6.18E-06	.	.	.	.	.	1.50	1.26–1.79	6.18E-06	1.07	1.00–1.15	7.68E-02
rs10004231	4p13	42857573		G	0.17	0.22	0.17	0.08–0.37	7.81E-06	.	.	.	.	.	0.17	0.08–0.37	7.81E-06^r^	0.58	0.45–0.76	5.74E-06^r^
rs6910061	6p24.2	11101918	SMIM13	A	0.17	0.11	1.74	1.36–2.22	8.55E-06	.	.	.	.	.	1.74	1.36–2.22	8.55E-06	1.04	0.96–1.12	6.34E-01
rs1353202	6q22.31	125724359		C	0.10	0.15	0.54	0.42–0.71	1.03E-05	.	.	.	.	.	0.54	0.42–0.71	1.03E-05^d^	0.91	0.84–0.98	2.67E-02
rs17210536	8q24.21	131217790	ASAP1	C	0.10	0.06	2.10	1.51–2.92	1.08E-05	.	.	.	.	.	2.10	1.51–2.92	1.08E-05^d^	1.15	1.03–1.28	1.13E-02
rs6994403	8q24.13	125754231		C	0.38	0.45	0.59	0.46–0.75	1.33E-05	.	.	.	.	.	0.59	0.46–0.75	1.33E-05^d^	0.97	0.91–1.03	2.20E-01
rs13152588	4p14	37365976	KIAA1239	G	0.57	0.65	0.59	0.47–0.75	1.41E-05	.	.	.	.	.	0.59	0.47–0.75	1.41E-05^r^	0.95	0.89–1.01	1.08E-01

^1^ P-values shown are additive unless another model is denoted next to the p-value (d = dominant model, r = recessive model). RA is risk allele. The odds ratio (OR) is presented for the risk allele, compared with the non-risk allele, for a given model. Direction (discovery) is read in the order: AA-AI-EA-MA; Direction (replication) is read in the order: AA-AI-EA; a “?” denotes that ethnicity’s data did not pass QC and was not included in the meta-analysis. A “+” or “-”indicates the direction of the effect in individuals of a specific ancestry.

Regions beyond *22q* provided suggestive evidence of association in the AA Discovery + FILR meta-analysis including rs1298908 on 10q22 (OR = 1.36, P = 8.83x10^-7^) between *MAT1A* and *ANXA11*, in a region dense with regulatory elements and transcription factors. There was also an association on 3p26 (rs304029, OR = 1.26 P = 1.10x10^-6^) within inositol 1,4,5-trisphosphate receptor, type 1 (*ITPR1*), a gene involved in cerebellar and autoimmune disorders but not renal involvement.[30] The genes in these other candidate regions (*ANXA11*, *MAT1A* and *ITPR1*) also show statistically significant differential expression in at least one population and compartment; as do *IGSF22* near candidate rs11766496 on chromosome 11, and *TNFRSF19* near rs95107795 on chromosome 13. Other top AA associated regions in **Table 2** do not have clear connections to kidney disease. Since *APOL1* association likely reflected inclusion of non-FIND cases with non-diabetic nephropathy, a GWAS was re-computed within AAs in the discovery sample, which only included subjects lacking two *APOL1* risk variants. The top 200 associations from this GWAS are summarized in **S7 Table**. The correlation between the–log10 (p-value) for GWAS with and with AA subjects with and without two *APOL1* risk variants is r = 0.82 (**S4 Fig**). The top association in this subset GWAS was rs2780902 on 1p31 (OR = 0.52, P = 2.98x10^-7^) within Janus kinase 1 (*JAK1*), a member of the protein-tyrosine kinases.[31] The ENCODE data shows that this SNP resides within a region with numerous transcription factors and DNase I hypersensitivity sites. *JAK1* is a widely expressed membrane associated phosphoprotein and is involved in interferon transduction pathway. This kinase links cytokine ligand binding to tyrosine phosphorylation of various known signaling proteins and the signal transducers and activators of transcription (STATs). Another interesting association among the top 10 associations is rs2596230 on 15q14 (OR = 1.56, P = 9.36x10^-6^) within ryanodine receptor 3 (*RYR3*).[32] The protein encoded by *RYR3* functions to release calcium from intercellular storage in many cellular processes and the gene is expressed in the kidney. The closely related gene, *RYR2*, is associated with albuminuria.[33] Our prior analyses of transcript expression in DKD biopsies provide additional support for the associations. Both *JAK1* and *RYR3* (and *RYR2*) show differential expression that is restricted to the European subjects with Stage III and Stage IV CKD. *JAK1* expression is increased in DKD in both compartments, while *RYR3* and *RYR2* are depressed in the glomerulus.[34] We also recomputed the genome wide discovery and trans-ethnic meta-analysis removing AA subjects with *APOL1*. The top 200 associations are summarized in **S8 Table**.

### American Indian Associations

Several regions provided evidence of association with DKD in AIs (**Table 3**; **S5B Table** and **S6B Table** summarize the top 200 SNP associations in the discovery GWAS and replication study, respectively). The strongest association was with rs12523822 on 6q.25 in the *SCAF8-CNKSR3* gene region (OR = 0.57, P = 5.74x10^-9^). This SNP is in strong LD with rs955333, the top hit in the trans-ethnic meta-analysis (r^2^ = 0.96 in AI unrelated controls); **S5 Fig** graphically illustrates the extended linkage disequilibrium in this region in all but the AA samples. The A allele at rs955333 is the ancestral allele and confers susceptibility to DKD (as the G allele has OR<1 in **Table 2**); the A allele has a frequency of 0.76 in the American Indian samples and 0.85 in European American samples, **but** is nearly monomorphic in African American samples. The allele frequencies are very similar in population-based samples: 0.85 in HapMap CEU, 1.00 in YRI, 0.77 in MEX and 0.76 in full-heritage American Indians from the southwestern United States (R Hanson, personal communication). Thus, the high risk allele at this locus does not appear to be Amerindian specific. The p-value for association in European Americans is 0.0013 and 1.3x10^-6^ in American Indian, suggesting that the signal does not come entirely from American Indians samples. Further fine-mapping or sequencing will be necessary to fully characterize the association signal within and across ethnic groups. Another association that approached genome-wide significance was rs13254600 (OR = 0.58, P = 5.54x10^-8^) on 8q24 within WD repeat domain 67 (*WDR67*). This gene is expressed in a wide variety of tissues, including kidney, and may affect cellular membrane functions by regulating Rab GTPase activity.[35] TBC1D31 (WDR67) mRNA is increased in both compartments of kidney tissue from AIs, but only in the glomerulus for European subjects with more advanced DKD.

Another SNP of interest is rs10019835 (OR = 0.70, 5.47x10^-7^) on 4q32 within guanylate cyclase 1, soluble, alpha 3 (*GUCY1A3*); the protein encoded by *GUCY1A3* serves as a receptor for nitric oxide,[36] which through its role in endothelial function may be a mediator of DKD.[37] *GUCY1A3* is differentially expressed in both tissue compartments and both DKD biopsy cohorts, and shows one of the strongest differences of all genes in candidate regions (especially among the European subjects who have more advanced DKD) (**S3A Table** and **S3B Table; S6 Fig**). In addition, the candidate SNP rs10019835 has a tubulo-interstitial specific eQTL with the full-length isoform of *GUCY1A3* (NM_000856, P = 4.97x10^-4^). The shortest isoform of the gene (NM_001130687) has a glomerular eQTL with rs12504357 (P = 2.63x10^-5^), an intronic SNP that is 5kb upstream of the associated variant. These two eQTL SNPs have *D’* = 1 in some populations, likely reflecting low allele frequencies in the reference populations. Integrin alpha 6 (*ITGA6*, rs13421350, 2q31, OR = 0.58, P = 5.54x10^-8^) is involved in cell adhesion and is expressed in the kidney. The gene shows negative differential expression in Europeans with DKD, and it has both glomerular and tubulo-interstitial eQTL. The glomerular eQTL is with the SNP rs6758468 (P = 5.41x10^-4^), which is 143kb from the candidate; while the tubulo-interstitial eQTL is with rs12469788 (P = 3.26x10^-4^), which is 5kb from the candidate with *D’* = 1, but negligible *r* ^2^. Finally, rs10952362 on 7q36 near *XRCC2* (rs10952362, OR = 1.91, P = 7.99x10^-8^), a gene involved in DNA repair was strongly associated with DKD.[38] We find that *XRCC2* is repressed in the tubulo-interstitial kidney tissue from AIs.

### European American Associations

EA subjects comprised the smallest group within FIND and power to detect variants associated with DKD was limited (**S3 Fig**). None of the associations in the EA Discovery + FILR meta-analysis had a p-value <10^−5^ (**Table 3; S5C Table** and **S6C Table** summarize the top 200 SNP associations in the discovery GWAS and replication study, respectively).

### Mexican American Associations

Several suggestive associations were identified in the MA Discovery GWAS (**Table 3**; **S5D Table** summarizes the top 200 SNP associations in the GWAS). No replication cohort was available to be genotyped in FILR, so only the Discovery GWAS and trans-ethnic meta-analysis are reported (**Tables 2 and 3**). The strongest association was on 12q24 for rs7975752, located ~242 kb downstream of the mediator complex subunit 13-like (*MED13L*) gene (OR = 1.76, P = 1.67 x 10^−6^). MED13L functions as a transcriptional coactivator for RNA polymerase II-transcribed genes. While its functional significance in DKD is unclear, gene variants 4 Mb downstream (rs614226) and upstream (rs653178) on 12q24 show genome-wide significant association with ESKD [9] and CKD [39] in Europeans. We see that MED13L is repressed in both compartments in kidney tissue from AIs but only in the glomerular transcriptome in the European subjects. Association was observed between DKD and rs731565 (P = 4.06 x 10^−6^) residing within an intronic region of the contactin-associated protein-like 2 (*CNTNAP2*) gene on 7q36. SNP rs7805747, approximately 4 Mb downstream from rs731565 has been associated with CKD in European populations [39] Finally, rs4849965, 1.2 Mb upstream of the SRY-related HMG-box 11 (*SOX11*) gene on 2p25.2 trended toward association with DKD (OR 1.50, 95% CI 1.26–1.79; P = 6.18x10^-6^) and has previously been associated with CKD in Europeans.[39] We find that absolute tubulo-interstitial expression of SOX11 in AIs is correlated with ACR (r = 0.66, q = 0.029).

### Conclusions

The current FIND GWAS comprises the largest genetic analysis for severe DKD based upon risk for progression to ESKD in EA and high-risk non-European ethnic groups including AAs, AIs, and MAs. As in other GWAS, results support a role for multiple DKD susceptibility genes, each with weak effects. A number of the SNPs most strongly associated with DKD had additional support from compartment-specific gene expression measures and eQTL analysis obtained in European and American Indian populations. A novel chromosome 6q25.2 DKD locus was identified in AI samples; SNPs in this region had genome-wide significant association and consistent directions of effect in the meta-analysis across all ethnic groups. Independent support for this region comes from an association with serum creatinine/eGFR in a GWAS in East Asian populations (P = 2.6 x 10^−5^ at rs4870304) [40]. Strengths of the FIND GWAS were the severe phenotype in cases, focus on DKD in T2D, and inclusion of non-European populations. The 6q25.2 locus requires fine mapping and additional replication in independent sample sets of diabetic subjects with and without DKD that has sufficient power to detect associated, common variants with moderate effect size. Once localized and replicated, functional studies in animal and cell culture models will be necessary to discover the biological mechanisms responsible for the association of DKD with the underlying genetic architecture.

As in other GWAS for complex disease, many previously identified DKD loci were not replicated in the FIND analyses. The inconsistency between our data and published DKD GWAS could reflect that FIND limited the DKD case group to subjects with ESKD and DKD with heavy proteinuria felt to be at high risk for progression to ESKD. FIND did not include microalbuminuric participants as “cases” in the Discovery cohort, choosing instead to focus on advanced nephropathy. However, some microalbuminuric participants with ACR<100 mg/g were included in the replication analysis. Prior GWAS focused on European and Asian DKD populations, often enriched for T1D-associated DKD. Genetic associations may not replicate across other populations; for example, association of *APOL1* variants with non-diabetic kidney disease is limited to populations with recent African ancestry. Another possible interpretation is the variants, which regulate DKD pathogenesis, are distinct for T1D and T2D, although a meta-analysis including both T1D and T2D subjects may identify shared loci. Finally, the DKD phenotype in the FIND GWAS relied on standard, stringent clinical criteria for advanced DKD. This approach limited phenotypic heterogeneity but potentially minimized the utility of cross-study comparisons. Although heavy proteinuria is a hallmark of DKD, recent analyses suggest approximately one third of patients with diabetes and an eGFR <60 ml/min per 1.73 m^2^ had normal urinary protein excretion.[4] This would justify the focus of FIND on advanced DKD. Although not the only DKD phenotype with a genetic component, several investigators recently proposed using ESKD as the optimal DKD phenotype in genetic association studies.[41,42] The availability of bio-samples from patients with advanced DKD is limited. Therefore, entry criteria in the present replication cohorts were loosened to increase sample size; this likely included a small number of participants with non-diabetic CKD (or DKD less likely to progress to ESKD). The AA non-FIND cases used in our replication cohort appear to have included individuals with DM and coincident focal segmental glomerulosclerosis (FSGS), an effect addressed via partitioning based on *APOL1* G1 and G2.[28] As in all GWAS, some non-nephropathy controls may develop DKD. This effect would bias results toward the null making it less likely to detect significant association.

FIND was well-powered to detect common risk variants with moderate effect sizes shared across ethnic groups. It was also well powered to use differences in effect sizes to help localize the region of association via transracial mapping. However, it was not powered to detect modest ethnic-specific effects that are not shared with another ethnicity or gene-gene interactions. Thus, these ethnic-specific scans provide important hypothesis generating results for subsequent meta-analyses, pathway enrichment analyses and hypothesis generation.

## Materials and Methods

### Ethics Statement

The FIND was completed in accordance with the principles of the Declaration of Helsinki. Written informed consent was obtained from all participants. The Institutional Review Board at each participating center (Case Western Reserve University, Cleveland, OH, Harbor-University of California Los Angeles Medical Center, Johns Hopkins University, Baltimore, National Institute of Diabetes and Digestive and Kidney Diseases, Phoenix, AZ, University of California, Los Angeles, CA, University of New Mexico, Albuquerque, NM, University of Texas Health Science Center at San Antonio, San Antonio, TX, Wake Forest School of Medicine, Winston-Salem, NC) approved all procedures, and all study subjects provided written informed consent. A certificate of confidentiality was filed at the National Institutes of Health.

### Samples

#### Discovery cohorts

FIND is a multi-ancestry family study of severe DKD.[11] Index cases had advanced DKD, likely to progress to ESKD based on clinical criteria, and at least one informative sibling with either DKD or long-standing DM without nephropathy. Detailed phenotype criteria for enrollment have been reported.[26,43,44] Index cases of AA, EA, MA and AI ethnicity were included in the Discovery GWAS; all had DM duration >5 years and/or DR, with UACR >1 g/g or ESKD. Unrelated controls had DM duration ≥9 years, UACR <30 mg/g (equating to overnight albumin excretion <20 mcg/min), and serum creatinine <1.6 mg/dl ([122 μmol/L] men) or <1.4 mg/dl ([107 μmol/L] women). In AA, EA and MA only unrelated cases and controls were included; since AI participants were largely recruited from relatively small communities all available cases and controls meeting criteria were included, regardless of relationships.

Additional non-FIND-study DKD cases and controls (with and without DM) were genotyped to increase power (**S1 Table**). Non-FIND samples included unrelated DKD cases and controls of self-reported African American ethnicity recruited at Wake Forest,[45] Case Western Reserve [46] and Howard Universities;[47] unrelated cases and controls of EA ethnicity recruited at Wake Forest [48] and Case Western Reserve;[49,50] cases and controls of AI ethnicity recruited at NIDDK-Phoenix; [51] and cases and controls of MA ethnicity recruited in San Antonio and Los Angeles.[52]

#### Replication cohorts

The FIND Large Replication (FILR) Study was comprised of samples independent from the Discovery cohorts. AA and EA replication cohorts were unrelated individuals recruited at Wake Forest, Johns Hopkins, Case Western Reserve and Harbor UCLA Universities and out-of-study control data from the Genetic Association Information Network (GAIN) consortium;[53] AI replication cohorts consisted of pedigree data from NIDDK-Phoenix [51] and from the Dakota and Oklahoma centers of the Strong Heart Family Study.[54] Replication cases had DM duration >5 years and/or DR, UACR ≥0.3 g/g (equating to overnight albumin excretion >200 mcg/min) and/or proteinuria >500 mg/day or ESKD. DM controls had an eGFR >60 ml/min/1.73 m^2^, UACR <30 mg/g after 10 year DM duration or UACR <100 mg/g after 15 year DM duration. GAIN study subjects with and without DM were used as controls; no kidney function data were available for these individuals. GAIN samples were excluded for specific SNPs, if MAFs were inconsistent with those in FIND controls. Additional MA subjects were not available for inclusion in FILR.

#### Samples analyzed

Based on ancestry, the FIND discovery GWAS samples included: (i) AA: 1564 DKD cases (633 in FIND, 931 out of study), 369 controls with DM lacking nephropathy (277 in FIND, 92 out of study) and 1,288 non-diabetic non-nephropathy controls (all out of study); (ii) AI: 538 DKD cases, 319 controls with DM lacking nephropathy; (iii) EA: 342 DKD cases, 404 controls with DM lacking nephropathy; and (iv) MA: 779 DKD cases and 594 controls with DM lacking nephropathy. The FILR replication study included: (i) AA: 950 DKD cases, 50 controls with DM lacking nephropathy and 1,887 non-diabetic non-nephropathy controls; (ii) AI: 471 DKD cases, 340 controls with DM lacking nephropathy and 486 non-diabetic non-nephropathy controls and (iii) EA: 582 DKD cases, 205 controls with DM lacking nephropathy and 2,568 non-diabetic non-nephropathy controls. FILR samples were genotyped at loci including the top associated SNPs from the Discovery GWAS, eQTL associations, literature-based candidate gene polymorphisms and ancestry informative markers (AIMs). S1 Table delineates the sample sources in the Discovery GWAS and FILR, stratified by ancestry.

### Genotyping and Statistical Methods

See Supplementary Methods (**S1 Text**).

### SNP Selection for the Discovery and Replication Study

The DNA samples that comprise the Discovery cohorts, plus an additional 244 blind duplicates were genotyped on the Affymetrix Genome-Wide Human 6.0 SNP array (see S1 Text Supplemental Methods for details). The FILR replication samples were genotyped for 3,937 SNPs selected based on the strength of the statistical association from the Discovery GWAS. Additional SNPs were included based on the FIND eQTL association and candidate gene SNPs previously reported to be associated with DKD (see S1 Text Supplemental Methods for details). Specifically, within each ancestry group, the SNPs with the strongest statistical evidence of association were identified; a few additional SNPs from each region with supportive but weaker evidence of association were also identified (i.e., associations due to LD but r^2^<0.95 with the primary associated SNP). This redundancy was designed to limit the number of regions not represented in the replication study due to genotyping failure. In total, 3,019 SNPs (821 AA, 790 AI, 608 EA, and 800 MA) were genotyped for FILR based solely on statistical association with DKD within an ethnicity. The trans-ethnic meta-analysis of the discovery cohort identified another 436 SNPs nominally associated with DKD (p<0.0003). In addition, 482 SNPs (121 AA, 133 AI, 122 EA, 14 MA, meta-analysis 92) were chosen with the smallest L_2_-norm (i.e., Euclidean distance) of the–log_10_ (*p*-values) from GWAS and eQTL association analyses, provided that *p* <0.01 from GWAS. Here, the L_2_-norm was defined relative to the maximum of the–log_10_ (*p*-values) from the GWAS and eQTL and provides an ordering of the combined evidence for eQTL and association with DKD. SNP associations in FILR were considered “replicated” if both the association reached statistical significance and direction of the association was consistent with the Discovery analysis. Finally, 278 AIMs were genotyped to allow for adjustment of potential population substructure. Thus, FILR was designed as a replication study and not a large-scale trans-ethnic fine-mapping study. Subsequent studies will complete fine-mapping to localize associations.

## Supporting Information

S1 Text Supplementary Materials and Methods including a complete membership list of FIND. (DOCX)

S1 Table FIND sample counts. (DOCX)

S2 Table A. Kidney tissue-compartment eQTL in AI biopsy participants corresponding to genomic regions determined by trans-ethnic meta-analysis GWAS results (Table 2). B. Differential expression of genes occurring in genomic regions determined by trans-ethnic meta-analysis GWAS candidates (Table 2). (DOCX)

S3 Table A. Kidney tissue-compartment eQTL in AI biopsy participants corresponding to genomic regions determined by candidates from ethnicity specific GWAS (Table 3) B. Differential expression of genes occurring in genomic regions determined by candidates from ethnicity specific GWAS (Table 3). (DOCX)

S4 Table Counts of differentially expressed genes at q ≤ 0.05 for ERCB and AI biopsy cohorts against the Living Donor cohort. (DOCX)

S5 Table Top 200 associations from the Discovery GWAS–A. African American, B. American Indian, C. European Ancestry and D. Mexican American. (DOCX)

S6 Table Top 200 associations from the FIND Replication–A. African American, B. American Indian and C. European ancestry. (DOCX)

S7 Table Top 200 associations from the FIND African American GWAS, excluding subjects with 2 copies of APOL1 G1 and/or G2 risk variants. (DOCX)

S8 Table Top 200 associations from the FIND Discovery GWAS Meta-analysis, excluding African American subjects with 2 copies of APOL1 G1 and/or G2risk variants. (DOCX)

S1 Fig FIND: Principle components 1 versus 2. The principal component (PC) analysis identified PCs that genetically partitioned the Discovery sample into ancestry groups consistent with self-report.
(TIFF)

S2 Fig P-P plots of discovery GWAS. The logistic regression model, which included the PCs as covariates, reduced the inflation factor to nominal levels and combined with the P-P plot show no evidence of a systematic inflation.
(DOCX)

S3 Fig Power curves. To estimate power, unmatched case and control subjects from Discovery plus Replication [FILR] included: AA: 2514 cases, 3594 controls; AI: 1009 cases, 1145 controls; EA: 924 cases, 3177 controls; MA: 779 cases, 594 controls (no Replication samples); and Meta-analysis: 5226 cases, 8510 controls. The following assumptions were used for power analysis: Additive Model; α = 1 x 10^−6^; and DM Population prevalence k_p_ = 0.30.
(TIFF)

S4 Fig Correlation between-log_10_(p-value) in the discovery AA GWAS including and excluding subjects with *APOL1* risk genotypes. The correlation between the–log_10_(p-value) for GWAS with and with AA subjects with and without two *APOL1* risk variants is r = 0.82.
(TIFF)

S5 Fig Ethnic-specific linkage disequilibrium patterns on 6q25.2. The *SCAF8-CNKSR3* region shows extended linkage disequilibriums in all ethnicities but AA.
(TIFF)

S6 Fig Plot of GUCY1A3 expression levels (log2 transformed) against variant rs10019835 genotypes showing effect indicated by eQTL in glomerulus but not the tubulo-interstitium/cortex compartment in American Indian participants. The full-length isoform NM_000856 has a tissue-specific tubulo-interstitial eQTL with AI GWAS candidate rs10019835 (P = 4.97 x 10^−4^, glomerulus not significant at p > 0.00024), and short isoform NM_001130687 having a glomerular eQTL with intronic SNP rs12504357 (P = 2.63 x 10^−5^, tubulo-interstitium not significant at p>0.05). Both isoforms satisfy the test for expression in both tissues.
(TIFF)
