# Supplementary material for: Genome-Wide Association and Trans-ethnic Meta-Analysis for Advanced Diabetic Kidney Disease: Family Investigation of Nephropathy and Diabetes (FIND)
Source: PLoS Genet. 2015 Aug 25;11(8):e1005352. doi: 10.1371/journal.pgen.1005352 (PMC4549309; doi:10.1371/journal.pgen.1005352)
Supplement: S1 Text — (DOCX) [file pgen.1005352.s001.docx]

**Genome-wide association and trans-ethnic meta-analysis for advanced diabetic kidney disease: Family Investigation of Nephropathy and Diabetes (FIND)**

**Members of the Family Investigation of Nephropathy and Diabetes Research Group**

*Genetic Analysis and Data Coordinating Center, Case Western Reserve University, Cleveland, Ohio:* RC Elston**, SK Iyengar*, KAB Goddard**, JM Olson** (deceased), RP Igo, Jr., S Ialacci^#^, C Fondran, J Fondran, A Horvath, G Jun, K Kramp, SRE Quade, M Slaughter, E Zaletel.

*Participating Investigator Centers:*

*Case Western Reserve University, Cleveland, OH:* JR Sedor*, J Schelling**, A Sehgal**, A Pickens^#^, L Humbert^#^, L Getz-Fradley^#^.

*Harbor-University of California Los Angeles Medical Center:* S Adler*, HE Collins-Schramm**^§^, E Ipp**, H Li**^§^, M Pahl**^†^, MF Seldin**^§^, J LaPage^#^, B Walker^#^, C Garcia^#^, J Gonzalez^#^, L Ingram-Drake^#^.

*Johns Hopkins University, Baltimore, MD:* M. Klag*, R. Parekh*, L Kao** (deceased), L Mead**, T Whitehead^#^, J Chester^#^.

*National Institute of Diabetes and Digestive and Kidney Diseases (NIDDK), Phoenix, AZ:* WC Knowler*, RL Hanson**, RG Nelson**, A Malhotra**, L Jones^#^, R Juan^#^, R Lovelace^#^, C Luethe^#^, LM Phillips^#^, J Sewemaenewa^#^, I Sili^#^, B Waseta^#^.

*University of California, Los Angeles, CA:* MF Saad*, SB Nicholas*, X Guo**, J Rotter**, K Taylor**, M Budgett^#^, F Hariri^#^.

*University of New Mexico, Albuquerque, NM:* P Zager*, V Shah**, M Scavini^#^, A Bobelu^#^.

*University of Texas Health Science Center at San Antonio, San Antonio, TX:* H Abboud*, NH Arar (deceased)**, R Duggirala**, Farook Thameem**, BS Kasinath**, R Plaetke**, M Stern**, C Goyes^#^, V Sartorio^#^, T Abboud^#^, L Hernandez^#^.

*Wake-Forest University, Winston-Salem, NC:* BI Freedman* ^‡^, DW Bowden**, SC Satko**, SS Rich**, S Warren^#^, S Viverette^#^, G Brooks^#^, R Young^#^, M Spainhour^#^.

*Laboratory of Genomic Diversity, National Cancer Institute, Frederick MD:* C Winkler*, MW Smith**, M Thompson^#^, R Hanson^#^, B Kessing^#^.

*NIDDK Program Office:* JP Briggs, PL Kimmel, R Rasooly.

*External Advisory Committee:* D Warnock (chair), A.R Chakraborty, GM Dunston, SJ O’Brien (ad hoc), R Spielman (deceased).

*Principal Investigator; **Co-investigator^; #^Program Coordinator

^§^University of California, Davis, CA

^†^University of California, Irvine, CA

^‡^Study Chair

**Supplementary Methods**

**Genotyping**

*Discovery cohort*. The DNA samples that comprise the Discovery cohorts, plus an additional 244 blind duplicates were genotyped on the Affymetrix Genome-Wide Human 6.0 SNP array [55] using the Affymetrix Commercial Service (Santa Clara, CA) via a contract with Translational Genomics Research Institute (TGEN). Samples submitted contained 100 ng/μl in low Tris/EDTA buffer and were tested for quality using PicoGreen. Samples were plated according to ethnicity and included HapMap controls and blind duplicates on each plate. Genotypes were called using Birdseed version 2 algorithm [56] implemented in Affymetrix Genotyping Console software. Genotype clusters for associated SNPs considered for FILR were manually examined for quality of the genotype calling.

*Replication cohort.* A custom Infinium chip including 6,000 bead types was manufactured at Illumina and genotyped at the Genome and Transcriptome Sequencing Core at Case Western Reserve University following the Infinium HD Assay Ultra manual protocol. Genotypes were called using the Illumina GenomeStudio version 2011.1 (<http://www.illumina.com/informatics/sequencing-microarray-data-analysis/genomestudio.ilmn>). SNPs not designable to the custom Illumina assay were genotyped using the KASP Assay at LCG Genomics (formally KBiosciences; Teddington, Middlesex, UK) and called via the KASP SNP Genotyping System.

Genotyping of the rs73885319 and rs71785313 nephropathy variants in the apolipoprotein L1 gene (*APOL1*) was performed using the Taqman assay in 703 AA ESKD cases with diabetes from the FIND Mapping by Admixture Disequilibrium (MALD) study and Choices for Healthy Outcomes In Caring for ESRD (CHOICE) studies and 806 FIND MALD controls by Kao *et al*.[29]

**Statistical Methods**

*Sample quality control*. Each sample genotype utilized in the discovery GWAS had an Affymetrix contrast quality control (QC) score >0.4 and a call rate >0.95 (Affymetrix, Inc. [2006]. BRLMM; an improved genotype calling method for the GeneChip Human Mapping 500K Array Set. Technical Report April 14, 2006 <http://www.affymetrix.com/support/technical/whitepapers/brlmm_whitepaper.pdf>). In addition, each sample was tested for cryptic relatedness (duplicates and first-degree relatives) via proportion of alleles shared identical by descent using the program PLINK [57] and RELTEST (S.A.G.E. [2011]. Statistical Analysis for Genetic Epidemiology, version 6.1. http//Darwin.cwru.edu/sage/.) Autosomal heterozygosity was computed using PLINK and ethnic-specific upper and lower thresholds for inclusion were determined from the ethnic-specific distributions. Samples were excluded if their self-reported gender was inconsistent with the genetically determined sex. Most samples had genotype data from prior studies; these data were compared to genotype data from the GWAS for sample verification.

Similar QC requirements were used in replication study samples. Here, the samples were required to have call rates >0.95 and were tested for cryptic relatedness (duplicates and first-degree relatives) and autosomal heterozygosity as described above.

*Admixture and SNP statistical quality control.* To account for potential population substructure, two principal component (PC) analyses were conducted using SNPs that passed QC and were not in genomic regions with extended LD.[58] Markers in the following regions were excluded: chromosomes 5 (44-51.5 Mb), 6 (25-33.5 Mb), 8 (8-12 Mb), 11 (45-57 Mb), and 17 (40-43 Mb). The PC analysis was conducted on the combined ancestry samples but separately for the GWAS and the replication study. Individuals whose self-reported ancestry did not match the clustering pattern of the PC were removed from the association analyses. In both the discovery GWAS and replication study, the first two PCs were determined to account for a large proportion of the genetic variation in the trans-ethnic PC analysis and appropriately reduce the inflation factor in the ancestry-specific logistic regression models.

*Tests for association*. To test for an association between an individual SNP and DKD, a logistic regression model was computed separately for each ancestry as implemented in the software SNPLash (<https://www.phs.wfubmc.edu>). In the ancestry-specific GWAS analyses, the logistic regression models included the covariates age, recruitment center, gender and the first two PCs. Adjustment for hemoglobin A1c and diabetic retinopathy were not performed, because participants had advanced nephropathy and recent glycemic control is less relevant to this outcome (shortened red blood cell survival also limits the accuracy of hemoglobin A1c) and diabetic retinopathy was included in enrollment criteria. Replication study analyses included gender and two PCs, as age was not available and recruitment center was not necessary. For both the discovery and replication studies, the tests of association were computed for the three *a priori* genetic models (i.e., dominant, additive, recessive). The additive genetic model was the primary inference unless there was significant evidence of departure from an additive genetic model (*p* <0.05), where departure was tested using the non-linear contrast for the three genotype levels. To increase the robustness of the tests of association, the additive and recessive genetic models required at least 10 and 30 individuals homozygous for the minor allele, respectively, and the dominant model required at least 10 individuals heterozygous or homozygous for the minor allele. Primary inference was on SNPs with overall missingness <0.05, no evidence of differential missingness between cases and controls (P<0.05), no evidence of departures from Hardy-Weinberg equilibrium (HWE) expectations (P<0.0001). SNPs with evidence for association but with significant evidence of a departure from HWE were examined for consistency with flanking markers in LD.

The GWAS association analysis using the FIND Discovery cohorts contrasted individuals with T2D versus T2D-associated DKD. The Replication analyses only considered SNPs from the GWAS, as well as a small number of published DKD candidate gene SNPs and eQTLs. Further, the allele frequency in analyses including out-of-study controls needed to be similar to the allele frequencies of T2D subjects without DKD to be viewed as a replication, which was informative for DKD. Thus, SNPs reported in this paper are T2D-DKD susceptibility loci and not T2D loci.

*APOL1* contains two major non-diabetic nephropathy risk variants (G1 and G2) found almost exclusively in individuals of recent African descent.[27] Despite association with CKD in hyperglycemic patients, they do not appear to associate with frank DKD.[59] Because of potential confounding effects of *APOL1* and the possibility that diabetic participants with non-diabetic etiologies of CKD could not be entirely excluded using clinical criteria, the GWAS analysis was repeated in AAs lacking two *APOL1* risk variants.

*Meta-analysis.* A series of meta-analyses were conducted to combine the evidence of association across the study using the weighted inverse normal method, weighted by sample size, as implemented in METAL.[60] First, a meta-analysis was conducted within each ancestry to combine the evidence for association across the discovery GWAS and replication study. Second, a meta-analysis was conducted that combined the evidence for association across ancestries in the discovery GWAS. Third, a meta-analysis was conducted that combined evidence of association across ethnicities within the replication study. Finally, an overall study meta-analysis of the ancestry-specific meta-analyses was conducted. Assuming criteria for numbers of homozygotes were met (discussed above), meta-analysis p-values were calculated for additive, dominant and recessive models.

*Analysis of gene expression and eQTL association.* Gene expression measurements were obtained from micro-dissected glomerular and tubulo-interstitial/cortical (TI) compartments,[61] and used in analysis of differential expression, correlated expression, and eQTL association. Differential and correlated expression were computed between living donor biopsies (n=18) and each of American Indian (n=22) and European biopsies (n=7); while expression measurements used for the eQTL associations are from protocol kidney biopsies of American Indian participants in a longitudinal study of DKD, who were also genotyped on the Affymetrix Genome-Wide Human 6.0 SNP array.[62] Of the eQTL participants, 65 have glomerular and 54 have TI expression profiles. Sample mRNA was hybridized to Affymetrix HGU133A and HGU133 Plus 2 microarrays [61, 63] and the image files obtained were processed with the GenePattern analysis pipeline [http://www.genepattern.com]. Background adjustment, quantile normalization and probe-set summarization were performed using Robust Multichip Analysis,[64] and batch correction done with Combat.[65] Gene-level analysis was used for differential and correlated expression, while RefSeq transcripts-level analysis was used for eQTL. Brainarray custom CDF [66] were used to annotate probe-sets for both analyses, with gene-level annotations from the Human Entrez Gene custom CDF (v.10), and transcript-level annotations from the RefSeq Custom CDF v12.1.0. Post-annotation, genes or transcripts were removed if for all participants the corresponding probe-set expression level was less than the median plus two standard deviations of the 27 negative Affymetrix control probe-sets.[67]

Differential gene expression was assessed using *Significance Analysis of Microarray* (SAM) as implemented in the MeV suite.[68] Correlation analyses were performed using GraphPad Prism version 6 statistical software (La Jolla California USA). In both cases, a *q*-value ≤ 0.05 was considered statistically significant.

For the eQTL analysis, the transcripts common to HGU133A and HGU133 Plus 2 annotations in the RefSeq Custom CDF were used in the analysis. Transcripts with no corresponding NCBI Entrez Gene entries were also removed, and a single probe-set was identified for each transcript by removing duplicates with identical expression. Genotyping QC was performed in the same way as for the GWAS SNPs, followed by filtering SNPs with minor allele frequency (MAF) <0.1 determined within the eQTL participants. After filtering, there were 13,179 transcripts and 464,043 SNPs for glomerular participants, and 13,061 transcripts and 460,921 SNPs for the TI participants.

An initial eQTL analysis used to prioritize GWAS candidates was performed using Merlin [69] with covariates of gender and renin-angiotensin system blocking medications, as well as the first four PCs of expression (excluding any PCs that correlate significantly [at p<0.05] with measured clinical observations of eGFR or UACR). The regressions were computed for all transcripts and SNPs for each tissue compartment, and restricted to associations in *cis* regions for each gene model. The *cis* region was defined as 150 kb upstream to 50 kb downstream of an extended gene model defined by finding the longest genomic region covered by the Consensus Coding Sequence Project (CCDS), Ensembl, RefSeq and UCSC gene models, as previously reported.[9] The *cis* eQTL significant at *p* <0.05 were identified in each compartment for the top 3000 GWAS candidates from each ethnicity. Reported eQTL associations were subsequently computed with Matrix-eQTL [70] using the covariates: gender, treatment and the first PCs of gene expression (3 for glomerulus, and 4 for TI), which were included to account for unknown environmental effects.

Supplementary Methods Reference List

9. Sandholm N, Salem RM, McKnight AJ, Brennan EP, Forsblom C, Isakova T, McKay GJ, Williams WW, Sadlier DM, Makinen VP, Swan EJ, Palmer C, Boright AP, Ahlqvist E, Deshmukh HA, Keller BJ, Huang H, Ahola AJ, Fagerholm E, Gordin D, Harjutsalo V, He B, Heikkila O, Hietala K, Kyto J, Lahermo P, Lehto M, Lithovius R, Osterholm AM, Parkkonen M, Pitkaniemi J, Rosengard-Barlund M, Saraheimo M, Sarti C, Soderlund J, Soro-Paavonen A, Syreeni A, Thorn LM, Tikkanen H, Tolonen N, Tryggvason K, Tuomilehto J, Waden J, Gill GV, Prior S, Guiducci C, Mirel DB, Taylor A, Hosseini SM, Parving HH, Rossing P, Tarnow L, Ladenvall C, henc-Gelas F, Lefebvre P, Rigalleau V, Roussel R, Tregouet DA, Maestroni A, Maestroni S, Falhammar H, Gu T, Mollsten A, Cimponeriu D, Ioana M, Mota M, Mota E, Serafinceanu C, Stavarachi M, Hanson RL, Nelson RG, Kretzler M, Colhoun HM, Panduru NM, Gu HF, Brismar K, Zerbini G, Hadjadj S, Marre M, Groop L, Lajer M, Bull SB, Waggott D, Paterson AD, Savage DA, Bain SC, Martin F, Hirschhorn JN, Godson C, Florez JC, Groop PH, Maxwell AP (2012) New susceptibility loci associated with kidney disease in type 1 diabetes. PLoS Genet 8: e1002921.

27. Genovese G, Friedman DJ, Ross MD, Lecordier L, Uzureau P, Freedman BI, Bowden DW, Langefeld CD, Oleksyk TK, Uscinski Knob AL, Bernhardy AJ, Hicks PJ, Nelson GW, Vanhollebeke B, Winkler CA, Kopp JB, Pays E, Pollak MR (2010) Association of trypanolytic ApoL1 variants with kidney disease in African Americans. Science 329: 841-845.

29. Kao WH (2012) Diabetic Nephropathy Fails to Associate with the APOL1/MYH9 Locus or Type 2 Diabetes Mellitus Susceptibility Genes: The Family Investigation of Nephropathy and Diabetes (FIND) Consortium . J Am Soc Nephol 23: 249A.

55. McCarroll SA, Kuruvilla FG, Korn JM, Cawley S, Nemesh J, Wysoker A, Shapero MH, de Bakker PI, Maller JB, Kirby A, Elliott AL, Parkin M, Hubbell E, Webster T, Mei R, Veitch J, Collins PJ, Handsaker R, Lincoln S, Nizzari M, Blume J, Jones KW, Rava R, Daly mj, Gabriel SB, Altshuler D (2008) Integrated detection and population-genetic analysis of SNPs and copy number variation. Nat Genet 40: 1166-1174.

56. Korn JM, Kuruvilla FG, McCarroll SA, Wysoker A, Nemesh J, Cawley S, Hubbell E, Veitch J, Collins PJ, Darvishi K, Lee C, Nizzari MM, Gabriel SB, Purcell S, Daly mj, Altshuler D (2008) Integrated genotype calling and association analysis of SNPs, common copy number polymorphisms and rare CNVs. Nat Genet 40: 1253-1260.

57. Purcell S, Neale B, Todd-Brown K, Thomas L, Ferreira MA, Bender D, Maller J, Sklar P, de Bakker PI, Daly mj, Sham PC (2007) PLINK: a tool set for whole-genome association and population-based linkage analyses. Am J Hum Genet 81: 559-575.

58. Price AL, Weale ME, Patterson N, Myers SR, Need AC, Shianna KV, Ge D, Rotter JI, Torres E, Taylor KD, Goldstein DB, Reich D (2008) Long-range LD can confound genome scans in admixed populations. Am J Hum Genet 83: 132-135.

59. Parsa A, Kao WH, Xie D, Astor BC, Li M, Hsu CY, Feldman HI, Parekh RS, Kusek JW, Greene TH, Fink JC, Anderson AH, Choi MJ, Wright JT, Jr., Lash JP, Freedman BI, Ojo A, Winkler CA, Raj DS, Kopp JB, He J, Jensvold NG, Tao K, Lipkowitz MS, Appel LJ (2013) APOL1 risk variants, race, and progression of chronic kidney disease. N Engl J Med 369: 2183-2196.

60. Willer CJ, Li Y, Abecasis GR (2010) METAL: fast and efficient meta-analysis of genomewide association scans. Bioinformatics 26: 2190-2191.

61. Cohen CD, Frach K, Schlondorff D, Kretzler M (2002) Quantitative gene expression analysis in renal biopsies: a novel protocol for a high-throughput multicenter application. Kidney Int 61: 133-140.

62. Weil EJ, Fufaa G, Jones LI, Lovato T, Lemley KV, Hanson RL, Knowler WC, Bennett PH, Yee B, Myers BD, Nelson RG (2013) Effect of losartan on prevention and progression of early diabetic nephropathy in American Indians with type 2 diabetes. Diabetes 62: 3224-3231.

63. Cohen CD, Klingenhoff A, Boucherot A, Nitsche A, Henger A, Brunner B, Schmid H, Merkle M, Saleem MA, Koller KP, Werner T, Grone HJ, Nelson PJ, Kretzler M (2006) Comparative promoter analysis allows de novo identification of specialized cell junction-associated proteins. Proc Natl Acad Sci U S A 103: 5682-5687. 0511257103 [pii];10.1073/pnas.0511257103 [doi].

64. Irizarry RA, Hobbs B, Collin F, Beazer-Barclay YD, Antonellis KJ, Scherf U, Speed TP (2003) Exploration, normalization, and summaries of high density oligonucleotide array probe level data. Biostatistics 4: 249-264.

65. Johnson WE, Li C, Rabinovic A (2007) Adjusting batch effects in microarray expression data using empirical Bayes methods. Biostatistics 8: 118-127.

66. Dai M, Wang P, Boyd AD, Kostov G, Athey B, Jones EG, Bunney WE, Myers RM, Speed TP, Akil H, Watson SJ, Meng F (2005) Evolving gene/transcript definitions significantly alter the interpretation of GeneChip data. Nucleic Acids Res 33: e175.

67. Berthier CC, Bethunaickan R, Gonzalez-Rivera T, Nair V, Ramanujam M, Zhang W, Bottinger EP, Segerer S, Lindenmeyer M, Cohen CD, Davidson A, Kretzler M (2012) Cross-species transcriptional network analysis defines shared inflammatory responses in murine and human lupus nephritis. J Immunol 189: 988-1001.

68. Saeed AI, Sharov V, White J, Li J, Liang W, Bhagabati N, Braisted J, Klapa M, Currier T, Thiagarajan M, Sturn A, Snuffin M, Rezantsev A, Popov D, Ryltsov A, Kostukovich E, Borisovsky I, Liu Z, Vinsavich A, Trush V, Quackenbush J (2003) TM4: a free, open-source system for microarray data management and analysis. Biotechniques 34: 374-378.

69. Abecasis GR, Cherny SS, Cookson WO, Cardon LR (2002) Merlin--rapid analysis of dense genetic maps using sparse gene flow trees. Nat Genet 30: 97-101.

70. Shabalin AA (2012) Matrix eQTL: ultra fast eQTL analysis via large matrix operations. Bioinformatics 28: 1353-1358.
