# Supplementary material for: Genome-Wide Association and Trans-ethnic Meta-Analysis for Advanced Diabetic Kidney Disease: Family Investigation of Nephropathy and Diabetes (FIND)
Source: PLoS Genet. 2015 Aug 25;11(8):e1005352. doi: 10.1371/journal.pgen.1005352 (PMC4549309; doi:10.1371/journal.pgen.1005352)
Supplement: S1 Table — (DOCX) [file pgen.1005352.s002.docx]

**Supplemental Table S1. FIND sample counts**

|  | **African American** | **American Indian** | **European American** | **Mexican American** |
| --- | --- | --- | --- | --- |
| **DISCOVERY** |  |  |  |  |
| **FIND Cases** | 633 | 538 | 342 | 779 |
| **FIND Diabetic Controls** | 277 | 319 | 404 | 594 |
| **Out-of-Study Cases** | 931^1,2^ | - | - | - |
| **Out-of-Study Non-Diabetic Controls** | 1288^1,2^ | - | - | - |
| **Out-of-Study Diabetic Controls** | 92^2^ | - | - | - |
|  |  |  |  |  |
| **REPLICATION** |  |  |  |  |
| **FIND Cases** | 950 | 471 | 582 | - |
| **FIND Diabetic Controls** | 50 | 340 | 205 | - |
| **FIND Non-Diabetic Controls** | 1 | 486 | 23 | - |
| **Out-of-Study Non-Diabetic Controls** | 1886^3^ | - | 2545^3^ | - |

^1^ Wake Forest T2DM AA GWAS

^2^ Howard University Family Study

^3^ GAIN via dbGAP
