# Supplementary material for: Genome-Wide Association and Trans-ethnic Meta-Analysis for Advanced Diabetic Kidney Disease: Family Investigation of Nephropathy and Diabetes (FIND)
Source: PLoS Genet. 2015 Aug 25;11(8):e1005352. doi: 10.1371/journal.pgen.1005352 (PMC4549309; doi:10.1371/journal.pgen.1005352)
Supplement: S2 Table — (DOCX) [file pgen.1005352.s003.docx]

**Supplemental Table S2a.** Kidney tissue-compartment eQTL in AI biopsy participants corresponding to genomic regions determined by trans-ethnic meta-analysis GWAS results (Table 2). Tests with p-values satisfying 0.00024 ≤ p for glomerulus and 0.00026 ≤ p for tubulo-interstitium are not significant.

|  |  |  |  |  |  | **eQTL p-value** | |
| --- | --- | --- | --- | --- | --- | --- | --- |
| **Chr** | **Candidate** | **GeneID** | **Symbol** | **RefSeq** | **eSNP** | **Glomerulus** | **Tubulo-interstitium** |
| 6 | rs955333 | 26034 | IPCEF1 | NM_001130699 | rs2499645 | p>0.05 | 2.34E-04 |
| 9 | rs4879670 | 2683 | B4GALT1 | NM_001497 | rs869 | 4.97E-04 | 2.09E-04 |
| 9 | rs4879670 | 2683 | B4GALT1 | NM_001497 | rs3780481 | 6.02E-04 | 2.38E-05 |
| 9 | rs4879670 | 55234 | SMU1 | NM_018225 | rs1013217 | p>0.05 | 1.27E-04 |
| 22 | rs136161 | 11020 | IFT27 | NM_006860 | rs9622461 | 1.30E-04 | 0.00026<p<=0.05 |
| 22 | rs136161 | 4627 | MYH9 | NM_002473 | rs2481 | p>0.05 | 4.10E-04 |

**Supplemental Table S2b.** Differential expression of genes occurring in genomic regions determined by trans-ethnic meta-analysis GWAS candidates (Table 2) and significant at q ≤ 0.05 in at least one kidney tissue compartment. Differential expression is between expression levels for Living Donors and both European ancestry (ERCB) and American Indian (AI) protocol biopsy participants as determined by SAM. Reported q-values are based on tests of all genes expressed in both Living Donors and the respective biopsy cohort (Supplemental Table 4). Tests that could not be completed are marked with an asterisk (*).

|  | | | | **Glomerulus** | | | | **Tubulo-Interstitium** | | | |
| --- | --- | --- | --- | --- | --- | --- | --- | --- | --- | --- | --- |
|  |  |  |  | **ERCB DKD** | | **AI DKD** | | **ERCB DKD** | | **AI DKD** | |
| **Chr** | **RSID** | **Symbol** | **GeneID** | **logFC** | **q-value** | **logFC** | **q-value** | **logFC** | **q-value** | **logFC** | **q-value** |
| 2 | rs6432852 | SCN1A | 6323 | – | * | – | * | 0.06 | 0.4 | -0.05 | 0.32 |
| 6 | rs955333 | CNKSR3 | 154043 | 0.18 | 0.08 | 0.12 | 0.07 | 0.18 | 0.12 | 0.22 | 0 |
| 6 | rs955333 | SCAF8 | 22828 | 0.28 | 0.01 | 0.32 | 0 | 0.29 | 0.02 | 0.21 | 0.01 |
| 10 | rs1997066 | SORCS3 | 22986 | – | * | – | * | 0.18 | 0.07 | 0.07 | 0.17 |
| 11 | rs12285658 | KDM4D | 55693 | -0.1 | 0.07 | -0.07 | 0.08 | -0.06 | 0.37 | 0.05 | 0.24 |
| 12 | rs11107616 | NAV3 | 89795 | 0.28 | 0 | 0.13 | 0 | 0.29 | 0.07 | 0.02 | 0.44 |
| 22 | rs136161 | APOL1 | 8542 | 0.22 | 0.05 | 0.74 | 0 | 0.43 | 0 | – | * |
| 22 | rs5750250 | MYH9 | 4627 | -0.11 | 0.21 | 0.17 | 0.01 | 0.1 | 0.43 | -0.35 | 0 |
