# Supplementary material for: Genome-Wide Association and Trans-ethnic Meta-Analysis for Advanced Diabetic Kidney Disease: Family Investigation of Nephropathy and Diabetes (FIND)
Source: PLoS Genet. 2015 Aug 25;11(8):e1005352. doi: 10.1371/journal.pgen.1005352 (PMC4549309; doi:10.1371/journal.pgen.1005352)
Supplement: S3 Table — (DOCX) [file pgen.1005352.s004.docx]

**Supplemental Table S3A.** Kidney tissue-compartment eQTL in AI biopsy participants corresponding to genomic regions determined by candidates from ethnicity specific GWAS (Table 3). Tests with p-values satisfying 0.00024 ≤ p for glomerulus and 0.00026 ≤ p for tubulo-interstitium are not significant.

| **African Ancestry** | | | | | | | |
| --- | --- | --- | --- | --- | --- | --- | --- |
|  | | | | | | **eQTL p-value** | |
| **Chr** | **Candidate** | **GeneID** | **Symbol** | **RefSeq** | **eSNP** | **Glomerulus** | **Tubulo-interstitium** |
| 6 | rs590884 | 5071 | PARK2 | NM_004562 | rs881829 | 4.35E-04 | p>0.05 |
| 7 | rs6943931 | 9771 | RAPGEF5 | NM_012294 | rs4722113 | 9.71E-05 | p>0.05 |
| 7 | rs6943931 | 54543 | TOMM7 | NM_019059 | rs17147339 | 0.00024<p<=0.05 | 3.39E-04 |
| 10 | rs1298908 | 6441 | SFTPD | NM_003019 | rs10887288 | 2.40E-06 | p>0.05 |
| 11 | rs10766496 | 3948 | LDHC | NM_002301 | rs2403279 | 2.91E-07 | 2.93E-11 |
| 17 | rs8067287 | 23164 | MPRIP | NM_015134 | rs4641803 | 6.13E-15 | 0.00026<p<=0.05 |
| 17 | rs8067287 | 23164 | MPRIP | NM_015134 | rs10852844 | 9.53E-14 | 6.12E-04 |
| 22 | rs136161 | 11020 | IFT27 | NM_006860 | rs9622461 | 1.30E-04 | 0.00026<p<=0.05 |
| 22 | rs136161 | 4627 | MYH9 | NM_002473 | rs2481 | p>0.05 | 4.10E-04 |
| 22 | rs5750250 | 11020 | IFT27 | NM_006860 | rs9622461 | 1.30E-04 | 0.00026<p<=0.05 |
| 22 | rs5750250 | 4627 | MYH9 | NM_002473 | rs2481 | p>0.05 | 4.10E-04 |
| **American Indian** | | | | | | | |
|  |  |  |  |  |  | **eQTL p-value** | |
| **Chr** | **Candidate** | **GeneID** | **Symbol** | **RefSeq** | **eSNP** | **Glomerulus** | **Tubulo-interstitium** |
| 2 | rs13421350 | 3655 | ITGA6 | NM_000210 | rs6758468 | 5.41E-04 | p>0.05 |
| 2 | rs13421350 | 3655 | ITGA6 | NM_000210 | rs12469788 | 0.00024<p<=0.05 | 3.27E-04 |
| 3 | rs4453858 | 8801 | SUCLG2 | NM_003848 | rs12497038 | 4.23E-05 | 0.00026<p<=0.05 |
| 4 | rs10019835 | 2982 | GUCY1A3 | NM_001130687 | rs12504357 | 2.63E-05 | p>0.05 |
| 4 | rs10019835 | 2982 | GUCY1A3 | NM_000856 | rs10019835 | 0.00024<p<=0.05 | 4.97E-04 |
| 6 | rs12523822 | 26034 | IPCEF1 | NM_001130699 | rs2499645 | p>0.05 | 2.34E-04 |
| 8 | rs13254600 | 55093 | WDYHV1 | NM_018024 | rs6415480 | 6.78E-07 | 3.09E-08 |
| 8 | rs2077212 | 285 | ANGPT2 | NM_001118887 | rs11989215 | 4.36E-04 | p>0.05 |
| **European Ancestry** | | | | | | | |
|  |  |  |  |  |  | **eQTL p-value** | |
| **Chr** | **Candidate** | **GeneID** | **Symbol** | **RefSeq** | **eSNP** | **Glomerulus** | **Tubulo-interstitium** |
| 3 | rs7636648 | 8997 | KALRN | NM_001024660 | rs12695438 | 9.72E-05 | 0.00026<p<=0.05 |
| 3 | rs7636648 | 8997 | KALRN | NM_001024660 | rs16834939 | p>0.05 | 4.04E-04 |
| 6 | rs9294977 | 387263 | C6orf120 | NM_001029863 | rs9371122 | 5.07E-04 | 0.00026<p<=0.05 |
| **Mexican Ancestry** | | | | | | | |
|  |  |  |  |  |  | **eQTL p-value** | |
| **Chr** | **Candidate** | **GeneID** | **Symbol** | **RefSeq** | **eSNP** | **Glomerulus** | **Tubulo-interstitium** |
| 4 | rs10004231 | 10396 | ATP8A1 | NM_001105529 | rs10008052 | 3.17E-06 | p>0.05 |
| 4 | rs10004231 | 10396 | ATP8A1 | NM_001105529 | rs1563849 | 0.00024<p<=0.05 | 5.60E-05 |

**Supplemental Table S3B.** Differential expression of genes occurring in genomic regions determined by candidates from ethnicity specific GWAS (Table 3) and significant at q ≤ 0.05 in at least one kidney tissue compartment for one biopsy cohort. Differential expression is between expression levels for Living Donors and both European ancestry (ERCB) and American Indian (AI) protocol biopsy participants as determined by SAM. Reported q-values are based on tests of all genes expressed in both Living Donors and the respective biopsy cohort (Supplemental Table 4). Tests that could not be completed are marked with an asterisk (*).

| **African Ancestry** | | | | | | | | | | | |
| --- | --- | --- | --- | --- | --- | --- | --- | --- | --- | --- | --- |
|  | | | | **Glomerulus** | | | | **Tubulo-Interstitium** | | | |
|  |  |  |  | **ERCB DKD** | | **AI DKD** | | **ERCB DKD** | | **AI DKD** | |
| **Chr** | **RSID** | **Symbol** | **GeneID** | **logFC** | **q-value** | **logFC** | **q-value** | **logFC** | **q-value** | **logFC** | **q-value** |
| 1 | rs2789002 | JAK1 | 3716 | 0.24 | 0.03 | 0.04 | 0.16 | 0.44 | 0 | 0.05 | 0.45 |
| 3 | rs304029 | ITPR1 | 3708 | -0.07 | 0.28 | -0.18 | 0.06 | -0.34 | 0.02 | -0.04 | 0.47 |
| 10 | rs1298908 | ANXA11 | 311 | 0.35 | 0 | 0.25 | 0 | 0.39 | 0.01 | 0.02 | 0.52 |
| 10 | rs1298908 | MAT1A | 4143 | -0.11 | 0.19 | -0.17 | 0.02 | – | * | -0.01 | 0.54 |
| 11 | rs10766496 | IGSF22 | 283284 | 0.07 | 0.29 | 0.09 | 0.04 | -0.06 | 0.4 | -0.17 | 0.03 |
| 11 | rs12285658 | KDM4D | 55693 | -0.1 | 0.07 | -0.07 | 0.08 | -0.06 | 0.37 | 0.05 | 0.24 |
| 13 | rs9510795 | TNFRSF19 | 55504 | 0.65 | 0 | 0.13 | 0.03 | 0.8 | 0 | 0.92 | 0 |
| 15 | rs2596230 | RYR3 | 6263 | -0.65 | 0.02 | -0.16 | 0.25 | -0.07 | 0.33 | -0.01 | 0.55 |
| 22 | rs136161 | APOL1 | 8542 | 0.22 | 0.05 | 0.74 | 0 | 0.43 | 0 | – | * |
| 22 | rs5750250 | MYH9 | 4627 | -0.11 | 0.21 | 0.17 | 0.01 | 0.1 | 0.43 | -0.35 | 0 |
| **American Indian** | | | | | | | | | | | |
|  | | | | **Glomerulus** | | | | **Tubulo-Interstitium** | | | |
|  |  |  |  | **ERCB DKD** | | **AI DKD** | | **ERCB DKD** | | **AI DKD** | |
| **Chr** | **RSID** | **Symbol** | **GeneID** | **logFC** | **q-value** | **logFC** | **q-value** | **logFC** | **q-value** | **logFC** | **q-value** |
| 2 | rs13421350 | ITGA6 | 3655 | -0.22 | 0.05 | -0.03 | 0.26 | -0.17 | 0.12 | 0.14 | 0.11 |
| 3 | rs4453858 | SUCLG2 | 8801 | -0.36 | 0 | -0.32 | 0 | -0.39 | 0.01 | -0.15 | 0.07 |
| 4 | rs10019835 | GUCY1A3 | 2982 | 1.7 | 0 | 0.63 | 0 | 1.27 | 0 | 0.49 | 0 |
| 6 | rs955333 | CNKSR3 | 154043 | 0.18 | 0.08 | 0.12 | 0.07 | 0.18 | 0.12 | 0.22 | 0 |
| 6 | rs955333 | SCAF8 | 22828 | 0.28 | 0.01 | 0.32 | 0 | 0.29 | 0.02 | 0.21 | 0.01 |
| 7 | rs10952362 | XRCC2 | 7516 | -0.2 | 0.13 | -0.15 | 0.11 | -0.04 | 0.47 | -0.27 | 0.02 |
| 8 | rs13254600 | TBC1D31 | 93594 | 0.21 | 0.02 | 0.22 | 0 | 0.09 | 0.4 | 0.19 | 0.03 |
| 12 | rs10778560 | BTBD11 | 121551 | -0.39 | 0.02 | -0.11 | 0.21 | -0.54 | 0 | -0.2 | 0.07 |
| **European Ancestry** | | | | | | | | | | | |
|  | | | | **Glomerulus** | | | | **Tubulo-Interstitium** | | | |
|  |  |  |  | **ERCB DKD** | | **AI DKD** | | **ERCB DKD** | | **AI DKD** | |
| **Chr** | **RSID** | **Symbol** | **GeneID** | **logFC** | **q-value** | **logFC** | **q-value** | **logFC** | **q-value** | **logFC** | **q-value** |
| 3 | rs7636648 | CCDC14 | 64770 | 0.25 | 0.01 | 0.14 | 0 | 0.19 | 0.07 | 0.11 | 0.07 |
| 3 | rs7636648 | MYLK | 4638 | 0.07 | 0.29 | 0.24 | 0 | 0.25 | 0.16 | -0.18 | 0.12 |
| 3 | rs9815887 | RBMS3 | 27303 | – | * | -0.08 | 0.22 | 0.52 | 0.03 | – | * |
| 6 | rs9294977 | THBS2 | 7058 | 3.52 | 0 | 0.52 | 0.17 | 2.1 | 0 | 0.3 | 0.4 |
| **Mexican Ancestry** | | | | | | | | | | | |
|  | | | | **Glomerulus** | | | | **Tubulo-Interstitium** | | | |
|  |  |  |  | **ERCB DKD** | | **AI DKD** | | **ERCB DKD** | | **AI DKD** | |
| **Chr** | **RSID** | **Symbol** | **GeneID** | **logFC** | **q-value** | **logFC** | **q-value** | **logFC** | **q-value** | **logFC** | **q-value** |
| 2 | rs4849965 | SOX11 | 6664 | -0.06 | 0.18 | -0.03 | 0.24 | 0.06 | 0.44 | -0.02 | 0.54 |
| 7 | rs731565 | CNTNAP2 | 26047 | – | * | – | * | 0.06 | 0.38 | -0.01 | 0.56 |
| 8 | rs17210536 | ASAP1 | 50807 | -0.13 | 0.13 | -0.19 | 0.02 | 0.26 | 0.05 | 0.05 | 0.51 |
| 8 | rs17210536 | FAM49B | 51571 | 0.54 | 0 | 0.16 | 0.01 | 0.3 | 0.01 | 0.03 | 0.54 |
| 8 | rs6994403 | MTSS1 | 9788 | -0.07 | 0.2 | 0.07 | 0.03 | -0.28 | 0 | 0 | 0.57 |
| 8 | rs6994403 | PVT1 | 5820 | -0.23 | 0.03 | -0.19 | 0.01 | -0.1 | 0.3 | -0.08 | 0.31 |
| 8 | rs6994403 | ZHX2 | 22882 | 0.35 | 0.01 | 0.15 | 0.02 | 0.36 | 0 | 0.09 | 0.15 |
| 12 | rs7975752 | MED13L | 23389 | -0.35 | 0 | -0.14 | 0.05 | -0.06 | 0.38 | -0.24 | 0 |
