# Supplementary material for: Genome-Wide Association and Trans-ethnic Meta-Analysis for Advanced Diabetic Kidney Disease: Family Investigation of Nephropathy and Diabetes (FIND)
Source: PLoS Genet. 2015 Aug 25;11(8):e1005352. doi: 10.1371/journal.pgen.1005352 (PMC4549309; doi:10.1371/journal.pgen.1005352)
Supplement: S4 Table — (DOCX) [file pgen.1005352.s005.docx]

**Supplemental Table S4.** Counts of differentially expressed genes at q ≤ 0.05 for ERCB and AI biopsy cohorts against the Living Donor cohort.

|  | **Glomerulus** | | **Tubulo-Interstitium** | |
| --- | --- | --- | --- | --- |
| **Biopsy cohort** | **q ≤ 0.05** | **Genes tested** | **q ≤ 0.05** | **Genes tested** |
| ERCB DKD | 8452 | 16767 | 2870 | 16946 |
| AI DKD | 9431 | 16809 | 4784 | 16946 |
