# Supplementary material for: Genome-Wide Association and Trans-ethnic Meta-Analysis for Advanced Diabetic Kidney Disease: Family Investigation of Nephropathy and Diabetes (FIND)
Source: PLoS Genet. 2015 Aug 25;11(8):e1005352. doi: 10.1371/journal.pgen.1005352 (PMC4549309; doi:10.1371/journal.pgen.1005352)
Supplement: S5 Table — (DOCX) [file pgen.1005352.s006.docx]

**Supplemental Table S5a.**

**Top 200 associations from the Discovery GWAS – African Ancestry**

| **SNP** | **Cytoband** | **Position** | **RA** | **Case  RAF** | **Control RAF** | **OR** | **95% CI** | **P-value** | |
| --- | --- | --- | --- | --- | --- | --- | --- | --- | --- |
| rs1249910 | 3q13.2 | 112391174 | T | 0.60 | 0.65 | 0.49 | 0.38-0.63 | 7.28E-08 | ^d^ |
| rs5750250 | 22q12.3 | 36708483 | G | 0.58 | 0.51 | 1.39 | 1.23-1.57 | 2.56E-07 |  |
| rs1945380 | 11q14.1 | 80418965 | C | 0.46 | 0.50 | 0.73 | 0.64-0.82 | 5.37E-07 |  |
| rs7972171 | 12q13.2 | 55444946 | C | 0.06 | 0.09 | 0.55 | 0.44-0.70 | 1.17E-06 |  |
| rs1259770 | 12p11.21 | 32076768 | C | 0.57 | 0.54 | 1.67 | 1.34-2.07 | 4.30E-06 | ^d^ |
| rs1259769 | 12p11.21 | 32076736 | T | 0.57 | 0.54 | 1.67 | 1.34-2.07 | 4.31E-06 | ^d^ |
| rs9510795 | 13q12.12 | 24216012 | A | 0.66 | 0.61 | 1.51 | 1.27-1.81 | 4.59E-06 | ^r^ |
| rs1259771 | 12p11.21 | 32076823 | T | 0.57 | 0.54 | 1.66 | 1.33-2.06 | 5.25E-06 | ^d^ |
| rs7715214 | 5q34 | 160626657 | T | 0.10 | 0.13 | 0.61 | 0.50-0.76 | 5.71E-06 | ^d^ |
| rs442432 | 6q27 | 169474908 | C | 0.10 | 0.08 | 1.65 | 1.33-2.04 | 6.15E-06 |  |
| rs6943931 | 7p15.3 | 22414646 | C | 0.24 | 0.28 | 0.67 | 0.56-0.80 | 6.84E-06 | ^d^ |
| rs13395141 | 2q23.1 | 148955901 | T | 0.24 | 0.29 | 0.67 | 0.56-0.80 | 7.14E-06 | ^d^ |
| rs10840257 | 11p15.4 | 9568463 | C | 0.45 | 0.39 | 1.33 | 1.17-1.51 | 1.02E-05 |  |
| rs16838616 | 1p36.32 | 4368304 | T | 0.18 | 0.21 | 0.71 | 0.61-0.83 | 1.07E-05 |  |
| rs17093425 | 20q11.23 | 34772913 | A | 0.15 | 0.18 | 0.69 | 0.59-0.82 | 1.07E-05 |  |
| rs1556990 | 10p13 | 13550822 | A | 0.05 | 0.08 | 0.55 | 0.43-0.72 | 1.17E-05 | ^d^ |
| rs1382334 | 5q34 | 160652367 | A | 0.10 | 0.13 | 0.63 | 0.51-0.77 | 1.22E-05 | ^d^ |
| rs16949855 | 16q12.1 | 51540509 | T | 0.27 | 0.29 | 0.49 | 0.36-0.68 | 1.43E-05 | ^r^ |
| rs4118071 | 11q14.1 | 80425472 | A | 0.49 | 0.52 | 0.76 | 0.68-0.86 | 1.53E-05 |  |
| rs1080826 | 5p13.2 | 34545200 | T | 0.09 | 0.12 | 0.63 | 0.51-0.78 | 1.62E-05 | ^d^ |
| rs4748042 | 10p13 | 13554693 | G | 0.05 | 0.07 | 0.56 | 0.43-0.73 | 1.68E-05 | ^d^ |
| rs1460753 | 4q21.3 | 87223468 | C | 0.58 | 0.52 | 1.31 | 1.16-1.48 | 1.80E-05 |  |
| rs4748043 | 10p13 | 13556487 | A | 0.05 | 0.07 | 0.56 | 0.43-0.73 | 1.82E-05 | ^d^ |
| rs8020941 | 14q32.2 | 97533362 | T | 0.20 | 0.22 | 0.68 | 0.57-0.81 | 1.84E-05 | ^d^ |
| rs2030074 | 11q14.1 | 80644238 | T | 0.54 | 0.50 | 1.30 | 1.15-1.47 | 1.95E-05 |  |
| rs6540495 | 1q32.2 | 209046438 | A | 0.22 | 0.26 | 0.73 | 0.64-0.85 | 1.98E-05 |  |
| rs2702996 | 8q24.22 | 134055485 | A | 0.09 | 0.11 | 0.62 | 0.50-0.77 | 1.99E-05 | ^d^ |
| rs9936891 | 16p13.2 | 7906045 | T | 0.42 | 0.38 | 1.48 | 1.23-1.77 | 2.01E-05 | ^d^ |
| rs6962291 | 7q34 | 139671117 | T | 0.36 | 0.40 | 0.76 | 0.67-0.86 | 2.01E-05 |  |
| rs12196325 | 6q27 | 166513487 | T | 0.28 | 0.24 | 1.46 | 1.23-1.74 | 2.13E-05 | ^d^ |
| rs17654300 | 19p13.11 | 17788648 | G | 0.09 | 0.12 | 0.64 | 0.52-0.79 | 2.17E-05 |  |
| rs1534537 | 2q32.3 | 197263186 | C | 0.19 | 0.15 | 1.51 | 1.25-1.82 | 2.20E-05 | ^d^ |
| rs9648284 | 7p15.3 | 22417214 | T | 0.25 | 0.29 | 0.69 | 0.58-0.82 | 2.27E-05 | ^d^ |
| rs10223391 | 6q27 | 169485408 | C | 0.37 | 0.33 | 1.31 | 1.16-1.49 | 2.29E-05 |  |
| rs10747977 | 12q14.2 | 63517883 | T | 0.14 | 0.12 | 1.56 | 1.27-1.92 | 2.31E-05 | ^d^ |
| rs6058434 | 20q11.23 | 34795372 | T | 0.43 | 0.45 | 0.62 | 0.50-0.78 | 2.41E-05 | ^r^ |
| rs2596230 | 15q14 | 33720726 | G | 0.17 | 0.14 | 1.45 | 1.22-1.73 | 2.48E-05 |  |
| rs17226520 | 7q35 | 147498340 | T | 0.15 | 0.12 | 1.48 | 1.24-1.78 | 2.49E-05 |  |
| rs864553 | 1p21.3 | 95378425 | G | 0.22 | 0.19 | 1.48 | 1.23-1.77 | 2.56E-05 | ^d^ |
| rs1725472 | 19q13.13 | 38588045 | T | 0.64 | 0.60 | 1.31 | 1.15-1.48 | 2.61E-05 |  |
| rs12179836 | 6q27 | 169485639 | G | 0.33 | 0.29 | 1.33 | 1.16-1.51 | 2.66E-05 |  |
| rs878838 | 12p11.22 | 29620687 | T | 0.10 | 0.07 | 1.61 | 1.29-2.01 | 2.84E-05 |  |
| rs11134528 | 5q34 | 168203926 | T | 0.13 | 0.11 | 1.57 | 1.27-1.93 | 2.85E-05 | ^d^ |
| rs12119864 | 1p36.32 | 5022767 | G | 0.08 | 0.10 | 0.62 | 0.49-0.77 | 2.86E-05 | ^d^ |
| rs6827098 | 4q21.3 | 87221480 | T | 0.56 | 0.50 | 1.30 | 1.15-1.47 | 2.87E-05 |  |
| rs1328183 | 1q31.2 | 193759244 | A | 0.19 | 0.22 | 0.68 | 0.57-0.82 | 3.37E-05 | ^d^ |
| rs1467404 | 6q23.2 | 133090141 | A | 0.53 | 0.47 | 1.30 | 1.15-1.47 | 3.48E-05 |  |
| rs1970728 | 11q14.1 | 80394267 | C | 0.36 | 0.39 | 0.77 | 0.68-0.87 | 3.63E-05 |  |
| rs12697291 | 5p13.3 | 33598017 | T | 0.31 | 0.35 | 0.76 | 0.67-0.86 | 3.70E-05 |  |
| rs7767855 | 6p24.3 | 10239788 | T | 0.46 | 0.43 | 1.30 | 1.15-1.47 | 3.74E-05 |  |
| rs6958656 | 7p14.2 | 37182833 | G | 0.32 | 0.29 | 1.44 | 1.21-1.72 | 3.77E-05 | ^d^ |
| rs2938783 | 5q12.1 | 59105651 | G | 0.39 | 0.37 | 1.46 | 1.22-1.74 | 3.78E-05 | ^d^ |
| rs2247732 | 20q11.23 | 34703060 | A | 0.16 | 0.18 | 0.71 | 0.61-0.84 | 3.98E-05 |  |
| rs6462731 | 7p14.2 | 37187557 | A | 0.33 | 0.30 | 1.44 | 1.21-1.71 | 3.98E-05 | ^d^ |
| rs12072357 | 1p22.3 | 88279922 | T | 0.11 | 0.14 | 0.65 | 0.53-0.80 | 4.00E-05 | ^d^ |
| rs4878640 | 9p13.3 | 36126723 | T | 0.41 | 0.37 | 1.45 | 1.22-1.74 | 4.04E-05 | ^d^ |
| rs16961587 | 15q21.1 | 49052809 | G | 0.12 | 0.10 | 1.53 | 1.25-1.88 | 4.09E-05 |  |
| rs10258404 | 7q31.32 | 122664695 | G | 0.14 | 0.10 | 1.57 | 1.27-1.95 | 4.13E-05 | ^d^ |
| rs153516 | 5q31.3 | 142979183 | A | 0.19 | 0.16 | 1.48 | 1.23-1.78 | 4.15E-05 | ^d^ |
| rs136161 | 22q12.3 | 36657432 | G | 0.78 | 0.75 | 1.45 | 1.21-1.73 | 4.17E-05 | ^r^ |
| rs7662459 | 4q35.2 | 190282697 | T | 0.51 | 0.47 | 1.29 | 1.14-1.46 | 4.18E-05 |  |
| rs6501053 | 16p13.2 | 8062273 | G | 0.15 | 0.19 | 0.72 | 0.61-0.84 | 4.34E-05 |  |
| rs1039751 | 11q21 | 94695433 | G | 0.18 | 0.20 | 0.73 | 0.62-0.85 | 4.36E-05 |  |
| rs4242276 | 6q25.1 | 151303497 | G | 0.59 | 0.54 | 1.30 | 1.14-1.47 | 4.37E-05 |  |
| rs219474 | 4q25 | 109313182 | C | 0.62 | 0.59 | 1.46 | 1.22-1.75 | 4.39E-05 | ^r^ |
| rs9493454 | 6q23.2 | 133144629 | G | 0.43 | 0.38 | 1.30 | 1.15-1.47 | 4.48E-05 |  |
| rs6852320 | 4q31.23 | 148782064 | C | 0.31 | 0.34 | 0.76 | 0.66-0.87 | 4.52E-05 |  |
| rs11206152 | 1p32.3 | 53831095 | G | 0.55 | 0.55 | 0.63 | 0.50-0.79 | 4.54E-05 | ^d^ |
| rs6000229 | 22q12.3 | 36686159 | C | 0.65 | 0.59 | 1.30 | 1.15-1.48 | 4.62E-05 |  |
| rs1452144 | 3q22.1 | 132627628 | T | 0.32 | 0.34 | 0.69 | 0.58-0.83 | 5.01E-05 | ^d^ |
| rs3915922 | 1p31.3 | 65368562 | G | 0.40 | 0.43 | 0.77 | 0.68-0.87 | 5.03E-05 |  |
| rs310196 | 1p31.3 | 65351622 | G | 0.39 | 0.43 | 0.77 | 0.68-0.87 | 5.13E-05 |  |
| rs1060463 | 19p13.12 | 16025176 | G | 0.52 | 0.55 | 0.64 | 0.52-0.79 | 5.15E-05 | ^d^ |
| rs1479842 | 7q35 | 147025943 | A | 0.40 | 0.37 | 1.44 | 1.21-1.72 | 5.27E-05 | ^d^ |
| rs1362602 | 12p12.3 | 14838469 | T | 0.56 | 0.51 | 1.53 | 1.25-1.89 | 5.36E-05 | ^d^ |
| rs12187377 | 5q12.1 | 60567241 | G | 0.35 | 0.31 | 1.31 | 1.15-1.50 | 5.48E-05 |  |
| rs16946080 | 15q22.2 | 63195611 | T | 0.05 | 0.07 | 0.59 | 0.46-0.76 | 5.56E-05 |  |
| rs7850246 | 9p21.3 | 20645480 | G | 0.15 | 0.10 | 1.52 | 1.24-1.87 | 5.62E-05 | ^d^ |
| rs11132640 | 4q35.2 | 190281768 | C | 0.51 | 0.47 | 1.29 | 1.14-1.46 | 5.75E-05 |  |
| rs7769051 | 6q23.2 | 133146796 | T | 0.34 | 0.29 | 1.31 | 1.15-1.50 | 5.78E-05 |  |
| rs12636302 | 3q26.1 | 162009921 | C | 0.39 | 0.35 | 1.30 | 1.14-1.48 | 5.88E-05 |  |
| rs4708589 | 6q27 | 169488784 | G | 0.30 | 0.27 | 1.32 | 1.15-1.52 | 5.91E-05 |  |
| rs2095270 | 13q33.1 | 101900361 | A | 0.22 | 0.20 | 1.45 | 1.21-1.73 | 5.96E-05 | ^d^ |
| rs6011478 | 20q13.33 | 61556771 | G | 0.24 | 0.27 | 0.75 | 0.65-0.86 | 6.17E-05 |  |
| rs17040826 | 4q25 | 110686776 | G | 0.34 | 0.39 | 0.70 | 0.58-0.83 | 6.26E-05 | ^d^ |
| rs1489707 | 5q34 | 160537180 | G | 0.25 | 0.21 | 1.35 | 1.17-1.57 | 6.38E-05 |  |
| rs9680776 | 22q11.1 | 17000277 | A | 0.43 | 0.39 | 1.29 | 1.14-1.46 | 6.41E-05 |  |
| rs2050757 | 9p24.3 | 1513917 | T | 0.24 | 0.28 | 0.75 | 0.66-0.87 | 6.81E-05 |  |
| rs6581470 | 12q14.2 | 63516676 | A | 0.14 | 0.12 | 1.52 | 1.24-1.86 | 7.02E-05 | ^d^ |
| rs476445 | 6q26 | 163540127 | C | 0.55 | 0.52 | 1.52 | 1.24-1.87 | 7.12E-05 | ^d^ |
| rs6723745 | 2p21 | 42090563 | A | 0.15 | 0.19 | 0.72 | 0.61-0.84 | 7.19E-05 |  |
| rs4708584 | 6q27 | 169486046 | A | 0.30 | 0.27 | 1.32 | 1.15-1.51 | 7.36E-05 |  |
| rs2956224 | 8p21.2 | 25455401 | G | 0.18 | 0.15 | 1.41 | 1.19-1.66 | 7.37E-05 |  |
| rs711635 | 3p26.1 | 4490513 | T | 0.38 | 0.34 | 1.43 | 1.20-1.70 | 7.50E-05 | ^d^ |
| rs9961535 | 18q21.1 | 48111928 | A | 0.13 | 0.15 | 0.70 | 0.59-0.84 | 7.57E-05 |  |
| rs2791131 | 1q23.3 | 163507977 | T | 0.24 | 0.21 | 1.43 | 1.20-1.71 | 7.61E-05 | ^d^ |
| rs1654365 | 19q13.13 | 38586721 | G | 0.20 | 0.23 | 0.74 | 0.64-0.86 | 7.73E-05 |  |
| rs10929676 | 2p25.1 | 10717282 | G | 0.38 | 0.34 | 1.43 | 1.20-1.71 | 7.75E-05 | ^d^ |
| rs7997640 | 13q12.3 | 30744080 | G | 0.43 | 0.45 | 0.68 | 0.57-0.83 | 7.88E-05 | ^d^ |
| rs3847427 | 10p14 | 11402047 | C | 0.48 | 0.52 | 0.78 | 0.69-0.88 | 7.89E-05 |  |
| rs6736816 | 2p16.3 | 50255589 | C | 0.18 | 0.22 | 0.70 | 0.58-0.83 | 8.00E-05 | ^d^ |
| rs1450053 | 3q22.1 | 132884215 | A | 0.52 | 0.56 | 0.78 | 0.68-0.88 | 8.23E-05 |  |
| rs7121863 | 11p14.2 | 26324119 | A | 0.06 | 0.08 | 0.61 | 0.48-0.78 | 8.26E-05 |  |
| rs1486936 | 11q13.1 | 63670387 | C | 0.36 | 0.39 | 0.70 | 0.59-0.84 | 8.29E-05 | ^d^ |
| rs9966877 | 18q21.1 | 48116918 | A | 0.13 | 0.15 | 0.70 | 0.59-0.84 | 8.29E-05 |  |
| rs6985673 | 8p11.22 | 38884176 | C | 0.14 | 0.16 | 0.68 | 0.56-0.83 | 8.41E-05 | ^d^ |
| rs10759255 | 9q31.2 | 110497951 | G | 0.33 | 0.37 | 0.70 | 0.59-0.84 | 8.41E-05 | ^d^ |
| rs17073277 | 6q21 | 112393393 | T | 0.07 | 0.09 | 0.61 | 0.48-0.78 | 8.44E-05 | ^d^ |
| rs9918936 | 9p24.3 | 1511341 | G | 0.23 | 0.27 | 0.76 | 0.66-0.87 | 8.50E-05 |  |
| rs17141842 | 11q14.1 | 80978480 | T | 0.13 | 0.15 | 0.68 | 0.56-0.82 | 8.53E-05 | ^d^ |
| rs11101416 | 10q11.22 | 49865727 | G | 0.44 | 0.46 | 0.78 | 0.69-0.88 | 8.55E-05 |  |
| rs213085 | 18q22.1 | 61721713 | A | 0.13 | 0.17 | 0.68 | 0.56-0.82 | 8.59E-05 | ^d^ |
| rs1771362 | 1p34.3 | 34661652 | G | 0.24 | 0.20 | 1.35 | 1.16-1.57 | 8.64E-05 |  |
| rs2891412 | 12q24.13 | 113318558 | A | 0.08 | 0.10 | 0.63 | 0.50-0.80 | 8.69E-05 | ^d^ |
| rs10450594 | 11p13 | 33028973 | G | 0.08 | 0.10 | 0.65 | 0.53-0.81 | 8.75E-05 |  |
| rs6451146 | 5p13.2 | 34564023 | G | 0.14 | 0.16 | 0.71 | 0.60-0.85 | 8.85E-05 |  |
| rs7281104 | 21q11.2 | 15918577 | G | 0.38 | 0.34 | 1.30 | 1.14-1.47 | 8.91E-05 |  |
| rs1614979 | 19q13.13 | 38577437 | C | 0.21 | 0.24 | 0.75 | 0.65-0.87 | 9.03E-05 |  |
| rs7214746 | 17q11.2 | 27537626 | C | 0.09 | 0.06 | 1.59 | 1.26-2.01 | 9.24E-05 |  |
| rs711633 | 3p26.1 | 4491261 | G | 0.27 | 0.23 | 1.32 | 1.15-1.52 | 9.24E-05 |  |
| rs11171911 | 12q13.3 | 57017515 | A | 0.09 | 0.11 | 0.63 | 0.50-0.80 | 9.25E-05 | ^d^ |
| rs2889222 | 2q32.3 | 197293583 | A | 0.12 | 0.09 | 1.49 | 1.22-1.83 | 9.36E-05 |  |
| rs6801601 | 3p14.1 | 65754783 | A | 0.49 | 0.47 | 1.28 | 1.13-1.45 | 9.43E-05 |  |
| rs16952149 | 18q21.1 | 48084560 | A | 0.15 | 0.18 | 0.72 | 0.61-0.85 | 9.51E-05 |  |
| rs711636 | 3p26.1 | 4489777 | G | 0.27 | 0.23 | 1.32 | 1.15-1.52 | 9.52E-05 |  |
| rs10766496 | 11p15.1 | 18738718 | T | 0.20 | 0.22 | 0.70 | 0.59-0.84 | 9.52E-05 | ^d^ |
| rs2836797 | 21q22.2 | 40345484 | G | 0.17 | 0.21 | 0.73 | 0.63-0.86 | 9.57E-05 |  |
| rs2695988 | 5q34 | 165671235 | T | 0.07 | 0.09 | 0.62 | 0.49-0.79 | 9.59E-05 | ^d^ |
| rs2590948 | 20q11.23 | 34755152 | A | 0.16 | 0.18 | 0.72 | 0.62-0.85 | 9.77E-05 |  |
| rs2531149 | 4p15.32 | 15914006 | T | 0.48 | 0.47 | 1.47 | 1.21-1.78 | 9.78E-05 | ^d^ |
| rs1923360 | 10q22.3 | 81185978 | G | 0.36 | 0.40 | 0.78 | 0.69-0.88 | 9.79E-05 |  |
| rs7316817 | 12p13.2 | 10909412 | C | 0.14 | 0.17 | 0.68 | 0.57-0.83 | 9.85E-05 | ^d^ |
| rs1395199 | 4q22.3 | 97177286 | A | 0.47 | 0.44 | 1.46 | 1.21-1.77 | 9.90E-05 | ^d^ |
| rs778933 | 4q13.1 | 63258582 | G | 0.15 | 0.18 | 0.72 | 0.62-0.85 | 9.97E-05 |  |
| rs6011473 | 20q13.33 | 61551306 | C | 0.24 | 0.27 | 0.76 | 0.66-0.87 | 1.00E-04 |  |
| rs304029 | 3p26.1 | 4545824 | G | 0.31 | 0.27 | 1.41 | 1.19-1.68 | 1.01E-04 | ^d^ |
| rs11216492 | 11q23.3 | 117536758 | C | 0.22 | 0.20 | 1.43 | 1.19-1.71 | 1.01E-04 | ^d^ |
| rs2780902 | 1p31.3 | 65329100 | A | 0.55 | 0.59 | 0.78 | 0.69-0.88 | 1.01E-04 |  |
| rs1596398 | 12q21.1 | 74046523 | A | 0.15 | 0.13 | 1.43 | 1.20-1.72 | 1.02E-04 |  |
| rs1569150 | 2q32.3 | 197294510 | C | 0.12 | 0.09 | 1.49 | 1.22-1.82 | 1.04E-04 |  |
| rs2600956 | 3q22.1 | 132624113 | T | 0.32 | 0.34 | 0.71 | 0.59-0.84 | 1.05E-04 | ^d^ |
| rs10842508 | 12p12.1 | 25371462 | T | 0.23 | 0.25 | 0.75 | 0.65-0.87 | 1.06E-04 |  |
| rs12241778 | 10q24.1 | 98851426 | A | 0.11 | 0.10 | 0.13 | 0.05-0.36 | 1.07E-04 | ^r^ |
| rs3125709 | 13q12.3 | 29952089 | C | 0.12 | 0.14 | 0.67 | 0.55-0.82 | 1.07E-04 | ^d^ |
| rs6434851 | 2q32.3 | 197280834 | T | 0.12 | 0.09 | 1.49 | 1.22-1.82 | 1.08E-04 |  |
| rs10199188 | 2q32.3 | 197292752 | C | 0.12 | 0.09 | 1.49 | 1.22-1.83 | 1.09E-04 |  |
| rs860544 | 14q12 | 25234818 | G | 0.23 | 0.25 | 0.75 | 0.65-0.87 | 1.09E-04 |  |
| rs4766613 | 12q24.11 | 110016559 | A | 0.18 | 0.21 | 0.70 | 0.58-0.84 | 1.11E-04 | ^d^ |
| rs2295001 | 20q13.33 | 61541028 | C | 0.30 | 0.34 | 0.78 | 0.68-0.88 | 1.11E-04 |  |
| rs16877964 | 5q12.1 | 59676014 | G | 0.18 | 0.20 | 0.70 | 0.58-0.84 | 1.13E-04 | ^d^ |
| rs184360 | 4p15.2 | 25362101 | C | 0.44 | 0.46 | 0.69 | 0.57-0.83 | 1.13E-04 | ^d^ |
| rs7925064 | 11q14.1 | 80627680 | T | 0.56 | 0.53 | 1.27 | 1.13-1.43 | 1.14E-04 |  |
| rs11232404 | 11q14.1 | 80657390 | T | 0.51 | 0.47 | 1.27 | 1.12-1.43 | 1.14E-04 |  |
| rs16946074 | 15q22.2 | 63194012 | G | 0.05 | 0.07 | 0.61 | 0.48-0.79 | 1.14E-04 |  |
| rs7613653 | 3p22.2 | 37878813 | G | 0.21 | 0.23 | 0.75 | 0.64-0.87 | 1.16E-04 |  |
| rs13207423 | 6q27 | 166505924 | G | 0.20 | 0.17 | 1.44 | 1.20-1.73 | 1.17E-04 | ^d^ |
| rs2427540 | 20q13.33 | 62476559 | A | 0.34 | 0.31 | 1.29 | 1.13-1.47 | 1.17E-04 |  |
| rs1492408 | 8p21.2 | 24400223 | T | 0.24 | 0.21 | 1.34 | 1.15-1.55 | 1.17E-04 |  |
| rs778934 | 4q13.1 | 63259417 | A | 0.15 | 0.18 | 0.73 | 0.62-0.85 | 1.18E-04 |  |
| rs2531899 | 17q23.1 | 58007193 | A | 0.10 | 0.08 | 1.59 | 1.26-2.02 | 1.19E-04 | ^d^ |
| rs2283846 | 22q12.1 | 28165334 | A | 0.34 | 0.39 | 0.78 | 0.69-0.89 | 1.20E-04 |  |
| rs1396626 | 1p21.3 | 96025546 | A | 0.45 | 0.49 | 0.79 | 0.70-0.89 | 1.20E-04 |  |
| rs7435763 | 4q31.23 | 148774243 | T | 0.19 | 0.22 | 0.70 | 0.58-0.84 | 1.21E-04 | ^d^ |
| rs17058759 | 5q34 | 160531764 | C | 0.24 | 0.20 | 1.34 | 1.16-1.56 | 1.21E-04 |  |
| rs31546 | 5q31.1 | 135277503 | C | 0.33 | 0.37 | 0.71 | 0.59-0.84 | 1.22E-04 | ^d^ |
| rs4733781 | 8q24.21 | 131296767 | C | 0.15 | 0.16 | 3.85 | 1.94-7.66 | 1.22E-04 | ^r^ |
| rs17777617 | 5q11.2 | 58371239 | A | 0.07 | 0.10 | 0.64 | 0.51-0.80 | 1.25E-04 |  |
| rs1458234 | 4q13.2 | 69713250 | T | 0.08 | 0.10 | 0.66 | 0.53-0.81 | 1.26E-04 |  |
| rs6495697 | 15q14 | 34929751 | C | 0.44 | 0.40 | 1.28 | 1.13-1.45 | 1.27E-04 |  |
| rs855641 | 19q13.13 | 38582383 | T | 0.19 | 0.22 | 0.75 | 0.64-0.87 | 1.28E-04 |  |
| rs3218064 | 19q12 | 30312350 | T | 0.08 | 0.06 | 1.67 | 1.29-2.18 | 1.29E-04 | ^d^ |
| rs8027939 | 15q14 | 34931364 | T | 0.44 | 0.40 | 1.28 | 1.13-1.45 | 1.29E-04 |  |
| rs3910443 | 12p11.22 | 29670262 | T | 0.08 | 0.06 | 1.60 | 1.26-2.04 | 1.29E-04 |  |
| rs1569079 | 7q11.22 | 70224743 | G | 0.07 | 0.06 | 1.68 | 1.29-2.19 | 1.30E-04 |  |
| rs3772899 | 3p12.2 | 81734097 | G | 0.24 | 0.29 | 0.77 | 0.67-0.88 | 1.31E-04 |  |
| rs11609445 | 12q24.11 | 110027100 | A | 0.18 | 0.21 | 0.70 | 0.59-0.84 | 1.31E-04 | ^d^ |
| rs1589921 | 15q11.2 | 23750045 | A | 0.10 | 0.08 | 1.54 | 1.23-1.92 | 1.31E-04 |  |
| rs931842 | 4q22.3 | 97264534 | G | 0.48 | 0.44 | 1.45 | 1.20-1.76 | 1.32E-04 | ^d^ |
| rs1970730 | 11q14.1 | 80393468 | T | 0.47 | 0.50 | 0.79 | 0.69-0.89 | 1.33E-04 |  |
| rs12549843 | 8p21.2 | 24395863 | G | 0.23 | 0.21 | 1.34 | 1.15-1.55 | 1.33E-04 |  |
| rs8080043 | 17p11.2 | 20634859 | A | 0.28 | 0.24 | 1.41 | 1.18-1.68 | 1.35E-04 | ^d^ |
| rs1443729 | 12q21.1 | 73947391 | T | 0.49 | 0.45 | 1.27 | 1.12-1.44 | 1.35E-04 |  |
| rs8124478 | 20q13.33 | 62306190 | G | 0.38 | 0.33 | 1.28 | 1.13-1.46 | 1.35E-04 |  |
| rs4598084 | 6q16.1 | 96530099 | A | 0.53 | 0.56 | 0.69 | 0.57-0.83 | 1.36E-04 | ^r^ |
| rs6891639 | 5q23.1 | 117927862 | T | 0.08 | 0.10 | 0.67 | 0.54-0.82 | 1.37E-04 |  |
| rs10081610 | 8p23.1 | 12663987 | C | 0.57 | 0.54 | 1.26 | 1.12-1.43 | 1.37E-04 |  |
| rs17353111 | 7p21.1 | 19407264 | G | 0.13 | 0.16 | 0.71 | 0.60-0.85 | 1.38E-04 |  |
| rs4384817 | 2p13.1 | 74226141 | A | 0.10 | 0.07 | 1.52 | 1.23-1.89 | 1.38E-04 |  |
| rs1979026 | 15q25.1 | 80738546 | G | 0.48 | 0.45 | 1.27 | 1.12-1.43 | 1.38E-04 |  |
| rs446649 | 20p11.21 | 25467359 | G | 0.53 | 0.49 | 1.27 | 1.12-1.44 | 1.38E-04 |  |
| rs1999435 | 9p24.3 | 1513849 | C | 0.25 | 0.29 | 0.77 | 0.67-0.88 | 1.40E-04 |  |
| rs452748 | 13q33.3 | 109481163 | C | 0.50 | 0.46 | 1.45 | 1.20-1.76 | 1.42E-04 | ^d^ |
| rs12058047 | 1q42.3 | 234891007 | A | 0.20 | 0.17 | 1.43 | 1.19-1.72 | 1.43E-04 | ^d^ |
| rs12471412 | 2q32.3 | 197274355 | C | 0.14 | 0.11 | 1.44 | 1.19-1.74 | 1.43E-04 |  |
| rs7572957 | 2p23.2 | 29487405 | C | 0.35 | 0.31 | 1.40 | 1.18-1.67 | 1.43E-04 | ^d^ |
| rs4652554 | 1q25.3 | 181032155 | T | 0.38 | 0.33 | 1.28 | 1.13-1.46 | 1.44E-04 |  |
| rs7770081 | 6q23.2 | 133089569 | T | 0.50 | 0.44 | 1.27 | 1.12-1.44 | 1.44E-04 |  |
| rs1478929 | 1p34.3 | 34663812 | T | 0.23 | 0.20 | 1.34 | 1.15-1.55 | 1.44E-04 |  |
| rs13347176 | 20q13.33 | 61532610 | G | 0.24 | 0.28 | 0.76 | 0.67-0.88 | 1.45E-04 |  |
| rs7696457 | 4p15.1 | 32947005 | T | 0.68 | 0.71 | 0.77 | 0.67-0.88 | 1.46E-04 |  |

Direction: RA is reference allele. The odds ratio (OR) is presented for the reference allele, compared with the non-reference allele, for a given model.

**Supplemental Table S5b.**

**Top 200 associations from the Discovery GWAS – American Indian**

| **SNP** | **Cytoband** | **Position** | **RA** | **Case  RAF** | **Control RAF** | **OR** | **95% CI** | **P-value** | |
| --- | --- | --- | --- | --- | --- | --- | --- | --- | --- |
| rs764596 | 12q24.21 | 115921838 | C | 0.06 | 0.12 | 0.42 | 0.28-0.62 | 1.23E-05 |  |
| rs8042933 | 15q26.3 | 100364846 | G | 0.16 | 0.09 | 2.38 | 1.61-3.52 | 1.35E-05 | ^d^ |
| rs11829064 | 12q24.21 | 115921279 | G | 0.06 | 0.12 | 0.40 | 0.26-0.61 | 1.59E-05 | ^d^ |
| rs7380299 | 5q35.3 | 180543318 | G | 0.32 | 0.39 | 0.49 | 0.35-0.68 | 1.66E-05 | ^d^ |
| rs13154832 | 5q34 | 161515020 | T | 0.29 | 0.21 | 2.02 | 1.47-2.79 | 1.70E-05 | ^d^ |
| rs2085086 | 15q26.3 | 100360092 | G | 0.16 | 0.09 | 2.35 | 1.59-3.48 | 1.90E-05 | ^d^ |
| rs3217763 | 4q27 | 122741662 | A | 0.24 | 0.16 | 1.85 | 1.39-2.46 | 2.25E-05 |  |
| rs4972722 | 2q31.1 | 175649317 | T | 0.41 | 0.33 | 1.61 | 1.29-2.01 | 3.00E-05 |  |
| rs12634258 | 3p14.2 | 61291738 | A | 0.60 | 0.50 | 1.59 | 1.27-1.98 | 3.79E-05 |  |
| rs7651261 | 3q26.33 | 181023400 | T | 0.12 | 0.06 | 2.29 | 1.54-3.39 | 3.96E-05 |  |
| rs12458786 | 18q11.2 | 23030650 | A | 0.24 | 0.31 | 0.51 | 0.38-0.71 | 3.98E-05 | ^d^ |
| rs786315 | 9q21.2 | 79241173 | C | 0.53 | 0.62 | 0.63 | 0.50-0.78 | 4.11E-05 |  |
| rs9316263 | 13q14.2 | 47766580 | C | 0.45 | 0.37 | 1.98 | 1.43-2.74 | 4.21E-05 | ^d^ |
| rs316331 | 7q11.21 | 65604622 | A | 0.18 | 0.25 | 0.56 | 0.43-0.74 | 4.42E-05 |  |
| rs1935341 | 9q21.13 | 74128003 | T | 0.12 | 0.06 | 2.47 | 1.60-3.80 | 4.43E-05 | ^d^ |
| rs11212359 | 11q22.3 | 107638761 | C | 0.17 | 0.23 | 0.50 | 0.36-0.70 | 4.46E-05 | ^d^ |
| rs8104916 | 19p13.2 | 13398235 | G | 0.23 | 0.16 | 2.00 | 1.43-2.79 | 4.55E-05 | ^d^ |
| rs11642775 | 16q22.1 | 70749997 | T | 0.64 | 0.68 | 0.33 | 0.19-0.56 | 4.66E-05 | ^d^ |
| rs7595480 | 2p25.3 | 367736 | C | 0.17 | 0.24 | 0.51 | 0.37-0.71 | 4.70E-05 | ^d^ |
| rs2283054 | 7q31.2 | 117126401 | T | 0.52 | 0.45 | 2.10 | 1.47-3.00 | 4.85E-05 | ^d^ |
| rs2366907 | 10p12.2 | 24499924 | A | 0.60 | 0.52 | 2.06 | 1.45-2.92 | 4.94E-05 | ^r^ |
| rs2132173 | 3p21.31 | 47247060 | C | 0.56 | 0.47 | 1.58 | 1.26-1.96 | 4.96E-05 |  |
| rs2305634 | 3p21.31 | 47043538 | A | 0.56 | 0.47 | 1.58 | 1.27-1.97 | 5.25E-05 |  |
| rs11688307 | 2p25.3 | 353274 | A | 0.17 | 0.24 | 0.51 | 0.37-0.71 | 5.33E-05 | ^d^ |
| rs11692082 | 2p25.3 | 370778 | C | 0.17 | 0.24 | 0.51 | 0.37-0.71 | 5.35E-05 | ^d^ |
| rs981679 | 16p13.3 | 6427786 | G | 0.64 | 0.53 | 1.62 | 1.28-2.05 | 5.49E-05 |  |
| rs4786964 | 16p13.3 | 7145567 | C | 0.19 | 0.26 | 0.58 | 0.44-0.75 | 5.50E-05 |  |
| rs4502705 | 4q21.1 | 78004668 | T | 0.35 | 0.46 | 0.63 | 0.50-0.79 | 6.03E-05 |  |
| rs11693451 | 2p25.3 | 371148 | G | 0.17 | 0.24 | 0.52 | 0.37-0.71 | 6.12E-05 | ^d^ |
| rs3936155 | 10p12.2 | 24503255 | G | 0.59 | 0.52 | 2.04 | 1.44-2.89 | 6.30E-05 | ^r^ |
| rs10739592 | 9q33.2 | 123971612 | G | 0.59 | 0.68 | 0.64 | 0.51-0.79 | 6.37E-05 |  |
| rs4256889 | 10p12.2 | 24500127 | G | 0.59 | 0.51 | 2.03 | 1.44-2.88 | 6.50E-05 | ^r^ |
| rs210987 | 5q34 | 161535755 | C | 0.30 | 0.23 | 1.90 | 1.39-2.6 | 6.53E-05 | ^d^ |
| rs4273138 | 18q12.3 | 43239395 | A | 0.14 | 0.21 | 0.55 | 0.41-0.74 | 6.62E-05 |  |
| rs6740249 | 2p25.3 | 355800 | A | 0.17 | 0.23 | 0.52 | 0.37-0.72 | 6.64E-05 | ^d^ |
| rs7610636 | 3p21.31 | 47064436 | C | 0.44 | 0.53 | 0.64 | 0.52-0.80 | 6.78E-05 |  |
| rs4730779 | 7q31.2 | 117023535 | G | 0.40 | 0.47 | 0.45 | 0.30-0.67 | 6.88E-05 | ^r^ |
| rs7379517 | 5q35.3 | 180541251 | G | 0.33 | 0.39 | 0.51 | 0.37-0.71 | 7.02E-05 | ^d^ |
| rs7808424 | 7q31.2 | 117067822 | C | 0.57 | 0.50 | 2.16 | 1.48-3.15 | 7.11E-05 | ^d^ |
| rs1636334 | 7q36.1 | 149099607 | C | 0.13 | 0.07 | 2.24 | 1.50-3.33 | 7.14E-05 |  |
| rs12636851 | 3p21.31 | 47463567 | C | 0.44 | 0.52 | 0.64 | 0.52-0.80 | 7.42E-05 |  |
| rs2157946 | 7q31.2 | 117030698 | G | 0.39 | 0.47 | 0.45 | 0.30-0.67 | 7.46E-05 | ^r^ |
| rs629548 | 19p12 | 21414082 | A | 0.09 | 0.15 | 0.51 | 0.37-0.71 | 7.50E-05 |  |
| rs11971971 | 7q11.21 | 65068631 | C | 0.20 | 0.27 | 0.59 | 0.45-0.76 | 7.58E-05 |  |
| rs4234465 | 3p21.31 | 47026885 | C | 0.43 | 0.52 | 0.65 | 0.52-0.80 | 7.62E-05 |  |
| rs12497008 | 3q26.33 | 181283743 | T | 0.65 | 0.70 | 0.52 | 0.38-0.72 | 7.64E-05 | ^r^ |
| rs6940797 | 6q15 | 90208120 | T | 0.46 | 0.40 | 2.30 | 1.52-3.47 | 7.70E-05 | ^r^ |
| rs2290545 | 3p21.31 | 46967040 | C | 0.43 | 0.51 | 0.65 | 0.52-0.80 | 8.03E-05 |  |
| rs7875405 | 9q21.33 | 89955096 | C | 0.58 | 0.48 | 1.56 | 1.25-1.95 | 8.05E-05 |  |
| rs11642770 | 16q22.1 | 70749970 | T | 0.65 | 0.68 | 0.34 | 0.20-0.58 | 8.14E-05 | ^d^ |
| rs7637487 | 3p14.2 | 61282503 | A | 0.48 | 0.38 | 1.55 | 1.25-1.93 | 8.34E-05 |  |
| rs7977987 | 12q14.3 | 65707574 | A | 0.34 | 0.25 | 1.67 | 1.29-2.15 | 8.66E-05 |  |
| rs10211178 | 2q36.1 | 221604105 | C | 0.31 | 0.25 | 1.88 | 1.37-2.57 | 8.67E-05 | ^d^ |
| rs17133180 | 7p12.2 | 49321523 | G | 0.42 | 0.51 | 0.49 | 0.34-0.70 | 8.74E-05 | ^d^ |
| rs7732687 | 5q22.2 | 111571642 | T | 0.44 | 0.50 | 0.49 | 0.34-0.70 | 8.84E-05 | ^d^ |
| rs4525874 | 3p14.1 | 69197709 | G | 0.10 | 0.15 | 0.47 | 0.33-0.69 | 8.86E-05 | ^d^ |
| rs10872032 | 6q21 | 109676776 | T | 0.31 | 0.38 | 0.61 | 0.48-0.78 | 8.92E-05 |  |
| rs7606753 | 2q36.1 | 221556078 | G | 0.30 | 0.24 | 1.87 | 1.37-2.57 | 9.04E-05 | ^d^ |
| rs6800271 | 3p21.31 | 47470787 | T | 0.44 | 0.52 | 0.65 | 0.52-0.80 | 9.07E-05 |  |
| rs2462569 | 7q11.21 | 65474846 | C | 0.20 | 0.27 | 0.59 | 0.45-0.77 | 9.39E-05 |  |
| rs4801848 | 19q13.33 | 51200073 | G | 0.12 | 0.19 | 0.55 | 0.41-0.75 | 9.48E-05 |  |
| rs7031261 | 9q21.2 | 79231317 | G | 0.40 | 0.31 | 1.60 | 1.26-2.03 | 9.48E-05 |  |
| rs2483287 | 1p36.32 | 3259677 | A | 0.37 | 0.31 | 1.87 | 1.36-2.56 | 9.50E-05 | ^d^ |
| rs2062278 | 3p21.31 | 47416761 | C | 0.44 | 0.52 | 0.65 | 0.52-0.81 | 9.97E-05 |  |
| rs709607 | 7q11.21 | 65449541 | C | 0.20 | 0.27 | 0.58 | 0.45-0.77 | 1.00E-04 |  |
| rs11085797 | 19p13.2 | 11997610 | A | 0.48 | 0.58 | 0.64 | 0.51-0.80 | 1.00E-04 |  |
| rs7628631 | 3p21.31 | 47560863 | T | 0.44 | 0.52 | 0.65 | 0.52-0.81 | 1.01E-04 |  |
| rs295442 | 3p21.31 | 47335881 | A | 0.43 | 0.51 | 0.65 | 0.52-0.81 | 1.02E-04 |  |
| rs17670646 | 18q12.3 | 43240831 | T | 0.14 | 0.21 | 0.56 | 0.42-0.75 | 1.02E-04 |  |
| rs4747482 | 10p12.2 | 24506608 | G | 0.59 | 0.52 | 1.99 | 1.41-2.81 | 1.02E-04 | ^r^ |
| rs4727849 | 7q31.2 | 117014072 | T | 0.40 | 0.47 | 0.46 | 0.31-0.68 | 1.04E-04 | ^r^ |
| rs9877709 | 3p14.2 | 61282140 | C | 0.48 | 0.38 | 1.54 | 1.24-1.92 | 1.07E-04 |  |
| rs11945401 | 4q21.1 | 78026629 | G | 0.35 | 0.46 | 0.64 | 0.51-0.80 | 1.07E-04 |  |
| rs12230075 | 12p12.1 | 25557895 | G | 0.23 | 0.30 | 0.61 | 0.48-0.78 | 1.07E-04 |  |
| rs12655488 | 5q34 | 161609025 | A | 0.29 | 0.20 | 1.89 | 1.37-2.61 | 1.07E-04 | ^d^ |
| rs2582908 | 11p14.1 | 28522535 | C | 0.26 | 0.20 | 1.70 | 1.30-2.23 | 1.08E-04 |  |
| rs4718269 | 7q11.21 | 65200778 | C | 0.20 | 0.27 | 0.59 | 0.45-0.77 | 1.08E-04 |  |
| rs12536069 | 7p22.1 | 4909470 | C | 0.11 | 0.16 | 0.49 | 0.34-0.70 | 1.08E-04 | ^d^ |
| rs12698509 | 7q11.21 | 65418876 | T | 0.20 | 0.27 | 0.59 | 0.45-0.77 | 1.09E-04 |  |
| rs1464614 | 3p21.31 | 47654375 | C | 0.51 | 0.44 | 1.55 | 1.24-1.94 | 1.13E-04 |  |
| rs10781028 | 9q21.13 | 74168943 | C | 0.11 | 0.06 | 2.38 | 1.53-3.70 | 1.13E-04 | ^d^ |
| rs183294 | 5q34 | 161498737 | A | 0.29 | 0.22 | 1.87 | 1.36-2.57 | 1.14E-04 | ^d^ |
| rs313813 | 7q11.21 | 65503500 | T | 0.20 | 0.27 | 0.59 | 0.45-0.77 | 1.15E-04 |  |
| rs10989157 | 9q22.32 | 98388345 | T | 0.40 | 0.45 | 0.51 | 0.37-0.72 | 1.16E-04 | ^d^ |
| rs16952209 | 17q22 | 50866238 | G | 0.29 | 0.19 | 1.68 | 1.29-2.20 | 1.16E-04 |  |
| rs4727851 | 7q31.2 | 117048082 | C | 0.40 | 0.47 | 0.46 | 0.31-0.68 | 1.16E-04 | ^r^ |
| rs9313913 | 5q34 | 161603958 | A | 0.30 | 0.22 | 1.86 | 1.36-2.55 | 1.18E-04 | ^d^ |
| rs160643 | 7q11.21 | 65558222 | T | 0.18 | 0.25 | 0.58 | 0.44-0.77 | 1.19E-04 |  |
| rs11612273 | 12q14.3 | 65707858 | C | 0.33 | 0.25 | 1.65 | 1.28-2.13 | 1.19E-04 |  |
| rs3816779 | 3p21.31 | 47543389 | T | 0.50 | 0.42 | 1.54 | 1.24-1.93 | 1.21E-04 |  |
| rs4274776 | 3p21.31 | 47647358 | G | 0.51 | 0.44 | 1.55 | 1.24-1.94 | 1.23E-04 |  |
| rs7110331 | 11p15.5 | 1683564 | G | 0.24 | 0.18 | 1.88 | 1.36-2.61 | 1.24E-04 | ^d^ |
| rs2243480 | 7q11.21 | 65599196 | T | 0.18 | 0.25 | 0.58 | 0.44-0.77 | 1.26E-04 |  |
| rs2116441 | 2p16.2 | 54995746 | C | 0.32 | 0.27 | 3.42 | 1.82-6.43 | 1.28E-04 | ^r^ |
| rs2897642 | 11q22.1 | 99812735 | T | 0.34 | 0.39 | 0.54 | 0.39-0.74 | 1.30E-04 | ^d^ |
| rs698468 | 9q31.2 | 109809970 | A | 0.55 | 0.47 | 1.55 | 1.24-1.95 | 1.32E-04 |  |
| rs2029945 | 5q34 | 162962672 | T | 0.57 | 0.50 | 2.00 | 1.40-2.86 | 1.33E-04 | ^r^ |
| rs11130137 | 3p21.31 | 47578838 | T | 0.56 | 0.48 | 1.53 | 1.23-1.91 | 1.33E-04 |  |
| rs11642920 | 16q24.1 | 85211552 | G | 0.36 | 0.48 | 0.65 | 0.52-0.81 | 1.34E-04 |  |
| rs211014 | 5q34 | 161576418 | T | 0.30 | 0.22 | 1.85 | 1.35-2.54 | 1.34E-04 | ^d^ |
| rs647625 | 5q34 | 161582777 | T | 0.30 | 0.22 | 1.85 | 1.35-2.54 | 1.34E-04 | ^d^ |
| rs2061197 | 3p21.31 | 47001350 | T | 0.43 | 0.51 | 0.66 | 0.53-0.82 | 1.34E-04 |  |
| rs8180040 | 3p21.31 | 47388947 | A | 0.43 | 0.51 | 0.65 | 0.52-0.81 | 1.35E-04 |  |
| rs10989496 | 9q22.32 | 98397006 | T | 0.40 | 0.45 | 0.52 | 0.37-0.73 | 1.38E-04 | ^d^ |
| rs12534221 | 7q32.3 | 131287990 | A | 0.38 | 0.32 | 1.86 | 1.35-2.55 | 1.39E-04 | ^d^ |
| rs4683301 | 3p21.31 | 46931478 | A | 0.54 | 0.45 | 1.54 | 1.23-1.92 | 1.39E-04 |  |
| rs16852004 | 3q23 | 141646962 | A | 0.67 | 0.73 | 0.62 | 0.49-0.79 | 1.40E-04 |  |
| rs7432694 | 3q13.13 | 110930787 | T | 0.46 | 0.40 | 1.88 | 1.36-2.60 | 1.41E-04 | ^d^ |
| rs12497655 | 3q13.31 | 116764010 | G | 0.26 | 0.33 | 0.55 | 0.40-0.75 | 1.43E-04 | ^d^ |
| rs12131795 | 1q23.3 | 162350635 | A | 0.14 | 0.19 | 0.55 | 0.41-0.75 | 1.44E-04 |  |
| rs6477556 | 9q31.2 | 109810348 | G | 0.54 | 0.47 | 1.55 | 1.24-1.94 | 1.45E-04 |  |
| rs134933 | 22q12.1 | 27425790 | A | 0.54 | 0.64 | 0.64 | 0.51-0.81 | 1.46E-04 |  |
| rs210994 | 5q34 | 161619014 | T | 0.31 | 0.23 | 1.84 | 1.34-2.52 | 1.47E-04 | ^d^ |
| rs11128115 | 3p14.1 | 69203054 | G | 0.10 | 0.15 | 0.49 | 0.34-0.71 | 1.47E-04 | ^d^ |
| rs6778830 | 3p14.1 | 69188122 | G | 0.10 | 0.15 | 0.49 | 0.34-0.71 | 1.47E-04 | ^d^ |
| rs10248393 | 7q31.2 | 116998176 | A | 0.60 | 0.53 | 2.13 | 1.44-3.15 | 1.47E-04 | ^d^ |
| rs7628747 | 3p21.31 | 47001990 | T | 0.57 | 0.49 | 1.52 | 1.22-1.89 | 1.47E-04 |  |
| rs9961444 | 18p11.31 | 5733654 | T | 0.14 | 0.09 | 2.02 | 1.41-2.92 | 1.48E-04 |  |
| rs989694 | 5q34 | 161564481 | G | 0.39 | 0.30 | 1.83 | 1.34-2.50 | 1.51E-04 | ^d^ |
| rs383402 | 7q11.21 | 65586653 | A | 0.18 | 0.25 | 0.59 | 0.45-0.77 | 1.52E-04 |  |
| rs2407306 | 11q22.1 | 99812967 | A | 0.34 | 0.39 | 0.54 | 0.39-0.74 | 1.52E-04 | ^d^ |
| rs4673826 | 2q34 | 215285673 | A | 0.10 | 0.16 | 0.50 | 0.35-0.71 | 1.54E-04 | ^d^ |
| rs9617449 | 22q13.32 | 49192163 | T | 0.79 | 0.87 | 0.55 | 0.41-0.75 | 1.55E-04 |  |
| rs4259369 | 7p12.2 | 49326514 | G | 0.44 | 0.52 | 0.50 | 0.35-0.71 | 1.56E-04 | ^d^ |
| rs6006524 | 22q13.2 | 44058677 | T | 0.12 | 0.16 | 0.49 | 0.34-0.71 | 1.56E-04 | ^d^ |
| rs10213803 | 5q34 | 161630286 | A | 0.31 | 0.23 | 1.84 | 1.34-2.52 | 1.58E-04 | ^d^ |
| rs11669683 | 19q13.32 | 46408356 | C | 0.10 | 0.15 | 0.52 | 0.37-0.73 | 1.59E-04 |  |
| rs4148689 | 7q31.2 | 117143602 | G | 0.52 | 0.46 | 1.99 | 1.39-2.85 | 1.59E-04 | ^d^ |
| rs4452186 | 2p25.3 | 392767 | T | 0.23 | 0.29 | 0.61 | 0.47-0.79 | 1.60E-04 |  |
| rs7734935 | 5q34 | 161596680 | A | 0.31 | 0.23 | 1.84 | 1.34-2.52 | 1.60E-04 | ^d^ |
| rs784788 | 9q31.2 | 109811529 | A | 0.55 | 0.47 | 1.55 | 1.23-1.94 | 1.61E-04 |  |
| rs11675742 | 2q36.1 | 221630579 | G | 0.30 | 0.24 | 1.83 | 1.34-2.51 | 1.61E-04 | ^d^ |
| rs784790 | 9q31.2 | 109812226 | T | 0.55 | 0.47 | 1.55 | 1.23-1.94 | 1.61E-04 |  |
| rs6468284 | 8p12 | 30172267 | G | 0.68 | 0.75 | 0.61 | 0.47-0.79 | 1.63E-04 |  |
| rs17763208 | 4p15.1 | 30455150 | G | 0.53 | 0.47 | 1.97 | 1.39-2.81 | 1.63E-04 | ^d^ |
| rs17070580 | 6q21 | 109738714 | C | 0.30 | 0.37 | 0.63 | 0.49-0.80 | 1.63E-04 |  |
| rs11617287 | 13q12.2 | 27859588 | T | 0.23 | 0.30 | 0.55 | 0.40-0.75 | 1.64E-04 | ^d^ |
| rs7704963 | 5q35.3 | 178436854 | G | 0.43 | 0.37 | 1.87 | 1.35-2.60 | 1.64E-04 | ^d^ |
| rs11154896 | 6q23.3 | 137253534 | G | 0.28 | 0.36 | 0.63 | 0.50-0.80 | 1.64E-04 |  |
| rs6570112 | 6q23.3 | 137249971 | T | 0.28 | 0.36 | 0.63 | 0.50-0.80 | 1.64E-04 |  |
| rs6570115 | 6q23.3 | 137253162 | C | 0.72 | 0.64 | 1.58 | 1.24-2.00 | 1.64E-04 |  |
| rs937863 | 3p14.1 | 69170848 | C | 0.10 | 0.15 | 0.49 | 0.34-0.71 | 1.64E-04 | ^d^ |
| rs4858888 | 3p21.31 | 47405305 | T | 0.44 | 0.52 | 0.66 | 0.53-0.82 | 1.67E-04 |  |
| rs9316279 | 13q14.2 | 47841615 | A | 0.20 | 0.13 | 1.95 | 1.38-2.76 | 1.67E-04 | ^d^ |
| rs17085484 | 13q12.2 | 27930039 | G | 0.58 | 0.49 | 1.51 | 1.22-1.87 | 1.67E-04 |  |
| rs4574109 | 2q36.1 | 221626421 | A | 0.30 | 0.25 | 1.83 | 1.33-2.50 | 1.68E-04 | ^d^ |
| rs1896886 | 7q31.2 | 117200510 | A | 0.52 | 0.46 | 1.99 | 1.39-2.85 | 1.70E-04 | ^d^ |
| rs12495221 | 3p21.31 | 48179178 | C | 0.44 | 0.36 | 1.54 | 1.23-1.93 | 1.70E-04 |  |
| rs12454702 | 18q12.3 | 43236930 | G | 0.13 | 0.19 | 0.57 | 0.42-0.76 | 1.73E-04 |  |
| rs9854461 | 3q26.33 | 181310591 | T | 0.65 | 0.70 | 0.54 | 0.39-0.74 | 1.73E-04 | ^r^ |
| rs2534744 | 16p13.3 | 6425600 | T | 0.65 | 0.55 | 1.55 | 1.23-1.94 | 1.73E-04 |  |
| rs1572280 | 9q22.32 | 98401842 | C | 0.40 | 0.45 | 0.52 | 0.37-0.73 | 1.74E-04 | ^d^ |
| rs17569795 | 14q12 | 26688690 | T | 0.49 | 0.42 | 1.54 | 1.23-1.93 | 1.74E-04 |  |
| rs13254600 | 8q24.13 | 124089526 | T | 0.20 | 0.27 | 0.55 | 0.40-0.75 | 1.74E-04 | ^d^ |
| rs6078266 | 20p12.2 | 11602966 | T | 0.39 | 0.48 | 0.52 | 0.37-0.73 | 1.74E-04 | ^d^ |
| rs3765155 | 3q23 | 141641015 | C | 0.67 | 0.73 | 0.63 | 0.49-0.80 | 1.75E-04 |  |
| rs6779171 | 3p14.1 | 69188396 | C | 0.10 | 0.15 | 0.49 | 0.34-0.71 | 1.75E-04 | ^d^ |
| rs2428192 | 6q21 | 110787371 | T | 0.36 | 0.30 | 1.83 | 1.33-2.50 | 1.76E-04 | ^d^ |
| rs4965545 | 15q26.3 | 100366384 | T | 0.12 | 0.06 | 2.32 | 1.49-3.60 | 1.78E-04 | ^d^ |
| rs1037754 | 15q14 | 35299625 | A | 0.23 | 0.16 | 1.71 | 1.29-2.26 | 1.78E-04 |  |
| rs2293226 | 3p21.31 | 47626782 | G | 0.51 | 0.44 | 1.53 | 1.23-1.92 | 1.80E-04 |  |
| rs17104928 | 14q13.3 | 37142554 | T | 0.07 | 0.12 | 0.47 | 0.31-0.70 | 1.81E-04 | ^d^ |
| rs9885646 | 6q21 | 109742460 | T | 0.30 | 0.37 | 0.63 | 0.49-0.80 | 1.82E-04 |  |
| rs12520313 | 5q34 | 162965169 | G | 0.57 | 0.50 | 1.98 | 1.38-2.83 | 1.82E-04 | ^r^ |
| rs210997 | 5q34 | 161617523 | T | 0.31 | 0.23 | 1.83 | 1.33-2.50 | 1.83E-04 | ^d^ |
| rs1404147 | 7q11.21 | 65264524 | T | 0.20 | 0.27 | 0.61 | 0.47-0.79 | 1.84E-04 |  |
| rs4701026 | 5q35.3 | 178458214 | T | 0.34 | 0.43 | 0.65 | 0.51-0.81 | 1.84E-04 |  |
| rs4933622 | 10q23.1 | 82672226 | T | 0.38 | 0.41 | 0.44 | 0.29-0.68 | 1.85E-04 | ^r^ |
| rs80515 | 22q13.32 | 49178311 | T | 0.11 | 0.05 | 2.31 | 1.49-3.57 | 1.85E-04 |  |
| rs5746892 | 22q11.1 | 17258201 | G | 0.43 | 0.34 | 1.84 | 1.34-2.54 | 1.86E-04 | ^d^ |
| rs4767365 | 12q24.21 | 116187565 | T | 0.13 | 0.17 | 0.51 | 0.36-0.73 | 1.86E-04 | ^d^ |
| rs6078265 | 20p12.2 | 11599971 | C | 0.39 | 0.48 | 0.52 | 0.37-0.74 | 1.88E-04 | ^d^ |
| rs2366447 | 4q13.3 | 73633046 | A | 0.47 | 0.39 | 1.52 | 1.22-1.90 | 1.88E-04 |  |
| rs7205063 | 16q24.1 | 86981532 | T | 0.27 | 0.21 | 1.64 | 1.27-2.13 | 1.89E-04 |  |
| rs495746 | 5q34 | 161624883 | T | 0.32 | 0.24 | 1.81 | 1.33-2.48 | 1.91E-04 | ^d^ |
| rs11013962 | 10p12.2 | 24495586 | C | 0.41 | 0.49 | 0.65 | 0.52-0.81 | 1.94E-04 |  |
| rs724815 | 14q12 | 26596160 | T | 0.24 | 0.29 | 0.55 | 0.40-0.75 | 1.94E-04 | ^d^ |
| rs10461018 | 3p21.31 | 46995242 | T | 0.43 | 0.51 | 0.66 | 0.54-0.82 | 1.94E-04 |  |
| rs574445 | 3q25.1 | 150594685 | T | 0.45 | 0.40 | 1.87 | 1.34-2.59 | 1.94E-04 | ^d^ |
| rs17002335 | 4q21.1 | 77952542 | T | 0.37 | 0.46 | 0.65 | 0.52-0.82 | 1.97E-04 |  |
| rs7106726 | 11p15.4 | 7232673 | A | 0.40 | 0.32 | 1.58 | 1.24-2.01 | 1.99E-04 |  |
| rs17216707 | 20q13.2 | 52732362 | G | 0.33 | 0.39 | 0.65 | 0.51-0.81 | 2.03E-04 |  |
| rs262750 | 5q34 | 165049326 | A | 0.16 | 0.22 | 0.59 | 0.45-0.78 | 2.03E-04 |  |
| rs43025 | 7q31.2 | 117104507 | T | 0.55 | 0.49 | 2.01 | 1.39-2.90 | 2.03E-04 | ^d^ |
| rs8067341 | 17q21.31 | 41079374 | C | 0.41 | 0.32 | 1.55 | 1.23-1.95 | 2.03E-04 |  |
| rs4321767 | 5q34 | 162965894 | C | 0.43 | 0.50 | 0.51 | 0.36-0.73 | 2.04E-04 | ^d^ |
| rs211015 | 5q34 | 161575653 | T | 0.30 | 0.23 | 1.82 | 1.33-2.50 | 2.04E-04 | ^d^ |
| rs10497364 | 2q31.1 | 171236108 | T | 0.30 | 0.23 | 1.62 | 1.26-2.09 | 2.04E-04 |  |
| rs12711963 | 2p25.2 | 4973678 | C | 0.07 | 0.11 | 0.48 | 0.33-0.71 | 2.04E-04 |  |
| rs211000 | 5q34 | 161615430 | C | 0.32 | 0.24 | 1.81 | 1.32-2.48 | 2.05E-04 | ^d^ |
| rs10764398 | 10p12.2 | 23566105 | T | 0.50 | 0.43 | 1.88 | 1.35-2.63 | 2.06E-04 | ^d^ |
| rs4359107 | 10p12.2 | 23552510 | G | 0.50 | 0.43 | 1.88 | 1.35-2.63 | 2.06E-04 | ^d^ |
| rs10252350 | 7q31.2 | 116998882 | A | 0.60 | 0.53 | 2.09 | 1.42-3.09 | 2.06E-04 | ^d^ |
| rs1403579 | 3p21.31 | 47659379 | G | 0.51 | 0.44 | 1.53 | 1.22-1.91 | 2.07E-04 |  |
| rs2163339 | 7p12.2 | 49330688 | T | 0.44 | 0.52 | 0.50 | 0.35-0.72 | 2.07E-04 | ^d^ |
| rs2497402 | 13q31.1 | 85851824 | C | 0.52 | 0.43 | 1.55 | 1.23-1.96 | 2.08E-04 |  |
| rs1969025 | 5q35.3 | 178425192 | G | 0.44 | 0.37 | 1.86 | 1.34-2.58 | 2.08E-04 | ^d^ |
| rs6779055 | 3p14.1 | 69188280 | C | 0.10 | 0.15 | 0.50 | 0.34-0.72 | 2.09E-04 | ^d^ |
| rs7331313 | 13q13.3 | 37990293 | T | 0.28 | 0.22 | 1.61 | 1.25-2.08 | 2.11E-04 |  |
| rs2211045 | 1q32.2 | 209054775 | G | 0.22 | 0.28 | 0.55 | 0.40-0.75 | 2.11E-04 | ^d^ |

Direction: RA is reference allele. The odds ratio (OR) is presented for the reference allele, compared with the non-reference allele, for a given model.

**Supplemental Table S5c.**

**Top 200 associations from the Discovery GWAS – European Ancestry**

| **SNP** | **Cytoband** | **Position** | **RA** | **Case  RAF** | **Control RAF** | **OR** | **95% CI** | **P-value** | |
| --- | --- | --- | --- | --- | --- | --- | --- | --- | --- |
| rs1182671 | 9p22.1 | 19751149 | C | 0.40 | 0.29 | 2.12 | 1.54-2.92 | 3.77E-06 | ^d^ |
| rs12531478 | 7p21.2 | 15239894 | A | 0.16 | 0.09 | 2.24 | 1.59-3.15 | 3.96E-06 |  |
| rs4887445 | 15q25.3 | 86549964 | G | 0.40 | 0.34 | 2.95 | 1.85-4.69 | 4.98E-06 | ^r^ |
| rs10869882 | 9q21.2 | 79681226 | G | 0.37 | 0.29 | 2.08 | 1.51-2.85 | 6.56E-06 | ^d^ |
| rs217682 | 14q23.2 | 62356724 | G | 0.44 | 0.34 | 1.74 | 1.36-2.22 | 8.22E-06 |  |
| rs7087529 | 10q26.13 | 125711882 | C | 0.44 | 0.35 | 2.10 | 1.51-2.91 | 1.03E-05 | ^d^ |
| rs1969542 | 11p15.2 | 14034815 | G | 0.29 | 0.38 | 0.59 | 0.46-0.74 | 1.14E-05 |  |
| rs7424620 | 2q37.1 | 235416983 | A | 0.17 | 0.09 | 2.35 | 1.60-3.44 | 1.19E-05 | ^d^ |
| rs4849989 | 2p25.3 | 3814727 | G | 0.30 | 0.42 | 0.59 | 0.46-0.75 | 1.22E-05 |  |
| rs10861797 | 12q21.2 | 79686699 | A | 0.41 | 0.33 | 2.07 | 1.49-2.87 | 1.37E-05 | ^d^ |
| rs2348145 | 15q25.3 | 87878603 | C | 0.36 | 0.25 | 1.71 | 1.34-2.18 | 1.62E-05 |  |
| rs6496293 | 15q25.3 | 86522776 | G | 0.42 | 0.35 | 2.69 | 1.71-4.23 | 1.88E-05 | ^r^ |
| rs7170230 | 15q25.3 | 86522943 | C | 0.42 | 0.35 | 2.68 | 1.70-4.22 | 1.96E-05 | ^r^ |
| rs12148198 | 15q26.1 | 90121428 | G | 0.31 | 0.37 | 0.28 | 0.15-0.50 | 2.11E-05 | ^r^ |
| rs12410214 | 1q41 | 219805224 | C | 0.30 | 0.21 | 1.73 | 1.34-2.23 | 2.51E-05 |  |
| rs4399516 | 15q26.1 | 90158180 | T | 0.30 | 0.37 | 0.28 | 0.16-0.51 | 2.66E-05 | ^r^ |
| rs2024077 | 12p13.33 | 2450595 | G | 0.39 | 0.28 | 1.97 | 1.43-2.70 | 2.75E-05 | ^d^ |
| rs11145266 | 9q21.2 | 79689203 | A | 0.39 | 0.31 | 1.97 | 1.43-2.71 | 3.02E-05 | ^d^ |
| rs41009 | 2p25.1 | 8095158 | T | 0.12 | 0.17 | 0.47 | 0.33-0.67 | 3.04E-05 | ^d^ |
| rs12405272 | 1q41 | 219842739 | T | 0.28 | 0.19 | 1.74 | 1.34-2.26 | 3.08E-05 |  |
| rs11023983 | 11p15.1 | 16566923 | G | 0.52 | 0.44 | 2.22 | 1.53-3.23 | 3.10E-05 | ^r^ |
| rs11073610 | 15q25.3 | 86526743 | G | 0.41 | 0.34 | 2.62 | 1.66-4.13 | 3.17E-05 | ^r^ |
| rs13047322 | 21q22.13 | 38171655 | T | 0.32 | 0.23 | 1.70 | 1.32-2.18 | 3.39E-05 |  |
| rs12538095 | 7p21.2 | 15242206 | C | 0.17 | 0.11 | 2.26 | 1.54-3.33 | 3.56E-05 | ^d^ |
| rs4796906 | 18p11.22 | 10838059 | G | 0.58 | 0.64 | 0.40 | 0.26-0.62 | 3.66E-05 | ^d^ |
| rs609203 | 20p13 | 3670945 | A | 0.27 | 0.19 | 1.96 | 1.42-2.69 | 3.76E-05 | ^d^ |
| rs17769186 | 2q24.3 | 167348363 | A | 0.19 | 0.28 | 0.51 | 0.37-0.70 | 3.82E-05 | ^d^ |
| rs16981265 | 20p11.23 | 19860135 | T | 0.13 | 0.06 | 2.28 | 1.54-3.38 | 3.98E-05 |  |
| rs10746103 | 12q21.2 | 79697287 | A | 0.41 | 0.33 | 1.98 | 1.43-2.74 | 4.26E-05 | ^d^ |
| rs10861796 | 12q21.2 | 79686676 | G | 0.41 | 0.33 | 1.98 | 1.43-2.74 | 4.26E-05 | ^d^ |
| rs2119606 | 15q26.1 | 90150943 | A | 0.30 | 0.36 | 0.29 | 0.16-0.53 | 4.52E-05 | ^r^ |
| rs6420033 | 5p15.33 | 1922870 | C | 0.13 | 0.20 | 0.52 | 0.38-0.71 | 4.64E-05 |  |
| rs2122926 | 15q25.3 | 87879196 | G | 0.35 | 0.25 | 1.68 | 1.31-2.15 | 4.80E-05 |  |
| rs7315303 | 12q21.2 | 79578860 | G | 0.41 | 0.34 | 1.97 | 1.42-2.74 | 4.89E-05 | ^d^ |
| rs6415276 | 7p13 | 43449174 | G | 0.36 | 0.43 | 0.39 | 0.25-0.62 | 4.92E-05 | ^r^ |
| rs17458706 | 5p13.1 | 38848813 | C | 0.32 | 0.23 | 1.68 | 1.31-2.16 | 5.03E-05 |  |
| rs6979418 | 7q36.1 | 152424679 | C | 0.65 | 0.74 | 0.60 | 0.47-0.77 | 5.16E-05 |  |
| rs6791324 | 3p14.1 | 67437836 | A | 0.11 | 0.18 | 0.52 | 0.38-0.71 | 5.18E-05 |  |
| rs9943482 | 11p12 | 41074215 | T | 0.60 | 0.52 | 2.36 | 1.56-3.58 | 5.30E-05 | ^d^ |
| rs11922584 | 3p14.1 | 67442818 | T | 0.11 | 0.18 | 0.52 | 0.38-0.71 | 5.45E-05 |  |
| rs4531033 | 8q24.22 | 132209926 | C | 0.57 | 0.46 | 1.59 | 1.27-1.99 | 5.46E-05 |  |
| rs1995626 | 4q28.1 | 125812575 | C | 0.17 | 0.26 | 0.51 | 0.37-0.71 | 5.46E-05 | ^d^ |
| rs7074930 | 10p12.31 | 20562165 | T | 0.29 | 0.21 | 1.73 | 1.32-2.25 | 5.47E-05 |  |
| rs7495191 | 15q25.3 | 86485291 | A | 0.39 | 0.33 | 2.64 | 1.65-4.24 | 5.48E-05 | ^r^ |
| rs10906396 | 10p13 | 13551876 | A | 0.30 | 0.40 | 0.61 | 0.48-0.78 | 5.50E-05 |  |
| rs8127309 | 21q22.13 | 38172737 | G | 0.38 | 0.28 | 1.61 | 1.28-2.02 | 5.64E-05 |  |
| rs10055107 | 5q21.3 | 108651741 | T | 0.18 | 0.10 | 1.98 | 1.42-2.75 | 5.64E-05 |  |
| rs274717 | 5p15.31 | 6721067 | C | 0.31 | 0.35 | 0.34 | 0.20-0.58 | 5.71E-05 | ^r^ |
| rs13134269 | 4q28.2 | 129412788 | C | 0.19 | 0.28 | 0.59 | 0.46-0.77 | 5.76E-05 |  |
| rs11083386 | 18q12.1 | 27760598 | G | 0.46 | 0.56 | 0.63 | 0.51-0.79 | 5.87E-05 |  |
| rs10734121 | 11q14.3 | 89656239 | G | 0.19 | 0.13 | 1.92 | 1.40-2.64 | 5.94E-05 |  |
| rs2293650 | 12q21.2 | 79690484 | T | 0.34 | 0.28 | 1.92 | 1.40-2.65 | 6.06E-05 | ^d^ |
| rs4725769 | 7q36.1 | 147930789 | T | 0.32 | 0.24 | 1.72 | 1.32-2.24 | 6.24E-05 |  |
| rs6073312 | 20q13.12 | 42709034 | T | 0.08 | 0.14 | 0.48 | 0.33-0.69 | 6.29E-05 |  |
| rs10930226 | 2q24.3 | 167346017 | T | 0.20 | 0.29 | 0.52 | 0.38-0.72 | 6.44E-05 | ^d^ |
| rs17750221 | 4q12 | 55345487 | T | 0.38 | 0.46 | 0.51 | 0.37-0.71 | 6.49E-05 | ^d^ |
| rs17750246 | 4q12 | 55345973 | A | 0.38 | 0.46 | 0.51 | 0.37-0.71 | 6.49E-05 | ^d^ |
| rs2412586 | 4q12 | 55347852 | A | 0.38 | 0.46 | 0.51 | 0.37-0.71 | 6.49E-05 | ^d^ |
| rs17458741 | 5p13.1 | 38849383 | T | 0.32 | 0.23 | 1.66 | 1.29-2.14 | 6.85E-05 |  |
| rs10861795 | 12q21.2 | 79686542 | G | 0.41 | 0.33 | 1.94 | 1.40-2.69 | 6.86E-05 | ^d^ |
| rs12202296 | 6p22.1 | 29908469 | C | 0.37 | 0.30 | 1.90 | 1.39-2.61 | 6.95E-05 | ^d^ |
| rs1564179 | 8p23.2 | 4469330 | C | 0.60 | 0.68 | 0.62 | 0.49-0.78 | 6.99E-05 |  |
| rs6968572 | 7p21.3 | 8356809 | T | 0.59 | 0.48 | 1.57 | 1.25-1.95 | 7.12E-05 |  |
| rs11088226 | 21q22.11 | 33925531 | G | 0.27 | 0.19 | 1.73 | 1.32-2.26 | 7.19E-05 |  |
| rs4726929 | 7q36.1 | 147934639 | C | 0.32 | 0.24 | 1.69 | 1.30-2.19 | 7.25E-05 |  |
| rs11011907 | 10p12.31 | 20539426 | T | 0.28 | 0.20 | 1.72 | 1.31-2.24 | 7.32E-05 |  |
| rs16920199 | 10p12.31 | 20557095 | C | 0.28 | 0.20 | 1.73 | 1.32-2.27 | 7.42E-05 |  |
| rs4369215 | 1q43 | 242708560 | G | 0.15 | 0.09 | 2.15 | 1.47-3.13 | 7.43E-05 | ^d^ |
| rs10762786 | 10q22.3 | 80225164 | T | 0.34 | 0.43 | 0.52 | 0.37-0.72 | 7.47E-05 | ^d^ |
| rs2419027 | 18q12.1 | 27785716 | C | 0.46 | 0.56 | 0.64 | 0.51-0.80 | 7.58E-05 |  |
| rs547144 | 10q22.3 | 79012469 | C | 0.49 | 0.58 | 0.64 | 0.51-0.80 | 7.74E-05 |  |
| rs17561170 | 16q21 | 64475113 | T | 0.13 | 0.06 | 2.20 | 1.49-3.25 | 7.77E-05 |  |
| rs2099610 | 2q23.3 | 150784214 | T | 0.33 | 0.42 | 0.62 | 0.49-0.79 | 7.83E-05 |  |
| rs4702140 | 5p15.1 | 16334639 | A | 0.13 | 0.07 | 2.28 | 1.51-3.42 | 7.84E-05 | ^d^ |
| rs2169673 | 10q22.3 | 78989637 | T | 0.46 | 0.55 | 0.64 | 0.52-0.80 | 7.91E-05 |  |
| rs6901838 | 6p12.1 | 56195276 | T | 0.32 | 0.38 | 0.36 | 0.21-0.59 | 8.09E-05 | ^r^ |
| rs12481305 | 20p11.23 | 19854409 | A | 0.12 | 0.06 | 2.21 | 1.49-3.28 | 8.10E-05 |  |
| rs4744821 | 9q21.2 | 79666678 | A | 0.29 | 0.23 | 1.89 | 1.38-2.59 | 8.13E-05 | ^d^ |
| rs13227748 | 7p15.3 | 23652561 | C | 0.32 | 0.23 | 1.88 | 1.37-2.58 | 8.28E-05 | ^d^ |
| rs1534701 | 7q36.1 | 147936563 | T | 0.32 | 0.24 | 1.68 | 1.30-2.18 | 8.31E-05 |  |
| rs41001 | 2p25.1 | 8091474 | A | 0.09 | 0.14 | 0.46 | 0.32-0.68 | 8.37E-05 | ^d^ |
| rs4968578 | 17q23.2 | 59702981 | A | 0.40 | 0.48 | 0.43 | 0.28-0.65 | 8.52E-05 | ^r^ |
| rs1918287 | 7q36.1 | 147938494 | G | 0.32 | 0.24 | 1.68 | 1.30-2.18 | 8.63E-05 |  |
| rs9976123 | 21q22.3 | 43129312 | T | 0.44 | 0.51 | 0.50 | 0.35-0.70 | 8.63E-05 | ^d^ |
| rs2418454 | 18q12.1 | 27717594 | C | 0.52 | 0.42 | 1.55 | 1.25-1.94 | 8.65E-05 |  |
| rs17687280 | 19p12 | 24339720 | A | 0.25 | 0.18 | 1.91 | 1.38-2.64 | 8.68E-05 | ^d^ |
| rs11642923 | 16q21 | 64463355 | T | 0.13 | 0.06 | 2.17 | 1.47-3.20 | 8.86E-05 |  |
| rs3105773 | 8q24.11 | 118703810 | T | 0.12 | 0.06 | 2.37 | 1.54-3.66 | 9.02E-05 | ^d^ |
| rs1327665 | 6p22.3 | 20210374 | A | 0.56 | 0.48 | 2.01 | 1.42-2.86 | 9.04E-05 | ^r^ |
| rs274667 | 5p15.31 | 6710771 | A | 0.32 | 0.35 | 0.36 | 0.21-0.60 | 9.07E-05 | ^r^ |
| rs1182673 | 9p22.1 | 19751588 | G | 0.35 | 0.27 | 1.88 | 1.37-2.57 | 9.08E-05 | ^d^ |
| rs7097549 | 10p14 | 11873116 | A | 0.19 | 0.28 | 0.59 | 0.45-0.77 | 9.12E-05 |  |
| rs1406604 | 7q36.1 | 147939341 | A | 0.32 | 0.24 | 1.68 | 1.29-2.18 | 9.40E-05 |  |
| rs2960023 | 18q12.1 | 27782015 | C | 0.51 | 0.41 | 1.55 | 1.25-1.94 | 9.40E-05 |  |
| rs954640 | 18q12.1 | 27688654 | T | 0.52 | 0.42 | 1.55 | 1.24-1.93 | 9.42E-05 |  |
| rs919659 | 8q24.21 | 129686633 | T | 0.22 | 0.14 | 2.00 | 1.41-2.82 | 9.49E-05 | ^d^ |
| rs4535723 | 8q24.22 | 132230281 | A | 0.33 | 0.44 | 0.64 | 0.51-0.80 | 9.57E-05 |  |
| rs6924804 | 6p12.1 | 56195334 | A | 0.32 | 0.39 | 0.36 | 0.22-0.61 | 9.64E-05 | ^r^ |
| rs9933242 | 16q12.1 | 52495720 | C | 0.50 | 0.44 | 2.12 | 1.45-3.09 | 9.64E-05 | ^r^ |
| rs11648590 | 16q21 | 64459622 | T | 0.13 | 0.07 | 2.15 | 1.46-3.16 | 9.69E-05 |  |
| rs4381631 | 17q24.2 | 64766443 | G | 0.40 | 0.51 | 0.51 | 0.36-0.72 | 9.72E-05 | ^d^ |
| rs1609480 | 3p25.3 | 10901959 | C | 0.51 | 0.60 | 0.51 | 0.36-0.71 | 9.96E-05 | ^r^ |
| rs11086401 | 20q13.2 | 51328785 | T | 0.42 | 0.51 | 0.46 | 0.31-0.68 | 1.01E-04 | ^r^ |
| rs3096420 | 16p12.1 | 26243264 | G | 0.46 | 0.52 | 0.47 | 0.32-0.69 | 1.03E-04 | ^r^ |
| rs1229743 | 5q31.2 | 136710961 | T | 0.29 | 0.35 | 0.54 | 0.39-0.73 | 1.04E-04 | ^d^ |
| rs2785990 | 1q41 | 219687432 | C | 0.28 | 0.37 | 0.63 | 0.50-0.79 | 1.04E-04 |  |
| rs4736753 | 8q24.22 | 132225548 | C | 0.58 | 0.47 | 1.56 | 1.25-1.96 | 1.06E-04 |  |
| rs17560394 | 16q21 | 64451377 | A | 0.13 | 0.07 | 2.13 | 1.45-3.12 | 1.07E-04 |  |
| rs4726933 | 7q36.1 | 147937132 | A | 0.31 | 0.24 | 1.67 | 1.29-2.17 | 1.07E-04 |  |
| rs11124140 | 2q12.2 | 107485371 | C | 0.78 | 0.73 | 1.87 | 1.36-2.58 | 1.07E-04 | ^r^ |
| rs7766123 | 6q12 | 69062963 | T | 0.25 | 0.32 | 0.54 | 0.39-0.74 | 1.08E-04 | ^d^ |
| rs2949536 | 18q12.1 | 27790205 | C | 0.51 | 0.41 | 1.54 | 1.24-1.92 | 1.08E-04 |  |
| rs265071 | 4q22.3 | 96401017 | C | 0.55 | 0.49 | 2.11 | 1.45-3.09 | 1.08E-04 | ^d^ |
| rs17561386 | 16q21 | 64479769 | A | 0.13 | 0.06 | 2.15 | 1.46-3.17 | 1.09E-04 |  |
| rs2915767 | 10q25.3 | 118345726 | T | 0.12 | 0.06 | 2.22 | 1.48-3.32 | 1.11E-04 |  |
| rs6425457 | 1q25.2 | 177937441 | A | 0.65 | 0.57 | 1.90 | 1.37-2.63 | 1.11E-04 | ^r^ |
| rs1483597 | 4q28.1 | 125782537 | T | 0.15 | 0.23 | 0.57 | 0.43-0.76 | 1.11E-04 |  |
| rs602497 | 18q12.3 | 40992565 | G | 0.37 | 0.28 | 1.86 | 1.36-2.54 | 1.12E-04 | ^d^ |
| rs499974 | 11q13.5 | 75455021 | T | 0.14 | 0.20 | 0.55 | 0.40-0.74 | 1.13E-04 |  |
| rs41441449 | 16q21 | 64463085 | A | 0.12 | 0.06 | 2.19 | 1.47-3.25 | 1.13E-04 |  |
| rs1245807 | 12q21.2 | 79636158 | C | 0.34 | 0.28 | 1.86 | 1.36-2.55 | 1.13E-04 | ^d^ |
| rs17563153 | 16q21 | 64512167 | C | 0.13 | 0.07 | 2.14 | 1.45-3.15 | 1.14E-04 |  |
| rs17642079 | 16q21 | 64485520 | C | 0.12 | 0.06 | 2.19 | 1.47-3.25 | 1.14E-04 |  |
| rs12458902 | 18q12.1 | 27720148 | C | 0.51 | 0.41 | 1.55 | 1.24-1.93 | 1.14E-04 |  |
| rs11624667 | 14q32.13 | 96047535 | G | 0.54 | 0.47 | 2.03 | 1.42-2.91 | 1.16E-04 | ^r^ |
| rs1579749 | 5q21.2 | 103715467 | C | 0.15 | 0.22 | 0.52 | 0.37-0.73 | 1.17E-04 | ^d^ |
| rs1524806 | 18q12.1 | 27710130 | T | 0.52 | 0.42 | 1.54 | 1.24-1.92 | 1.17E-04 |  |
| rs2915748 | 10q25.3 | 118313265 | T | 0.12 | 0.06 | 2.19 | 1.47-3.27 | 1.18E-04 |  |
| rs1526968 | 12q21.2 | 79585008 | C | 0.34 | 0.28 | 1.86 | 1.36-2.55 | 1.19E-04 | ^d^ |
| rs171752 | 5q21.3 | 107066192 | C | 0.43 | 0.52 | 0.50 | 0.35-0.71 | 1.19E-04 | ^d^ |
| rs1437950 | 2q23.3 | 150814389 | C | 0.29 | 0.36 | 0.62 | 0.49-0.79 | 1.20E-04 |  |
| rs769122 | 20p11.23 | 19708351 | C | 0.19 | 0.25 | 0.53 | 0.38-0.73 | 1.21E-04 | ^d^ |
| rs12591347 | 15q26.1 | 90175251 | A | 0.35 | 0.40 | 0.38 | 0.23-0.62 | 1.21E-04 | ^r^ |
| rs3737741 | 1p33 | 47000566 | T | 0.53 | 0.58 | 0.46 | 0.31-0.68 | 1.22E-04 | ^d^ |
| rs6689437 | 1q43 | 242708729 | G | 0.15 | 0.09 | 2.10 | 1.44-3.07 | 1.23E-04 | ^d^ |
| rs903269 | 10p11.23 | 31040517 | T | 0.33 | 0.38 | 0.37 | 0.23-0.62 | 1.23E-04 | ^r^ |
| rs2537828 | 17q24.2 | 65643086 | T | 0.24 | 0.16 | 1.73 | 1.31-2.29 | 1.23E-04 |  |
| rs2289669 | 17p11.2 | 19463343 | A | 0.49 | 0.38 | 1.55 | 1.24-1.95 | 1.24E-04 |  |
| rs9325467 | 10q23.33 | 95991336 | T | 0.37 | 0.42 | 0.43 | 0.28-0.66 | 1.24E-04 | ^r^ |
| rs17561749 | 16q21 | 64484861 | T | 0.13 | 0.06 | 2.14 | 1.45-3.16 | 1.24E-04 |  |
| rs17561853 | 16q21 | 64487405 | G | 0.13 | 0.06 | 2.14 | 1.45-3.16 | 1.24E-04 |  |
| rs13225790 | 7q21.11 | 77609140 | A | 0.47 | 0.54 | 0.50 | 0.35-0.71 | 1.25E-04 | ^r^ |
| rs4726935 | 7q36.1 | 147937412 | A | 0.31 | 0.24 | 1.66 | 1.28-2.16 | 1.28E-04 |  |
| rs1316990 | 4q31.3 | 154995958 | A | 0.24 | 0.18 | 1.71 | 1.30-2.26 | 1.29E-04 |  |
| rs28741 | 2p25.1 | 8089351 | T | 0.10 | 0.15 | 0.48 | 0.33-0.70 | 1.30E-04 | ^d^ |
| rs4325274 | 11p15.1 | 16623906 | G | 0.35 | 0.26 | 1.59 | 1.25-2.03 | 1.34E-04 |  |
| rs11011920 | 10p12.31 | 20554139 | A | 0.27 | 0.20 | 1.70 | 1.29-2.23 | 1.35E-04 |  |
| rs11248868 | 16p13.3 | 1373593 | C | 0.11 | 0.17 | 0.49 | 0.34-0.70 | 1.36E-04 | ^d^ |
| rs10853816 | 19q12 | 30068177 | A | 0.39 | 0.45 | 0.44 | 0.29-0.67 | 1.37E-04 | ^r^ |
| rs10213695 | 5q21.3 | 108674964 | C | 0.17 | 0.10 | 1.90 | 1.37-2.65 | 1.37E-04 |  |
| rs11187825 | 10q23.33 | 95987313 | C | 0.37 | 0.42 | 0.43 | 0.28-0.66 | 1.37E-04 | ^r^ |
| rs667547 | 20p12.2 | 10468262 | C | 0.41 | 0.47 | 0.52 | 0.37-0.73 | 1.39E-04 | ^d^ |
| rs1826814 | 12q21.32 | 88037493 | T | 0.53 | 0.64 | 0.65 | 0.52-0.81 | 1.39E-04 |  |
| rs2141701 | 12q21.2 | 79662833 | A | 0.35 | 0.29 | 1.85 | 1.35-2.54 | 1.41E-04 | ^d^ |
| rs10781409 | 9q21.2 | 79661721 | T | 0.29 | 0.24 | 1.84 | 1.35-2.53 | 1.41E-04 | ^d^ |
| rs12077521 | 1p35.2 | 30285915 | A | 0.38 | 0.48 | 0.65 | 0.52-0.81 | 1.41E-04 |  |
| rs4866870 | 5p12 | 44150422 | A | 0.08 | 0.13 | 0.46 | 0.31-0.68 | 1.42E-04 | ^d^ |
| rs17642194 | 16q21 | 64487430 | G | 0.13 | 0.07 | 2.12 | 1.44-3.12 | 1.44E-04 |  |
| rs7242121 | 18q12.1 | 27641481 | C | 0.55 | 0.45 | 1.50 | 1.22-1.85 | 1.45E-04 |  |
| rs903270 | 10p11.23 | 31040363 | T | 0.33 | 0.37 | 0.37 | 0.22-0.62 | 1.45E-04 | ^r^ |
| rs1406603 | 7q36.1 | 147939285 | T | 0.36 | 0.28 | 1.62 | 1.26-2.07 | 1.45E-04 |  |
| rs2833890 | 21q22.11 | 33927886 | A | 0.27 | 0.20 | 1.68 | 1.29-2.20 | 1.48E-04 |  |
| rs975795 | 16q21 | 63094682 | A | 0.54 | 0.47 | 2.07 | 1.42-3.02 | 1.48E-04 | ^d^ |
| rs4954962 | 2q22.1 | 138788075 | A | 0.38 | 0.46 | 0.53 | 0.38-0.73 | 1.49E-04 | ^d^ |
| rs11646730 | 16q21 | 64469164 | G | 0.13 | 0.07 | 2.11 | 1.44-3.11 | 1.49E-04 |  |
| rs17560727 | 16q21 | 64460864 | T | 0.12 | 0.06 | 2.12 | 1.44-3.13 | 1.49E-04 |  |
| rs4676960 | 3p13 | 73928049 | G | 0.05 | 0.11 | 0.42 | 0.27-0.66 | 1.49E-04 |  |
| rs2377686 | 2q12.2 | 107463331 | A | 0.77 | 0.72 | 1.85 | 1.34-2.54 | 1.50E-04 | ^r^ |
| rs3105766 | 8q24.11 | 118698804 | C | 0.11 | 0.06 | 2.32 | 1.50-3.60 | 1.51E-04 | ^d^ |
| rs2241377 | 19p13.11 | 16957638 | C | 0.52 | 0.63 | 0.65 | 0.52-0.81 | 1.51E-04 |  |
| rs17643139 | 16q21 | 64510608 | T | 0.13 | 0.07 | 2.11 | 1.43-3.11 | 1.54E-04 |  |
| rs17643212 | 16q21 | 64511272 | A | 0.13 | 0.07 | 2.11 | 1.43-3.11 | 1.54E-04 |  |
| rs17341203 | 1p35.2 | 30304090 | G | 0.38 | 0.48 | 0.65 | 0.52-0.81 | 1.55E-04 |  |
| rs1245837 | 12q21.2 | 79652280 | C | 0.35 | 0.29 | 1.84 | 1.34-2.53 | 1.55E-04 | ^d^ |
| rs10828003 | 10p12.31 | 20580125 | C | 0.28 | 0.21 | 1.66 | 1.28-2.17 | 1.56E-04 |  |
| rs2287144 | 16q12.1 | 52502318 | T | 0.49 | 0.44 | 2.08 | 1.42-3.03 | 1.56E-04 | ^r^ |
| rs12297321 | 12q13.11 | 47109387 | T | 0.10 | 0.18 | 0.50 | 0.35-0.72 | 1.56E-04 | ^d^ |
| rs16981268 | 20p11.23 | 19860352 | A | 0.12 | 0.06 | 2.17 | 1.45-3.23 | 1.57E-04 |  |
| rs4862780 | 4q34.3 | 180109258 | C | 0.70 | 0.79 | 0.62 | 0.48-0.79 | 1.58E-04 |  |
| rs1232602 | 20p12.2 | 10598076 | T | 0.31 | 0.38 | 0.64 | 0.51-0.81 | 1.59E-04 |  |
| rs1428931 | 5q21.3 | 108685676 | C | 0.18 | 0.11 | 1.83 | 1.34-2.51 | 1.59E-04 |  |
| rs2232109 | 7p13 | 43916491 | T | 0.10 | 0.15 | 0.48 | 0.33-0.70 | 1.60E-04 | ^d^ |
| rs2391322 | 1p22.1 | 94343023 | C | 0.44 | 0.35 | 1.55 | 1.24-1.95 | 1.61E-04 |  |
| rs868260 | 5q21.3 | 108654729 | A | 0.18 | 0.11 | 1.87 | 1.35-2.58 | 1.62E-04 |  |
| rs10032178 | 4q32.1 | 159136928 | A | 0.33 | 0.23 | 1.61 | 1.26-2.07 | 1.62E-04 |  |
| rs11642838 | 16q21 | 64454026 | G | 0.13 | 0.07 | 2.09 | 1.43-3.07 | 1.63E-04 |  |
| rs1354741 | 4q28.1 | 125788061 | A | 0.09 | 0.16 | 0.48 | 0.33-0.71 | 1.65E-04 | ^d^ |
| rs2681942 | 10p12.31 | 20561539 | T | 0.49 | 0.59 | 0.65 | 0.52-0.82 | 1.65E-04 |  |
| rs2833889 | 21q22.11 | 33927768 | G | 0.27 | 0.20 | 1.68 | 1.28-2.19 | 1.65E-04 |  |
| rs11679046 | 2q37.1 | 234258101 | T | 0.38 | 0.36 | 2.49 | 1.55-3.99 | 1.66E-04 | ^r^ |
| rs2367703 | 5p13.1 | 38856268 | T | 0.33 | 0.24 | 1.59 | 1.25-2.03 | 1.67E-04 |  |
| rs12138736 | 1p33 | 46999758 | T | 0.53 | 0.58 | 0.47 | 0.31-0.69 | 1.68E-04 | ^d^ |
| rs6425603 | 1q25.2 | 180018254 | T | 0.38 | 0.36 | 2.53 | 1.56-4.09 | 1.68E-04 | ^r^ |
| rs6821381 | 4q28.2 | 129438919 | G | 0.20 | 0.28 | 0.61 | 0.48-0.79 | 1.68E-04 |  |
| rs4762397 | 12q23.1 | 98052667 | G | 0.47 | 0.56 | 0.64 | 0.51-0.81 | 1.69E-04 |  |
| rs11113673 | 12q21.2 | 79708851 | C | 0.34 | 0.28 | 1.83 | 1.34-2.51 | 1.71E-04 | ^d^ |
| rs2681951 | 10p12.31 | 20546337 | C | 0.51 | 0.60 | 0.66 | 0.53-0.82 | 1.71E-04 |  |
| rs13276595 | 8p21.3 | 21283421 | T | 0.36 | 0.28 | 1.58 | 1.24-2.00 | 1.72E-04 |  |
| rs12679479 | 8p21.3 | 21282361 | T | 0.37 | 0.28 | 1.60 | 1.25-2.05 | 1.72E-04 |  |
| rs8045897 | 16q12.1 | 52509376 | A | 0.50 | 0.44 | 2.06 | 1.41-3.00 | 1.74E-04 | ^r^ |

Direction: RA is reference allele. The odds ratio (OR) is presented for the reference allele, compared with the non-reference allele, for a given model.

**Supplemental Table S5d.**

**Top 200 associations from the Discovery GWAS – Mexican Ancestry**

| **SNP** | **Cytoband** | **Position** | **RA** | **Case  RAF** | **Control RAF** | **OR** | **95% CI** | **P-value** | |
| --- | --- | --- | --- | --- | --- | --- | --- | --- | --- |
| rs7975752 | 12q24.21 | 116154654 | G | 0.31 | 0.25 | 1.76 | 1.39-2.21 | 1.67E-06 | ^d^ |
| rs10507265 | 12q24.21 | 116171381 | G | 0.30 | 0.24 | 1.76 | 1.39-2.22 | 1.96E-06 | ^d^ |
| rs731565 | 7q35 | 147406262 | T | 0.28 | 0.20 | 1.58 | 1.30-1.92 | 4.06E-06 |  |
| rs7779787 | 7q35 | 147368736 | A | 0.27 | 0.20 | 1.58 | 1.30-1.93 | 4.62E-06 |  |
| rs2168858 | 17p13.1 | 9387453 | G | 0.34 | 0.42 | 0.68 | 0.58-0.80 | 5.27E-06 |  |
| rs1424609 | 12q24.21 | 116163432 | G | 0.27 | 0.21 | 1.72 | 1.36-2.18 | 5.39E-06 | ^d^ |
| rs728571 | 16q12.2 | 55214093 | A | 0.32 | 0.39 | 0.67 | 0.56-0.80 | 5.70E-06 |  |
| rs4849965 | 2p25.2 | 4608927 | C | 0.36 | 0.28 | 1.50 | 1.26-1.79 | 6.18E-06 |  |
| rs6542610 | 2p25.2 | 4630194 | T | 0.38 | 0.30 | 1.49 | 1.26-1.78 | 6.23E-06 |  |
| rs10850556 | 12q24.21 | 116155853 | G | 0.27 | 0.22 | 1.71 | 1.35-2.16 | 6.77E-06 | ^d^ |
| rs6910061 | 6p24.2 | 11101918 | A | 0.17 | 0.11 | 1.74 | 1.36-2.22 | 8.55E-06 |  |
| rs1353202 | 6q22.31 | 125724359 | C | 0.10 | 0.15 | 0.54 | 0.42-0.71 | 1.03E-05 | ^d^ |
| rs17210536 | 8q24.21 | 131217790 | C | 0.10 | 0.06 | 2.10 | 1.51-2.92 | 1.08E-05 | ^d^ |
| rs5027679 | 6p24.2 | 11118871 | A | 0.17 | 0.11 | 1.74 | 1.36-2.23 | 1.16E-05 |  |
| rs6994403 | 8q24.13 | 125754231 | C | 0.38 | 0.45 | 0.59 | 0.46-0.75 | 1.33E-05 | ^d^ |
| rs13152588 | 4p14 | 37365976 | G | 0.57 | 0.65 | 0.59 | 0.47-0.75 | 1.41E-05 | ^r^ |
| rs974451 | 8q24.21 | 127708294 | C | 0.24 | 0.18 | 1.57 | 1.28-1.92 | 1.49E-05 |  |
| rs1491184 | 15q26.3 | 98536596 | T | 0.28 | 0.36 | 0.61 | 0.48-0.76 | 1.61E-05 | ^d^ |
| rs12501671 | 4q35.2 | 188030324 | C | 0.17 | 0.11 | 1.70 | 1.33-2.16 | 1.61E-05 |  |
| rs2581651 | 18q22.3 | 72926042 | G | 0.16 | 0.12 | 1.78 | 1.37-2.32 | 1.63E-05 | ^d^ |
| rs2144567 | 20p12.2 | 10122212 | T | 0.10 | 0.15 | 0.58 | 0.45-0.74 | 1.69E-05 |  |
| rs9845819 | 3p14.1 | 65501006 | C | 0.27 | 0.33 | 0.61 | 0.48-0.76 | 1.81E-05 | ^d^ |
| rs6993864 | 8q24.13 | 125754151 | G | 0.38 | 0.45 | 0.59 | 0.46-0.75 | 1.84E-05 | ^d^ |
| rs17675302 | 17q21.31 | 41895934 | A | 0.20 | 0.26 | 0.60 | 0.48-0.76 | 2.00E-05 | ^d^ |
| rs6923486 | 6q25.1 | 151392209 | A | 0.13 | 0.18 | 0.58 | 0.45-0.74 | 2.18E-05 | ^d^ |
| rs9533573 | 13q14.11 | 44235707 | C | 0.33 | 0.39 | 0.60 | 0.48-0.76 | 2.28E-05 | ^d^ |
| rs7188661 | 16p11.2 | 29287978 | C | 0.37 | 0.33 | 2.20 | 1.53-3.16 | 2.28E-05 | ^r^ |
| rs3851775 | 16p11.2 | 29279750 | G | 0.38 | 0.34 | 2.15 | 1.51-3.08 | 2.46E-05 | ^r^ |
| rs1949281 | 12q12 | 43585475 | A | 0.16 | 0.11 | 1.67 | 1.32-2.12 | 2.53E-05 |  |
| rs3737882 | 1q32.1 | 203034955 | C | 0.13 | 0.19 | 0.62 | 0.50-0.78 | 2.61E-05 |  |
| rs6918690 | 6q25.1 | 151395408 | G | 0.13 | 0.17 | 0.58 | 0.45-0.75 | 2.62E-05 | ^d^ |
| rs11150714 | 16p11.2 | 29283704 | T | 0.37 | 0.32 | 2.18 | 1.51-3.13 | 2.87E-05 | ^r^ |
| rs6043668 | 20p12.1 | 15989936 | C | 0.10 | 0.05 | 1.99 | 1.44-2.75 | 2.90E-05 |  |
| rs678395 | 10p15.1 | 6221318 | G | 0.07 | 0.12 | 0.55 | 0.42-0.73 | 2.96E-05 |  |
| rs1542790 | 6q22.31 | 125728608 | T | 0.10 | 0.15 | 0.56 | 0.43-0.74 | 2.97E-05 | ^d^ |
| rs1958 | 4p14 | 37360817 | A | 0.57 | 0.64 | 0.60 | 0.48-0.77 | 3.05E-05 | ^r^ |
| rs17170638 | 7q35 | 147369307 | G | 0.24 | 0.18 | 1.55 | 1.26-1.90 | 3.07E-05 |  |
| rs12598144 | 16p12.1 | 24310882 | T | 0.49 | 0.40 | 1.40 | 1.19-1.64 | 3.16E-05 |  |
| rs1479100 | 8q21.11 | 76083241 | A | 0.53 | 0.47 | 1.77 | 1.35-2.31 | 3.29E-05 | ^r^ |
| rs1486400 | 12q24.21 | 116026433 | G | 0.44 | 0.37 | 1.42 | 1.20-1.67 | 3.43E-05 |  |
| rs2917821 | 15q22.2 | 60144421 | C | 0.13 | 0.18 | 0.59 | 0.46-0.76 | 3.53E-05 | ^d^ |
| rs9995329 | 4q35.2 | 188032207 | G | 0.16 | 0.11 | 1.67 | 1.31-2.13 | 3.59E-05 |  |
| rs9998261 | 4q35.2 | 188023979 | A | 0.17 | 0.11 | 1.73 | 1.33-2.24 | 3.62E-05 | ^d^ |
| rs2639993 | 18q22.3 | 72926078 | A | 0.17 | 0.12 | 1.73 | 1.33-2.24 | 3.64E-05 | ^d^ |
| rs17517821 | 20p12.2 | 10133139 | C | 0.14 | 0.19 | 0.59 | 0.46-0.76 | 3.70E-05 | ^d^ |
| rs13329884 | 16p11.2 | 29281054 | C | 0.38 | 0.35 | 2.09 | 1.47-2.96 | 3.70E-05 | ^r^ |
| rs1146971 | 13q31.1 | 81908615 | C | 0.38 | 0.43 | 0.60 | 0.47-0.77 | 3.77E-05 | ^d^ |
| rs1520175 | 12q24.21 | 116160540 | C | 0.28 | 0.22 | 1.63 | 1.29-2.06 | 3.78E-05 | ^d^ |
| rs11132426 | 4q35.2 | 188041427 | G | 0.17 | 0.11 | 1.66 | 1.30-2.11 | 3.90E-05 |  |
| rs2327335 | 6p24.2 | 11119047 | A | 0.17 | 0.11 | 1.65 | 1.30-2.10 | 3.96E-05 |  |
| rs2372682 | 2q35 | 216874573 | C | 0.18 | 0.13 | 1.71 | 1.32-2.21 | 4.17E-05 | ^d^ |
| rs9375394 | 6q22.31 | 125729973 | T | 0.11 | 0.15 | 0.58 | 0.44-0.75 | 4.19E-05 | ^d^ |
| rs16953500 | 16q12.2 | 54442878 | G | 0.15 | 0.10 | 1.67 | 1.31-2.13 | 4.19E-05 |  |
| rs4932221 | 15q26.1 | 89967247 | C | 0.26 | 0.20 | 1.63 | 1.29-2.06 | 4.29E-05 | ^d^ |
| rs10858154 | 9q34.3 | 138608747 | A | 0.11 | 0.16 | 0.61 | 0.48-0.77 | 4.32E-05 |  |
| rs17019490 | 2p25.2 | 4653262 | C | 0.34 | 0.27 | 1.47 | 1.22-1.76 | 4.32E-05 |  |
| rs7130522 | 11q21 | 95710493 | A | 0.47 | 0.42 | 1.69 | 1.31-2.17 | 4.37E-05 | ^d^ |
| rs1016913 | 7p15.2 | 26053267 | C | 0.11 | 0.16 | 0.57 | 0.44-0.75 | 4.43E-05 | ^d^ |
| rs8038207 | 15q25.3 | 86316570 | A | 0.26 | 0.19 | 1.49 | 1.23-1.80 | 4.90E-05 |  |
| rs13333140 | 16q12.2 | 54443086 | C | 0.15 | 0.10 | 1.66 | 1.30-2.12 | 4.94E-05 |  |
| rs1605939 | 8q21.11 | 76070707 | A | 0.53 | 0.47 | 1.77 | 1.34-2.34 | 5.05E-05 | ^r^ |
| rs16851279 | 3q13.11 | 105266274 | G | 0.31 | 0.25 | 1.46 | 1.21-1.75 | 5.13E-05 |  |
| rs830406 | 9p13.3 | 33217212 | G | 0.39 | 0.45 | 0.60 | 0.47-0.77 | 5.14E-05 | ^d^ |
| rs902625 | 15q26.3 | 98537979 | T | 0.24 | 0.31 | 0.63 | 0.50-0.78 | 5.16E-05 | ^d^ |
| rs4609646 | 12q24.21 | 116079028 | C | 0.32 | 0.39 | 0.70 | 0.59-0.83 | 5.17E-05 |  |
| rs6736573 | 2p12 | 80007503 | C | 0.25 | 0.30 | 0.63 | 0.50-0.79 | 5.35E-05 | ^d^ |
| rs4932130 | 15q26.1 | 89966923 | G | 0.25 | 0.20 | 1.62 | 1.28-2.04 | 5.46E-05 | ^d^ |
| rs4841989 | 9q34.3 | 138381748 | A | 0.11 | 0.16 | 0.58 | 0.44-0.76 | 5.47E-05 | ^d^ |
| rs11128090 | 3p14.1 | 68804021 | T | 0.12 | 0.08 | 1.79 | 1.35-2.38 | 5.54E-05 |  |
| rs9895062 | 17p13.1 | 9370623 | G | 0.36 | 0.44 | 0.71 | 0.60-0.84 | 5.70E-05 |  |
| rs7895470 | 10p12.31 | 20235463 | C | 0.49 | 0.44 | 1.78 | 1.34-2.36 | 5.83E-05 | ^r^ |
| rs246755 | 5q31.1 | 131386278 | T | 0.55 | 0.46 | 1.40 | 1.19-1.66 | 5.85E-05 |  |
| rs1112403 | 3p14.1 | 65454372 | A | 0.30 | 0.35 | 0.63 | 0.50-0.79 | 5.91E-05 | ^d^ |
| rs11130054 | 3p21.31 | 45367939 | A | 0.47 | 0.52 | 0.58 | 0.45-0.76 | 6.00E-05 | ^d^ |
| rs17531431 | 17q21.31 | 41803893 | T | 0.18 | 0.23 | 0.62 | 0.49-0.78 | 6.10E-05 | ^d^ |
| rs1494152 | 6q22.31 | 125728865 | A | 0.10 | 0.15 | 0.58 | 0.44-0.76 | 6.21E-05 | ^d^ |
| rs7761853 | 6p22.3 | 22773338 | T | 0.11 | 0.17 | 0.62 | 0.49-0.78 | 6.41E-05 |  |
| rs851734 | 7q35 | 147362105 | G | 0.24 | 0.18 | 1.52 | 1.24-1.86 | 6.54E-05 |  |
| rs1574131 | 4p16.1 | 8041800 | A | 0.41 | 0.35 | 1.43 | 1.20-1.70 | 6.56E-05 |  |
| rs135368 | 22q13.31 | 47574009 | C | 0.25 | 0.20 | 1.47 | 1.22-1.78 | 6.58E-05 |  |
| rs2077368 | 15q26.1 | 89970765 | G | 0.25 | 0.19 | 1.61 | 1.27-2.04 | 6.70E-05 | ^d^ |
| rs12544686 | 8q21.11 | 76218074 | T | 0.51 | 0.45 | 1.74 | 1.33-2.29 | 6.77E-05 | ^r^ |
| rs2581652 | 18q22.3 | 72926545 | A | 0.16 | 0.12 | 1.70 | 1.31-2.21 | 6.87E-05 | ^d^ |
| rs41514147 | 4p16.1 | 8043682 | C | 0.41 | 0.35 | 1.42 | 1.20-1.69 | 6.91E-05 |  |
| rs26146 | 5p15.2 | 11478432 | C | 0.30 | 0.37 | 0.70 | 0.58-0.83 | 7.01E-05 |  |
| rs10031016 | 4q35.2 | 188023912 | T | 0.17 | 0.11 | 1.69 | 1.31-2.19 | 7.05E-05 | ^d^ |
| rs6906360 | 6p24.2 | 11118427 | T | 0.15 | 0.10 | 1.66 | 1.29-2.14 | 7.16E-05 |  |
| rs10744862 | 12q24.21 | 116144391 | T | 0.22 | 0.17 | 1.64 | 1.28-2.09 | 7.18E-05 | ^d^ |
| rs4808923 | 19p13.11 | 19232824 | A | 0.20 | 0.14 | 1.65 | 1.29-2.12 | 7.30E-05 | ^d^ |
| rs17605241 | 14q21.1 | 41564109 | G | 0.31 | 0.26 | 1.45 | 1.21-1.75 | 7.41E-05 |  |
| rs317906 | 4p15.32 | 16140605 | T | 0.42 | 0.49 | 0.72 | 0.61-0.85 | 7.46E-05 |  |
| rs11889150 | 2q37.3 | 239324542 | G | 0.12 | 0.18 | 0.60 | 0.46-0.77 | 7.55E-05 | ^d^ |
| rs7762961 | 6p24.2 | 11109669 | A | 0.15 | 0.10 | 1.74 | 1.32-2.28 | 7.60E-05 | ^d^ |
| rs17202203 | 7p14.1 | 37466564 | G | 0.23 | 0.29 | 0.69 | 0.58-0.83 | 7.65E-05 |  |
| rs4323164 | 4q35.2 | 187996662 | T | 0.20 | 0.14 | 1.64 | 1.29-2.10 | 7.69E-05 | ^d^ |
| rs4965895 | 15q26.3 | 98911342 | C | 0.53 | 0.48 | 1.69 | 1.30-2.20 | 7.73E-05 | ^d^ |
| rs6567288 | 18q21.33 | 60218334 | C | 0.44 | 0.37 | 1.61 | 1.27-2.04 | 7.85E-05 | ^d^ |
| rs1190100 | 14q22.3 | 58015648 | G | 0.12 | 0.18 | 0.64 | 0.51-0.80 | 7.89E-05 |  |
| rs3787862 | 21q22.13 | 39252701 | A | 0.11 | 0.15 | 0.58 | 0.45-0.76 | 7.93E-05 | ^d^ |
| rs4862772 | 4q35.2 | 188029191 | T | 0.16 | 0.11 | 1.64 | 1.28-2.09 | 8.00E-05 |  |
| rs1941101 | 18p11.21 | 13987707 | G | 0.14 | 0.19 | 0.65 | 0.52-0.80 | 8.09E-05 |  |
| rs1146975 | 13q31.1 | 81910279 | T | 0.39 | 0.44 | 0.62 | 0.48-0.78 | 8.09E-05 | ^d^ |
| rs7782999 | 7p14.1 | 37446693 | A | 0.42 | 0.36 | 1.61 | 1.27-2.04 | 8.10E-05 | ^d^ |
| rs2291377 | 3q13.11 | 105219699 | A | 0.32 | 0.25 | 1.44 | 1.20-1.73 | 8.30E-05 |  |
| rs6445482 | 3p14.1 | 65482662 | T | 0.29 | 0.34 | 0.63 | 0.50-0.79 | 8.34E-05 | ^d^ |
| rs10226770 | 7p21.3 | 12591794 | G | 0.38 | 0.31 | 1.40 | 1.18-1.65 | 8.46E-05 |  |
| rs2196918 | 2q24.3 | 164451683 | T | 0.06 | 0.10 | 0.54 | 0.40-0.74 | 8.55E-05 |  |
| rs470036 | 22q13.31 | 47572997 | T | 0.24 | 0.19 | 1.47 | 1.21-1.78 | 8.55E-05 |  |
| rs9644619 | 8p22 | 18987347 | G | 0.26 | 0.30 | 0.40 | 0.26-0.63 | 8.59E-05 | ^r^ |
| rs16953503 | 16q12.2 | 54443223 | A | 0.13 | 0.08 | 1.68 | 1.30-2.18 | 8.60E-05 |  |
| rs4283808 | 5q34 | 164370513 | C | 0.21 | 0.27 | 0.63 | 0.49-0.79 | 8.61E-05 | ^d^ |
| rs1041058 | 6q14.3 | 87698002 | A | 0.12 | 0.08 | 1.82 | 1.35-2.45 | 8.64E-05 | ^d^ |
| rs4317189 | 4p15.2 | 23495538 | T | 0.23 | 0.18 | 1.49 | 1.22-1.82 | 8.75E-05 |  |
| rs4641644 | 14q21.2 | 43803481 | T | 0.28 | 0.36 | 0.70 | 0.59-0.84 | 8.75E-05 |  |
| rs7613763 | 3q11.2 | 96056259 | C | 0.47 | 0.43 | 1.78 | 1.33-2.37 | 8.97E-05 | ^r^ |
| rs1436791 | 10q26.2 | 128325663 | T | 0.50 | 0.54 | 0.58 | 0.44-0.76 | 9.12E-05 | ^d^ |
| rs12623324 | 2q35 | 218016330 | C | 0.20 | 0.25 | 0.63 | 0.50-0.79 | 9.13E-05 | ^d^ |
| rs7920361 | 10p12.31 | 20237375 | C | 0.31 | 0.38 | 0.72 | 0.61-0.85 | 9.21E-05 |  |
| rs7454257 | 6q27 | 169212299 | G | 0.47 | 0.41 | 1.81 | 1.34-2.44 | 9.22E-05 | ^r^ |
| rs1946384 | 12q24.21 | 116080723 | C | 0.41 | 0.34 | 1.39 | 1.18-1.64 | 9.31E-05 |  |
| rs1999083 | 6q13 | 72450580 | A | 0.12 | 0.16 | 0.60 | 0.46-0.77 | 9.33E-05 | ^d^ |
| rs1205441 | 20q11.23 | 36876183 | A | 0.22 | 0.20 | 3.36 | 1.83-6.16 | 9.37E-05 | ^r^ |
| rs6836337 | 4q35.2 | 188015715 | A | 0.16 | 0.11 | 1.62 | 1.27-2.06 | 9.40E-05 |  |
| rs10494558 | 1q25.3 | 183147850 | G | 0.15 | 0.21 | 0.62 | 0.48-0.79 | 9.40E-05 | ^d^ |
| rs277190 | 12q12 | 43973663 | C | 0.22 | 0.25 | 0.63 | 0.50-0.80 | 9.68E-05 | ^d^ |
| rs1641001 | 16p13.2 | 8830022 | T | 0.35 | 0.33 | 2.13 | 1.45-3.11 | 9.80E-05 | ^r^ |
| rs9533534 | 13q14.11 | 44152727 | C | 0.48 | 0.42 | 1.38 | 1.18-1.63 | 9.87E-05 |  |
| rs2842158 | 10q26.2 | 128311780 | T | 0.35 | 0.32 | 2.17 | 1.47-3.21 | 9.92E-05 | ^r^ |
| rs8008487 | 14q21.1 | 41534471 | C | 0.37 | 0.31 | 1.41 | 1.19-1.68 | 9.96E-05 |  |
| rs679468 | 8q22.3 | 102595319 | T | 0.11 | 0.16 | 0.60 | 0.46-0.77 | 1.00E-04 | ^d^ |
| rs5756365 | 22q12.3 | 37228277 | A | 0.65 | 0.59 | 1.59 | 1.26-2.00 | 1.02E-04 | ^r^ |
| rs17019451 | 2p25.2 | 4624710 | C | 0.15 | 0.10 | 1.70 | 1.30-2.22 | 1.03E-04 | ^d^ |
| rs9906094 | 17p12 | 11616900 | A | 0.10 | 0.06 | 1.90 | 1.37-2.62 | 1.03E-04 | ^d^ |
| rs6582439 | 12q12 | 43592402 | G | 0.21 | 0.27 | 0.63 | 0.50-0.80 | 1.04E-04 | ^d^ |
| rs7314814 | 12q24.21 | 116109900 | C | 0.33 | 0.27 | 1.43 | 1.19-1.71 | 1.05E-04 |  |
| rs4618171 | 3p14.1 | 65442578 | T | 0.31 | 0.35 | 0.64 | 0.50-0.80 | 1.05E-04 | ^d^ |
| rs877226 | 2q31.3 | 182117625 | T | 0.19 | 0.26 | 0.67 | 0.55-0.82 | 1.06E-04 |  |
| rs2048793 | 18q21.33 | 60224279 | A | 0.39 | 0.33 | 1.58 | 1.25-1.99 | 1.07E-04 | ^d^ |
| rs12829889 | 12q21.31 | 81296813 | A | 0.12 | 0.08 | 1.80 | 1.34-2.43 | 1.08E-04 | ^d^ |
| rs2420929 | 10q26.13 | 123153176 | T | 0.25 | 0.30 | 0.69 | 0.58-0.84 | 1.08E-04 |  |
| rs7006947 | 8p23.1 | 9175706 | C | 0.33 | 0.27 | 1.42 | 1.19-1.69 | 1.08E-04 |  |
| rs7192938 | 16q23.1 | 77396290 | A | 0.06 | 0.11 | 0.54 | 0.40-0.74 | 1.09E-04 | ^d^ |
| rs1287505 | 13q31.1 | 81920601 | A | 0.38 | 0.44 | 0.62 | 0.49-0.79 | 1.10E-04 | ^d^ |
| rs9594975 | 13q14.11 | 44151908 | G | 0.46 | 0.39 | 1.38 | 1.17-1.62 | 1.12E-04 |  |
| rs8028722 | 15q26.3 | 98502566 | T | 0.27 | 0.34 | 0.70 | 0.58-0.84 | 1.14E-04 |  |
| rs1965297 | 12q24.21 | 116173802 | C | 0.31 | 0.26 | 1.58 | 1.25-1.99 | 1.15E-04 | ^d^ |
| rs10199699 | 2q22.3 | 146300691 | C | 0.23 | 0.17 | 1.59 | 1.26-2.01 | 1.16E-04 | ^d^ |
| rs204036 | 16p13.12 | 13336497 | T | 0.30 | 0.25 | 1.43 | 1.19-1.72 | 1.16E-04 |  |
| rs1526248 | 4q28.3 | 138591243 | C | 0.29 | 0.33 | 0.64 | 0.51-0.80 | 1.16E-04 | ^d^ |
| rs1459958 | 15q11.2 | 24098594 | C | 0.46 | 0.39 | 1.39 | 1.18-1.65 | 1.16E-04 |  |
| rs10410399 | 19p13.11 | 19206500 | C | 0.16 | 0.11 | 1.68 | 1.29-2.20 | 1.18E-04 | ^d^ |
| rs16958320 | 17p13.1 | 9376028 | G | 0.44 | 0.36 | 1.38 | 1.17-1.62 | 1.18E-04 |  |
| rs2291378 | 3q13.11 | 105219511 | G | 0.32 | 0.26 | 1.43 | 1.19-1.71 | 1.19E-04 |  |
| rs1377029 | 4q13.3 | 75733365 | G | 0.46 | 0.39 | 1.38 | 1.17-1.63 | 1.22E-04 |  |
| rs1377028 | 4q13.3 | 75738792 | G | 0.47 | 0.39 | 1.38 | 1.17-1.63 | 1.22E-04 |  |
| rs10758197 | 9p13.3 | 33208034 | A | 0.47 | 0.39 | 1.37 | 1.17-1.61 | 1.24E-04 |  |
| rs270377 | 6p24.3 | 7763018 | G | 0.42 | 0.46 | 0.62 | 0.48-0.79 | 1.24E-04 | ^d^ |
| rs373690 | 14q22.3 | 58021286 | A | 0.12 | 0.17 | 0.63 | 0.50-0.80 | 1.25E-04 |  |
| rs17076285 | 8p23.2 | 6083209 | T | 0.35 | 0.28 | 1.56 | 1.24-1.96 | 1.26E-04 | ^d^ |
| rs977266 | 4q13.3 | 75717601 | G | 0.51 | 0.58 | 0.73 | 0.62-0.86 | 1.26E-04 |  |
| rs10009459 | 4q32.1 | 158481615 | G | 0.09 | 0.13 | 0.58 | 0.44-0.77 | 1.28E-04 | ^d^ |
| rs646534 | 1p32.3 | 54734738 | A | 0.32 | 0.40 | 0.72 | 0.61-0.85 | 1.28E-04 |  |
| rs10950377 | 7p22.3 | 1835894 | C | 0.22 | 0.28 | 0.70 | 0.58-0.84 | 1.31E-04 |  |
| rs4530232 | 18q22.3 | 70622238 | A | 0.36 | 0.31 | 1.56 | 1.24-1.96 | 1.32E-04 | ^d^ |
| rs6553036 | 4q35.2 | 188019647 | A | 0.18 | 0.13 | 1.63 | 1.27-2.09 | 1.33E-04 | ^d^ |
| rs2325044 | 13q14.11 | 44145986 | A | 0.52 | 0.59 | 0.73 | 0.62-0.86 | 1.35E-04 |  |
| rs7923465 | 10p13 | 12761684 | A | 0.56 | 0.51 | 1.64 | 1.27-2.12 | 1.37E-04 | ^r^ |
| rs1610000 | 6q27 | 169194183 | C | 0.45 | 0.40 | 1.83 | 1.34-2.50 | 1.37E-04 | ^r^ |
| rs16894651 | 4p15.32 | 17208345 | T | 0.28 | 0.34 | 0.71 | 0.60-0.85 | 1.38E-04 |  |
| rs2715464 | 4q34.3 | 179555636 | A | 0.43 | 0.50 | 0.72 | 0.61-0.85 | 1.38E-04 |  |
| rs1318261 | 13q14.11 | 44147460 | C | 0.53 | 0.60 | 0.73 | 0.62-0.86 | 1.41E-04 |  |
| rs9467596 | 6p22.2 | 25783022 | G | 0.40 | 0.48 | 0.73 | 0.62-0.86 | 1.41E-04 |  |
| rs12485487 | 3p23 | 31541300 | A | 0.30 | 0.36 | 0.64 | 0.51-0.81 | 1.42E-04 | ^d^ |
| rs2714588 | 2p22.3 | 35675100 | A | 0.50 | 0.55 | 0.61 | 0.48-0.79 | 1.43E-04 | ^r^ |
| rs927984 | 6p22.2 | 25412987 | T | 0.11 | 0.07 | 1.74 | 1.31-2.33 | 1.45E-04 |  |
| rs1363759 | 19q13.32 | 47518884 | G | 0.46 | 0.38 | 1.37 | 1.16-1.61 | 1.46E-04 |  |
| rs1113762 | 4q35.1 | 183221707 | T | 0.07 | 0.10 | 0.58 | 0.43-0.77 | 1.46E-04 |  |
| rs11180647 | 12q21.2 | 76104713 | A | 0.34 | 0.28 | 1.55 | 1.24-1.95 | 1.46E-04 | ^d^ |
| rs4696737 | 4p16.1 | 8044224 | T | 0.23 | 0.18 | 1.58 | 1.25-2.00 | 1.47E-04 | ^d^ |
| rs12359272 | 10q24.1 | 97365163 | T | 0.41 | 0.46 | 0.62 | 0.48-0.79 | 1.48E-04 | ^d^ |
| rs567404 | 4p15.32 | 17201248 | G | 0.29 | 0.35 | 0.72 | 0.60-0.85 | 1.52E-04 |  |
| rs6751585 | 2p25.2 | 4589997 | T | 0.14 | 0.10 | 1.70 | 1.29-2.23 | 1.53E-04 | ^d^ |
| rs1299913 | 7p13 | 45215376 | A | 0.24 | 0.18 | 1.57 | 1.24-1.99 | 1.55E-04 | ^d^ |
| rs16903220 | 5q14.3 | 87820740 | A | 0.12 | 0.08 | 1.70 | 1.29-2.24 | 1.55E-04 |  |
| rs2309508 | 4q34.3 | 182590243 | A | 0.19 | 0.23 | 0.64 | 0.50-0.80 | 1.55E-04 | ^d^ |
| rs10462815 | 5p15.31 | 6415803 | C | 0.45 | 0.40 | 1.59 | 1.25-2.02 | 1.56E-04 | ^d^ |
| rs6751684 | 2p12 | 80023852 | A | 0.20 | 0.25 | 0.64 | 0.51-0.81 | 1.57E-04 | ^d^ |
| rs13292141 | 9p21.1 | 28602289 | A | 0.05 | 0.09 | 0.52 | 0.37-0.73 | 1.57E-04 | ^d^ |
| rs1552679 | 15q26.3 | 98501764 | A | 0.27 | 0.34 | 0.71 | 0.59-0.85 | 1.58E-04 |  |
| rs11688991 | 2p25.2 | 4621133 | T | 0.15 | 0.10 | 1.68 | 1.28-2.20 | 1.62E-04 | ^d^ |
| rs13142734 | 4p15.32 | 17200948 | G | 0.28 | 0.34 | 0.71 | 0.60-0.85 | 1.63E-04 |  |
| rs12913266 | 15q25.3 | 86294109 | A | 0.24 | 0.19 | 1.58 | 1.24-1.99 | 1.64E-04 | ^d^ |
| rs13424162 | 2p25.2 | 4608052 | T | 0.16 | 0.21 | 0.67 | 0.55-0.83 | 1.65E-04 |  |
| rs2425223 | 20q11.23 | 35001520 | A | 0.33 | 0.27 | 1.55 | 1.23-1.94 | 1.65E-04 | ^d^ |
| rs3760570 | 18q21.33 | 60239287 | G | 0.40 | 0.34 | 1.56 | 1.24-1.96 | 1.66E-04 | ^d^ |
| rs7613685 | 3p26.1 | 4185601 | C | 0.41 | 0.46 | 0.62 | 0.48-0.80 | 1.68E-04 | ^d^ |
| rs6841655 | 4p14 | 37358920 | T | 0.27 | 0.20 | 1.44 | 1.19-1.74 | 1.68E-04 |  |
| rs17019452 | 2p25.2 | 4625462 | A | 0.15 | 0.10 | 1.68 | 1.28-2.21 | 1.69E-04 | ^d^ |
| rs2744666 | 1q24.2 | 168737147 | C | 0.52 | 0.47 | 1.68 | 1.28-2.21 | 1.71E-04 | ^r^ |
| rs6854815 | 4p15.2 | 26466861 | T | 0.11 | 0.07 | 1.81 | 1.33-2.47 | 1.72E-04 | ^d^ |

Direction: RA is reference allele. The odds ratio (OR) is presented for the reference allele, compared with the non-reference allele, for a given model.
