# Supplementary material for: Genome-Wide Association and Trans-ethnic Meta-Analysis for Advanced Diabetic Kidney Disease: Family Investigation of Nephropathy and Diabetes (FIND)
Source: PLoS Genet. 2015 Aug 25;11(8):e1005352. doi: 10.1371/journal.pgen.1005352 (PMC4549309; doi:10.1371/journal.pgen.1005352)
Supplement: S6 Table — (DOCX) [file pgen.1005352.s007.docx]

**Supplemental Table S6a.**

**Top 200 associations from the FIND Replication – African Ancestry**

| **SNP** | **Cytoband** | **Position** | **RA** | **Case  RAF** | **Control RAF** | **OR** | **95% CI** | **P-value** | |
| --- | --- | --- | --- | --- | --- | --- | --- | --- | --- |
| rs2397972 | 15q26.2 | 96358652 | T | 0.17 | 0.12 | 1.55 | 1.30-1.85 | 1.35E-06 | ^d^ |
| rs9791642 | 7p21.1 | 16956379 | G | 0.12 | 0.08 | 1.59 | 1.30-1.94 | 6.19E-06 | ^d^ |
| rs13259109 | 8q23.3 | 113115897 | G | 0.11 | 0.10 | 1.46 | 1.21-1.76 | 6.72E-05 |  |
| rs1543380 | 20q13.2 | 50169212 | A | 0.57 | 0.62 | 0.71 | 0.60-0.84 | 8.00E-05 | ^r^ |
| rs6800003 | 3p24.3 | 16740524 | G | 0.15 | 0.12 | 1.39 | 1.18-1.63 | 8.59E-05 |  |
| rs11624667 | 14q32.13 | 96047535 | G | 0.16 | 0.14 | 1.42 | 1.18-1.70 | 1.44E-04 | ^d^ |
| rs10744862 | 12q24.21 | 116144391 | T | 0.08 | 0.07 | 1.56 | 1.24-1.97 | 1.48E-04 | ^d^ |
| rs6577427 | 1p36.23 | 7314231 | C | 0.36 | 0.39 | 0.80 | 0.71-0.90 | 2.24E-04 |  |
| rs1441541 | 5p15.33 | 3250352 | A | 0.50 | 0.68 | 0.46 | 0.30-0.70 | 3.10E-04 |  |
| rs10749550 | 10q23.1 | 82031197 | A | 0.07 | 0.06 | 1.57 | 1.22-2.00 | 3.47E-04 | ^d^ |
| rs1447125 | 11p12 | 41323240 | T | 0.47 | 0.43 | 1.37 | 1.15-1.64 | 3.86E-04 | ^d^ |
| rs4876279 | 8q23.3 | 113205771 | A | 0.16 | 0.14 | 1.31 | 1.12-1.54 | 6.96E-04 |  |
| rs590884 | 6q26 | 161378192 | T | 0.28 | 0.34 | 0.81 | 0.71-0.91 | 7.31E-04 |  |
| rs4599383 | 4p12 | 47545375 | C | 0.18 | 0.16 | 1.29 | 1.11-1.50 | 8.00E-04 |  |
| rs2252500 | 8q23.3 | 113191052 | G | 0.16 | 0.14 | 1.30 | 1.11-1.52 | 9.56E-04 |  |
| rs7782999 | 7p14.1 | 37446693 | A | 0.24 | 0.21 | 1.25 | 1.09-1.43 | 1.08E-03 |  |
| rs1504183 | 2p16.3 | 49374051 | G | 0.40 | 0.34 | 1.21 | 1.08-1.36 | 1.09E-03 |  |
| rs1437950 | 2q23.3 | 150814389 | C | 0.38 | 0.54 | 0.51 | 0.34-0.76 | 1.15E-03 |  |
| rs726253 | 2q23.3 | 150754826 | A | 0.36 | 0.53 | 0.51 | 0.34-0.76 | 1.15E-03 |  |
| rs713753 | 22q12.3 | 36658534 | C | 0.82 | 0.77 | 1.32 | 1.12-1.57 | 1.15E-03 | ^r^ |
| rs12425157 | 12p13.33 | 1357860 | A | 0.12 | 0.15 | 0.76 | 0.64-0.90 | 1.17E-03 |  |
| rs1298908 | 10q23.1 | 82013134 | G | 0.23 | 0.22 | 1.31 | 1.11-1.54 | 1.20E-03 | ^d^ |
| rs9379119 | 6p24.3 | 7620647 | T | 0.19 | 0.24 | 0.80 | 0.69-0.91 | 1.25E-03 |  |
| rs1424609 | 12q24.21 | 116163432 | G | 0.09 | 0.08 | 1.43 | 1.15-1.79 | 1.39E-03 | ^d^ |
| rs10807889 | 7p14.1 | 37447918 | T | 0.24 | 0.21 | 1.24 | 1.08-1.42 | 1.66E-03 |  |
| rs8002679 | 13q31.2 | 89317062 | C | 0.49 | 0.44 | 1.20 | 1.07-1.35 | 1.71E-03 |  |
| rs17171025 | 7p14.1 | 37444259 | A | 0.27 | 0.24 | 1.22 | 1.08-1.39 | 1.83E-03 |  |
| rs304029 | 3p26.1 | 4545824 | G | 0.31 | 0.28 | 1.21 | 1.07-1.37 | 2.08E-03 |  |
| rs886712 | 7q36.1 | 149102171 | C | 0.33 | 0.30 | 1.28 | 1.09-1.51 | 2.12E-03 | ^d^ |
| rs906584 | 9p21.1 | 32169408 | T | 0.72 | 0.75 | 0.82 | 0.72-0.93 | 2.19E-03 |  |
| rs11042391 | 11p15.5 | 2063598 | T | 0.33 | 0.48 | 0.54 | 0.36-0.80 | 2.21E-03 |  |
| rs674045 | 2q24.3 | 166288036 | A | 0.48 | 0.63 | 0.53 | 0.35-0.80 | 2.76E-03 |  |
| rs12634258 | 3p14.2 | 61291738 | A | 0.69 | 0.69 | 0.83 | 0.73-0.94 | 2.87E-03 |  |
| rs136161 | 22q12.3 | 36657432 | G | 0.78 | 0.74 | 1.28 | 1.09-1.51 | 2.98E-03 | ^r^ |
| rs4887445 | 15q25.3 | 86549964 | G | 0.66 | 0.68 | 0.79 | 0.67-0.92 | 3.36E-03 | ^r^ |
| rs12285658 | 11q21 | 94723623 | G | 0.09 | 0.10 | 0.73 | 0.59-0.90 | 3.52E-03 | ^d^ |
| rs2876167 | 6p24.3 | 9481372 | T | 0.23 | 0.27 | 0.79 | 0.67-0.92 | 3.57E-03 | ^d^ |
| rs2437704 | 16p13.2 | 8950273 | T | 0.39 | 0.35 | 1.27 | 1.08-1.50 | 3.62E-03 | ^d^ |
| rs2471662 | 12q21.1 | 75612012 | A | 0.37 | 0.43 | 0.84 | 0.75-0.95 | 4.16E-03 |  |
| rs768322 | 5q34 | 160531941 | C | 0.11 | 0.08 | 1.32 | 1.09-1.60 | 4.30E-03 |  |
| rs2994388 | 10q23.1 | 82034262 | G | 0.26 | 0.25 | 1.26 | 1.08-1.48 | 4.36E-03 | ^d^ |
| rs8176984 | 10p11.23 | 30731660 | A | 0.31 | 0.34 | 0.84 | 0.74-0.95 | 4.44E-03 |  |
| rs615189 | 9q21.33 | 87697154 | G | 0.61 | 0.64 | 0.79 | 0.67-0.93 | 4.44E-03 | ^r^ |
| rs9590752 | 13q14.11 | 43805598 | G | 0.24 | 0.25 | 0.79 | 0.67-0.93 | 4.46E-03 | ^d^ |
| rs4748900 | 10p14 | 7425786 | T | 0.63 | 0.64 | 0.79 | 0.67-0.93 | 4.55E-03 | ^r^ |
| rs7219778 | 17q25.1 | 71442830 | C | 0.63 | 0.59 | 1.18 | 1.05-1.33 | 4.70E-03 |  |
| rs6729671 | 2q24.3 | 168802437 | G | 0.27 | 0.30 | 0.84 | 0.74-0.95 | 4.91E-03 |  |
| rs1360141 | 9q21.13 | 74292016 | G | 0.46 | 0.49 | 0.85 | 0.76-0.95 | 4.92E-03 |  |
| rs10848438 | 12p13.33 | 1211576 | T | 0.15 | 0.15 | 1.26 | 1.07-1.49 | 5.03E-03 |  |
| rs558584 | 11q12.2 | 60608654 | G | 0.32 | 0.35 | 0.84 | 0.75-0.95 | 5.29E-03 |  |
| rs1331533 | 13q33.1 | 104378857 | G | 0.55 | 0.58 | 0.85 | 0.76-0.95 | 5.59E-03 |  |
| rs7660114 | 4p14 | 38732464 | C | 0.72 | 0.73 | 0.83 | 0.73-0.95 | 5.63E-03 |  |
| rs10004231 | 4p13 | 42857573 | G | 0.45 | 0.47 | 0.85 | 0.76-0.95 | 5.65E-03 |  |
| rs10065785 | 5q22.3 | 113601371 | C | 0.44 | 0.48 | 0.85 | 0.76-0.96 | 6.01E-03 |  |
| rs11185932 | 10q23.1 | 82295398 | A | 0.16 | 0.14 | 1.25 | 1.07-1.47 | 6.10E-03 |  |
| rs6947354 | 7q36.1 | 149097526 | G | 0.45 | 0.41 | 1.27 | 1.07-1.51 | 6.18E-03 | ^d^ |
| rs6542609 | 2p25.2 | 4581932 | T | 0.31 | 0.32 | 0.80 | 0.68-0.94 | 6.33E-03 | ^d^ |
| rs10177009 | 2p25.2 | 4654488 | C | 0.24 | 0.13 | 2.51 | 1.30-4.88 | 6.37E-03 | ^d^ |
| rs11861571 | 16p13.2 | 7954970 | A | 0.22 | 0.10 | 2.78 | 1.33-5.79 | 6.47E-03 | ^d^ |
| rs11073953 | 15q26.1 | 91353584 | A | 0.08 | 0.07 | 1.35 | 1.09-1.68 | 6.50E-03 |  |
| rs6966462 | 7q36.2 | 154157438 | C | 0.24 | 0.26 | 0.80 | 0.68-0.94 | 6.79E-03 | ^d^ |
| rs1476195 | 2q32.1 | 187725158 | G | 0.40 | 0.44 | 0.85 | 0.76-0.96 | 6.82E-03 |  |
| rs12936920 | 17q25.1 | 71422924 | C | 0.35 | 0.33 | 1.25 | 1.06-1.47 | 6.89E-03 | ^d^ |
| rs7454257 | 6q27 | 169212299 | G | 0.19 | 0.17 | 1.26 | 1.07-1.50 | 6.93E-03 | ^d^ |
| rs4863875 | 4q28.3 | 133487356 | C | 0.45 | 0.42 | 1.31 | 1.08-1.60 | 6.99E-03 | ^r^ |
| rs10887933 | 10q23.1 | 82223767 | G | 0.28 | 0.26 | 1.24 | 1.06-1.46 | 7.04E-03 | ^d^ |
| rs16949319 | 17p12 | 14164084 | C | 0.25 | 0.22 | 1.25 | 1.06-1.46 | 7.19E-03 | ^d^ |
| rs12505763 | 4q28.2 | 129216662 | G | 0.64 | 0.60 | 1.25 | 1.06-1.47 | 7.23E-03 | ^r^ |
| rs10873974 | 1p31.1 | 78774623 | G | 0.36 | 0.31 | 1.18 | 1.04-1.32 | 7.34E-03 |  |
| rs12322500 | 12p13.33 | 1211334 | A | 0.22 | 0.25 | 0.83 | 0.73-0.95 | 7.53E-03 |  |
| rs4527915 | 8q12.3 | 65963602 | C | 0.18 | 0.17 | 1.23 | 1.06-1.43 | 7.72E-03 |  |
| rs6570112 | 6q23.3 | 137249971 | T | 0.42 | 0.45 | 0.79 | 0.67-0.94 | 7.73E-03 | ^d^ |
| rs10785180 | 12q21.1 | 75646788 | C | 0.59 | 0.54 | 1.17 | 1.04-1.31 | 8.41E-03 |  |
| rs11136459 | 8p23.3 | 2000626 | C | 0.35 | 0.32 | 1.24 | 1.06-1.46 | 8.52E-03 | ^d^ |
| rs2121323 | 2q24.3 | 168804733 | T | 0.63 | 0.65 | 0.85 | 0.76-0.96 | 8.63E-03 |  |
| rs2884576 | 10p12.31 | 20337118 | A | 0.06 | 0.14 | 0.45 | 0.24-0.82 | 8.70E-03 |  |
| rs280531 | 3q29 | 193424529 | T | 0.26 | 0.24 | 1.24 | 1.06-1.45 | 8.77E-03 | ^d^ |
| rs2385715 | 22q11.1 | 17286147 | T | 0.33 | 0.36 | 0.86 | 0.76-0.96 | 9.08E-03 |  |
| rs595579 | 6q26 | 161371105 | C | 0.23 | 0.28 | 0.84 | 0.74-0.96 | 9.17E-03 |  |
| rs4541508 | 4q25 | 110682487 | G | 0.18 | 0.18 | 1.26 | 1.06-1.50 | 9.22E-03 | ^d^ |
| rs2131007 | 12q23.1 | 100400764 | T | 0.23 | 0.19 | 1.20 | 1.05-1.38 | 9.49E-03 |  |
| rs9478076 | 6q27 | 170011913 | C | 0.49 | 0.44 | 1.16 | 1.04-1.30 | 9.66E-03 |  |
| rs2194519 | 12p11.22 | 29423583 | C | 0.26 | 0.31 | 0.85 | 0.74-0.96 | 9.71E-03 |  |
| rs2146542 | 9q21.33 | 89968917 | C | 0.25 | 0.23 | 1.19 | 1.04-1.35 | 9.73E-03 |  |
| rs3735453 | 7q22.1 | 99229435 | C | 0.59 | 0.59 | 0.80 | 0.68-0.95 | 1.00E-02 | ^r^ |
| rs7583527 | 2q24.3 | 164464056 | T | 0.72 | 0.73 | 0.85 | 0.75-0.96 | 1.03E-02 |  |
| rs4979570 | 9q33.1 | 118392208 | T | 0.58 | 0.53 | 1.16 | 1.04-1.30 | 1.03E-02 |  |
| rs10051548 | 5q21.1 | 101211241 | A | 0.10 | 0.13 | 0.79 | 0.66-0.95 | 1.05E-02 |  |
| rs16936892 | 10p11.21 | 36587989 | C | 0.14 | 0.12 | 1.24 | 1.05-1.47 | 1.09E-02 |  |
| rs4949203 | 1p35.2 | 31781279 | C | 0.17 | 0.15 | 1.22 | 1.05-1.42 | 1.10E-02 |  |
| rs11067690 | 12q24.21 | 116074762 | C | 0.44 | 0.40 | 1.16 | 1.03-1.30 | 1.12E-02 |  |
| rs7533667 | 1p35.2 | 31788246 | G | 0.17 | 0.15 | 1.22 | 1.05-1.42 | 1.12E-02 |  |
| rs6425744 | 1p35.2 | 31850657 | A | 0.17 | 0.15 | 1.22 | 1.05-1.41 | 1.12E-02 |  |
| rs4413687 | 7p21.3 | 10071092 | C | 0.07 | 0.09 | 0.76 | 0.61-0.94 | 1.15E-02 |  |
| rs7003051 | 8q22.3 | 102664291 | A | 0.67 | 0.67 | 0.81 | 0.69-0.96 | 1.18E-02 | ^r^ |
| rs17203808 | 2q36.1 | 224189533 | A | 0.20 | 0.18 | 1.24 | 1.05-1.46 | 1.18E-02 | ^d^ |
| rs4273138 | 18q12.3 | 43239395 | A | 0.09 | 0.16 | 0.43 | 0.23-0.83 | 1.19E-02 | ^d^ |
| rs643938 | 3q13.12 | 107354130 | T | 0.63 | 0.66 | 0.86 | 0.76-0.97 | 1.22E-02 |  |
| rs1510212 | 16q21 | 63110027 | A | 0.70 | 0.71 | 0.85 | 0.75-0.97 | 1.23E-02 |  |
| rs10963125 | 9p22.2 | 17532000 | C | 0.10 | 0.13 | 0.80 | 0.67-0.95 | 1.26E-02 |  |
| rs9981186 | 21q22.2 | 41765173 | A | 0.23 | 0.20 | 1.19 | 1.04-1.36 | 1.26E-02 |  |
| rs10766496 | 11p15.1 | 18738718 | T | 0.18 | 0.21 | 0.81 | 0.68-0.96 | 1.27E-02 | ^d^ |
| rs2355988 | 19q13.31 | 43912939 | A | 0.28 | 0.18 | 2.19 | 1.18-4.06 | 1.34E-02 | ^d^ |
| rs6820719 | 4p15.2 | 23146887 | A | 0.49 | 0.52 | 0.87 | 0.78-0.97 | 1.35E-02 |  |
| rs1551544 | 8q12.1 | 59289347 | C | 0.27 | 0.25 | 1.22 | 1.04-1.43 | 1.35E-02 | ^d^ |
| rs6854600 | 4p14 | 38219277 | A | 0.51 | 0.53 | 0.87 | 0.78-0.97 | 1.36E-02 |  |
| rs11728461 | 4p14 | 40329548 | C | 0.19 | 0.18 | 1.20 | 1.04-1.39 | 1.36E-02 |  |
| rs1510424 | 8p22 | 14450366 | G | 0.07 | 0.06 | 1.34 | 1.06-1.69 | 1.36E-02 | ^d^ |
| rs4734567 | 8q22.3 | 102667066 | A | 0.67 | 0.67 | 0.82 | 0.70-0.96 | 1.38E-02 | ^r^ |
| rs2540934 | 2p22.2 | 37088871 | A | 0.25 | 0.22 | 1.22 | 1.04-1.44 | 1.41E-02 | ^d^ |
| rs2697697 | 4p15.32 | 17448213 | C | 0.13 | 0.10 | 1.24 | 1.04-1.48 | 1.42E-02 |  |
| rs10756491 | 9p23 | 13749917 | T | 0.15 | 0.24 | 0.54 | 0.33-0.88 | 1.44E-02 |  |
| rs2393791 | 12q24.31 | 121423956 | G | 0.30 | 0.33 | 0.82 | 0.70-0.96 | 1.45E-02 | ^d^ |
| rs1290365 | 18p11.21 | 13587740 | A | 0.38 | 0.41 | 0.87 | 0.77-0.97 | 1.48E-02 |  |
| rs1024603 | 14q24.3 | 74295380 | T | 0.22 | 0.25 | 0.82 | 0.70-0.96 | 1.50E-02 | ^d^ |
| rs6659650 | 1p35.2 | 31560369 | T | 0.66 | 0.61 | 1.22 | 1.04-1.44 | 1.51E-02 | ^r^ |
| rs7193727 | 16q23.3 | 83340836 | C | 0.42 | 0.46 | 0.87 | 0.77-0.97 | 1.51E-02 |  |
| rs1436400 | 16q21 | 63326670 | T | 0.13 | 0.21 | 0.52 | 0.31-0.88 | 1.53E-02 |  |
| rs26142 | 5p15.2 | 11474064 | T | 0.23 | 0.22 | 1.18 | 1.03-1.35 | 1.55E-02 |  |
| rs2132173 | 3p21.31 | 47247060 | C | 0.29 | 0.28 | 1.22 | 1.04-1.43 | 1.56E-02 | ^d^ |
| rs17084857 | 13q12.13 | 27469768 | G | 0.05 | 0.07 | 0.74 | 0.59-0.95 | 1.57E-02 |  |
| rs7242586 | 18q22.3 | 70657425 | A | 0.24 | 0.22 | 1.22 | 1.04-1.43 | 1.57E-02 | ^d^ |
| rs2987383 | 9q34.13 | 134703957 | G | 0.07 | 0.06 | 1.34 | 1.06-1.70 | 1.58E-02 | ^d^ |
| rs16847613 | 1q32.1 | 201033720 | T | 0.31 | 0.27 | 1.16 | 1.03-1.31 | 1.58E-02 |  |
| rs10950710 | 7p21.1 | 19349950 | C | 0.29 | 0.32 | 0.82 | 0.70-0.96 | 1.59E-02 | ^d^ |
| rs3103764 | 8q22.1 | 96393670 | C | 0.09 | 0.08 | 1.30 | 1.05-1.61 | 1.59E-02 | ^d^ |
| rs3753373 | 1p35.2 | 31740706 | G | 0.10 | 0.09 | 1.27 | 1.04-1.54 | 1.62E-02 |  |
| rs11154896 | 6q23.3 | 137253534 | G | 0.44 | 0.47 | 0.81 | 0.68-0.96 | 1.67E-02 | ^d^ |
| rs9332410 | 5q35.1 | 171658287 | C | 0.39 | 0.42 | 0.87 | 0.78-0.98 | 1.67E-02 |  |
| rs1771362 | 1p34.3 | 34661652 | G | 0.19 | 0.21 | 0.84 | 0.73-0.97 | 1.69E-02 |  |
| rs903269 | 10p11.23 | 31040517 | T | 0.41 | 0.45 | 0.87 | 0.78-0.98 | 1.70E-02 |  |
| rs13015012 | 2p25.3 | 3821252 | C | 0.23 | 0.26 | 0.85 | 0.75-0.97 | 1.72E-02 |  |
| rs4667466 | 2q24.2 | 163689147 | T | 0.64 | 0.65 | 0.82 | 0.70-0.97 | 1.75E-02 | ^r^ |
| rs5750250 | 22q12.3 | 36708483 | G | 0.58 | 0.52 | 1.15 | 1.02-1.29 | 1.76E-02 |  |
| rs7914650 | 10q23.1 | 82325999 | C | 0.49 | 0.51 | 0.87 | 0.78-0.98 | 1.77E-02 |  |
| rs10269703 | 7q32.3 | 131094679 | A | 0.37 | 0.34 | 1.15 | 1.02-1.29 | 1.77E-02 |  |
| rs10744530 | 12p13.33 | 1186111 | G | 0.13 | 0.13 | 1.23 | 1.04-1.47 | 1.81E-02 |  |
| rs10952362 | 7q36.1 | 152262798 | C | 0.40 | 0.36 | 1.22 | 1.03-1.44 | 1.82E-02 | ^d^ |
| rs4554955 | 12p13.33 | 1229978 | G | 0.59 | 0.69 | 0.58 | 0.37-0.91 | 1.86E-02 |  |
| rs172520 | 6p22.3 | 18258516 | A | 0.60 | 0.55 | 1.15 | 1.02-1.28 | 1.86E-02 |  |
| rs7961413 | 12q23.1 | 98793961 | T | 0.08 | 0.07 | 1.31 | 1.05-1.64 | 1.87E-02 | ^d^ |
| rs924930 | 1p35.2 | 31730086 | C | 0.10 | 0.09 | 1.26 | 1.04-1.53 | 1.88E-02 |  |
| rs4411562 | 17q25.3 | 76047612 | A | 0.24 | 0.21 | 1.17 | 1.03-1.34 | 1.89E-02 |  |
| rs7642022 | 3p21.31 | 48440963 | T | 0.17 | 0.14 | 1.23 | 1.04-1.47 | 1.90E-02 | ^d^ |
| rs197883 | 6q16.1 | 95540600 | A | 0.64 | 0.64 | 0.86 | 0.76-0.98 | 1.91E-02 |  |
| rs999952 | 10q23.31 | 90036680 | C | 0.25 | 0.27 | 0.83 | 0.70-0.97 | 1.92E-02 | ^d^ |
| rs10861796 | 12q21.2 | 79686676 | G | 0.35 | 0.38 | 0.87 | 0.78-0.98 | 1.93E-02 |  |
| rs7900957 | 10q23.1 | 82299847 | T | 0.29 | 0.27 | 1.21 | 1.03-1.42 | 2.00E-02 | ^d^ |
| rs1877119 | 8p23.1 | 8707197 | C | 0.84 | 0.81 | 1.23 | 1.03-1.46 | 2.00E-02 | ^r^ |
| rs10749578 | 10q23.1 | 82139115 | G | 0.08 | 0.07 | 1.31 | 1.04-1.65 | 2.02E-02 | ^d^ |
| rs1149753 | 10q22.3 | 79931986 | T | 0.16 | 0.25 | 0.55 | 0.34-0.91 | 2.03E-02 |  |
| rs11736188 | 4q12 | 54632844 | A | 0.07 | 0.06 | 1.32 | 1.04-1.66 | 2.03E-02 |  |
| rs644570 | 7p21.1 | 18385738 | G | 0.33 | 0.36 | 0.87 | 0.77-0.98 | 2.04E-02 |  |
| rs9640866 | 7q32.3 | 131056641 | G | 0.38 | 0.35 | 1.15 | 1.02-1.29 | 2.05E-02 |  |
| rs2005334 | 1p36.13 | 18467265 | T | 0.49 | 0.51 | 0.88 | 0.78-0.98 | 2.06E-02 |  |
| rs7291344 | 22q12.3 | 32930142 | G | 0.26 | 0.36 | 0.60 | 0.39-0.93 | 2.09E-02 |  |
| rs574075 | 11q24.3 | 128827052 | T | 0.30 | 0.34 | 0.87 | 0.77-0.98 | 2.13E-02 |  |
| rs6000209 | 22q12.3 | 36626724 | G | 0.18 | 0.10 | 2.37 | 1.14-4.94 | 2.14E-02 | ^d^ |
| rs12295437 | 11p15.2 | 12994339 | C | 0.18 | 0.15 | 1.19 | 1.03-1.38 | 2.15E-02 |  |
| rs12076492 | 1p31.3 | 62489016 | T | 0.23 | 0.20 | 1.17 | 1.02-1.34 | 2.18E-02 |  |
| rs9503215 | 6p25.2 | 2508395 | C | 0.19 | 0.16 | 1.19 | 1.03-1.37 | 2.18E-02 |  |
| rs12914880 | 15q22.2 | 60142340 | C | 0.05 | 0.07 | 0.75 | 0.59-0.96 | 2.18E-02 |  |
| rs877718 | 7q33 | 134354841 | G | 0.10 | 0.12 | 0.80 | 0.65-0.97 | 2.23E-02 | ^d^ |
| rs7651455 | 3p12.3 | 77950275 | C | 0.19 | 0.20 | 0.82 | 0.70-0.97 | 2.24E-02 | ^d^ |
| rs6105151 | 20p12.1 | 13691752 | C | 0.35 | 0.32 | 1.15 | 1.02-1.29 | 2.28E-02 |  |
| rs12592875 | 15q26.1 | 91353604 | C | 0.18 | 0.16 | 1.19 | 1.02-1.38 | 2.32E-02 |  |
| rs17183344 | 15q26.1 | 91357488 | T | 0.25 | 0.26 | 0.86 | 0.75-0.98 | 2.36E-02 |  |
| rs9678660 | 2q14.3 | 124291689 | T | 0.12 | 0.21 | 0.56 | 0.34-0.93 | 2.37E-02 |  |
| rs6544922 | 2p21 | 47011923 | G | 0.25 | 0.29 | 0.86 | 0.76-0.98 | 2.38E-02 |  |
| rs9592817 | 13q21.33 | 72589470 | G | 0.17 | 0.18 | 1.63 | 1.07-2.48 | 2.39E-02 | ^r^ |
| rs7214934 | 17p12 | 11307195 | A | 0.74 | 0.71 | 1.20 | 1.02-1.41 | 2.41E-02 | ^r^ |
| rs2550621 | 16q23.1 | 78675531 | A | 0.14 | 0.14 | 1.21 | 1.03-1.43 | 2.41E-02 |  |
| rs2669460 | 13q33.1 | 104643963 | A | 0.46 | 0.48 | 0.88 | 0.78-0.98 | 2.42E-02 |  |
| rs6768844 | 3p21.1 | 53004620 | A | 0.29 | 0.26 | 1.15 | 1.02-1.30 | 2.47E-02 |  |
| rs6474701 | 9p23 | 12333231 | C | 0.68 | 0.66 | 1.36 | 1.04-1.77 | 2.47E-02 | ^d^ |
| rs7715214 | 5q34 | 160626657 | T | 0.12 | 0.10 | 1.23 | 1.03-1.46 | 2.48E-02 |  |
| rs6863774 | 5q11.2 | 55695116 | A | 0.14 | 0.13 | 1.20 | 1.02-1.41 | 2.50E-02 |  |
| rs11086554 | 20q13.31 | 55171694 | G | 0.23 | 0.20 | 1.17 | 1.02-1.34 | 2.50E-02 |  |
| rs17373728 | 8q21.11 | 76225516 | C | 0.18 | 0.11 | 2.32 | 1.11-4.85 | 2.56E-02 | ^d^ |
| rs6921476 | 6p21.2 | 37548407 | A | 0.47 | 0.49 | 0.81 | 0.67-0.97 | 2.62E-02 | ^r^ |
| rs6813952 | 4q13.3 | 74844432 | C | 0.06 | 0.07 | 0.76 | 0.59-0.97 | 2.63E-02 | ^d^ |
| rs7316817 | 12p13.2 | 10909412 | C | 0.14 | 0.16 | 0.82 | 0.68-0.98 | 2.64E-02 | ^d^ |
| rs786318 | 9q21.2 | 79233313 | G | 0.63 | 0.64 | 0.88 | 0.78-0.99 | 2.70E-02 |  |
| rs2687864 | 3q24 | 145423008 | A | 0.21 | 0.24 | 0.86 | 0.75-0.98 | 2.72E-02 |  |
| rs6571786 | 14q13.3 | 37598506 | A | 0.14 | 0.12 | 1.20 | 1.02-1.41 | 2.75E-02 |  |
| rs1373813 | 3q22.2 | 135021043 | T | 0.60 | 0.61 | 0.88 | 0.79-0.99 | 2.75E-02 |  |
| rs2087357 | 9p13.2 | 37405235 | T | 0.07 | 0.06 | 1.29 | 1.03-1.61 | 2.77E-02 |  |
| rs7249040 | 19q13.33 | 49790602 | T | 0.30 | 0.32 | 0.87 | 0.77-0.99 | 2.80E-02 |  |
| rs10520673 | 15q25.3 | 88617721 | A | 0.23 | 0.14 | 1.92 | 1.07-3.44 | 2.80E-02 |  |
| rs7035756 | 9p24.1 | 6331274 | G | 0.06 | 0.07 | 0.76 | 0.59-0.97 | 2.81E-02 | ^d^ |
| rs1539857 | 18q21.2 | 48697936 | C | 0.27 | 0.28 | 0.87 | 0.76-0.99 | 2.83E-02 |  |
| rs7945128 | 11p11.2 | 43517122 | C | 0.65 | 0.61 | 1.14 | 1.01-1.27 | 2.89E-02 |  |
| rs1469438 | 5q35.1 | 170719260 | C | 0.61 | 0.60 | 0.78 | 0.63-0.98 | 2.92E-02 | ^d^ |
| rs6892117 | 5q33.1 | 151264985 | C | 0.24 | 0.27 | 0.87 | 0.76-0.99 | 2.96E-02 |  |
| rs295433 | 3p21.31 | 47362080 | T | 0.23 | 0.21 | 1.16 | 1.01-1.33 | 2.98E-02 |  |
| rs1055483 | 19q13.41 | 51676568 | C | 0.42 | 0.44 | 0.88 | 0.78-0.99 | 3.03E-02 |  |
| rs17353111 | 7p21.1 | 19407264 | G | 0.12 | 0.16 | 0.83 | 0.70-0.98 | 3.05E-02 |  |
| rs17017980 | 3p24.1 | 26446285 | T | 0.63 | 0.61 | 1.20 | 1.02-1.41 | 3.06E-02 | ^r^ |
| rs2827183 | 21q21.1 | 23358505 | G | 0.51 | 0.48 | 1.13 | 1.01-1.26 | 3.07E-02 |  |
| rs772598 | 1p22.3 | 87039945 | T | 0.46 | 0.46 | 1.23 | 1.02-1.48 | 3.08E-02 | ^r^ |

Direction: RA is reference allele. The odds ratio (OR) is presented for the reference allele, compared with the non-reference allele, for a given model.

**Supplemental Table S6b.**

**Top 200 associations from the FIND Replication – American Indian**

| **SNP** | **Cytoband** | **Position** | **RA** | **Case  RAF** | **Control RAF** | **OR** | **95% CI** | **P-value** | |
| --- | --- | --- | --- | --- | --- | --- | --- | --- | --- |
| rs4980233 | 10q26.13 | 124465540 | C | 0.23 | 0.31 | 0.55 | 0.44-0.70 | 1.10E-06 | ^d^ |
| rs1402572 | 6q25.2 | 154965837 | G | 0.19 | 0.27 | 0.56 | 0.44-0.71 | 1.26E-06 | ^d^ |
| rs12523822 | 6q25.2 | 154954420 | T | 0.18 | 0.26 | 0.56 | 0.44-0.71 | 1.76E-06 | ^d^ |
| rs1521279 | 6q25.2 | 154953099 | T | 0.18 | 0.26 | 0.56 | 0.44-0.71 | 1.76E-06 | ^d^ |
| rs1149753 | 10q22.3 | 79931986 | T | 0.17 | 0.10 | 1.74 | 1.39-2.19 | 1.93E-06 |  |
| rs279072 | 13q14.3 | 51119737 | T | 0.18 | 0.25 | 0.57 | 0.45-0.73 | 6.49E-06 | ^d^ |
| rs7895470 | 10p12.31 | 20235463 | C | 0.62 | 0.53 | 1.47 | 1.24-1.75 | 1.05E-05 |  |
| rs6474701 | 9p23 | 12333231 | C | 0.05 | 0.09 | 0.45 | 0.31-0.64 | 1.13E-05 | ^d^ |
| rs6432852 | 2q24.3 | 166754553 | G | 0.20 | 0.27 | 0.59 | 0.47-0.75 | 1.27E-05 | ^d^ |
| rs11011694 | 10p12.31 | 20237290 | G | 0.37 | 0.45 | 0.59 | 0.46-0.75 | 1.48E-05 | ^d^ |
| rs955333 | 6q25.2 | 154947408 | G | 0.17 | 0.24 | 0.59 | 0.46-0.75 | 1.63E-05 | ^d^ |
| rs807744 | 22q11.21 | 19104186 | A | 0.36 | 0.28 | 1.45 | 1.22-1.72 | 2.26E-05 |  |
| rs11785793 | 8p23.3 | 1365687 | A | 0.19 | 0.12 | 1.60 | 1.28-1.99 | 2.64E-05 |  |
| rs10019835 | 4q32.1 | 156633186 | T | 0.29 | 0.37 | 0.61 | 0.48-0.77 | 2.87E-05 | ^d^ |
| rs13421350 | 2q31.1 | 173318571 | A | 0.10 | 0.15 | 0.59 | 0.45-0.76 | 4.90E-05 |  |
| rs11976696 | 7p11.2 | 55232333 | C | 0.44 | 0.53 | 0.71 | 0.60-0.84 | 4.96E-05 |  |
| rs10987414 | 9q33.3 | 129459478 | A | 0.18 | 0.12 | 1.72 | 1.32-2.24 | 5.02E-05 | ^d^ |
| rs317866 | 4p15.32 | 16156273 | A | 0.52 | 0.61 | 0.71 | 0.60-0.84 | 5.78E-05 |  |
| rs13254600 | 8q24.13 | 124089526 | T | 0.16 | 0.23 | 0.61 | 0.48-0.78 | 7.81E-05 | ^d^ |
| rs12469173 | 2p16.1 | 59802586 | G | 0.63 | 0.56 | 1.40 | 1.18-1.65 | 9.39E-05 |  |
| rs10952362 | 7q36.1 | 152262798 | C | 0.58 | 0.50 | 1.38 | 1.17-1.63 | 1.20E-04 |  |
| rs17313372 | 8q24.13 | 124070432 | G | 0.16 | 0.22 | 0.63 | 0.49-0.80 | 1.78E-04 | ^d^ |
| rs4667466 | 2q24.2 | 163689147 | T | 0.48 | 0.39 | 1.36 | 1.16-1.60 | 1.87E-04 |  |
| rs6112221 | 20p11.23 | 19077650 | T | 0.38 | 0.46 | 0.73 | 0.62-0.86 | 1.91E-04 |  |
| rs10826267 | 10q21.1 | 60793767 | G | 0.64 | 0.72 | 0.72 | 0.60-0.85 | 1.93E-04 |  |
| rs217682 | 14q23.2 | 62356724 | G | 0.40 | 0.32 | 1.56 | 1.23-1.97 | 2.13E-04 | ^d^ |
| rs1896886 | 7q31.2 | 117200510 | A | 0.60 | 0.53 | 1.36 | 1.15-1.60 | 2.22E-04 |  |
| rs1510424 | 8p22 | 14450366 | G | 0.41 | 0.48 | 0.62 | 0.49-0.80 | 2.26E-04 | ^d^ |
| rs4730779 | 7q31.2 | 117023535 | G | 0.34 | 0.41 | 0.65 | 0.51-0.82 | 2.45E-04 | ^d^ |
| rs2157946 | 7q31.2 | 117030698 | G | 0.34 | 0.41 | 0.65 | 0.51-0.82 | 2.53E-04 | ^d^ |
| rs4148689 | 7q31.2 | 117143602 | G | 0.60 | 0.53 | 1.35 | 1.15-1.59 | 2.58E-04 |  |
| rs1437934 | 2q23.3 | 150765489 | T | 0.50 | 0.43 | 1.36 | 1.15-1.61 | 2.71E-04 |  |
| rs7332267 | 13q21.33 | 72509652 | C | 0.44 | 0.37 | 1.34 | 1.14-1.58 | 3.08E-04 |  |
| rs9592817 | 13q21.33 | 72589470 | G | 0.42 | 0.50 | 0.73 | 0.62-0.87 | 3.28E-04 |  |
| rs1878776 | 8q24.13 | 124103395 | T | 0.15 | 0.21 | 0.63 | 0.49-0.81 | 3.37E-04 | ^d^ |
| rs6488494 | 12p13.2 | 12229952 | A | 0.47 | 0.55 | 0.75 | 0.64-0.88 | 3.51E-04 |  |
| rs1887035 | 10p12.31 | 20208995 | T | 0.63 | 0.56 | 1.36 | 1.15-1.61 | 3.58E-04 |  |
| rs4384817 | 2p13.1 | 74226141 | A | 0.14 | 0.09 | 1.66 | 1.26-2.19 | 3.71E-04 | ^d^ |
| rs1441541 | 5p15.33 | 3250352 | A | 0.20 | 0.14 | 1.46 | 1.19-1.81 | 3.90E-04 |  |
| rs2099610 | 2q23.3 | 150784214 | T | 0.50 | 0.43 | 1.35 | 1.14-1.59 | 3.91E-04 |  |
| rs586294 | 1q32.2 | 208184309 | A | 0.27 | 0.21 | 1.40 | 1.16-1.68 | 4.03E-04 |  |
| rs7808424 | 7q31.2 | 117067822 | C | 0.61 | 0.54 | 1.35 | 1.14-1.59 | 4.07E-04 |  |
| rs684968 | 4p15.2 | 23496919 | C | 0.13 | 0.08 | 1.62 | 1.24-2.11 | 4.14E-04 |  |
| rs2283054 | 7q31.2 | 117126401 | T | 0.59 | 0.52 | 1.34 | 1.14-1.57 | 4.53E-04 |  |
| rs2077212 | 8p23.2 | 5982272 | G | 0.56 | 0.48 | 1.33 | 1.13-1.55 | 4.77E-04 |  |
| rs9937918 | 16q21 | 57601792 | T | 0.83 | 0.79 | 1.58 | 1.22-2.04 | 4.82E-04 | ^r^ |
| rs891382 | 4q31.22 | 147152340 | C | 0.09 | 0.05 | 1.83 | 1.30-2.57 | 5.15E-04 | ^d^ |
| rs4128419 | 8q12.1 | 61112594 | A | 0.30 | 0.25 | 1.50 | 1.19-1.88 | 5.45E-04 | ^d^ |
| rs12429087 | 13q21.31 | 62773498 | A | 0.11 | 0.07 | 1.65 | 1.24-2.19 | 5.56E-04 |  |
| rs4453858 | 3p14.1 | 67457498 | T | 0.51 | 0.59 | 0.74 | 0.63-0.88 | 5.61E-04 |  |
| rs6940797 | 6q15 | 90208120 | T | 0.37 | 0.42 | 0.66 | 0.52-0.84 | 5.92E-04 | ^d^ |
| rs4317189 | 4p15.2 | 23495538 | T | 0.13 | 0.08 | 1.60 | 1.22-2.09 | 5.97E-04 |  |
| rs1440363 | 8q13.3 | 73577503 | G | 0.30 | 0.35 | 0.73 | 0.61-0.87 | 6.06E-04 |  |
| rs6947354 | 7q36.1 | 149097526 | G | 0.33 | 0.39 | 0.67 | 0.53-0.84 | 6.09E-04 | ^d^ |
| rs4922234 | 8p22 | 16639271 | G | 0.49 | 0.43 | 1.33 | 1.13-1.56 | 6.70E-04 |  |
| rs43025 | 7q31.2 | 117104507 | T | 0.63 | 0.56 | 1.50 | 1.19-1.91 | 7.23E-04 | ^r^ |
| rs9918936 | 9p24.3 | 1511341 | G | 0.65 | 0.71 | 0.67 | 0.53-0.84 | 7.46E-04 | ^r^ |
| rs10927873 | 1p36.13 | 16251900 | A | 0.26 | 0.31 | 0.67 | 0.53-0.85 | 8.31E-04 | ^d^ |
| rs1290365 | 18p11.21 | 13587740 | A | 0.20 | 0.15 | 1.52 | 1.19-1.94 | 8.48E-04 | ^d^ |
| rs1567256 | 9p21.1 | 32169502 | A | 0.32 | 0.39 | 0.74 | 0.63-0.89 | 9.00E-04 |  |
| rs11208609 | 1p31.3 | 65678419 | G | 0.25 | 0.19 | 1.41 | 1.15-1.73 | 9.10E-04 |  |
| rs9310806 | 3p24.1 | 26551492 | A | 0.29 | 0.22 | 1.36 | 1.13-1.64 | 9.68E-04 |  |
| rs6723745 | 2p21 | 42090563 | A | 0.36 | 0.43 | 0.75 | 0.64-0.89 | 9.90E-04 |  |
| rs1555380 | 6q22.31 | 124654043 | T | 0.43 | 0.35 | 1.32 | 1.12-1.56 | 1.03E-03 |  |
| rs894784 | 15q25.1 | 79351176 | C | 0.09 | 0.13 | 0.61 | 0.46-0.82 | 1.03E-03 | ^d^ |
| rs1946384 | 12q24.21 | 116080723 | C | 0.35 | 0.28 | 1.34 | 1.13-1.60 | 1.04E-03 |  |
| rs10885650 | 10q25.3 | 116856071 | G | 0.14 | 0.19 | 0.69 | 0.55-0.86 | 1.06E-03 |  |
| rs12505641 | 4p15.2 | 23488892 | G | 0.19 | 0.13 | 1.49 | 1.17-1.89 | 1.07E-03 |  |
| rs734650 | 8q24.11 | 118719341 | G | 0.33 | 0.27 | 1.46 | 1.16-1.84 | 1.15E-03 | ^d^ |
| rs2298489 | 11q23.2 | 113235419 | T | 0.67 | 0.61 | 1.33 | 1.12-1.58 | 1.16E-03 |  |
| rs6081489 | 20p11.23 | 19076763 | A | 0.57 | 0.50 | 1.31 | 1.11-1.54 | 1.18E-03 |  |
| rs2788371 | 1p34.1 | 44343962 | G | 0.26 | 0.32 | 0.68 | 0.54-0.86 | 1.19E-03 | ^d^ |
| rs4884986 | 13q21.33 | 72653545 | T | 0.54 | 0.48 | 1.31 | 1.11-1.55 | 1.20E-03 |  |
| rs12469941 | 2q21.3 | 135629927 | G | 0.44 | 0.51 | 0.77 | 0.66-0.90 | 1.25E-03 |  |
| rs12497655 | 3q13.31 | 116764010 | G | 0.21 | 0.27 | 0.73 | 0.61-0.89 | 1.31E-03 |  |
| rs11214579 | 11q23.2 | 113208186 | C | 0.68 | 0.62 | 1.33 | 1.12-1.58 | 1.34E-03 |  |
| rs17594509 | 4p12 | 45307178 | A | 0.39 | 0.33 | 1.31 | 1.11-1.55 | 1.36E-03 |  |
| rs1081796 | 13q13.1 | 33036759 | A | 0.41 | 0.35 | 1.31 | 1.11-1.54 | 1.40E-03 |  |
| rs7990113 | 13q12.3 | 29898058 | C | 0.22 | 0.17 | 1.40 | 1.14-1.73 | 1.41E-03 |  |
| rs635594 | 8p23.1 | 8613387 | C | 0.52 | 0.48 | 1.52 | 1.18-1.97 | 1.43E-03 | ^r^ |
| rs16831882 | 3q26.1 | 160799812 | A | 0.18 | 0.13 | 1.43 | 1.15-1.78 | 1.50E-03 |  |
| rs2279267 | 4q33 | 170990053 | A | 0.28 | 0.22 | 1.35 | 1.12-1.63 | 1.62E-03 |  |
| rs12202357 | 6q24.3 | 146733674 | C | 0.13 | 0.09 | 1.51 | 1.17-1.96 | 1.71E-03 |  |
| rs11243273 | 6p24.3 | 8745358 | C | 0.35 | 0.39 | 0.69 | 0.55-0.87 | 1.72E-03 | ^d^ |
| rs726253 | 2q23.3 | 150754826 | A | 0.41 | 0.35 | 1.31 | 1.11-1.55 | 1.75E-03 |  |
| rs10274145 | 7p22.3 | 1614261 | T | 0.14 | 0.10 | 1.47 | 1.15-1.87 | 1.79E-03 |  |
| rs13231611 | 7q36.1 | 149099314 | T | 0.32 | 0.37 | 0.69 | 0.55-0.87 | 1.82E-03 | ^d^ |
| rs2698784 | 12q13.11 | 47780595 | C | 0.40 | 0.37 | 1.65 | 1.20-2.25 | 1.87E-03 | ^r^ |
| rs604924 | 6q26 | 161383479 | T | 0.35 | 0.29 | 1.30 | 1.10-1.54 | 1.89E-03 |  |
| rs13259109 | 8q23.3 | 113115897 | G | 0.38 | 0.32 | 1.45 | 1.15-1.83 | 1.93E-03 | ^d^ |
| rs12314846 | 12q15 | 68097596 | G | 0.42 | 0.48 | 0.68 | 0.53-0.87 | 1.96E-03 | ^d^ |
| rs1922159 | 10q21.1 | 56819932 | G | 0.33 | 0.28 | 1.33 | 1.11-1.59 | 1.99E-03 |  |
| rs12622724 | 2q31.1 | 173324908 | A | 0.10 | 0.15 | 0.67 | 0.53-0.87 | 1.99E-03 |  |
| rs444018 | 6p25.3 | 1217500 | G | 0.20 | 0.15 | 1.39 | 1.13-1.71 | 2.00E-03 |  |
| rs307869 | 2q24.3 | 163761591 | A | 0.42 | 0.36 | 1.29 | 1.10-1.51 | 2.03E-03 |  |
| rs6602820 | 10p15.1 | 6596242 | G | 0.19 | 0.24 | 0.69 | 0.54-0.87 | 2.04E-03 | ^d^ |
| rs6942294 | 6q24.2 | 143707820 | T | 0.10 | 0.06 | 1.65 | 1.20-2.27 | 2.08E-03 | ^d^ |
| rs9541141 | 13q21.32 | 68095175 | A | 0.33 | 0.38 | 0.69 | 0.55-0.88 | 2.10E-03 | ^d^ |
| rs1574131 | 4p16.1 | 8041800 | A | 0.20 | 0.25 | 0.69 | 0.54-0.87 | 2.12E-03 | ^d^ |
| rs10906396 | 10p13 | 13551876 | A | 0.50 | 0.57 | 0.78 | 0.67-0.91 | 2.13E-03 |  |
| rs2612848 | 13q21.33 | 72564477 | A | 0.46 | 0.53 | 0.77 | 0.65-0.91 | 2.15E-03 |  |
| rs12579556 | 12p13.32 | 4237871 | C | 0.53 | 0.47 | 1.29 | 1.10-1.51 | 2.16E-03 |  |
| rs1999435 | 9p24.3 | 1513849 | C | 0.65 | 0.70 | 0.69 | 0.55-0.88 | 2.19E-03 | ^r^ |
| rs10502124 | 11q23.1 | 110894702 | C | 0.22 | 0.27 | 0.75 | 0.62-0.90 | 2.21E-03 |  |
| rs1469629 | 11q23.1 | 110895414 | G | 0.22 | 0.27 | 0.75 | 0.62-0.90 | 2.21E-03 |  |
| rs10778560 | 12q23.3 | 107801401 | C | 0.11 | 0.07 | 1.56 | 1.17-2.07 | 2.30E-03 |  |
| rs749137 | 12q23.3 | 107801897 | G | 0.11 | 0.07 | 1.56 | 1.17-2.07 | 2.30E-03 |  |
| rs10997295 | 10q21.3 | 68437615 | T | 0.76 | 0.73 | 1.44 | 1.14-1.82 | 2.32E-03 | ^r^ |
| rs2252500 | 8q23.3 | 113191052 | G | 0.39 | 0.32 | 1.44 | 1.14-1.82 | 2.33E-03 | ^d^ |
| rs17415295 | 1q32.1 | 204949826 | C | 0.09 | 0.06 | 1.57 | 1.17-2.10 | 2.34E-03 |  |
| rs17730056 | 11q23.1 | 110882350 | T | 0.16 | 0.21 | 0.68 | 0.53-0.87 | 2.35E-03 | ^d^ |
| rs17535075 | 11q23.1 | 110919219 | C | 0.19 | 0.24 | 0.69 | 0.54-0.88 | 2.44E-03 | ^d^ |
| rs4638339 | 11q23.1 | 110897937 | A | 0.19 | 0.24 | 0.69 | 0.54-0.88 | 2.44E-03 | ^d^ |
| rs2203817 | 3q13.31 | 116741261 | G | 0.20 | 0.26 | 0.75 | 0.62-0.90 | 2.57E-03 |  |
| rs1409571 | 13q21.33 | 72672465 | C | 0.41 | 0.47 | 0.77 | 0.66-0.91 | 2.62E-03 |  |
| rs1025897 | 4p15.32 | 16224242 | C | 0.58 | 0.65 | 0.78 | 0.66-0.92 | 2.72E-03 |  |
| rs2605883 | 8q21.11 | 74187254 | A | 0.23 | 0.22 | 2.15 | 1.30-3.55 | 2.75E-03 | ^r^ |
| rs9573355 | 13q22.1 | 74734988 | T | 0.80 | 0.76 | 1.44 | 1.13-1.82 | 2.77E-03 | ^r^ |
| rs16859648 | 3q24 | 147394647 | A | 0.25 | 0.21 | 1.42 | 1.13-1.80 | 2.84E-03 | ^d^ |
| rs9878045 | 3p24.3 | 20193100 | G | 0.12 | 0.08 | 1.48 | 1.14-1.92 | 3.05E-03 |  |
| rs6780449 | 3q21.3 | 125816063 | A | 0.72 | 0.78 | 0.75 | 0.62-0.91 | 3.06E-03 |  |
| rs10484795 | 6q23.3 | 137316830 | T | 0.12 | 0.16 | 0.70 | 0.55-0.88 | 3.07E-03 |  |
| rs10489271 | 1q25.1 | 173067216 | G | 0.22 | 0.28 | 0.75 | 0.62-0.91 | 3.20E-03 |  |
| rs4609646 | 12q24.21 | 116079028 | C | 0.60 | 0.67 | 0.76 | 0.63-0.91 | 3.21E-03 |  |
| rs6117253 | 20p12.3 | 6410235 | T | 0.60 | 0.67 | 0.78 | 0.66-0.92 | 3.38E-03 |  |
| rs740150 | 7q34 | 139675510 | G | 0.28 | 0.32 | 0.71 | 0.57-0.89 | 3.48E-03 | ^d^ |
| rs4922151 | 8p21.3 | 20141092 | T | 0.34 | 0.28 | 1.29 | 1.09-1.54 | 3.52E-03 |  |
| rs6773337 | 3p14.1 | 68851208 | G | 0.32 | 0.38 | 0.77 | 0.65-0.92 | 3.63E-03 |  |
| rs1242272 | 12q21.2 | 78328395 | C | 0.17 | 0.12 | 1.39 | 1.11-1.73 | 3.68E-03 |  |
| rs1894372 | 14q11.2 | 22634808 | G | 0.35 | 0.41 | 0.79 | 0.67-0.93 | 3.81E-03 |  |
| rs10440735 | 5q33.1 | 150976380 | T | 0.43 | 0.40 | 1.56 | 1.15-2.11 | 3.87E-03 | ^r^ |
| rs4876279 | 8q23.3 | 113205771 | A | 0.38 | 0.32 | 1.41 | 1.12-1.78 | 3.87E-03 | ^d^ |
| rs9367268 | 6p12.3 | 47192438 | T | 0.29 | 0.24 | 1.40 | 1.11-1.76 | 4.04E-03 | ^d^ |
| rs12621026 | 2q36.3 | 228822187 | A | 0.14 | 0.18 | 0.69 | 0.54-0.89 | 4.17E-03 | ^d^ |
| rs11758932 | 6q24.2 | 143701194 | C | 0.14 | 0.10 | 1.43 | 1.12-1.83 | 4.29E-03 |  |
| rs1563870 | 13q21.33 | 72503423 | T | 0.41 | 0.35 | 1.27 | 1.08-1.49 | 4.30E-03 |  |
| rs485325 | 11q14.1 | 78401625 | A | 0.15 | 0.17 | 0.69 | 0.53-0.89 | 4.32E-03 | ^d^ |
| rs12508126 | 4q32.2 | 162957578 | C | 0.25 | 0.20 | 1.32 | 1.09-1.59 | 4.34E-03 |  |
| rs947810 | 11q14.1 | 77172856 | A | 0.20 | 0.16 | 1.35 | 1.10-1.65 | 4.45E-03 |  |
| rs9294977 | 6q27 | 169624172 | G | 0.21 | 0.26 | 0.71 | 0.57-0.90 | 4.48E-03 | ^d^ |
| rs325833 | 5p13.1 | 41034181 | T | 0.44 | 0.38 | 1.26 | 1.07-1.48 | 4.51E-03 |  |
| rs302531 | 4q28.1 | 124166489 | G | 0.24 | 0.28 | 0.72 | 0.57-0.90 | 4.60E-03 | ^d^ |
| rs17554527 | 4p15.2 | 23519755 | A | 0.11 | 0.07 | 1.58 | 1.15-2.17 | 4.70E-03 | ^d^ |
| rs7201146 | 16p12.1 | 27102315 | A | 0.47 | 0.41 | 1.26 | 1.07-1.48 | 4.74E-03 |  |
| rs4626416 | 6p22.3 | 23135926 | A | 0.26 | 0.20 | 1.34 | 1.09-1.64 | 4.74E-03 |  |
| rs330102 | 6p24.3 | 8629179 | T | 0.35 | 0.38 | 0.72 | 0.57-0.90 | 4.88E-03 | ^d^ |
| rs3121757 | 13q12.3 | 30000197 | G | 0.31 | 0.26 | 1.39 | 1.10-1.74 | 4.97E-03 | ^d^ |
| rs9379119 | 6p24.3 | 7620647 | T | 0.47 | 0.54 | 0.79 | 0.68-0.93 | 5.08E-03 |  |
| rs701305 | 7q21.11 | 83556579 | G | 0.05 | 0.08 | 0.61 | 0.43-0.86 | 5.08E-03 |  |
| rs164016 | 19p13.3 | 6820951 | C | 0.47 | 0.43 | 1.49 | 1.13-1.97 | 5.14E-03 | ^r^ |
| rs11107504 | 12q21.2 | 78336732 | A | 0.16 | 0.12 | 1.37 | 1.10-1.71 | 5.22E-03 |  |
| rs17791199 | 13q21.33 | 72676321 | A | 0.37 | 0.44 | 0.78 | 0.66-0.93 | 5.61E-03 |  |
| rs12711963 | 2p25.2 | 4973678 | C | 0.15 | 0.11 | 1.41 | 1.10-1.79 | 5.66E-03 |  |
| rs12590425 | 14q23.1 | 61886770 | T | 0.29 | 0.33 | 0.78 | 0.65-0.93 | 5.69E-03 |  |
| rs2055052 | 8q22.1 | 96392380 | T | 0.05 | 0.07 | 0.58 | 0.39-0.85 | 5.77E-03 | ^d^ |
| rs1531246 | 12q24.32 | 128999121 | C | 0.29 | 0.36 | 0.79 | 0.66-0.93 | 5.89E-03 |  |
| rs3930757 | 7p15.3 | 22142073 | G | 0.48 | 0.42 | 1.25 | 1.07-1.47 | 5.91E-03 |  |
| rs16838982 | 3q21.3 | 127639072 | G | 0.19 | 0.23 | 0.72 | 0.57-0.91 | 5.91E-03 | ^d^ |
| rs7147710 | 14q24.1 | 69905725 | T | 0.16 | 0.19 | 0.71 | 0.55-0.90 | 5.94E-03 | ^d^ |
| rs11067690 | 12q24.21 | 116074762 | C | 0.68 | 0.74 | 0.77 | 0.64-0.93 | 6.00E-03 |  |
| rs4858753 | 3p24.3 | 20116904 | C | 0.41 | 0.36 | 1.26 | 1.07-1.49 | 6.21E-03 |  |
| rs4858754 | 3p24.3 | 20116934 | G | 0.41 | 0.36 | 1.26 | 1.07-1.49 | 6.21E-03 |  |
| rs10847812 | 12q24.33 | 129730925 | A | 0.11 | 0.08 | 1.51 | 1.13-2.04 | 6.22E-03 | ^d^ |
| rs6977665 | 7q31.2 | 116976830 | A | 0.44 | 0.50 | 0.80 | 0.68-0.94 | 6.27E-03 |  |
| rs277478 | 7q32.3 | 132114671 | T | 0.29 | 0.24 | 1.28 | 1.07-1.53 | 6.40E-03 |  |
| rs4077563 | 3q21.2 | 125769757 | A | 0.73 | 0.79 | 0.77 | 0.63-0.93 | 6.45E-03 |  |
| rs10840257 | 11p15.4 | 9568463 | C | 0.13 | 0.09 | 1.42 | 1.10-1.83 | 6.74E-03 |  |
| rs1026727 | 13q31.1 | 81824427 | T | 0.42 | 0.37 | 1.39 | 1.10-1.77 | 6.84E-03 | ^d^ |
| rs1270773 | 12q21.2 | 78332786 | C | 0.16 | 0.12 | 1.36 | 1.09-1.69 | 6.88E-03 |  |
| rs206335 | 13q13.1 | 32999951 | G | 0.40 | 0.46 | 0.80 | 0.68-0.94 | 7.18E-03 |  |
| rs10109898 | 8q24.23 | 136819328 | T | 0.23 | 0.28 | 0.72 | 0.57-0.92 | 7.18E-03 | ^d^ |
| rs2358874 | 10p12.31 | 20476246 | A | 0.08 | 0.06 | 1.59 | 1.13-2.22 | 7.20E-03 | ^d^ |
| rs16920484 | 10q21.3 | 66612353 | G | 0.50 | 0.54 | 0.70 | 0.54-0.91 | 7.22E-03 | ^d^ |
| rs16946009 | 12q24.21 | 116098300 | G | 0.60 | 0.67 | 0.78 | 0.65-0.94 | 7.80E-03 |  |
| rs1822063 | 8q13.3 | 73584744 | G | 0.43 | 0.48 | 0.80 | 0.68-0.94 | 7.96E-03 |  |
| rs2118828 | 2q13 | 110877051 | G | 0.35 | 0.41 | 0.80 | 0.67-0.94 | 8.06E-03 |  |
| rs11166618 | 8q24.23 | 136816446 | A | 0.23 | 0.28 | 0.73 | 0.58-0.92 | 8.26E-03 | ^d^ |
| rs752697 | 20q11.23 | 34746826 | C | 0.10 | 0.14 | 0.70 | 0.54-0.91 | 8.33E-03 |  |
| rs310231 | 1p31.3 | 65340817 | C | 0.57 | 0.63 | 0.79 | 0.67-0.94 | 8.47E-03 |  |
| rs6422705 | 2p25.2 | 4975953 | T | 0.15 | 0.11 | 1.38 | 1.08-1.75 | 8.53E-03 |  |
| rs12511735 | 4p16.1 | 8105010 | C | 0.26 | 0.29 | 0.74 | 0.58-0.93 | 8.71E-03 | ^d^ |
| rs310230 | 1p31.3 | 65341002 | A | 0.57 | 0.63 | 0.79 | 0.67-0.94 | 8.73E-03 |  |
| rs528213 | 2q37.3 | 240127076 | T | 0.57 | 0.51 | 1.24 | 1.06-1.45 | 8.92E-03 |  |
| rs3870431 | 15q25.3 | 87895595 | C | 0.45 | 0.49 | 0.72 | 0.56-0.92 | 9.18E-03 | ^d^ |
| rs2482971 | 9p22.1 | 18682952 | G | 0.16 | 0.19 | 0.72 | 0.56-0.92 | 9.24E-03 | ^d^ |
| rs3739542 | 9p21.2 | 27172838 | C | 0.37 | 0.33 | 1.36 | 1.08-1.72 | 9.33E-03 | ^d^ |
| rs2881640 | 15q25.3 | 87460714 | T | 0.24 | 0.28 | 0.74 | 0.58-0.93 | 9.35E-03 | ^d^ |
| rs10143250 | 14q32.33 | 104723433 | T | 0.39 | 0.33 | 1.25 | 1.06-1.48 | 9.45E-03 |  |
| rs310236 | 1p31.3 | 65333857 | G | 0.42 | 0.37 | 1.25 | 1.06-1.49 | 9.61E-03 |  |
| rs441083 | 16q23.2 | 79228051 | C | 0.64 | 0.59 | 1.25 | 1.06-1.48 | 9.70E-03 |  |
| rs16862540 | 3q25.1 | 149887788 | C | 0.19 | 0.24 | 0.76 | 0.62-0.94 | 9.72E-03 |  |
| rs16960761 | 19q13.11 | 34432677 | G | 0.37 | 0.44 | 0.80 | 0.67-0.95 | 9.92E-03 |  |
| rs12305552 | 12q12 | 41797386 | A | 0.23 | 0.18 | 1.29 | 1.06-1.56 | 1.00E-02 |  |
| rs4810486 | 20q13.12 | 44766403 | G | 0.43 | 0.38 | 1.24 | 1.05-1.46 | 1.02E-02 |  |
| rs10891786 | 11q23.3 | 114843409 | T | 0.21 | 0.26 | 0.78 | 0.64-0.94 | 1.03E-02 |  |
| rs2284796 | 6q24.3 | 146746895 | C | 0.15 | 0.19 | 0.72 | 0.56-0.93 | 1.03E-02 | ^d^ |
| rs2393791 | 12q24.31 | 121423956 | G | 0.45 | 0.40 | 1.23 | 1.05-1.44 | 1.03E-02 |  |
| rs1331647 | 13q31.1 | 81934444 | C | 0.42 | 0.37 | 1.24 | 1.05-1.46 | 1.04E-02 |  |
| rs2122926 | 15q25.3 | 87879196 | G | 0.45 | 0.49 | 0.72 | 0.56-0.93 | 1.05E-02 | ^d^ |
| rs2348145 | 15q25.3 | 87878603 | C | 0.45 | 0.49 | 0.72 | 0.56-0.93 | 1.05E-02 | ^d^ |

Direction: RA is reference allele. The odds ratio (OR) is presented for the reference allele, compared with the non-reference allele, for a given model.

**Supplemental Table S6c.**

**Top 200 associations from the FIND Replication – European Ancestry**

| **SNP** | **Cytoband** | **Position** | **RA** | **Case  RAF** | **Control RAF** | **OR** | **95% CI** | **P-value** | |
| --- | --- | --- | --- | --- | --- | --- | --- | --- | --- |
| rs11198399 | 10q26.11 | 120177638 | C | 0.25 | 0.21 | 1.42 | 1.18-1.70 | 1.70E-04 | ^d^ |
| rs736830 | 6q21 | 109713299 | A | 0.61 | 0.57 | 1.40 | 1.16-1.69 | 3.65E-04 | ^r^ |
| rs1198061 | 10q21.1 | 60773378 | A | 0.61 | 0.56 | 1.39 | 1.16-1.68 | 5.33E-04 | ^r^ |
| rs11815025 | 10q21.1 | 61075275 | A | 0.17 | 0.13 | 1.41 | 1.16-1.72 | 5.98E-04 | ^d^ |
| rs1563916 | 8p21.3 | 21085331 | C | 0.42 | 0.38 | 1.25 | 1.09-1.42 | 1.03E-03 |  |
| rs4791207 | 17q24.1 | 63272453 | C | 0.24 | 0.28 | 0.74 | 0.62-0.89 | 1.26E-03 | ^d^ |
| rs10129960 | 14q21.2 | 43929782 | G | 0.52 | 0.46 | 1.23 | 1.08-1.40 | 1.34E-03 |  |
| rs353957 | 5q14.1 | 77113027 | A | 0.43 | 0.45 | 0.73 | 0.61-0.89 | 1.36E-03 | ^d^ |
| rs7650427 | 3p24.3 | 16522582 | G | 0.45 | 0.50 | 0.81 | 0.71-0.92 | 1.53E-03 |  |
| rs9644619 | 8p22 | 18987347 | G | 0.36 | 0.32 | 1.24 | 1.09-1.42 | 1.58E-03 |  |
| rs7636648 | 3q21.1 | 123624772 | A | 0.58 | 0.62 | 0.81 | 0.72-0.92 | 1.61E-03 |  |
| rs4796906 | 18p11.22 | 10838059 | G | 0.65 | 0.60 | 1.23 | 1.08-1.41 | 1.85E-03 |  |
| rs6841655 | 4p14 | 37358920 | T | 0.43 | 0.47 | 0.82 | 0.72-0.93 | 2.12E-03 |  |
| rs9294977 | 6q27 | 169624172 | G | 0.30 | 0.33 | 0.75 | 0.63-0.90 | 2.29E-03 | ^d^ |
| rs2910642 | 5q11.2 | 58396111 | C | 0.30 | 0.35 | 0.81 | 0.70-0.93 | 2.88E-03 |  |
| rs9900825 | 17q12 | 36152099 | A | 0.33 | 0.29 | 1.23 | 1.07-1.41 | 3.11E-03 |  |
| rs10859060 | 12q21.33 | 91225941 | G | 0.34 | 0.38 | 0.82 | 0.72-0.94 | 3.24E-03 |  |
| rs7249040 | 19q13.33 | 49790602 | T | 0.23 | 0.20 | 1.26 | 1.08-1.48 | 3.39E-03 |  |
| rs2930961 | 8q22.1 | 95443806 | G | 0.35 | 0.31 | 1.23 | 1.07-1.41 | 3.49E-03 |  |
| rs1479842 | 7q35 | 147025943 | A | 0.39 | 0.44 | 0.83 | 0.72-0.94 | 3.77E-03 |  |
| rs10882641 | 10q24.1 | 97371903 | G | 0.51 | 0.42 | 1.39 | 1.11-1.74 | 4.06E-03 |  |
| rs2647537 | 11p15.4 | 9125301 | C | 0.39 | 0.43 | 0.82 | 0.72-0.94 | 4.09E-03 |  |
| rs17531828 | 22q11.23 | 23558754 | G | 0.13 | 0.15 | 0.73 | 0.59-0.91 | 4.29E-03 | ^d^ |
| rs12469173 | 2p16.1 | 59802586 | G | 0.26 | 0.29 | 0.77 | 0.64-0.92 | 4.52E-03 | ^d^ |
| rs10491615 | 9p23 | 12984009 | C | 0.38 | 0.43 | 0.83 | 0.72-0.94 | 4.54E-03 |  |
| rs30101 | 5q11.2 | 52417299 | A | 0.64 | 0.59 | 1.21 | 1.06-1.39 | 4.76E-03 |  |
| rs12509404 | 4q27 | 122446364 | T | 0.09 | 0.12 | 0.73 | 0.59-0.91 | 4.81E-03 |  |
| rs6916579 | 6q21 | 109851638 | G | 0.32 | 0.35 | 0.77 | 0.64-0.92 | 4.97E-03 | ^d^ |
| rs7097483 | 10q11.23 | 52005303 | C | 0.12 | 0.09 | 1.33 | 1.09-1.63 | 5.39E-03 |  |
| rs2212596 | 21q22.2 | 39968534 | C | 0.52 | 0.49 | 1.33 | 1.08-1.62 | 6.06E-03 | ^r^ |
| rs4730287 | 7q31.1 | 107770624 | C | 0.48 | 0.50 | 0.74 | 0.59-0.92 | 6.17E-03 | ^r^ |
| rs164016 | 19p13.3 | 6820951 | C | 0.41 | 0.36 | 1.20 | 1.05-1.37 | 6.20E-03 |  |
| rs973060 | 18q22.2 | 68658623 | C | 0.38 | 0.34 | 1.20 | 1.05-1.37 | 6.30E-03 |  |
| rs10782162 | 6q21 | 109806263 | A | 0.32 | 0.35 | 0.78 | 0.65-0.93 | 6.34E-03 | ^d^ |
| rs7003051 | 8q22.3 | 102664291 | A | 0.31 | 0.27 | 1.21 | 1.06-1.39 | 6.48E-03 |  |
| rs1436217 | 10q11.23 | 52002852 | C | 0.12 | 0.09 | 1.33 | 1.08-1.63 | 6.63E-03 |  |
| rs17183713 | 5q14.1 | 77083796 | G | 0.09 | 0.12 | 0.72 | 0.57-0.92 | 6.92E-03 | ^d^ |
| rs11697869 | 20p13 | 1174075 | C | 0.06 | 0.09 | 0.70 | 0.55-0.91 | 6.96E-03 |  |
| rs267184 | 6p24.3 | 7833429 | C | 0.52 | 0.56 | 0.84 | 0.74-0.95 | 6.97E-03 |  |
| rs9493435 | 6q23.2 | 133088205 | G | 0.13 | 0.10 | 1.30 | 1.07-1.58 | 7.15E-03 |  |
| rs12505102 | 4p13 | 42722174 | G | 0.05 | 0.08 | 0.69 | 0.52-0.90 | 7.46E-03 |  |
| rs17083406 | 18q22.2 | 68672955 | A | 0.36 | 0.32 | 1.20 | 1.05-1.38 | 7.53E-03 |  |
| rs10007956 | 4q27 | 122700669 | T | 0.42 | 0.44 | 0.71 | 0.56-0.91 | 7.63E-03 | ^r^ |
| rs17070580 | 6q21 | 109738714 | C | 0.34 | 0.37 | 0.78 | 0.65-0.94 | 7.71E-03 | ^d^ |
| rs233935 | 6q23.2 | 132350493 | G | 0.44 | 0.40 | 1.19 | 1.05-1.35 | 7.80E-03 |  |
| rs4734567 | 8q22.3 | 102667066 | A | 0.30 | 0.27 | 1.28 | 1.07-1.53 | 7.92E-03 | ^d^ |
| rs6716270 | 2p14 | 66201731 | C | 0.13 | 0.16 | 0.77 | 0.64-0.94 | 8.00E-03 |  |
| rs9885646 | 6q21 | 109742460 | T | 0.34 | 0.37 | 0.78 | 0.65-0.94 | 8.05E-03 | ^d^ |
| rs955333 | 6q25.2 | 154947408 | G | 0.13 | 0.16 | 0.75 | 0.61-0.93 | 8.19E-03 | ^d^ |
| rs2393791 | 12q24.31 | 121423956 | G | 0.43 | 0.39 | 1.19 | 1.05-1.35 | 8.20E-03 |  |
| rs2171226 | 16p13.3 | 5588451 | T | 0.40 | 0.44 | 0.84 | 0.74-0.96 | 8.42E-03 |  |
| rs17310144 | 3q21.1 | 123665902 | C | 0.59 | 0.63 | 0.84 | 0.74-0.96 | 8.46E-03 |  |
| rs2357442 | 14q22.1 | 52607967 | C | 0.11 | 0.14 | 0.74 | 0.59-0.93 | 8.86E-03 | ^d^ |
| rs6850716 | 4p15.1 | 32582470 | C | 0.24 | 0.30 | 0.72 | 0.56-0.92 | 8.92E-03 |  |
| rs17194017 | 2q24.3 | 164456577 | G | 0.06 | 0.08 | 0.70 | 0.53-0.92 | 9.37E-03 |  |
| rs17472293 | 5q14.1 | 79133987 | A | 0.39 | 0.41 | 0.78 | 0.65-0.94 | 9.42E-03 | ^d^ |
| rs11690661 | 2q23.3 | 153774953 | A | 0.35 | 0.28 | 1.38 | 1.08-1.77 | 9.49E-03 |  |
| rs6948299 | 7p15.3 | 23982979 | G | 0.56 | 0.60 | 0.85 | 0.75-0.96 | 9.77E-03 |  |
| rs12504227 | 4p13 | 42721872 | T | 0.05 | 0.08 | 0.70 | 0.53-0.92 | 1.01E-02 |  |
| rs11930136 | 4q35.2 | 190278973 | A | 0.09 | 0.07 | 1.37 | 1.08-1.74 | 1.02E-02 | ^d^ |
| rs7703101 | 5q22.3 | 114597146 | C | 0.11 | 0.09 | 1.32 | 1.07-1.62 | 1.02E-02 |  |
| rs17729793 | 6p12.3 | 48087812 | C | 0.23 | 0.27 | 0.82 | 0.71-0.96 | 1.02E-02 |  |
| rs7432694 | 3q13.13 | 110930787 | T | 0.53 | 0.57 | 0.85 | 0.74-0.96 | 1.03E-02 |  |
| rs11716997 | 3q21.3 | 127551630 | C | 0.39 | 0.43 | 0.84 | 0.74-0.96 | 1.03E-02 |  |
| rs7782999 | 7p14.1 | 37446693 | A | 0.16 | 0.20 | 0.80 | 0.67-0.95 | 1.04E-02 |  |
| rs378963 | 6p24.3 | 7888328 | G | 0.58 | 0.61 | 0.84 | 0.74-0.96 | 1.09E-02 |  |
| rs4323164 | 4q35.2 | 187996662 | T | 0.13 | 0.15 | 0.76 | 0.61-0.94 | 1.09E-02 | ^d^ |
| rs10744530 | 12p13.33 | 1186111 | G | 0.63 | 0.59 | 1.19 | 1.04-1.35 | 1.12E-02 |  |
| rs215700 | 7p14.3 | 32399166 | A | 0.26 | 0.28 | 0.79 | 0.66-0.95 | 1.15E-02 | ^d^ |
| rs2876847 | 5p14.1 | 27108052 | T | 0.21 | 0.19 | 1.27 | 1.05-1.53 | 1.17E-02 | ^d^ |
| rs2880475 | 4q13.1 | 60874182 | C | 0.16 | 0.14 | 1.29 | 1.06-1.57 | 1.17E-02 | ^d^ |
| rs7572957 | 2p23.2 | 29487405 | C | 0.07 | 0.09 | 0.73 | 0.57-0.93 | 1.19E-02 |  |
| rs1369645 | 15q21.1 | 47788809 | A | 0.32 | 0.29 | 1.19 | 1.04-1.37 | 1.22E-02 |  |
| rs7076242 | 10q11.23 | 52002204 | A | 0.11 | 0.10 | 1.30 | 1.06-1.59 | 1.22E-02 |  |
| rs2236582 | 6q21 | 109765818 | C | 0.32 | 0.35 | 0.79 | 0.66-0.95 | 1.23E-02 | ^d^ |
| rs4554955 | 12p13.33 | 1229978 | G | 0.23 | 0.29 | 0.72 | 0.56-0.93 | 1.24E-02 |  |
| rs6096260 | 20q13.13 | 49715775 | T | 0.41 | 0.39 | 1.35 | 1.07-1.71 | 1.25E-02 | ^r^ |
| rs6437957 | 3q13.13 | 110934660 | A | 0.28 | 0.26 | 1.26 | 1.05-1.51 | 1.31E-02 | ^d^ |
| rs10872032 | 6q21 | 109676776 | T | 0.34 | 0.40 | 0.67 | 0.49-0.92 | 1.33E-02 | ^d^ |
| rs12448794 | 16p13.3 | 7196111 | T | 0.09 | 0.12 | 0.75 | 0.59-0.94 | 1.34E-02 | ^d^ |
| rs7430175 | 3q13.13 | 110938375 | C | 0.51 | 0.54 | 0.85 | 0.75-0.97 | 1.34E-02 |  |
| rs6484371 | 11p14.1 | 28438811 | A | 0.48 | 0.52 | 0.85 | 0.74-0.97 | 1.37E-02 |  |
| rs10807889 | 7p14.1 | 37447918 | T | 0.17 | 0.20 | 0.81 | 0.68-0.96 | 1.38E-02 |  |
| rs2432755 | 6p25.1 | 5387513 | G | 0.40 | 0.37 | 1.18 | 1.03-1.35 | 1.46E-02 |  |
| rs10794280 | 11p15.5 | 1062867 | T | 0.40 | 0.37 | 1.18 | 1.03-1.34 | 1.47E-02 |  |
| rs7637487 | 3p14.2 | 61282503 | A | 0.15 | 0.13 | 1.25 | 1.05-1.50 | 1.47E-02 |  |
| rs7143182 | 14q32.2 | 99428834 | T | 0.57 | 0.58 | 0.78 | 0.64-0.95 | 1.48E-02 | ^r^ |
| rs2826862 | 21q21.1 | 22865641 | G | 0.07 | 0.09 | 0.72 | 0.55-0.94 | 1.49E-02 | ^d^ |
| rs1888835 | 6q27 | 169574568 | G | 0.54 | 0.56 | 0.76 | 0.61-0.95 | 1.53E-02 | ^d^ |
| rs6996860 | 8p11.23 | 38243865 | G | 0.26 | 0.23 | 1.20 | 1.04-1.39 | 1.55E-02 |  |
| rs17171025 | 7p14.1 | 37444259 | A | 0.16 | 0.20 | 0.81 | 0.68-0.96 | 1.55E-02 |  |
| rs13119941 | 4p16.1 | 8100158 | A | 0.32 | 0.30 | 1.25 | 1.04-1.50 | 1.57E-02 | ^d^ |
| rs9480957 | 6q21 | 109751601 | T | 0.32 | 0.35 | 0.80 | 0.67-0.96 | 1.58E-02 | ^d^ |
| rs12530998 | 7p15.3 | 22822662 | T | 0.45 | 0.41 | 1.17 | 1.03-1.33 | 1.61E-02 |  |
| rs9877709 | 3p14.2 | 61282140 | C | 0.15 | 0.13 | 1.25 | 1.04-1.50 | 1.61E-02 |  |
| rs12440759 | 15q21.1 | 47804269 | G | 0.31 | 0.29 | 1.25 | 1.04-1.50 | 1.63E-02 | ^d^ |
| rs17109004 | 1p31.1 | 83191278 | C | 0.30 | 0.32 | 0.84 | 0.73-0.97 | 1.64E-02 |  |
| rs12501307 | 4p15.33 | 13540518 | A | 0.21 | 0.18 | 1.21 | 1.04-1.42 | 1.66E-02 |  |
| rs4135268 | 3p25.2 | 12437237 | C | 0.10 | 0.08 | 1.33 | 1.05-1.67 | 1.67E-02 | ^d^ |
| rs12499281 | 4q34.3 | 179694687 | A | 0.09 | 0.07 | 1.34 | 1.05-1.70 | 1.68E-02 | ^d^ |
| rs10820207 | 9q22.32 | 98408548 | T | 0.10 | 0.08 | 1.31 | 1.05-1.63 | 1.70E-02 |  |
| rs7733389 | 5q35.3 | 178974997 | C | 0.55 | 0.59 | 0.86 | 0.75-0.97 | 1.72E-02 |  |
| rs10990134 | 9q22.32 | 98407644 | A | 0.10 | 0.08 | 1.30 | 1.05-1.62 | 1.72E-02 |  |
| rs1572280 | 9q22.32 | 98401842 | C | 0.10 | 0.09 | 1.30 | 1.05-1.62 | 1.80E-02 |  |
| rs1402457 | 3q22.1 | 132765826 | T | 0.09 | 0.06 | 1.75 | 1.10-2.77 | 1.80E-02 | ^d^ |
| rs17108945 | 1p31.1 | 83170482 | A | 0.32 | 0.35 | 0.85 | 0.74-0.97 | 1.82E-02 |  |
| rs7869414 | 9p22.1 | 19082832 | A | 0.13 | 0.11 | 1.26 | 1.04-1.53 | 1.84E-02 |  |
| rs1537089 | 21q21.3 | 28499328 | A | 0.24 | 0.27 | 0.80 | 0.67-0.96 | 1.84E-02 | ^d^ |
| rs17415295 | 1q32.1 | 204949826 | C | 0.10 | 0.12 | 0.77 | 0.62-0.96 | 1.86E-02 |  |
| rs11032216 | 11p15.4 | 4441838 | A | 0.40 | 0.38 | 1.26 | 1.04-1.52 | 1.87E-02 | ^d^ |
| rs9605422 | 22q11.21 | 18362940 | T | 0.18 | 0.21 | 0.82 | 0.70-0.97 | 1.90E-02 |  |
| rs11582609 | 1p31.1 | 83220656 | A | 0.30 | 0.33 | 0.85 | 0.74-0.97 | 1.90E-02 |  |
| rs7652939 | 3p14.2 | 61287140 | G | 0.62 | 0.65 | 0.85 | 0.75-0.97 | 1.92E-02 |  |
| rs6709904 | 2p21 | 44080324 | C | 0.09 | 0.11 | 0.77 | 0.62-0.96 | 1.94E-02 |  |
| rs10830457 | 11q14.3 | 89975099 | G | 0.20 | 0.22 | 0.80 | 0.66-0.96 | 1.96E-02 | ^d^ |
| rs1500251 | 5q21.1 | 98593453 | T | 0.18 | 0.22 | 0.82 | 0.70-0.97 | 1.98E-02 |  |
| rs11079035 | 17q21.2 | 40289012 | T | 0.19 | 0.16 | 1.25 | 1.04-1.52 | 1.99E-02 | ^d^ |
| rs2582905 | 11p14.1 | 28510100 | G | 0.48 | 0.52 | 0.85 | 0.75-0.98 | 2.02E-02 |  |
| rs7655238 | 4p14 | 37045620 | C | 0.36 | 0.38 | 0.81 | 0.67-0.97 | 2.05E-02 | ^d^ |
| rs2829862 | 21q21.3 | 27054612 | C | 0.45 | 0.44 | 1.29 | 1.04-1.61 | 2.05E-02 | ^r^ |
| rs3859865 | 22q12.1 | 26158284 | T | 0.20 | 0.22 | 0.80 | 0.66-0.97 | 2.08E-02 | ^d^ |
| rs528213 | 2q37.3 | 240127076 | T | 0.63 | 0.61 | 1.38 | 1.05-1.81 | 2.10E-02 | ^d^ |
| rs1167827 | 7q11.23 | 75163169 | A | 0.39 | 0.42 | 0.86 | 0.75-0.98 | 2.14E-02 |  |
| rs2194519 | 12p11.22 | 29423583 | C | 0.34 | 0.37 | 0.85 | 0.75-0.98 | 2.15E-02 |  |
| rs4425416 | 4p14 | 40973209 | A | 0.26 | 0.30 | 0.84 | 0.73-0.98 | 2.15E-02 |  |
| rs10016635 | 4q34.2 | 176676766 | C | 0.09 | 0.11 | 0.75 | 0.59-0.96 | 2.16E-02 | ^d^ |
| rs10827221 | 10p11.22 | 33545464 | C | 0.35 | 0.31 | 1.17 | 1.02-1.34 | 2.16E-02 |  |
| rs2836555 | 21q22.2 | 39973752 | C | 0.25 | 0.30 | 0.74 | 0.58-0.96 | 2.18E-02 |  |
| rs1467404 | 6q23.2 | 133090141 | A | 0.13 | 0.11 | 1.25 | 1.03-1.51 | 2.19E-02 |  |
| rs13208889 | 6q13 | 71751997 | T | 0.21 | 0.27 | 0.75 | 0.58-0.96 | 2.22E-02 |  |
| rs261956 | 6p22.1 | 30264933 | A | 0.08 | 0.06 | 1.35 | 1.04-1.75 | 2.23E-02 | ^d^ |
| rs9298290 | 8q21.11 | 78039167 | T | 0.29 | 0.26 | 1.18 | 1.02-1.36 | 2.24E-02 |  |
| rs17552605 | 18p11.32 | 2791071 | C | 0.11 | 0.14 | 0.79 | 0.65-0.97 | 2.25E-02 |  |
| rs1167796 | 7q11.23 | 75173180 | T | 0.39 | 0.42 | 0.86 | 0.75-0.98 | 2.30E-02 |  |
| rs16903220 | 5q14.3 | 87820740 | A | 0.10 | 0.09 | 1.30 | 1.04-1.63 | 2.31E-02 | ^d^ |
| rs10989496 | 9q22.32 | 98397006 | T | 0.10 | 0.08 | 1.29 | 1.04-1.61 | 2.31E-02 |  |
| rs6686264 | 1p34.3 | 38192337 | T | 0.31 | 0.35 | 0.85 | 0.74-0.98 | 2.36E-02 |  |
| rs156436 | 5q11.2 | 53376613 | G | 0.40 | 0.38 | 1.24 | 1.03-1.50 | 2.39E-02 | ^d^ |
| rs13248631 | 8p11.23 | 38260815 | G | 0.52 | 0.56 | 0.86 | 0.76-0.98 | 2.41E-02 |  |
| rs17569795 | 14q12 | 26688690 | T | 0.36 | 0.39 | 0.86 | 0.75-0.98 | 2.41E-02 |  |
| rs1326005 | 1p36.31 | 5403585 | A | 0.37 | 0.40 | 0.86 | 0.75-0.98 | 2.45E-02 |  |
| rs6782715 | 3q25.1 | 150968277 | C | 0.54 | 0.50 | 1.16 | 1.02-1.31 | 2.46E-02 |  |
| rs1901892 | 3p14.2 | 61318721 | A | 0.18 | 0.16 | 1.25 | 1.03-1.52 | 2.46E-02 | ^d^ |
| rs17797945 | 18q11.2 | 22046656 | A | 0.25 | 0.28 | 0.81 | 0.68-0.97 | 2.48E-02 | ^d^ |
| rs10989157 | 9q22.32 | 98388345 | T | 0.10 | 0.08 | 1.29 | 1.03-1.60 | 2.50E-02 |  |
| rs9646322 | 16q23.1 | 79164851 | A | 0.24 | 0.21 | 1.19 | 1.02-1.38 | 2.50E-02 |  |
| rs7724668 | 5q35.3 | 180552412 | G | 0.42 | 0.38 | 1.16 | 1.02-1.32 | 2.54E-02 |  |
| rs12606602 | 18p11.22 | 8971951 | T | 0.42 | 0.44 | 0.80 | 0.67-0.97 | 2.55E-02 | ^d^ |
| rs6934161 | 6q23.2 | 133124077 | C | 0.13 | 0.11 | 1.24 | 1.03-1.50 | 2.56E-02 |  |
| rs11712096 | 3p22.1 | 42129108 | T | 0.43 | 0.40 | 1.16 | 1.02-1.32 | 2.57E-02 |  |
| rs7139704 | 13q14.2 | 47726258 | C | 0.25 | 0.22 | 1.18 | 1.02-1.37 | 2.59E-02 |  |
| rs1361933 | 20q13.13 | 46469317 | A | 0.32 | 0.30 | 1.23 | 1.03-1.47 | 2.60E-02 | ^d^ |
| rs11082423 | 18q11.2 | 20929994 | T | 0.51 | 0.49 | 1.26 | 1.03-1.54 | 2.65E-02 | ^r^ |
| rs267186 | 6p24.3 | 7834019 | C | 0.56 | 0.62 | 0.77 | 0.61-0.97 | 2.67E-02 |  |
| rs12508139 | 4q32.3 | 169322975 | G | 0.41 | 0.38 | 1.24 | 1.02-1.50 | 2.68E-02 | ^d^ |
| rs1859547 | 7q35 | 147200815 | C | 0.27 | 0.30 | 0.85 | 0.74-0.98 | 2.74E-02 |  |
| rs2582908 | 11p14.1 | 28522535 | C | 0.51 | 0.48 | 1.16 | 1.02-1.33 | 2.81E-02 |  |
| rs206335 | 13q13.1 | 32999951 | G | 0.40 | 0.43 | 0.86 | 0.76-0.98 | 2.81E-02 |  |
| rs7858186 | 9q31.2 | 109897161 | A | 0.42 | 0.45 | 0.87 | 0.76-0.98 | 2.82E-02 |  |
| rs6482376 | 10p12.2 | 24534039 | A | 0.21 | 0.24 | 0.84 | 0.72-0.98 | 2.87E-02 |  |
| rs7193727 | 16q23.3 | 83340836 | C | 0.57 | 0.53 | 1.15 | 1.02-1.31 | 2.87E-02 |  |
| rs1213659 | 1p32.2 | 58827046 | C | 0.11 | 0.15 | 0.67 | 0.47-0.96 | 2.89E-02 | ^d^ |
| rs12363459 | 11p14.1 | 28535612 | A | 0.52 | 0.49 | 1.16 | 1.02-1.33 | 2.89E-02 |  |
| rs12550148 | 8q24.13 | 126749121 | C | 0.14 | 0.12 | 1.26 | 1.02-1.55 | 2.91E-02 | ^d^ |
| rs7067773 | 10q11.23 | 52005271 | G | 0.10 | 0.08 | 1.28 | 1.02-1.59 | 2.94E-02 |  |
| rs1320025 | 3q24 | 147468001 | T | 0.20 | 0.18 | 1.20 | 1.02-1.41 | 2.94E-02 |  |
| rs202281 | 8q21.13 | 82203988 | A | 0.14 | 0.11 | 1.23 | 1.02-1.48 | 2.95E-02 |  |
| rs10176602 | 2p13.1 | 74120785 | T | 0.27 | 0.24 | 1.17 | 1.02-1.36 | 3.01E-02 |  |
| rs10135930 | 14q22.1 | 52609680 | G | 0.12 | 0.15 | 0.81 | 0.67-0.98 | 3.04E-02 |  |
| rs10497275 | 2q24.3 | 166846730 | C | 0.12 | 0.14 | 0.80 | 0.66-0.98 | 3.05E-02 |  |
| rs7769051 | 6q23.2 | 133146796 | T | 0.13 | 0.11 | 1.24 | 1.02-1.5 | 3.05E-02 |  |
| rs7956509 | 12q24.21 | 115910853 | T | 0.48 | 0.50 | 0.87 | 0.76-0.99 | 3.07E-02 |  |
| rs17686792 | 18q12.3 | 41613422 | G | 0.25 | 0.27 | 0.82 | 0.68-0.98 | 3.09E-02 | ^d^ |
| rs17763208 | 4p15.1 | 30455150 | G | 0.31 | 0.33 | 0.82 | 0.68-0.98 | 3.11E-02 | ^d^ |
| rs16851332 | 2q24.3 | 166845303 | A | 0.12 | 0.14 | 0.80 | 0.66-0.98 | 3.13E-02 |  |
| rs7606753 | 2q36.1 | 221556078 | G | 0.63 | 0.58 | 1.16 | 1.01-1.32 | 3.14E-02 |  |
| rs133882 | 22q12.1 | 26158628 | C | 0.22 | 0.24 | 0.82 | 0.68-0.98 | 3.15E-02 | ^d^ |
| rs10783957 | 12q14.1 | 59738145 | G | 0.16 | 0.18 | 0.83 | 0.69-0.98 | 3.21E-02 |  |
| rs3217753 | 4q27 | 122746325 | G | 0.34 | 0.31 | 1.22 | 1.02-1.46 | 3.28E-02 | ^d^ |
| rs305461 | 1p22.3 | 88189692 | G | 0.21 | 0.25 | 0.85 | 0.72-0.99 | 3.32E-02 |  |
| rs7526329 | 1q42.12 | 224617323 | C | 0.16 | 0.18 | 0.81 | 0.66-0.98 | 3.34E-02 | ^d^ |
| rs6540495 | 1q32.2 | 209046438 | A | 0.43 | 0.46 | 0.87 | 0.77-0.99 | 3.45E-02 |  |
| rs1476603 | 7q21.3 | 96614877 | C | 0.14 | 0.12 | 1.25 | 1.02-1.54 | 3.48E-02 | ^d^ |
| rs4338649 | 13q33.1 | 103658465 | A | 0.16 | 0.18 | 0.83 | 0.70-0.99 | 3.50E-02 |  |
| rs7130471 | 11p14.1 | 28456861 | C | 0.51 | 0.48 | 1.15 | 1.01-1.32 | 3.51E-02 |  |
| rs217181 | 16q22.2 | 72114002 | A | 0.17 | 0.19 | 0.81 | 0.67-0.99 | 3.56E-02 | ^d^ |
| rs12620007 | 2q33.3 | 207601691 | A | 0.58 | 0.53 | 1.27 | 1.02-1.59 | 3.59E-02 |  |
| rs1399903 | 3q26.1 | 161394104 | G | 0.10 | 0.08 | 1.26 | 1.02-1.56 | 3.59E-02 |  |
| rs9846724 | 3q13.31 | 114083774 | C | 0.37 | 0.40 | 0.87 | 0.76-0.99 | 3.62E-02 |  |
| rs10948402 | 6p12.3 | 48156101 | A | 0.25 | 0.28 | 0.86 | 0.74-0.99 | 3.62E-02 |  |
| rs17130136 | 1p22.3 | 88213827 | A | 0.09 | 0.11 | 0.79 | 0.64-0.99 | 3.65E-02 |  |
| rs2997581 | 9p24.1 | 7410738 | G | 0.47 | 0.47 | 0.79 | 0.63-0.99 | 3.66E-02 | ^r^ |
| rs972884 | 12p13.32 | 4937575 | A | 0.26 | 0.30 | 0.86 | 0.74-0.99 | 3.67E-02 |  |
| rs4887211 | 15q25.3 | 88754891 | C | 0.10 | 0.12 | 0.78 | 0.62-0.99 | 3.67E-02 | ^d^ |
| rs2193174 | 12p12.3 | 15304410 | C | 0.31 | 0.33 | 0.82 | 0.69-0.99 | 3.67E-02 | ^d^ |
| rs7131839 | 12p12.3 | 15280915 | T | 0.31 | 0.33 | 0.83 | 0.69-0.99 | 3.72E-02 | ^d^ |
| rs1950377 | 14q13.3 | 37558794 | T | 0.08 | 0.07 | 1.31 | 1.02-1.69 | 3.77E-02 | ^d^ |
| rs2837336 | 21q22.2 | 41338014 | A | 0.34 | 0.33 | 1.21 | 1.01-1.46 | 3.83E-02 | ^d^ |
| rs696284 | 9p13.3 | 33202548 | C | 0.33 | 0.34 | 0.83 | 0.69-0.99 | 3.87E-02 | ^d^ |
| rs9493454 | 6q23.2 | 133144629 | G | 0.13 | 0.11 | 1.22 | 1.01-1.48 | 3.92E-02 |  |

Direction: RA is reference allele. The odds ratio (OR) is presented for the reference allele, compared with the non-reference allele, for a given model.
