# Supplementary material for: Genome-Wide Association and Trans-ethnic Meta-Analysis for Advanced Diabetic Kidney Disease: Family Investigation of Nephropathy and Diabetes (FIND)
Source: PLoS Genet. 2015 Aug 25;11(8):e1005352. doi: 10.1371/journal.pgen.1005352 (PMC4549309; doi:10.1371/journal.pgen.1005352)
Supplement: S7 Table — (DOCX) [file pgen.1005352.s008.docx]

**Supplemental Table S7:**

**Top 200 associations from the FIND African American GWAS, excluding subjects with 2 copies of APOL1 G1 and/or G2 risk variants**

| **SNP** | **Cytoband** | **Position** | **RA** | **Case  RAF** | **Control RAF** | **Remove G1/G2 Samples** | | | | **All samples** | | | |
| --- | --- | --- | --- | --- | --- | --- | --- | --- | --- | --- | --- | --- | --- |
|  |  |  |  |  |  | **OR** | **95% CI** | **P-value** | | **OR** | **95% CI** | **P-value** | |
| rs2780902 | 1p31.3 | 65329100 | A | 0.54 | 0.59 | 0.52 | 0.40-0.66 | 2.98E-07 | ^d^ | 0.78 | 0.69-0.88 | 1.01E-04 |  |
| rs1249910 | 3q13.2 | 112391174 | T | 0.60 | 0.65 | 0.49 | 0.36-0.65 | 9.29E-07 | ^d^ | 0.49 | 0.38-0.63 | 7.28E-08 | ^d^ |
| rs1945380 | 11q14.1 | 80418965 | C | 0.46 | 0.50 | 0.72 | 0.63-0.82 | 2.39E-06 |  | 0.73 | 0.64-0.82 | 5.37E-07 |  |
| rs1396626 | 1p21.3 | 96025546 | A | 0.45 | 0.49 | 0.58 | 0.46-0.73 | 2.65E-06 | ^r^ | 0.79 | 0.70-0.89 | 1.20E-04 |  |
| rs4598084 | 6q16.1 | 96530099 | A | 0.52 | 0.56 | 0.60 | 0.49-0.75 | 3.30E-06 | ^r^ | 0.69 | 0.57-0.83 | 1.36E-04 | ^r^ |
| rs6962291 | 7q34 | 139671117 | T | 0.35 | 0.40 | 0.72 | 0.62-0.83 | 5.23E-06 |  | 0.76 | 0.67-0.86 | 2.01E-05 |  |
| rs2596230 | 15q14 | 33720726 | G | 0.17 | 0.14 | 1.56 | 1.28-1.89 | 9.39E-06 |  | 1.45 | 1.22-1.73 | 2.48E-05 |  |
| rs8020941 | 14q32.2 | 97533362 | T | 0.20 | 0.22 | 0.64 | 0.52-0.78 | 9.44E-06 | ^d^ | 0.68 | 0.57-0.81 | 1.84E-05 | ^d^ |
| rs7972171 | 12q13.2 | 55444946 | C | 0.06 | 0.09 | 0.55 | 0.42-0.72 | 1.11E-05 |  | 0.55 | 0.44-0.70 | 1.17E-06 |  |
| rs6943931 | 7p15.3 | 22414646 | C | 0.24 | 0.28 | 0.65 | 0.54-0.79 | 1.31E-05 | ^d^ | 0.67 | 0.56-0.80 | 6.84E-06 | ^d^ |
| rs12110644 | 6p24.3 | 10220826 | G | 0.15 | 0.18 | 0.63 | 0.51-0.78 | 1.34E-05 | ^d^ | 0.75 | 0.64-0.88 | 5.11E-04 |  |
| rs7715214 | 5q34 | 160626657 | T | 0.09 | 0.13 | 0.59 | 0.47-0.75 | 1.43E-05 | ^d^ | 0.61 | 0.50-0.76 | 5.71E-06 | ^d^ |
| rs1252269 | 12q14.3 | 67262772 | A | 0.66 | 0.62 | 1.55 | 1.27-1.88 | 1.45E-05 | ^r^ | 1.40 | 1.17-1.67 | 2.30E-04 | ^r^ |
| rs13395141 | 2q23.1 | 148955901 | T | 0.25 | 0.29 | 0.65 | 0.54-0.79 | 1.68E-05 | ^d^ | 0.67 | 0.56-0.80 | 7.14E-06 | ^d^ |
| rs903552 | 15q26.3 | 101997991 | T | 0.39 | 0.43 | 0.64 | 0.52-0.79 | 1.78E-05 | ^d^ | 0.71 | 0.59-0.85 | 2.53E-04 | ^d^ |
| rs10258404 | 7q31.32 | 122664695 | G | 0.13 | 0.10 | 1.70 | 1.33-2.16 | 1.84E-05 | ^d^ | 1.57 | 1.27-1.95 | 4.13E-05 | ^d^ |
| rs12636302 | 3q26.1 | 162009921 | C | 0.40 | 0.35 | 1.36 | 1.18-1.57 | 1.91E-05 |  | 1.30 | 1.14-1.48 | 5.88E-05 |  |
| rs12489378 | 3p14.2 | 63298608 | A | 0.45 | 0.38 | 1.35 | 1.18-1.55 | 2.23E-05 |  | 1.25 | 1.10-1.42 | 4.20E-04 |  |
| rs6501053 | 16p13.2 | 8062273 | G | 0.15 | 0.19 | 0.68 | 0.57-0.81 | 2.27E-05 |  | 0.72 | 0.61-0.84 | 4.34E-05 |  |
| rs2276549 | 2p23.2 | 29541104 | G | 0.81 | 0.76 | 1.54 | 1.26-1.89 | 2.30E-05 | ^r^ | 1.37 | 1.14-1.64 | 6.38E-04 | ^r^ |
| rs9653004 | 18q12.2 | 35537135 | C | 0.43 | 0.46 | 0.75 | 0.65-0.86 | 2.32E-05 |  | 0.81 | 0.72-0.92 | 7.18E-04 |  |
| rs9510795 | 13q12.12 | 24216012 | A | 0.65 | 0.61 | 1.53 | 1.26-1.86 | 2.36E-05 | ^r^ | 1.51 | 1.27-1.81 | 4.59E-06 | ^r^ |
| rs7767855 | 6p24.3 | 10239788 | T | 0.46 | 0.42 | 1.34 | 1.17-1.54 | 2.68E-05 |  | 1.30 | 1.15-1.47 | 3.74E-05 |  |
| rs12110526 | 6p24.3 | 10211357 | A | 0.07 | 0.09 | 0.57 | 0.43-0.74 | 2.90E-05 | ^d^ | 0.72 | 0.57-0.92 | 7.13E-03 | ^d^ |
| rs3087253 | 3p21.31 | 46418689 | C | 0.19 | 0.23 | 0.65 | 0.53-0.80 | 2.91E-05 | ^d^ | 0.73 | 0.61-0.88 | 7.43E-04 | ^d^ |
| rs9648284 | 7p15.3 | 22417214 | T | 0.25 | 0.29 | 0.67 | 0.55-0.81 | 3.27E-05 | ^d^ | 0.69 | 0.58-0.82 | 2.27E-05 | ^d^ |
| rs1382334 | 5q34 | 160652367 | A | 0.09 | 0.13 | 0.61 | 0.48-0.77 | 3.31E-05 | ^d^ | 0.63 | 0.51-0.77 | 1.22E-05 | ^d^ |
| rs13083543 | 3p13 | 71997483 | T | 0.12 | 0.15 | 0.63 | 0.50-0.78 | 3.35E-05 | ^d^ | 0.69 | 0.57-0.84 | 2.81E-04 | ^d^ |
| rs1069742 | 12q21.33 | 90838693 | G | 0.14 | 0.17 | 0.63 | 0.51-0.78 | 3.35E-05 | ^d^ | 0.75 | 0.61-0.91 | 3.65E-03 | ^d^ |
| rs1507435 | 3q12.3 | 100921878 | G | 0.52 | 0.55 | 0.64 | 0.52-0.79 | 3.62E-05 | ^r^ | 0.74 | 0.61-0.89 | 1.60E-03 | ^r^ |
| rs6011478 | 20q13.33 | 61556771 | G | 0.24 | 0.28 | 0.67 | 0.55-0.81 | 3.69E-05 | ^d^ | 0.75 | 0.65-0.86 | 6.17E-05 |  |
| rs11208534 | 1p31.3 | 65343066 | G | 0.20 | 0.24 | 0.71 | 0.60-0.83 | 3.70E-05 |  | 0.80 | 0.69-0.92 | 2.34E-03 |  |
| rs7997640 | 13q12.3 | 30744080 | G | 0.43 | 0.45 | 0.64 | 0.52-0.79 | 3.73E-05 | ^d^ | 0.68 | 0.57-0.83 | 7.88E-05 | ^d^ |
| rs2722546 | 3q22.3 | 136902177 | G | 0.25 | 0.29 | 0.73 | 0.63-0.85 | 3.75E-05 |  | 0.78 | 0.68-0.89 | 3.53E-04 |  |
| rs310236 | 1p31.3 | 65333857 | G | 0.50 | 0.45 | 1.63 | 1.29-2.05 | 3.86E-05 | ^r^ | 1.23 | 1.08-1.39 | 1.20E-03 |  |
| rs13128580 | 4p15.2 | 25772477 | T | 0.26 | 0.27 | 0.44 | 0.29-0.65 | 3.86E-05 | ^r^ | 0.53 | 0.38-0.75 | 2.64E-04 | ^r^ |
| rs16838616 | 1p36.32 | 4368304 | T | 0.17 | 0.21 | 0.70 | 0.59-0.83 | 3.90E-05 |  | 0.71 | 0.61-0.83 | 1.07E-05 |  |
| rs1979026 | 15q25.1 | 80738546 | G | 0.48 | 0.44 | 1.33 | 1.16-1.52 | 3.92E-05 |  | 1.27 | 1.12-1.43 | 1.38E-04 |  |
| rs1452144 | 3q22.1 | 132627628 | T | 0.32 | 0.35 | 0.66 | 0.55-0.81 | 4.15E-05 | ^d^ | 0.69 | 0.58-0.83 | 5.01E-05 | ^d^ |
| rs7572957 | 2p23.2 | 29487405 | C | 0.35 | 0.31 | 1.35 | 1.17-1.57 | 4.18E-05 |  | 1.40 | 1.18-1.67 | 1.43E-04 | ^d^ |
| rs7338328 | 13q12.12 | 24159525 | A | 0.29 | 0.26 | 1.38 | 1.18-1.62 | 4.23E-05 |  | 1.22 | 1.07-1.41 | 4.14E-03 |  |
| rs1648161 | 11q22.3 | 109919350 | T | 0.13 | 0.11 | 1.56 | 1.26-1.93 | 4.24E-05 |  | 1.39 | 1.15-1.69 | 8.21E-04 |  |
| rs6011473 | 20q13.33 | 61551306 | C | 0.24 | 0.28 | 0.67 | 0.55-0.81 | 4.26E-05 | ^d^ | 0.76 | 0.66-0.87 | 1.00E-04 |  |
| rs7948119 | 11p15.1 | 20650119 | T | 0.09 | 0.06 | 1.83 | 1.37-2.44 | 4.43E-05 | ^d^ | 1.56 | 1.21-2.01 | 7.00E-04 | ^d^ |
| rs10837600 | 11p12 | 41263119 | T | 0.39 | 0.43 | 0.66 | 0.53-0.80 | 4.45E-05 | ^d^ | 0.71 | 0.59-0.85 | 2.06E-04 | ^d^ |
| rs6494387 | 15q22.2 | 63345526 | A | 0.37 | 0.31 | 1.35 | 1.17-1.56 | 4.51E-05 |  | 1.25 | 1.09-1.42 | 8.80E-04 |  |
| rs4878640 | 9p13.3 | 36126723 | T | 0.42 | 0.37 | 1.51 | 1.24-1.84 | 4.60E-05 | ^d^ | 1.45 | 1.22-1.74 | 4.04E-05 | ^d^ |
| rs1024603 | 14q24.3 | 74295380 | T | 0.24 | 0.27 | 0.67 | 0.55-0.81 | 4.73E-05 | ^d^ | 0.73 | 0.61-0.86 | 2.86E-04 | ^d^ |
| rs1328183 | 1q31.2 | 193759244 | A | 0.19 | 0.22 | 0.66 | 0.54-0.81 | 4.75E-05 | ^d^ | 0.68 | 0.57-0.82 | 3.37E-05 | ^d^ |
| rs4869233 | 5q15 | 94425542 | G | 0.40 | 0.41 | 0.59 | 0.45-0.76 | 4.81E-05 | ^r^ | 0.86 | 0.76-0.98 | 2.23E-02 |  |
| rs11071505 | 15q22.2 | 60471417 | G | 0.27 | 0.22 | 1.50 | 1.23-1.83 | 4.81E-05 | ^d^ | 1.30 | 1.12-1.50 | 4.32E-04 |  |
| rs1589921 | 15q11.2 | 23750045 | A | 0.10 | 0.08 | 1.65 | 1.30-2.10 | 4.85E-05 |  | 1.54 | 1.23-1.92 | 1.31E-04 |  |
| rs2970199 | 1q41 | 222177454 | A | 0.21 | 0.17 | 1.45 | 1.21-1.73 | 4.85E-05 |  | 1.32 | 1.13-1.55 | 6.01E-04 |  |
| rs3737744 | 1p33 | 46810530 | C | 0.19 | 0.16 | 1.53 | 1.25-1.88 | 5.04E-05 | ^d^ | 1.36 | 1.13-1.64 | 1.25E-03 | ^d^ |
| rs1450397 | 4q32.2 | 163274065 | A | 0.14 | 0.11 | 1.53 | 1.25-1.88 | 5.05E-05 |  | 1.43 | 1.19-1.73 | 1.50E-04 |  |
| rs2095270 | 13q33.1 | 101900361 | A | 0.23 | 0.19 | 1.51 | 1.24-1.84 | 5.09E-05 | ^d^ | 1.45 | 1.21-1.73 | 5.96E-05 | ^d^ |
| rs6718491 | 2p12 | 81287257 | C | 0.46 | 0.42 | 1.33 | 1.16-1.53 | 5.11E-05 |  | 1.27 | 1.12-1.44 | 2.21E-04 |  |
| rs2956224 | 8p21.2 | 25455401 | G | 0.18 | 0.14 | 1.47 | 1.22-1.78 | 5.12E-05 |  | 1.41 | 1.19-1.66 | 7.37E-05 |  |
| rs13347176 | 20q13.33 | 61532610 | G | 0.24 | 0.28 | 0.67 | 0.55-0.81 | 5.14E-05 | ^d^ | 0.76 | 0.67-0.88 | 1.45E-04 |  |
| rs153735 | 3p14.1 | 64162544 | T | 0.21 | 0.24 | 0.72 | 0.61-0.84 | 5.19E-05 |  | 0.78 | 0.65-0.93 | 5.43E-03 | ^d^ |
| rs2702996 | 8q24.22 | 134055485 | A | 0.09 | 0.11 | 0.61 | 0.48-0.77 | 5.25E-05 | ^d^ | 0.62 | 0.50-0.77 | 1.99E-05 | ^d^ |
| rs7335175 | 13q33.1 | 103488664 | G | 0.09 | 0.06 | 1.72 | 1.32-2.25 | 5.38E-05 |  | 1.44 | 1.14-1.82 | 2.54E-03 |  |
| rs10200680 | 2q36.1 | 223961877 | A | 0.22 | 0.19 | 1.51 | 1.24-1.85 | 5.43E-05 | ^d^ | 1.31 | 1.09-1.57 | 3.97E-03 | ^d^ |
| rs12219473 | 10p12.2 | 24564139 | T | 0.09 | 0.07 | 1.78 | 1.34-2.35 | 5.45E-05 | ^d^ | 1.41 | 1.10-1.80 | 6.68E-03 | ^d^ |
| rs1556990 | 10p13 | 13550822 | A | 0.06 | 0.08 | 0.55 | 0.41-0.74 | 5.61E-05 | ^d^ | 0.55 | 0.43-0.72 | 1.17E-05 | ^d^ |
| rs11971428 | 7q34 | 139679245 | A | 0.48 | 0.44 | 1.33 | 1.16-1.52 | 5.64E-05 |  | 1.26 | 1.12-1.43 | 2.12E-04 |  |
| rs1362602 | 12p12.3 | 14838469 | T | 0.56 | 0.51 | 1.61 | 1.28-2.02 | 5.67E-05 | ^d^ | 1.53 | 1.25-1.89 | 5.36E-05 | ^d^ |
| rs998422 | 20q13.12 | 45359408 | G | 0.18 | 0.14 | 1.48 | 1.22-1.80 | 5.68E-05 |  | 1.34 | 1.13-1.60 | 9.67E-04 |  |
| rs3915922 | 1p31.3 | 65368562 | G | 0.39 | 0.43 | 0.75 | 0.65-0.86 | 5.85E-05 |  | 0.77 | 0.68-0.87 | 5.03E-05 |  |
| rs16873932 | 8q23.1 | 106918465 | C | 0.34 | 0.36 | 0.55 | 0.41-0.74 | 5.94E-05 | ^r^ | 0.67 | 0.52-0.87 | 2.96E-03 | ^r^ |
| rs7214746 | 17q11.2 | 27537626 | C | 0.09 | 0.06 | 1.70 | 1.31-2.20 | 5.99E-05 |  | 1.59 | 1.26-2.01 | 9.24E-05 |  |
| rs2600956 | 3q22.1 | 132624113 | T | 0.32 | 0.34 | 0.67 | 0.55-0.82 | 6.20E-05 | ^d^ | 0.71 | 0.59-0.84 | 1.05E-04 | ^d^ |
| rs11089653 | 22q12.3 | 34698979 | T | 0.18 | 0.19 | 0.35 | 0.21-0.58 | 6.22E-05 | ^r^ | 0.46 | 0.29-0.72 | 6.71E-04 | ^r^ |
| rs1113643 | 2p23.2 | 29539536 | A | 0.81 | 0.76 | 1.51 | 1.23-1.85 | 6.41E-05 | ^r^ | 1.28 | 1.10-1.49 | 1.46E-03 |  |
| rs310196 | 1p31.3 | 65351622 | G | 0.39 | 0.43 | 0.75 | 0.65-0.86 | 6.44E-05 |  | 0.77 | 0.68-0.87 | 5.13E-05 |  |
| rs10840257 | 11p15.4 | 9568463 | C | 0.45 | 0.39 | 1.33 | 1.16-1.53 | 6.59E-05 |  | 1.33 | 1.17-1.51 | 1.02E-05 |  |
| rs10828859 | 10p12.31 | 18822342 | C | 0.22 | 0.25 | 0.67 | 0.55-0.82 | 6.62E-05 | ^d^ | 0.73 | 0.61-0.87 | 4.55E-04 | ^d^ |
| rs2374731 | 12q23.3 | 108285600 | A | 0.27 | 0.30 | 0.68 | 0.56-0.82 | 6.73E-05 | ^d^ | 0.72 | 0.60-0.85 | 1.82E-04 | ^d^ |
| rs7850246 | 9p21.3 | 20645480 | G | 0.15 | 0.11 | 1.58 | 1.26-1.98 | 6.75E-05 | ^d^ | 1.52 | 1.24-1.87 | 5.62E-05 | ^d^ |
| rs9311863 | 3p14.2 | 63259509 | T | 0.37 | 0.32 | 1.34 | 1.16-1.55 | 6.95E-05 |  | 1.27 | 1.12-1.45 | 2.50E-04 |  |
| rs6736816 | 2p16.3 | 50255589 | C | 0.17 | 0.22 | 0.67 | 0.54-0.81 | 7.07E-05 | ^d^ | 0.70 | 0.58-0.83 | 8.00E-05 | ^d^ |
| rs17654300 | 19p13.11 | 17788648 | G | 0.09 | 0.12 | 0.61 | 0.48-0.78 | 7.10E-05 | ^d^ | 0.64 | 0.52-0.79 | 2.17E-05 |  |
| rs11130920 | 3p14.2 | 63285738 | A | 0.44 | 0.49 | 0.76 | 0.66-0.87 | 7.11E-05 |  | 0.80 | 0.71-0.90 | 2.46E-04 |  |
| rs1541807 | 16p13.12 | 12791629 | C | 0.28 | 0.31 | 0.68 | 0.56-0.82 | 7.16E-05 | ^d^ | 0.78 | 0.68-0.90 | 4.61E-04 |  |
| rs864553 | 1p21.3 | 95378425 | G | 0.22 | 0.19 | 1.50 | 1.23-1.84 | 7.21E-05 | ^d^ | 1.48 | 1.23-1.77 | 2.56E-05 | ^d^ |
| rs327215 | 8p21.2 | 26492962 | C | 0.14 | 0.10 | 1.52 | 1.24-1.87 | 7.25E-05 |  | 1.38 | 1.14-1.66 | 7.69E-04 |  |
| rs17015624 | 3p12.3 | 77959159 | G | 0.20 | 0.16 | 1.52 | 1.24-1.87 | 7.25E-05 | ^d^ | 1.40 | 1.16-1.69 | 3.78E-04 | ^d^ |
| rs4118071 | 11q14.1 | 80425472 | A | 0.49 | 0.52 | 0.76 | 0.66-0.87 | 7.26E-05 |  | 0.76 | 0.68-0.86 | 1.53E-05 |  |
| rs10475999 | 5q35.1 | 171486904 | A | 0.28 | 0.31 | 0.68 | 0.56-0.82 | 7.32E-05 | ^d^ | 0.72 | 0.60-0.85 | 1.72E-04 | ^d^ |
| rs1970728 | 11q14.1 | 80394267 | C | 0.36 | 0.40 | 0.76 | 0.66-0.87 | 7.36E-05 |  | 0.77 | 0.68-0.87 | 3.63E-05 |  |
| rs6441441 | 3q26.1 | 162003027 | G | 0.35 | 0.30 | 1.34 | 1.16-1.55 | 7.38E-05 |  | 1.26 | 1.11-1.44 | 5.58E-04 |  |
| rs1569079 | 7q11.22 | 70224743 | G | 0.07 | 0.06 | 1.81 | 1.35-2.42 | 7.53E-05 |  | 1.68 | 1.29-2.19 | 1.30E-04 |  |
| rs16892547 | 6q26 | 161886037 | G | 0.65 | 0.60 | 1.49 | 1.22-1.82 | 7.63E-05 | ^r^ | 1.33 | 1.11-1.59 | 1.71E-03 | ^r^ |
| rs4748042 | 10p13 | 13554693 | G | 0.05 | 0.07 | 0.56 | 0.42-0.74 | 7.81E-05 | ^d^ | 0.56 | 0.43-0.73 | 1.68E-05 | ^d^ |
| rs932403 | 6p24.3 | 9520993 | A | 0.34 | 0.37 | 0.75 | 0.65-0.87 | 7.82E-05 |  | 0.79 | 0.70-0.90 | 3.39E-04 |  |
| rs6011460 | 20q13.33 | 61532837 | C | 0.24 | 0.28 | 0.68 | 0.56-0.82 | 7.88E-05 | ^d^ | 0.77 | 0.67-0.89 | 2.71E-04 |  |
| rs9642825 | 8q23.3 | 115487251 | A | 0.26 | 0.23 | 1.38 | 1.17-1.61 | 7.96E-05 |  | 1.31 | 1.14-1.52 | 1.79E-04 |  |
| rs2055438 | 5q14.2 | 81962788 | A | 0.38 | 0.40 | 0.59 | 0.45-0.76 | 8.01E-05 | ^r^ | 0.75 | 0.59-0.95 | 1.68E-02 | ^r^ |
| rs1494347 | 9p22.3 | 14790488 | C | 0.07 | 0.09 | 0.59 | 0.45-0.77 | 8.07E-05 | ^d^ | 0.67 | 0.53-0.84 | 6.27E-04 |  |
| rs9883161 | 3q26.1 | 162004273 | G | 0.35 | 0.30 | 1.34 | 1.16-1.55 | 8.15E-05 |  | 1.26 | 1.11-1.44 | 5.32E-04 |  |
| rs4748043 | 10p13 | 13556487 | A | 0.06 | 0.07 | 0.56 | 0.42-0.75 | 8.19E-05 | ^d^ | 0.56 | 0.43-0.73 | 1.82E-05 | ^d^ |
| rs8066684 | 17q24.3 | 69379799 | C | 0.35 | 0.40 | 0.76 | 0.66-0.87 | 8.21E-05 |  | 0.82 | 0.73-0.93 | 2.39E-03 |  |
| rs1435128 | 4q31.21 | 142159475 | T | 0.12 | 0.10 | 1.64 | 1.28-2.10 | 8.23E-05 | ^d^ | 1.55 | 1.24-1.95 | 1.54E-04 | ^d^ |
| rs11690874 | 2q35 | 220877631 | T | 0.48 | 0.52 | 0.76 | 0.66-0.87 | 8.53E-05 |  | 0.79 | 0.70-0.89 | 1.80E-04 |  |
| rs11664246 | 18q12.2 | 35535315 | T | 0.42 | 0.46 | 0.76 | 0.67-0.87 | 8.53E-05 |  | 0.82 | 0.73-0.93 | 1.63E-03 |  |
| rs1374539 | 11p12 | 41342481 | G | 0.33 | 0.37 | 0.76 | 0.66-0.87 | 8.68E-05 |  | 0.82 | 0.72-0.93 | 1.67E-03 |  |
| rs2424503 | 20p11.21 | 23035104 | G | 0.69 | 0.72 | 0.74 | 0.64-0.86 | 8.77E-05 |  | 0.79 | 0.69-0.90 | 6.04E-04 |  |
| rs442432 | 6q27 | 169474908 | C | 0.10 | 0.08 | 1.60 | 1.27-2.03 | 8.80E-05 |  | 1.65 | 1.33-2.04 | 6.15E-06 |  |
| rs4733781 | 8q24.21 | 131296767 | C | 0.16 | 0.16 | 4.87 | 2.20-10.75 | 9.14E-05 | ^r^ | 3.85 | 1.94-7.66 | 1.22E-04 | ^r^ |
| rs12508442 | 4q13.2 | 68106430 | T | 0.19 | 0.16 | 1.51 | 1.23-1.86 | 9.23E-05 | ^d^ | 1.41 | 1.17-1.71 | 3.36E-04 | ^d^ |
| rs17686792 | 18q12.3 | 41613422 | G | 0.07 | 0.06 | 1.74 | 1.32-2.30 | 9.24E-05 |  | 1.61 | 1.25-2.07 | 2.10E-04 |  |
| rs12198826 | 6q27 | 169499546 | A | 0.11 | 0.08 | 1.61 | 1.27-2.05 | 9.26E-05 |  | 1.48 | 1.19-1.85 | 3.69E-04 |  |
| rs7232163 | 18p11.22 | 8553982 | T | 0.46 | 0.51 | 0.64 | 0.51-0.80 | 9.32E-05 | ^r^ | 0.68 | 0.55-0.83 | 1.70E-04 | ^r^ |
| rs7079700 | 10q11.21 | 45478681 | G | 0.19 | 0.17 | 1.44 | 1.20-1.73 | 9.34E-05 |  | 1.35 | 1.14-1.59 | 3.99E-04 |  |
| rs878838 | 12p11.22 | 29620687 | T | 0.10 | 0.07 | 1.64 | 1.28-2.10 | 9.35E-05 |  | 1.61 | 1.29-2.01 | 2.84E-05 |  |
| rs7228775 | 18q22.1 | 61681014 | G | 0.52 | 0.48 | 1.56 | 1.25-1.95 | 9.46E-05 | ^r^ | 1.45 | 1.19-1.78 | 2.59E-04 | ^r^ |
| rs17129320 | 14q32.12 | 94364512 | C | 0.20 | 0.23 | 0.68 | 0.55-0.82 | 9.48E-05 | ^d^ | 0.73 | 0.61-0.87 | 5.77E-04 | ^d^ |
| rs4691996 | 4q32.3 | 165496866 | A | 0.45 | 0.48 | 0.65 | 0.53-0.81 | 9.59E-05 | ^d^ | 0.74 | 0.61-0.90 | 2.87E-03 | ^d^ |
| rs1416043 | 1p33 | 48863365 | T | 0.14 | 0.17 | 0.69 | 0.57-0.83 | 9.63E-05 |  | 0.75 | 0.63-0.88 | 6.26E-04 |  |
| rs219474 | 4q25 | 109313182 | C | 0.62 | 0.59 | 1.49 | 1.22-1.81 | 9.64E-05 | ^r^ | 1.46 | 1.22-1.75 | 4.39E-05 | ^r^ |
| rs6058434 | 20q11.23 | 34795372 | T | 0.43 | 0.44 | 0.62 | 0.48-0.79 | 9.65E-05 | ^r^ | 0.62 | 0.50-0.78 | 2.41E-05 | ^r^ |
| rs4355350 | 4q32.2 | 163259741 | C | 0.27 | 0.23 | 1.36 | 1.17-1.59 | 9.69E-05 |  | 1.30 | 1.13-1.50 | 2.12E-04 |  |
| rs31546 | 5q31.1 | 135277503 | C | 0.32 | 0.37 | 0.75 | 0.65-0.87 | 9.84E-05 |  | 0.71 | 0.59-0.84 | 1.22E-04 | ^d^ |
| rs9936891 | 16p13.2 | 7906045 | T | 0.41 | 0.38 | 1.48 | 1.22-1.81 | 9.85E-05 | ^d^ | 1.48 | 1.23-1.77 | 2.01E-05 | ^d^ |
| rs7117559 | 11p15.4 | 4752136 | A | 0.23 | 0.27 | 0.74 | 0.63-0.86 | 9.89E-05 |  | 0.73 | 0.61-0.87 | 4.01E-04 | ^d^ |
| rs7772958 | 6q27 | 166632497 | T | 0.17 | 0.20 | 0.67 | 0.55-0.82 | 9.93E-05 | ^d^ | 0.73 | 0.61-0.88 | 7.87E-04 | ^d^ |
| rs1460753 | 4q21.3 | 87223468 | C | 0.57 | 0.52 | 1.31 | 1.14-1.50 | 1.00E-04 |  | 1.31 | 1.16-1.48 | 1.80E-05 |  |
| rs11084564 | 19q12 | 31321565 | G | 0.55 | 0.50 | 1.31 | 1.14-1.50 | 1.01E-04 |  | 1.23 | 1.09-1.38 | 1.01E-03 |  |
| rs11127018 | 2p11.2 | 86454914 | C | 0.55 | 0.50 | 1.31 | 1.14-1.50 | 1.01E-04 |  | 1.24 | 1.09-1.40 | 6.76E-04 |  |
| rs4942830 | 13q14.2 | 50019376 | A | 0.38 | 0.44 | 0.76 | 0.66-0.87 | 1.01E-04 |  | 0.72 | 0.60-0.86 | 3.33E-04 | ^d^ |
| rs16961674 | 15q21.1 | 49101996 | G | 0.10 | 0.08 | 1.64 | 1.28-2.10 | 1.04E-04 |  | 1.53 | 1.22-1.91 | 2.14E-04 |  |
| rs7923069 | 10q21.1 | 60614297 | A | 0.13 | 0.10 | 1.59 | 1.26-2.01 | 1.06E-04 | ^d^ | 1.40 | 1.14-1.73 | 1.63E-03 | ^d^ |
| rs13354740 | 5p15.33 | 2370988 | T | 0.08 | 0.11 | 0.62 | 0.48-0.79 | 1.10E-04 |  | 0.73 | 0.59-0.91 | 4.09E-03 |  |
| rs280355 | 12q12 | 41404105 | C | 0.33 | 0.29 | 1.46 | 1.21-1.77 | 1.10E-04 | ^d^ | 1.29 | 1.12-1.47 | 2.61E-04 |  |
| rs10837613 | 11p12 | 41299498 | T | 0.37 | 0.41 | 0.76 | 0.66-0.87 | 1.10E-04 |  | 0.73 | 0.61-0.87 | 5.80E-04 | ^d^ |
| rs1534537 | 2q32.3 | 197263186 | C | 0.19 | 0.15 | 1.51 | 1.23-1.86 | 1.11E-04 | ^d^ | 1.51 | 1.25-1.82 | 2.20E-05 | ^d^ |
| rs1553025 | 1p31.3 | 65364794 | C | 0.39 | 0.43 | 0.75 | 0.65-0.87 | 1.11E-04 |  | 0.77 | 0.67-0.88 | 9.85E-05 |  |
| rs10929676 | 2p25.1 | 10717282 | G | 0.38 | 0.34 | 1.47 | 1.21-1.79 | 1.12E-04 | ^d^ | 1.43 | 1.20-1.71 | 7.75E-05 | ^d^ |
| rs12137305 | 1q41 | 222220701 | T | 0.11 | 0.09 | 1.57 | 1.25-1.97 | 1.13E-04 |  | 1.45 | 1.18-1.78 | 4.39E-04 |  |
| rs3130578 | 6p21.32 | 33018310 | T | 0.13 | 0.15 | 0.69 | 0.57-0.83 | 1.14E-04 |  | 0.74 | 0.63-0.88 | 6.40E-04 |  |
| rs12196325 | 6q27 | 166513487 | T | 0.28 | 0.24 | 1.46 | 1.20-1.77 | 1.19E-04 | ^d^ | 1.46 | 1.23-1.74 | 2.13E-05 | ^d^ |
| rs184360 | 4p15.2 | 25362101 | C | 0.43 | 0.46 | 0.66 | 0.54-0.82 | 1.21E-04 | ^d^ | 0.69 | 0.57-0.83 | 1.13E-04 | ^d^ |
| rs9301196 | 13q33.3 | 107847432 | T | 0.07 | 0.11 | 0.62 | 0.49-0.79 | 1.21E-04 |  | 0.66 | 0.53-0.82 | 2.11E-04 |  |
| rs1427992 | 12p12.2 | 20433640 | G | 0.36 | 0.41 | 0.76 | 0.66-0.87 | 1.22E-04 |  | 0.79 | 0.70-0.90 | 3.34E-04 |  |
| rs4077972 | 3p13 | 71996829 | T | 0.06 | 0.08 | 0.58 | 0.44-0.77 | 1.24E-04 | ^d^ | 0.61 | 0.48-0.79 | 1.83E-04 | ^d^ |
| rs10759255 | 9q31.2 | 110497951 | G | 0.32 | 0.37 | 0.76 | 0.66-0.87 | 1.24E-04 |  | 0.70 | 0.59-0.84 | 8.41E-05 | ^d^ |
| rs9563751 | 13q21.2 | 60204504 | T | 0.11 | 0.13 | 0.64 | 0.51-0.80 | 1.25E-04 | ^d^ | 0.68 | 0.55-0.84 | 2.76E-04 | ^d^ |
| rs12187377 | 5q12.1 | 60567241 | G | 0.36 | 0.31 | 1.33 | 1.15-1.54 | 1.25E-04 |  | 1.31 | 1.15-1.50 | 5.48E-05 |  |
| rs17002860 | 4q21.21 | 78918575 | A | 0.05 | 0.07 | 0.57 | 0.43-0.76 | 1.25E-04 | ^d^ | 0.64 | 0.50-0.83 | 7.04E-04 | ^d^ |
| rs304029 | 3p26.1 | 4545824 | G | 0.31 | 0.27 | 1.45 | 1.20-1.76 | 1.25E-04 | ^d^ | 1.41 | 1.19-1.68 | 1.01E-04 | ^d^ |
| rs4426155 | 11q14.1 | 81201287 | A | 0.05 | 0.06 | 0.57 | 0.43-0.76 | 1.25E-04 |  | 0.66 | 0.52-0.85 | 1.45E-03 |  |
| rs6764486 | 3p26.1 | 7083387 | G | 0.28 | 0.30 | 0.69 | 0.57-0.83 | 1.26E-04 | ^d^ | 0.77 | 0.65-0.92 | 3.77E-03 | ^d^ |
| rs594074 | 6p25.1 | 6552994 | A | 0.07 | 0.10 | 0.60 | 0.46-0.78 | 1.29E-04 | ^d^ | 0.64 | 0.51-0.81 | 2.29E-04 | ^d^ |
| rs7016842 | 8p21.2 | 25456297 | A | 0.17 | 0.14 | 1.45 | 1.20-1.75 | 1.29E-04 |  | 1.38 | 1.16-1.63 | 2.22E-04 |  |
| rs1868694 | 4q32.2 | 163271146 | C | 0.14 | 0.12 | 1.49 | 1.22-1.83 | 1.30E-04 |  | 1.40 | 1.16-1.69 | 3.61E-04 |  |
| rs860544 | 14q12 | 25234818 | G | 0.23 | 0.25 | 0.73 | 0.62-0.86 | 1.33E-04 |  | 0.75 | 0.65-0.87 | 1.09E-04 |  |
| rs12354293 | 1p21.3 | 96013073 | A | 0.29 | 0.33 | 0.75 | 0.65-0.87 | 1.33E-04 |  | 0.79 | 0.69-0.89 | 2.83E-04 |  |
| rs2195979 | 19q12 | 31321306 | G | 0.55 | 0.50 | 1.30 | 1.14-1.49 | 1.33E-04 |  | 1.22 | 1.08-1.38 | 1.29E-03 |  |
| rs9839965 | 3q22.3 | 136894133 | C | 0.23 | 0.28 | 0.68 | 0.56-0.83 | 1.35E-04 | ^d^ | 0.76 | 0.64-0.91 | 2.46E-03 | ^d^ |
| rs10090607 | 8q11.23 | 53777472 | G | 0.53 | 0.56 | 0.63 | 0.50-0.80 | 1.35E-04 | ^d^ | 0.82 | 0.72-0.93 | 1.50E-03 |  |
| rs2979740 | 8p21.2 | 25455330 | G | 0.17 | 0.14 | 1.45 | 1.20-1.75 | 1.36E-04 |  | 1.38 | 1.16-1.63 | 2.30E-04 |  |
| rs2224184 | 20p12.2 | 11501142 | C | 0.55 | 0.58 | 0.67 | 0.54-0.82 | 1.38E-04 | ^r^ | 0.70 | 0.58-0.85 | 2.20E-04 | ^r^ |
| rs16908458 | 8q21.13 | 81956892 | G | 0.17 | 0.20 | 0.67 | 0.55-0.83 | 1.40E-04 | ^d^ | 0.70 | 0.59-0.85 | 1.76E-04 | ^d^ |
| rs2630594 | 12p11.21 | 32206697 | C | 0.44 | 0.40 | 1.49 | 1.21-1.82 | 1.40E-04 | ^d^ | 1.40 | 1.17-1.69 | 3.18E-04 | ^d^ |
| rs7310814 | 12q23.3 | 104772623 | A | 0.47 | 0.42 | 1.31 | 1.14-1.50 | 1.40E-04 |  | 1.27 | 1.12-1.44 | 1.50E-04 |  |
| rs11123389 | 2q14.1 | 117411169 | C | 0.55 | 0.51 | 1.31 | 1.14-1.50 | 1.41E-04 |  | 1.19 | 1.05-1.34 | 5.68E-03 |  |
| rs10203946 | 2p16.3 | 52829150 | C | 0.09 | 0.07 | 1.66 | 1.28-2.15 | 1.41E-04 |  | 1.55 | 1.20-1.99 | 7.69E-04 | ^d^ |
| rs17494681 | 1q25.3 | 181480183 | A | 0.10 | 0.07 | 1.63 | 1.27-2.10 | 1.43E-04 |  | 1.54 | 1.22-1.94 | 2.95E-04 |  |
| rs10903640 | 10p15.3 | 2083067 | T | 0.65 | 0.63 | 1.46 | 1.20-1.78 | 1.43E-04 | ^r^ | 1.38 | 1.15-1.64 | 3.81E-04 | ^r^ |
| rs2347572 | 16q23.2 | 79318712 | C | 0.12 | 0.10 | 1.54 | 1.23-1.93 | 1.44E-04 |  | 1.41 | 1.15-1.72 | 9.91E-04 |  |
| rs2117624 | 4q28.3 | 133474143 | G | 0.32 | 0.37 | 0.76 | 0.66-0.88 | 1.46E-04 |  | 0.81 | 0.71-0.92 | 1.01E-03 |  |
| rs4652554 | 1q25.3 | 181032155 | T | 0.38 | 0.32 | 1.32 | 1.14-1.52 | 1.48E-04 |  | 1.28 | 1.13-1.46 | 1.44E-04 |  |
| rs439922 | 4q25 | 107819233 | A | 0.55 | 0.57 | 0.63 | 0.49-0.80 | 1.48E-04 | ^d^ | 0.83 | 0.73-0.94 | 2.48E-03 |  |
| rs7281104 | 21q11.2 | 15918577 | G | 0.39 | 0.34 | 1.32 | 1.14-1.52 | 1.49E-04 |  | 1.30 | 1.14-1.47 | 8.91E-05 |  |
| rs4849507 | 2q14.1 | 117410623 | T | 0.55 | 0.50 | 1.31 | 1.14-1.50 | 1.50E-04 |  | 1.19 | 1.05-1.34 | 5.83E-03 |  |
| rs10516657 | 4q21.22 | 82698105 | T | 0.44 | 0.49 | 0.63 | 0.50-0.80 | 1.52E-04 | ^r^ | 0.83 | 0.73-0.94 | 3.10E-03 |  |
| rs4868814 | 5q34 | 163045735 | T | 0.28 | 0.32 | 0.75 | 0.65-0.87 | 1.53E-04 |  | 0.80 | 0.70-0.91 | 8.53E-04 |  |
| rs11037969 | 11p11.2 | 44389241 | C | 0.11 | 0.14 | 0.64 | 0.51-0.81 | 1.53E-04 | ^d^ | 0.69 | 0.57-0.85 | 4.35E-04 | ^d^ |
| rs17141842 | 11q14.1 | 80978480 | T | 0.13 | 0.16 | 0.66 | 0.53-0.82 | 1.53E-04 | ^d^ | 0.68 | 0.56-0.82 | 8.53E-05 | ^d^ |
| rs815807 | 2p21 | 47381245 | G | 0.27 | 0.22 | 1.36 | 1.16-1.59 | 1.56E-04 |  | 1.26 | 1.09-1.45 | 1.83E-03 |  |
| rs10807627 | 6p22.3 | 18681219 | A | 0.49 | 0.45 | 1.50 | 1.22-1.85 | 1.56E-04 | ^d^ | 1.19 | 1.05-1.34 | 6.55E-03 |  |
| rs7031666 | 9q32 | 116423629 | T | 0.28 | 0.31 | 0.69 | 0.57-0.84 | 1.57E-04 | ^d^ | 0.73 | 0.61-0.87 | 3.44E-04 | ^d^ |
| rs490049 | 13q13.1 | 33564863 | A | 0.12 | 0.15 | 0.65 | 0.52-0.81 | 1.58E-04 | ^d^ | 0.71 | 0.58-0.87 | 9.26E-04 | ^d^ |
| rs1998478 | 13q12.12 | 24721774 | C | 0.63 | 0.58 | 1.47 | 1.20-1.79 | 1.58E-04 | ^r^ | 1.32 | 1.10-1.57 | 2.74E-03 | ^r^ |
| rs711633 | 3p26.1 | 4491261 | G | 0.28 | 0.24 | 1.35 | 1.15-1.57 | 1.58E-04 |  | 1.32 | 1.15-1.52 | 9.24E-05 |  |
| rs6691652 | 1q31.2 | 193717698 | G | 0.13 | 0.16 | 0.68 | 0.55-0.83 | 1.59E-04 |  | 0.71 | 0.58-0.86 | 6.47E-04 | ^d^ |
| rs7334243 | 13q12.12 | 24639175 | G | 0.29 | 0.27 | 2.00 | 1.40-2.87 | 1.59E-04 | ^r^ | 1.13 | 0.98-1.29 | 9.07E-02 |  |
| rs1466533 | 6q24.2 | 143860599 | A | 0.36 | 0.32 | 1.45 | 1.20-1.76 | 1.60E-04 | ^d^ | 1.32 | 1.11-1.57 | 1.72E-03 | ^d^ |
| rs2531149 | 4p15.32 | 15914006 | T | 0.48 | 0.47 | 1.51 | 1.22-1.86 | 1.60E-04 | ^d^ | 1.47 | 1.21-1.78 | 9.78E-05 | ^d^ |
| rs12480895 | 20q13.12 | 43069126 | G | 0.24 | 0.28 | 0.69 | 0.57-0.84 | 1.61E-04 | ^d^ | 0.81 | 0.71-0.93 | 2.61E-03 |  |
| rs4282823 | 1p36.22 | 11216665 | T | 0.22 | 0.18 | 1.48 | 1.21-1.81 | 1.62E-04 | ^d^ | 1.32 | 1.10-1.58 | 2.68E-03 | ^d^ |
| rs6801601 | 3p14.1 | 65754783 | A | 0.50 | 0.47 | 1.30 | 1.14-1.50 | 1.62E-04 |  | 1.28 | 1.13-1.45 | 9.43E-05 |  |
| rs1970730 | 11q14.1 | 80393468 | T | 0.47 | 0.49 | 0.77 | 0.67-0.88 | 1.64E-04 |  | 0.79 | 0.69-0.89 | 1.33E-04 |  |
| rs7939459 | 11p15.4 | 5208393 | T | 0.54 | 0.49 | 1.31 | 1.14-1.50 | 1.65E-04 |  | 1.26 | 1.12-1.43 | 2.48E-04 |  |
| rs2922037 | 11p12 | 41309275 | C | 0.33 | 0.36 | 0.76 | 0.66-0.88 | 1.66E-04 |  | 0.82 | 0.72-0.93 | 2.70E-03 |  |
| rs7234360 | 18q22.1 | 61681464 | G | 0.52 | 0.50 | 1.53 | 1.23-1.91 | 1.67E-04 | ^r^ | 1.43 | 1.17-1.75 | 4.06E-04 | ^r^ |
| rs12992354 | 2p23.3 | 25341838 | C | 0.07 | 0.10 | 0.60 | 0.46-0.78 | 1.68E-04 | ^d^ | 0.72 | 0.57-0.92 | 7.06E-03 | ^d^ |
| rs6547973 | 2p23.2 | 29969390 | G | 0.37 | 0.31 | 1.32 | 1.14-1.53 | 1.68E-04 |  | 1.40 | 1.18-1.67 | 1.70E-04 | ^d^ |
| rs2221220 | 8q22.1 | 96533330 | T | 0.22 | 0.26 | 0.74 | 0.63-0.87 | 1.69E-04 |  | 0.77 | 0.67-0.88 | 2.44E-04 |  |
| rs12119864 | 1p36.32 | 5022767 | G | 0.08 | 0.10 | 0.62 | 0.48-0.79 | 1.70E-04 | ^d^ | 0.62 | 0.49-0.77 | 2.86E-05 | ^d^ |

Direction: RA is reference allele. The odds ratio (OR) is presented for the reference allele, compared with the non-reference allele, for a given model.
