# Supplementary material for: Genome-Wide Association and Trans-ethnic Meta-Analysis for Advanced Diabetic Kidney Disease: Family Investigation of Nephropathy and Diabetes (FIND)
Source: PLoS Genet. 2015 Aug 25;11(8):e1005352. doi: 10.1371/journal.pgen.1005352 (PMC4549309; doi:10.1371/journal.pgen.1005352)
Supplement: S8 Table — (DOCX) [file pgen.1005352.s009.docx]

**Supplemental Table S8:**

**Top 200 associations from the FIND Discovery GWAS Meta-analysis, excluding African American subjects with 2 copies of APOL1 G1 and/or G2risk variants**

| **SNP** | **Cytoband** | **Position** | **RA** | **OR** | **CI** | **P-value** | | **Direction** | **Heterogeneity** |
| --- | --- | --- | --- | --- | --- | --- | --- | --- | --- |
| rs903552 | 15q26.3 | 101997991 | T | 0.74 | 0.65-0.85 | 1.68E-06 | ^d^ | ---- | 0.3114 |
| rs490049 | 13q13.1 | 33564863 | A | 0.50 | 0.34-0.73 | 1.75E-06 | ^d^ | ---- | 0.6980 |
| rs17373728 | 8q21.11 | 76225516 | C | 1.34 | 1.19-1.52 | 2.13E-06 |  | ?+++ | 0.7624 |
| rs1605939 | 8q21.11 | 76070707 | A | 1.59 | 1.29-1.96 | 2.67E-06 | ^r^ | ?+++ | 0.4926 |
| rs12531478 | 7p21.2 | 15239894 | A | 2.24 | 1.59-3.15 | 3.96E-06 |  | ??+? | 1.0000 |
| rs13120432 | 4q22.3 | 96943038 | A | 0.65 | 0.52-0.80 | 4.04E-06 | ^d^ | ---- | 0.9453 |
| rs594074 | 6p25.1 | 6552994 | A | 0.71 | 0.61-0.82 | 4.84E-06 | ^d^ | ---- | 0.4869 |
| rs10911184 | 1q25.3 | 182965667 | A | 0.81 | 0.74-0.90 | 4.86E-06 |  | ---- | 0.6560 |
| rs6927188 | 6p21.1 | 42912589 | C | 0.74 | 0.65-0.85 | 5.72E-06 |  | -?-- | 0.1733 |
| rs41395344 | 8q21.11 | 76214041 | C | 0.66 | 0.56-0.79 | 5.79E-06 | ^d^ | ?--- | 0.2545 |
| rs11105956 | 12q21.33 | 91447523 | C | 0.74 | 0.65-0.85 | 5.87E-06 |  | --?- | 0.8011 |
| rs1249910 | 3q13.2 | 112391174 | T | 0.72 | 0.62-0.84 | 7.66E-06 | ^d^ | -+-- | 0.0332 |
| rs2243480 | 7q11.21 | 65599196 | T | 0.71 | 0.61-0.83 | 8.47E-06 |  | ?--- | 0.1487 |
| rs6538254 | 12q21.33 | 91344988 | T | 0.77 | 0.69-0.86 | 8.51E-06 |  | --?- | 0.7927 |
| rs4652753 | 1q25.3 | 182947346 | T | 0.82 | 0.75-0.91 | 9.14E-06 |  | ---- | 0.4108 |
| rs1677894 | 12q21.2 | 78337936 | G | 1.44 | 1.22-1.70 | 9.38E-06 |  | ?+++ | 0.7705 |
| rs12048692 | 1q25.3 | 182939070 | A | 0.82 | 0.74-0.90 | 9.42E-06 |  | ---- | 0.5011 |
| rs12219473 | 10p12.2 | 24564139 | T | 1.58 | 1.29-1.93 | 9.61E-06 |  | ++?? | 0.7908 |
| rs2596230 | 15q14 | 33720726 | G | 1.36 | 1.17-1.59 | 9.66E-06 |  | +?++ | 0.2354 |
| rs12099807 | 12p13.32 | 5130354 | T | 1.28 | 1.14-1.44 | 9.73E-06 |  | ++++ | 0.6678 |
| rs948612 | 18q22.3 | 68783000 | T | 0.81 | 0.74-0.89 | 1.02E-05 |  | ---- | 0.6918 |
| rs936589 | 4p14 | 37359427 | T | 1.26 | 1.12-1.41 | 1.03E-05 |  | ++++ | 0.0668 |
| rs6841655 | 4p14 | 37358920 | T | 1.26 | 1.12-1.41 | 1.04E-05 |  | ++++ | 0.0596 |
| rs12884663 | 14q32.2 | 97517316 | A | 0.74 | 0.65-0.85 | 1.06E-05 | ^d^ | ---- | 0.9722 |
| rs11089653 | 22q12.3 | 34698979 | T | 0.38 | 0.25-0.60 | 1.10E-05 | ^r^ | -?-? | 0.8238 |
| rs12544686 | 8q21.11 | 76218074 | T | 1.25 | 1.13-1.37 | 1.25E-05 |  | ++++ | 0.1946 |
| rs4718317 | 7q11.21 | 65648901 | A | 0.72 | 0.62-0.84 | 1.27E-05 |  | ?--- | 0.1908 |
| rs2176823 | 12q23.2 | 103547606 | C | 0.64 | 0.50-0.82 | 1.28E-05 | ^d^ | ---- | 0.2089 |
| rs2021779 | 7q32.3 | 132127963 | A | 1.43 | 1.22-1.68 | 1.32E-05 | ^d^ | ++++ | 0.2614 |
| rs286268 | 19p13.2 | 11839011 | T | 1.35 | 1.18-1.53 | 1.39E-05 | ^d^ | ++++ | 0.5128 |
| rs1252269 | 12q14.3 | 67262772 | A | 1.55 | 1.27-1.88 | 1.45E-05 | ^r^ | +??? | 1.0000 |
| rs2241733 | 7q32.3 | 132130033 | A | 1.44 | 1.23-1.68 | 1.46E-05 | ^d^ | ++++ | 0.1830 |
| rs2271934 | 12q23.2 | 103553204 | C | 0.65 | 0.50-0.83 | 1.46E-05 | ^d^ | ---- | 0.2417 |
| rs4879670 | 9p13.3 | 33205408 | G | 1.31 | 1.15-1.50 | 1.48E-05 | ^d^ | ++-+ | 0.2047 |
| rs7522372 | 1q25.3 | 182942845 | A | 0.83 | 0.75-0.91 | 1.55E-05 |  | ---- | 0.5416 |
| rs12692787 | 2q24.3 | 166754152 | T | 1.23 | 1.13-1.35 | 1.71E-05 |  | ++++ | 0.0908 |
| rs316329 | 7q11.21 | 65608416 | C | 0.73 | 0.62-0.85 | 1.77E-05 |  | ?--- | 0.2423 |
| rs7146783 | 14q24.2 | 73024003 | C | 1.68 | 1.34-2.11 | 1.83E-05 | ^r^ | ++++ | 0.1690 |
| rs1536792 | 9p23 | 12972382 | T | 0.66 | 0.54-0.81 | 1.85E-05 | ^d^ | ?--- | 0.2375 |
| rs383402 | 7q11.21 | 65586653 | A | 0.73 | 0.63-0.85 | 1.88E-05 |  | ?--- | 0.1279 |
| rs11065374 | 12q24.31 | 121400205 | G | 0.83 | 0.76-0.90 | 1.93E-05 |  | ---- | 0.4728 |
| rs890419 | 11p15.4 | 4410469 | T | 1.22 | 1.11-1.34 | 2.08E-05 |  | ++++ | 0.1284 |
| rs12641075 | 4q32.2 | 163874652 | A | 1.37 | 1.18-1.58 | 2.12E-05 | ^d^ | ++++ | 0.6876 |
| rs1948607 | 4q12 | 55706827 | A | 1.45 | 1.21-1.73 | 2.13E-05 | ^r^ | ++++ | 0.6431 |
| rs17421687 | 12p11.21 | 31454562 | T | 0.42 | 0.28-0.64 | 2.15E-05 | ^d^ | -?-- | 0.8642 |
| rs13238247 | 7q22.3 | 106160902 | T | 0.83 | 0.76-0.91 | 2.26E-05 |  | ---- | 0.9938 |
| rs4411336 | 12q12 | 40069333 | C | 1.27 | 1.14-1.41 | 2.27E-05 |  | ++++ | 0.4767 |
| rs6501053 | 16p13.2 | 8062273 | G | 0.68 | 0.57-0.81 | 2.27E-05 |  | -??? | 1.0000 |
| rs7093473 | 10q24.33 | 105535331 | C | 0.54 | 0.38-0.78 | 2.30E-05 | ^d^ | ---- | 0.7762 |
| rs10498963 | 6q15 | 89272006 | C | 0.42 | 0.29-0.63 | 2.38E-05 | ^r^ | ---- | 0.7341 |
| rs17141617 | 10p12.33 | 17775259 | G | 0.58 | 0.46-0.75 | 2.40E-05 | ^d^ | -??- | 0.2724 |
| rs10758197 | 9p13.3 | 33208034 | A | 1.29 | 1.13-1.47 | 2.41E-05 | ^d^ | ++-+ | 0.1816 |
| rs7715214 | 5q34 | 160626657 | T | 0.68 | 0.54-0.85 | 2.42E-05 | ^d^ | -?-- | 0.1970 |
| rs10510947 | 3p14.1 | 65829344 | G | 1.45 | 1.22-1.73 | 2.42E-05 | ^d^ | ++++ | 0.6373 |
| rs16946009 | 12q24.21 | 116098300 | G | 0.77 | 0.67-0.87 | 2.43E-05 |  | --?- | 0.1972 |
| rs1912709 | 8q21.11 | 76178563 | C | 1.91 | 1.40-2.60 | 2.45E-05 | ^r^ | ?+++ | 0.9526 |
| rs10784034 | 12q12 | 40130740 | T | 1.27 | 1.13-1.41 | 2.48E-05 |  | ++++ | 0.3862 |
| rs11107504 | 12q21.2 | 78336732 | A | 1.42 | 1.20-1.67 | 2.53E-05 |  | ?+++ | 0.8251 |
| rs12110644 | 6p24.3 | 10220826 | G | 0.81 | 0.72-0.91 | 2.55E-05 |  | -++- | 0.0142 |
| rs11107616 | 12q21.2 | 78364780 | C | 1.42 | 1.20-1.69 | 2.55E-05 |  | ?+++ | 0.9815 |
| rs17598593 | 11p14.1 | 28761974 | G | 2.06 | 1.47-2.88 | 2.60E-05 | ^d^ | +?++ | 0.8870 |
| rs7798078 | 7q35 | 147048920 | A | 0.64 | 0.51-0.82 | 2.70E-05 | ^r^ | ---- | 0.6332 |
| rs13292141 | 9p21.1 | 28602289 | A | 0.55 | 0.39-0.79 | 2.72E-05 | ^d^ | ---- | 0.1647 |
| rs1329036 | 9p21.3 | 23829911 | T | 0.64 | 0.52-0.78 | 2.72E-05 | ^d^ | --?- | 0.6345 |
| rs12297321 | 12q13.11 | 47109387 | T | 0.69 | 0.59-0.82 | 2.74E-05 | ^d^ | ---- | 0.0576 |
| rs3920455 | 12q21.33 | 91452184 | G | 0.77 | 0.68-0.87 | 2.75E-05 |  | --?- | 0.9422 |
| rs10197120 | 2q34 | 214273906 | C | 0.73 | 0.64-0.85 | 2.77E-05 |  | -?-- | 0.4792 |
| rs2526614 | 7p21.1 | 19064020 | G | 0.73 | 0.63-0.85 | 2.86E-05 | ^d^ | ---- | 0.9808 |
| rs10807702 | 7q11.21 | 65767843 | T | 0.74 | 0.63-0.86 | 2.86E-05 |  | ?--- | 0.2632 |
| rs11657125 | 17q24.2 | 66767551 | C | 0.83 | 0.75-0.91 | 2.91E-05 |  | ---- | 0.3421 |
| rs734704 | 8p23.1 | 6388306 | A | 0.69 | 0.57-0.82 | 2.91E-05 | ^r^ | ---- | 0.5431 |
| rs11107562 | 12q21.2 | 78355782 | C | 1.43 | 1.20-1.69 | 2.93E-05 |  | ?+++ | 0.9646 |
| rs1499614 | 7q11.21 | 65730798 | T | 0.73 | 0.63-0.86 | 2.99E-05 |  | ?--- | 0.2583 |
| rs891382 | 4q31.22 | 147152340 | C | 1.36 | 1.18-1.57 | 3.00E-05 |  | +?++ | 0.2074 |
| rs11029890 | 11p15.5 | 1493609 | T | 0.68 | 0.56-0.82 | 3.10E-05 | ^r^ | --0- | 0.4126 |
| rs6432852 | 2q24.3 | 166754553 | G | 0.82 | 0.75-0.90 | 3.17E-05 |  | ---- | 0.7771 |
| rs4784500 | 16q12.2 | 55192543 | C | 0.64 | 0.50-0.82 | 3.18E-05 | ^r^ | ---- | 0.0306 |
| rs1479100 | 8q21.11 | 76083241 | A | 1.25 | 1.12-1.39 | 3.25E-05 |  | ++++ | 0.1207 |
| rs17101762 | 10q23.1 | 85094792 | G | 1.57 | 1.27-1.95 | 3.42E-05 |  | +??+ | 0.6317 |
| rs2737426 | 8q24.22 | 134486658 | G | 0.72 | 0.62-0.83 | 3.45E-05 | ^d^ | ---- | 0.1275 |
| rs734702 | 8p23.1 | 6388213 | A | 0.69 | 0.57-0.82 | 3.47E-05 | ^r^ | ---- | 0.5396 |
| rs9324623 | 5q32 | 149006335 | C | 1.40 | 1.19-1.64 | 3.51E-05 | ^r^ | ++++ | 0.8663 |
| rs10199236 | 2q24.3 | 166794411 | G | 1.23 | 1.12-1.34 | 3.52E-05 |  | ++++ | 0.1034 |
| rs1997065 | 10q25.1 | 106763629 | G | 0.63 | 0.50-0.79 | 3.54E-05 | ^d^ | -?-- | 0.5886 |
| rs316327 | 7q11.21 | 65609201 | G | 0.74 | 0.63-0.86 | 3.63E-05 |  | ?--- | 0.2944 |
| rs215389 | 12q13.11 | 47764433 | T | 0.69 | 0.58-0.82 | 3.81E-05 |  | ??-- | 0.9652 |
| rs7312279 | 12q21.1 | 73701937 | C | 1.20 | 1.09-1.31 | 3.82E-05 |  | ++++ | 0.9346 |
| rs1488982 | 2q34 | 214227945 | C | 0.73 | 0.63-0.85 | 3.85E-05 |  | -?-- | 0.3902 |
| rs12711878 | 2q14.1 | 118182554 | G | 0.83 | 0.76-0.90 | 3.86E-05 |  | ---- | 0.8066 |
| rs1187046 | 10p11.22 | 33300985 | G | 0.75 | 0.65-0.88 | 3.93E-05 | ^d^ | ---- | 0.6226 |
| rs7037941 | 9p21.3 | 20536297 | C | 0.75 | 0.66-0.86 | 3.94E-05 | ^d^ | ---- | 0.5619 |
| rs7174138 | 15q12 | 27703715 | G | 1.24 | 1.12-1.37 | 4.06E-05 |  | ++++ | 0.5072 |
| rs7295119 | 12q21.1 | 73697698 | G | 1.19 | 1.09-1.30 | 4.08E-05 |  | ++++ | 0.8832 |
| rs270289 | 6q25.2 | 153742076 | T | 0.54 | 0.41-0.72 | 4.17E-05 | ^r^ | ---- | 0.6344 |
| rs16949855 | 16q12.1 | 51540509 | T | 0.47 | 0.33-0.68 | 4.27E-05 | ^r^ | -??? | 1.0000 |
| rs12797187 | 11q24.2 | 127447330 | T | 0.67 | 0.55-0.81 | 4.31E-05 | ^d^ | ---- | 0.9327 |
| rs13140757 | 4q34.3 | 180077942 | A | 1.25 | 1.13-1.38 | 4.33E-05 |  | ++++ | 0.1003 |
| rs13078932 | 3p25.3 | 10896829 | C | 2.38 | 1.58-3.61 | 4.34E-05 | ^r^ | +?+? | 0.4252 |
| rs7948119 | 11p15.1 | 20650119 | T | 1.83 | 1.37-2.44 | 4.43E-05 | ^d^ | +??? | 1.0000 |
| rs10840257 | 11p15.4 | 9568463 | C | 1.22 | 1.09-1.36 | 4.45E-05 |  | +?++ | 0.2165 |
| rs12405272 | 1q41 | 219842739 | T | 4.67 | 2.23-9.80 | 4.45E-05 | ^r^ | ??+? | 1.0000 |
| rs280565 | 9q21.13 | 76051882 | A | 0.70 | 0.55-0.90 | 4.48E-05 | ^d^ | --+- | 0.3525 |
| rs1078034 | 10p11.22 | 33312997 | A | 0.75 | 0.64-0.87 | 4.51E-05 | ^d^ | ---- | 0.5694 |
| rs16867595 | 8q22.3 | 102399034 | T | 1.31 | 1.13-1.52 | 4.53E-05 | ^d^ | ++++ | 0.6643 |
| rs2016141 | 12p13.32 | 5146003 | C | 1.31 | 1.15-1.49 | 4.54E-05 |  | ++++ | 0.8365 |
| rs6555005 | 5p15.1 | 17443266 | T | 0.76 | 0.66-0.87 | 4.54E-05 |  | -?-- | 0.5760 |
| rs6806547 | 3p14.1 | 63893477 | G | 1.35 | 1.16-1.58 | 4.61E-05 |  | ?+++ | 0.7360 |
| rs1382334 | 5q34 | 160652367 | A | 0.65 | 0.53-0.80 | 4.62E-05 |  | -??? | 1.0000 |
| rs11744603 | 5q11.2 | 53611730 | A | 0.52 | 0.38-0.71 | 4.65E-05 | ^d^ | ??-- | 0.7999 |
| rs6599130 | 3p22.1 | 41025167 | C | 0.76 | 0.66-0.87 | 4.72E-05 | ^d^ | ---- | 0.6100 |
| rs1659885 | 2q12.3 | 108433169 | A | 0.72 | 0.60-0.86 | 4.72E-05 | ^r^ | ---- | 0.9129 |
| rs16934303 | 11p12 | 40085949 | T | 0.45 | 0.31-0.66 | 4.79E-05 | ^r^ | -??? | 1.0000 |
| rs4881740 | 11p15.5 | 1519981 | T | 0.64 | 0.51-0.80 | 4.80E-05 | ^r^ | ---- | 0.9511 |
| rs17469788 | 14q12 | 32483947 | T | 1.32 | 1.16-1.51 | 4.85E-05 |  | +?++ | 0.9821 |
| rs1589921 | 15q11.2 | 23750045 | A | 1.65 | 1.30-2.10 | 4.85E-05 |  | +??? | 1.0000 |
| rs11071505 | 15q22.2 | 60471417 | G | 1.40 | 1.19-1.64 | 4.90E-05 |  | +??? | 1.0000 |
| rs1997522 | 5q11.2 | 55651577 | T | 0.71 | 0.57-0.90 | 4.91E-05 | ^d^ | ---- | 0.6735 |
| rs2796292 | 10p11.23 | 29606383 | T | 1.61 | 1.27-2.05 | 4.95E-05 | ^r^ | ?+++ | 0.9236 |
| rs2707832 | 7q11.21 | 66136549 | T | 0.74 | 0.64-0.86 | 5.13E-05 |  | ?--- | 0.2955 |
| rs10056782 | 5q32 | 146400490 | T | 0.76 | 0.66-0.88 | 5.19E-05 | ^r^ | ---- | 0.4177 |
| rs4837240 | 9q34.11 | 130873428 | A | 1.31 | 1.14-1.50 | 5.37E-05 | ^d^ | ++++ | 0.3725 |
| rs7335175 | 13q33.1 | 103488664 | G | 1.72 | 1.32-2.25 | 5.38E-05 |  | +??? | 1.0000 |
| rs2737424 | 8q24.22 | 134486021 | G | 0.72 | 0.62-0.84 | 5.42E-05 | ^d^ | ---- | 0.3889 |
| rs316331 | 7q11.21 | 65604622 | A | 0.76 | 0.66-0.87 | 5.49E-05 |  | ---- | 0.0139 |
| rs2191201 | 12p13.32 | 5146857 | A | 1.31 | 1.15-1.49 | 5.51E-05 |  | ++++ | 0.8232 |
| rs7784712 | 7p21.1 | 19059890 | A | 1.49 | 1.24-1.78 | 5.56E-05 | ^d^ | +?++ | 0.2430 |
| rs6421196 | 12q12 | 40085007 | C | 1.28 | 1.14-1.43 | 5.66E-05 |  | ++++ | 0.3432 |
| rs12047601 | 1q25.3 | 182957178 | C | 0.83 | 0.75-0.92 | 5.72E-05 |  | ---- | 0.4440 |
| rs11009147 | 10p11.22 | 33211227 | C | 0.74 | 0.63-0.88 | 5.82E-05 | ^d^ | ---- | 0.8903 |
| rs734701 | 8p23.1 | 6388247 | C | 0.70 | 0.58-0.84 | 5.83E-05 | ^r^ | ---- | 0.5378 |
| rs1263619 | 2q33.3 | 207954639 | T | 0.65 | 0.53-0.79 | 5.84E-05 | ^d^ | ---- | 0.2190 |
| rs7199677 | 16q23.3 | 82945551 | C | 0.79 | 0.69-0.89 | 5.89E-05 | ^d^ | ---- | 0.9151 |
| rs7332267 | 13q21.33 | 72509652 | C | 1.25 | 1.12-1.39 | 5.93E-05 |  | ++++ | 0.6786 |
| rs13384459 | 2q34 | 214183769 | T | 0.74 | 0.64-0.86 | 5.95E-05 |  | -?-- | 0.5282 |
| rs2026718 | 9p23 | 12964715 | G | 0.72 | 0.61-0.85 | 5.96E-05 | ^d^ | ---- | 0.6811 |
| rs10475891 | 5q34 | 168212096 | C | 1.26 | 1.12-1.41 | 5.96E-05 |  | ++++ | 0.6512 |
| rs7214746 | 17q11.2 | 27537626 | C | 1.70 | 1.31-2.20 | 5.99E-05 |  | +??? | 1.0000 |
| rs17362920 | 2q34 | 214118499 | C | 0.74 | 0.64-0.86 | 6.01E-05 |  | -?-- | 0.3087 |
| rs13238478 | 7q11.21 | 66388082 | C | 0.75 | 0.64-0.87 | 6.04E-05 |  | ?--- | 0.3324 |
| rs11077899 | 17q25.3 | 75306821 | G | 1.82 | 1.26-2.62 | 6.05E-05 | ^r^ | +??+ | 0.0219 |
| rs841160 | 1q44 | 247912691 | G | 1.57 | 1.19-2.08 | 6.07E-05 | ^d^ | +??+ | 0.6214 |
| rs12489378 | 3p14.2 | 63298608 | A | 1.18 | 1.06-1.32 | 6.07E-05 |  | +-++ | 0.1071 |
| rs976164 | 9p13.3 | 33204104 | G | 1.20 | 1.09-1.31 | 6.16E-05 |  | ++-+ | 0.0698 |
| rs10492818 | 16q22.2 | 72316884 | T | 2.32 | 1.54-3.51 | 6.21E-05 | ^r^ | +??? | 1.0000 |
| rs11655764 | 17q21.31 | 43836477 | T | 0.77 | 0.68-0.88 | 6.29E-05 | ^d^ | ---- | 0.8897 |
| rs1969588 | 7q31.1 | 112682515 | C | 0.43 | 0.28-0.65 | 6.29E-05 | ^r^ | -??? | 1.0000 |
| rs12064551 | 1q32.1 | 203924763 | T | 2.79 | 1.43-5.43 | 6.30E-05 | ^d^ | +??+ | 0.7757 |
| rs4495951 | 12q12 | 40099914 | A | 1.27 | 1.14-1.43 | 6.40E-05 |  | ++++ | 0.2904 |
| rs2490486 | 10p11.22 | 32716257 | T | 0.77 | 0.67-0.88 | 6.51E-05 |  | -??- | 0.8671 |
| rs233244 | 21q22.3 | 45973402 | C | 1.25 | 1.12-1.41 | 6.53E-05 |  | ++++ | 0.7431 |
| rs2619051 | 12q21.2 | 78382236 | T | 1.30 | 1.15-1.46 | 6.56E-05 |  | ++++ | 0.4104 |
| rs17708094 | 16q23.1 | 78677032 | C | 0.59 | 0.46-0.77 | 6.69E-05 | ^d^ | -?-- | 0.7879 |
| rs1323590 | 9p24.1 | 8455942 | T | 0.75 | 0.65-0.86 | 6.77E-05 | ^d^ | ---- | 0.4957 |
| rs1996294 | 2q14.1 | 118162097 | G | 0.72 | 0.62-0.84 | 6.79E-05 | ^d^ | ---- | 0.5826 |
| rs12574699 | 11q22.3 | 103683034 | T | 0.78 | 0.69-0.89 | 6.81E-05 |  | -?-- | 0.7353 |
| rs6582145 | 12q21.1 | 73735491 | A | 1.19 | 1.09-1.30 | 6.84E-05 |  | ++++ | 0.8225 |
| rs1997066 | 10q25.1 | 106763563 | C | 0.59 | 0.45-0.77 | 6.85E-05 | ^d^ | -?-- | 0.5840 |
| rs12196229 | 6q27 | 169107058 | C | 1.40 | 1.19-1.66 | 6.93E-05 |  | +?++ | 0.6722 |
| rs996829 | 16p12.1 | 26323436 | C | 1.30 | 1.09-1.57 | 7.02E-05 | ^d^ | ++++ | 0.4740 |
| rs1108991 | 11p15.5 | 1537517 | C | 0.65 | 0.52-0.81 | 7.04E-05 | ^r^ | ---- | 0.9396 |
| rs13012468 | 2p25.3 | 2215203 | G | 1.86 | 1.35-2.55 | 7.21E-05 | ^d^ | +?++ | 0.5515 |
| rs17694142 | 2q34 | 214269942 | C | 0.75 | 0.65-0.86 | 7.24E-05 |  | -?-- | 0.5739 |
| rs327215 | 8p21.2 | 26492962 | C | 1.52 | 1.24-1.87 | 7.25E-05 |  | +??? | 1.0000 |
| rs9869659 | 3q23 | 141640396 | T | 0.70 | 0.58-0.84 | 7.28E-05 | ^r^ | ---- | 0.5730 |
| rs1488981 | 2q34 | 214228020 | G | 0.74 | 0.63-0.86 | 7.45E-05 |  | -?-- | 0.4174 |
| rs7862066 | 9p22.3 | 14880104 | G | 1.55 | 1.20-2.00 | 7.50E-05 | ^r^ | ++++ | 0.6079 |
| rs10846428 | 12p12.3 | 16954133 | G | 0.74 | 0.64-0.86 | 7.51E-05 | ^d^ | ---- | 0.8780 |
| rs12490808 | 3p13 | 72315369 | T | 0.76 | 0.67-0.87 | 7.52E-05 | ^d^ | ---- | 0.1429 |
| rs744985 | 9p13.2 | 37392087 | C | 0.80 | 0.72-0.89 | 7.53E-05 |  | ---- | 0.2168 |
| rs11107763 | 12q21.2 | 78391958 | C | 1.40 | 1.18-1.65 | 7.61E-05 |  | ?+++ | 0.9792 |
| rs16937581 | 10q21.1 | 55333463 | A | 0.60 | 0.45-0.82 | 7.62E-05 | ^d^ | ---- | 0.8060 |
| rs16892547 | 6q26 | 161886037 | G | 1.49 | 1.22-1.82 | 7.63E-05 | ^r^ | +??? | 1.0000 |
| rs10200680 | 2q36.1 | 223961877 | A | 1.31 | 1.08-1.59 | 7.65E-05 | ^d^ | ++++ | 0.1903 |
| rs12638260 | 3p24.3 | 20402065 | C | 0.77 | 0.68-0.88 | 7.71E-05 |  | -?-- | 0.4700 |
| rs3860587 | 3p24.3 | 21886008 | G | 0.58 | 0.43-0.76 | 7.78E-05 | ^r^ | ?--- | 0.6176 |
| rs4666960 | 2q32.1 | 184997066 | T | 0.48 | 0.32-0.72 | 7.78E-05 | ^d^ | -??- | 0.5052 |
| rs4748042 | 10p13 | 13554693 | G | 0.56 | 0.42-0.74 | 7.81E-05 | ^d^ | -??? | 1.0000 |
| rs10959331 | 9p24.3 | 1095101 | C | 0.57 | 0.42-0.76 | 7.86E-05 | ^d^ | ---- | 0.2783 |
| rs10756436 | 9p23 | 12967249 | C | 0.69 | 0.57-0.83 | 7.86E-05 |  | ?-?- | 0.9419 |
| rs1187087 | 10p11.22 | 33281622 | C | 0.78 | 0.68-0.90 | 7.93E-05 | ^d^ | ---- | 0.6816 |
| rs12458786 | 18q11.2 | 23030650 | A | 0.71 | 0.60-0.84 | 7.93E-05 |  | ?-?- | 0.3049 |
| rs1556990 | 10p13 | 13550822 | A | 0.57 | 0.43-0.75 | 7.97E-05 |  | -??? | 1.0000 |
| rs10185045 | 2q34 | 214117303 | G | 0.75 | 0.65-0.87 | 8.01E-05 |  | -?-- | 0.4364 |
| rs12599351 | 16q24.1 | 86024616 | G | 0.53 | 0.38-0.73 | 8.02E-05 | ^r^ | ??-- | 0.9883 |
| rs2397051 | 6p12.3 | 51435344 | T | 1.54 | 1.26-1.88 | 8.07E-05 | ^d^ | ++++ | 0.3766 |
| rs17464499 | 22q12.1 | 27891715 | G | 0.62 | 0.49-0.79 | 8.09E-05 | ^d^ | -?-- | 0.4668 |
| rs2161058 | 3q28 | 190294290 | T | 1.97 | 1.31-2.97 | 8.15E-05 | ^d^ | +??+ | 0.8535 |
| rs4748043 | 10p13 | 13556487 | A | 0.56 | 0.42-0.75 | 8.19E-05 | ^d^ | -??? | 1.0000 |
| rs17098034 | 1p31.1 | 76532801 | G | 1.38 | 1.17-1.63 | 8.20E-05 | ^d^ | ++?+ | 0.9304 |
| rs6096030 | 20q13.13 | 49237508 | A | 0.20 | 0.09-0.44 | 8.33E-05 | ^r^ | ?-?? | 1.0000 |
| rs2836992 | 21q22.2 | 40735606 | T | 1.55 | 1.24-1.94 | 8.36E-05 | ^r^ | ++++ | 0.5990 |
| rs6030341 | 20q12 | 41207365 | A | 0.76 | 0.67-0.87 | 8.39E-05 | ^d^ | ---- | 0.6187 |
| rs10828859 | 10p12.31 | 18822342 | C | 0.79 | 0.70-0.90 | 8.44E-05 | ^d^ | ---- | 0.1802 |
| rs331124 | 2q23.3 | 150870860 | G | 0.68 | 0.56-0.83 | 8.44E-05 | ^d^ | -?-- | 0.4516 |
| rs1427074 | 8q21.13 | 82250613 | A | 2.46 | 1.63-3.70 | 8.50E-05 | ^r^ | ++++ | 0.1708 |
| rs4895312 | 5q23.1 | 120849727 | C | 0.67 | 0.54-0.82 | 8.66E-05 |  | ??-- | 0.4417 |
| rs8085386 | 18q21.32 | 57589409 | A | 0.33 | 0.17-0.65 | 9.02E-05 | ^d^ | -??- | 0.9539 |
| rs6809448 | 3q23 | 142353730 | A | 0.58 | 0.44-0.76 | 9.09E-05 | ^r^ | -?+- | 0.1049 |
| rs1328183 | 1q31.2 | 193759244 | A | 0.71 | 0.60-0.84 | 9.17E-05 |  | -??? | 1.0000 |
| rs1762530 | 10p11.22 | 32966615 | T | 0.76 | 0.65-0.87 | 9.21E-05 |  | -??- | 0.7562 |

^1^ P-values shown are additive unless another model isdenoted next to the p-value (d=dominant model,r=recessive model).direction isread in the order: AA-AI-EA-MA; a “?”denotes that ethnicity’s data did not pass QC and was not included in the meta-analysis.

Direction: RA is reference allele. The odds ratio (OR) is presented for the reference allele, compared with the non-reference allele, for a given model.
