# Supplementary figures and images for: Genome-Wide Association and Trans-ethnic Meta-Analysis for Advanced Diabetic Kidney Disease: Family Investigation of Nephropathy and Diabetes (FIND)
Source: PLoS Genet. 2015 Aug 25;11(8):e1005352. doi: 10.1371/journal.pgen.1005352 (PMC4549309; doi:10.1371/journal.pgen.1005352)

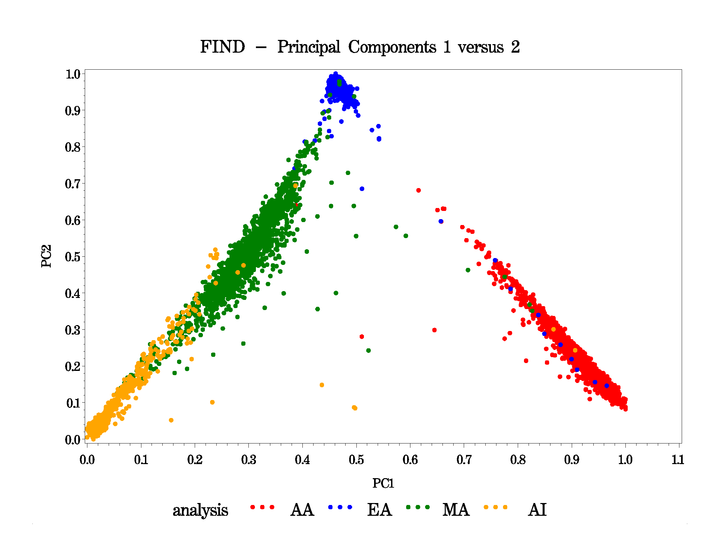

Supplement: S1 Fig — The principal component (PC) analysis identified PCs that genetically partitioned the Discovery sample into ancestry groups consistent with self-report. (TIFF) [file pgen.1005352.s010.tiff]

**Supplemental Figure S2.**


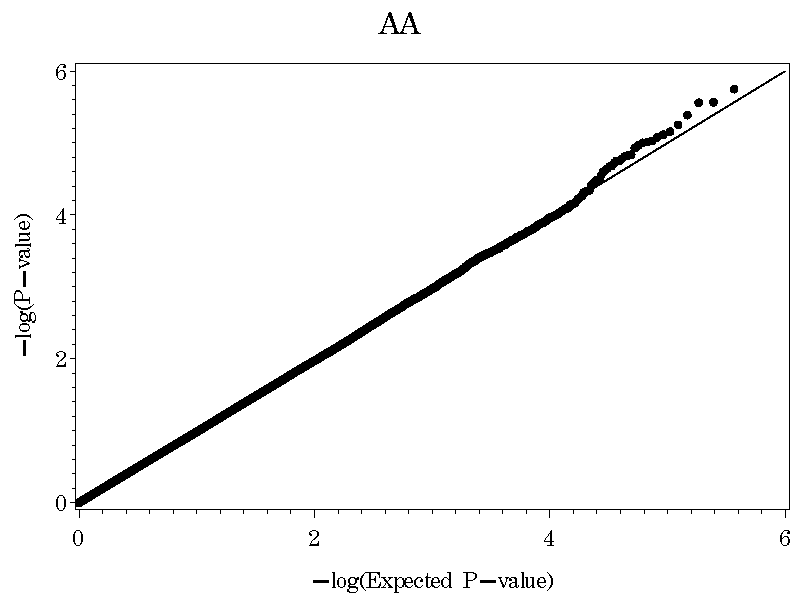

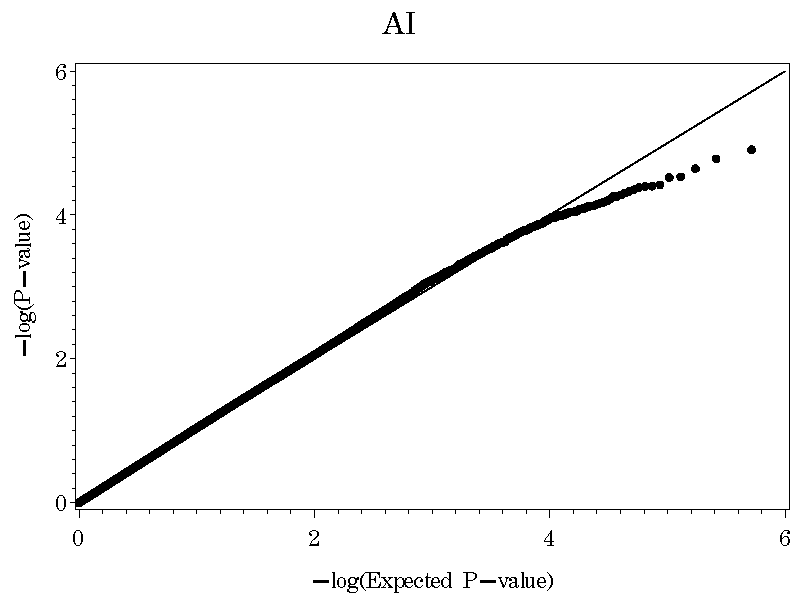

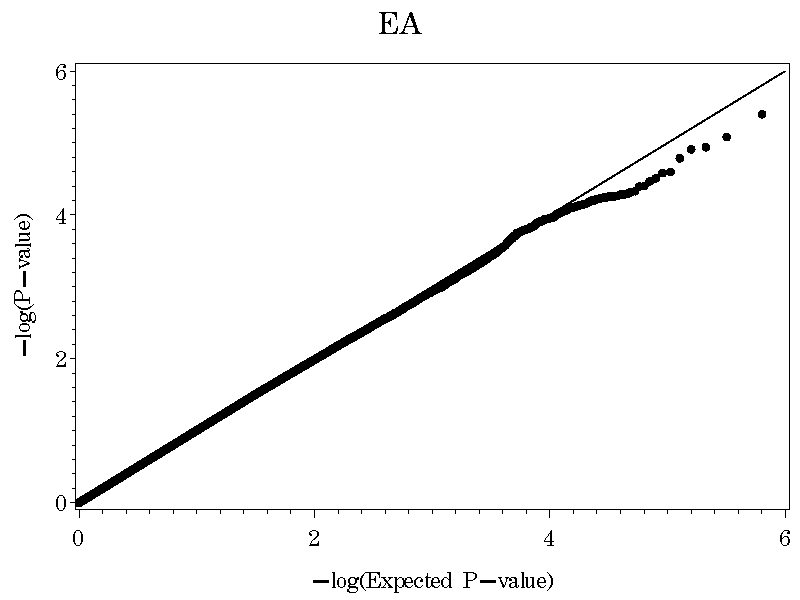

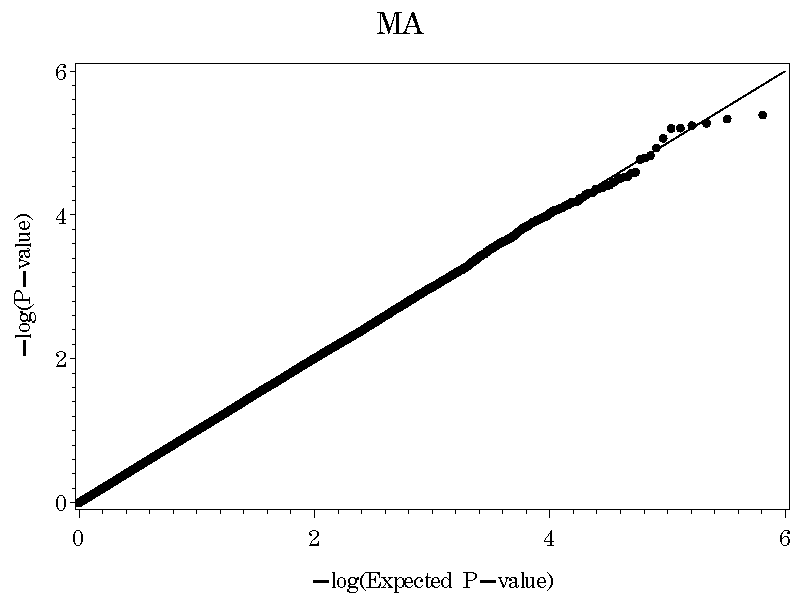

Supplement: S2 Fig — The logistic regression model, which included the PCs as covariates, reduced the inflation factor to nominal levels and combined with the P-P plot show no evidence of a systematic inflation. (DOCX) [file pgen.1005352.s011.docx]

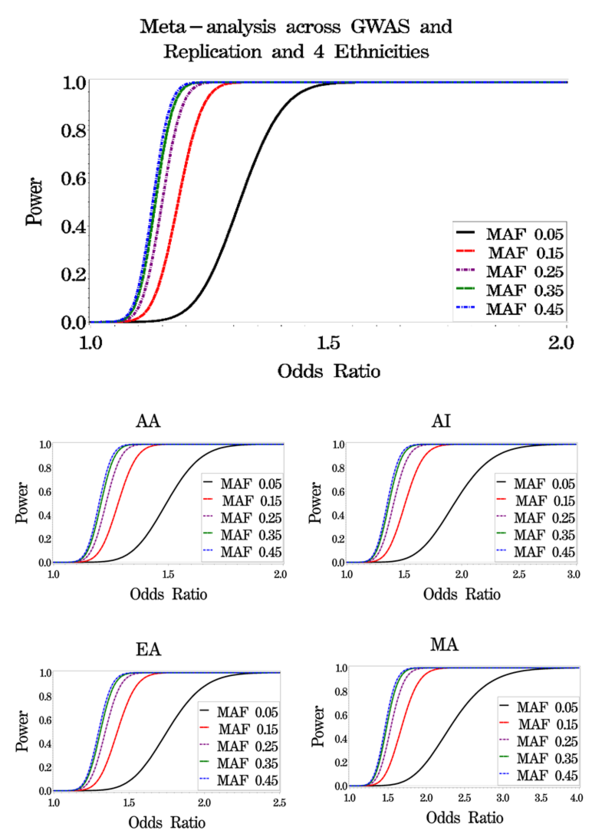

Supplement: S3 Fig — To estimate power, unmatched case and control subjects from Discovery plus Replication [FILR] included: AA: 2514 cases, 3594 controls; AI: 1009 cases, 1145 controls; EA: 924 cases, 3177 controls; MA: 779 cases, 594 controls (no Replication samples); and Meta-analysis: 5226 cases, 8510 controls. The following assumptions were used for power analysis: Additive Model; α = 1 x 10^−6^; and DM Population prevalence kp = 0.30. (TIFF) [file pgen.1005352.s012.tiff]

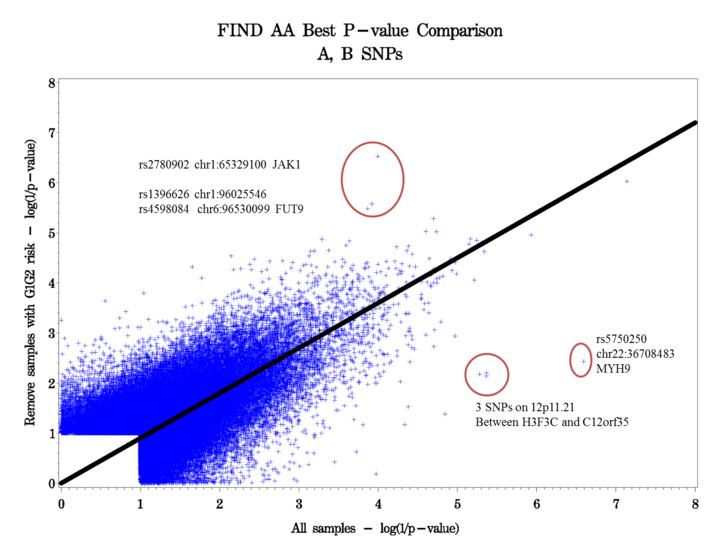

Supplement: S4 Fig — The correlation between the–log10(p-value) for GWAS with and with AA subjects with and without two APOL1 risk variants is r = 0.82. (TIFF) [file pgen.1005352.s013.tiff]

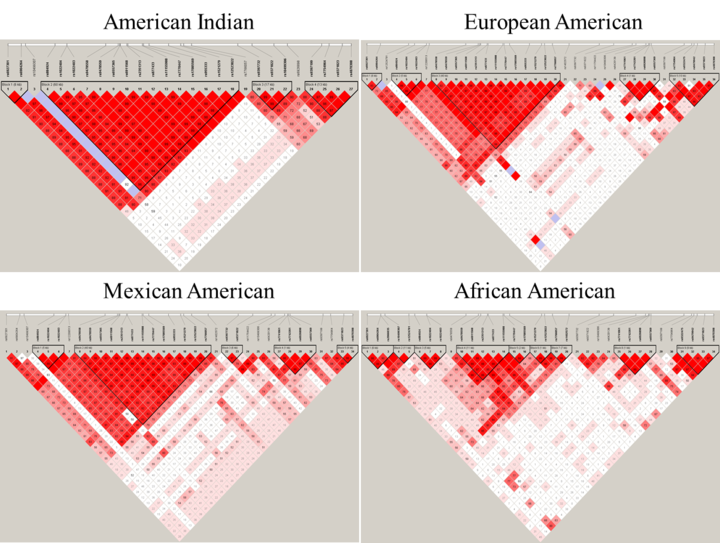

Supplement: S5 Fig — The SCAF8-CNKSR3 region shows extended linkage disequilibriums in all ethnicities but AA. (TIFF) [file pgen.1005352.s014.tiff]

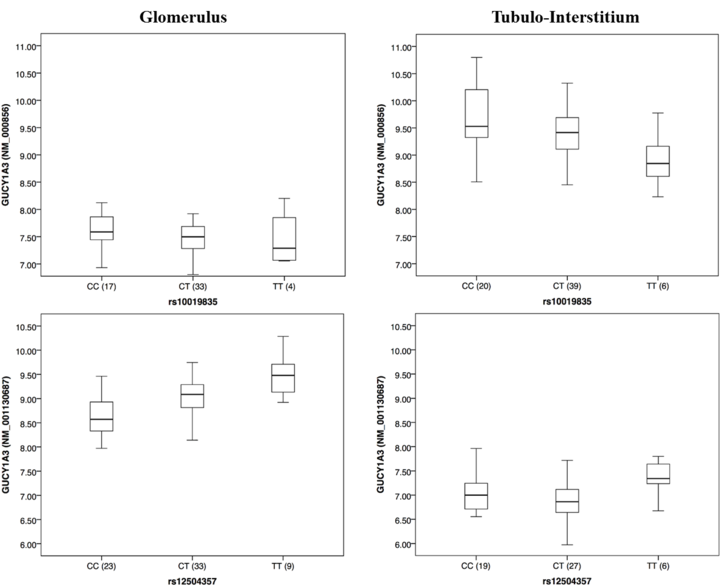

Supplement: S6 Fig — The full-length isoform NM_000856 has a tissue-specific tubulo-interstitial eQTL with AI GWAS candidate rs10019835 (P = 4.97 x 10^−4^, glomerulus not significant at p > 0.00024), and short isoform NM_001130687 having a glomerular eQTL with intronic SNP rs12504357 (P = 2.63 x 10^−5^, tubulo-interstitium not significant at p>0.05). Both isoforms satisfy the test for expression in both tissues. (TIFF) [file pgen.1005352.s015.tiff]
